# Deploying an asthma dashboard to support quality improvement across a nationally representative sentinel network of 7.6 million people in England

**DOI:** 10.1038/s41533-024-00377-8

**Published:** 2024-06-29

**Authors:** Mome Mukherjee, Cecilia Okusi, Gavin Jamie, Rachel Byford, Filipa Ferreira, Monica Fletcher, Simon de Lusignan, Aziz Sheikh

**Affiliations:** 1 Asthma UK Centre for Applied Research, Usher Institute, The University of Edinburgh, Edinburgh, UK; 2 HDR UK BREATHE Respiratory Data Hub, Usher Institute, The University of Edinburgh, Edinburgh, UK; 3 HDR UK Better Care, Usher Institute, The University of Edinburgh, Edinburgh, UK; 4 Clinical Informatics and Health Outcomes Research Group, Nuffield Department of Primary Care Health Sciences, University of Oxford, Oxford, UK; 5 Royal College of General Practitioners (RCGP) Research and Surveillance Centre (RSC), London, UK; 6 Nuffield Department of Primary Care Health Sciences, University of Oxford, Oxford, UK

**Keywords:** Health services, Asthma

## Abstract

Every year, there are ~100,000 hospital admissions for asthma in the UK, many of which are potentially preventable. Evidence suggests that carefully conceptualised and implemented audit and feedback (A&F) cycles have the potential to improve clinical outcomes for those with chronic conditions. We wanted to investigate the technical feasibility of developing a near-real time asthma dashboard to support A&F interventions for asthma management in primary care. We extracted cross-sectional data on asthma from 756 participating GP practices in the Oxford-Royal College of General Practitioners Research and Surveillance Centre (RCGP RSC) database in England comprising 7.6 million registered people. Summary indicators for a GP practice were compared to all participating RCGP RSC practices using practice-level data, for the week 6–12th-Mar-2023. A weekly, automated asthma dashboard with features that can support electronic-A&F cycles that compared key asthma indicators for a GP practice to RCGP RSC could be created (https://tinyurl.com/3ydtrt85): 12-weeks-incidence 0.4% vs 0.4%, annual prevalence 6.1% vs 6.7%, inhaled relievers to preventer 1.2 vs 1.1, self-management plan given 83.4% vs 60.8%, annual reviews 36.8% vs 57.3%, prednisolone prescriptions 2.0% vs 3.2%, influenza vaccination 56.6% vs 55.5%, pneumococcal vaccination ever (aged ≥65 years) 90.2% vs 84.1% and current smokers 14.9% vs 14.8%. Across the RCGP RSC, the rate of hospitalisations was 0.024%; comparative data had to be suppressed for the study practice because of small numbers. We have successfully created an automated near real-time asthma dashboard that can be used to support A&F initiatives to improve asthma care and outcomes in primary care.

## Introduction

Evidence-based guidelines for asthma management and effective medications are available to inform clinical practice. Yet thousands of people with asthma still suffer unnecessary symptoms and potentially preventable asthma exacerbations. There are around 4.3 million people with clinician-reported-diagnosed-and-treated asthma in the UK (6.8%), which result in nearly 100,000 hospital admissions, 1100 deaths per year and costs the UK public sector at least £1 billion^1^. Besides, asthma deaths increased by 33% between 2008 and 2018 in England and Wales^2^. This is despite the Quality and Outcomes Framework (QOF) implemented across the UK since 2004, whereby general practices were remunerated for providing good quality care, including for asthma. It is known that there can be better asthma outcomes by addressing clinical guideline adherence, guided self-management, and the provision of personal asthma action plans^3–6^. Implementations of asthma guidelines vary across regions and emerging evidence takes several years to translate into practice, with time and resources being the main barriers^7^. Thus, new approaches are needed, such as a Learning Health System, whereby data about patient care are turned into knowledge, and this is fed back into evidence-based clinical practice and trained healthcare professionals (HCP) can be assisted towards change in the health system^7,8^. Since asthma is largely managed in primary care in the UK, which has rich electronic healthcare data and a history of pioneering health information technology, there are excellent opportunities to transform clinical records into shareable knowledge rapidly with HCP. One such opportunity is to measure and monitor asthma management and outcomes using an audit and feedback (A&F) dashboard for asthma based on primary care records, which could be fed back to HCPs.

A&F is a widely employed quality improvement strategy to promote the implementation of evidence-based practices by providing recipients a summary of their monitoring over a specified period of time. A&F can provide objective data regarding discrepancies between current practice and target monitoring, comparing to other HCPs, and thus are an improvement over self-assessment or self-monitoring^9,10^.

Evidence from A&F literature are implemented in quality improvement (QI) exercises. However, measuring effectiveness is not straight-forward. A systematic review on the effectiveness of QOF, which also included three previous systematic reviews, reported that given the nature of evidence, they could not attribute if QOF was the causal factor for the modest reduction in increase in emergency admissions across diseases^11^. Visual analytics and dashboards are common QI tools. A review defined a dashboard as a performance measurement tool that queried electronic data and presented results in an easy-to-interpret graphical format^12^. While the literature on QI tools is developing, systematic reviews on clinical decision support systems (CDSS) in routine care of asthma reported that although CDSS were under-used, asthma outcomes improved when they were used^13^. They recommended better integration with routine workflow^13^. Furthermore, computerised alerts, when part of an integrated healthcare system, led to reductions in excessive prescribing of short-acting beta agonists (SABAs)^14^. Thus, they recommended other ways of motivating primary care^11^. A QI programme for persistent asthma care in USA through education, data feedback and sharing of best practices reported improvement in use of asthma action plans and asthma control tests and reductions in asthma exacerbations^15^. Another QI programme in USA between 2008 and 2016, which used a collaborative learning model among paediatricians, along with a monthly dashboard, reported significant improvement in seven out of eight clinical asthma management measures^16^.

Utilisation of dashboards has been ascribed to socio-cultural and technical factors. The socio-cultural factors were HCPs anxiety of electronic surveillance of their performance, information overload caused by monitoring and alerts and the use of indicators that were not considered particularly useful by HCPs^12^. Another review on dashboards across mixed healthcare settings, reported that there is some evidence that through providing constant information through clinical and/or QI dashboards, for example via screensavers, can improve quality adherence and patients’ outcomes but which characteristics improved outcomes were unclear^17^. Co-developing visualisation tools with interdisciplinary teams towards a human-centred design, which were flexible, could be adjusted to users’ preference, organised by body system and colour-coded, were found to improve efficiency, safety, quality, accuracy and satisfaction across healthcare settings^18^. In order to improve asthma outcomes and picking up the recommendations from the literature above, our objectives were to investigate if a dashboard: (a) could be created with near-real time primary-care data with an A&F loop providing tailored feedback for QI on asthma to general practices and their teams; and (b) can provide information on incidence and prevalence of asthma, modifiable risk factors and outcomes for asthma, comparing a GP practice to the average of other practices in the network.

## Methods

### Setting and participants

We used pseudonymised data from the primary care sentinel cohort (PCSC) in the Oxford-Royal College of General Practitioners (RCGP) Research and Surveillance Centre (RSC) database. These data were held in Oxford-RCGP Clinical Informatics Digital (ORCHID) Hub Trusted Research Environment (TRE)^19^, in England. A subset of these GP practices, the PCSC group of practices, undertake sentinel surveillance—virological and serological^20^. The number of GP practices in the PCSC group was 756 at the time of the study^20^. There are a further 1000 practices who provide data for the UK Health Security Agency’s (UKHSA) Syndromic Surveillance, which were not included in this study. These other practices are labelled Syndromic Surveillance General Practices (SSGPs)^20^. What is labelled as “RSC data” was really the PCSC of the RSC. Data were extracted from each practices electronic health record (EHR)^21^. Data were pseudonymised extract^22^. The extraction was being conducted for sentinel surveillance^20^, but reused for this study with ethical approval. The data utilised were either registration data, coded or numeric data associated with coded data. The processing to produce the dashboard just used counts and no personal data were extracted to do this. People who had codes suggesting they opted out of record sharing, were excluded from analyses^23^.

### Definitions

People with asthma were defined as anyone who was registered in RSC, who had a diagnosis of asthma by their GP and were prescribed with asthma medication in the last 12 months or if they had a hospital admission with asthma as their primary reason for admission recorded by the GP.

### Measures of interest

The measures of interest were:

i. incidence and prevalence of asthma diagnosis,
ii. asthma modifiable factors - asthma self-management plan given, asthma review, ratio of inhaled reliever to preventer medications, influenza vaccination, pneumococcal polysaccharide vaccination (PPV) ever given in 65-year-olds-and-above (to prevent invasive pneumococcal disease, example septicaemia, pneumonia and meningitis)^24,25^, smoking status – active smoker,
iii. asthma exacerbation events: GP-recorded asthma exacerbations, GP’s prescriptions of prednisolone, GP-recorded accident and emergency (A&E) department attendance or hospital admissions for asthma.

### Dashboard

The dashboard was created for all the PCSC practices in RSC. It was built in a style used across our RSC dashboards, though was not promoted to them. A near-real time automated asthma dashboard displaying weekly data, accessible over the web, was created which compared individual GP practices to the average of other practices in the PCSC-RSC network, on asthma incidence and prevalence and quantified asthma modifiable risk factors and outcomes (https://public.tableau.com/views/Asthma_MyPracticeDashboard/Asthma?:language=en-GB&:display_count=n&:origin=viz_share_link&:showVizHome=n&:toolbar=no). The dashboard was developed to sit on top of a pseudonymised set of the practices’ routine data. Key performance indicators were identified (ii) that might be amenable to primary care interventions. We also identified key outcomes (iii). We studied the dashboard in detail in a single study practice. The study practice had an age-sex profile and sociodemographic distribution close to the English national average.

The way the dashboard was set up, these practices’ routine EHR data could be searched for i–iii. These searches produced counts in all the prespecified fields that are in the dashboard. These searches were run twice weekly across of all RSC practices in the PCSC. The dashboard was developed by consensus as a co-design process with representative GPs, researchers, and primary care colleagues. The generic design, development and deployment of the dashboards developed were discussed previously^26^. Its goal was to identify: (1) if incident cases were being identified (coded) and how this compared with the rest of the network (i), (2) if processes of care which might affect outcomes were being implemented (ii), and (3) to look at outcomes that might be coded into the GP records (iii).

GP practices can log in using their practice key and can select the week they want to view. There are visual icons, used as performance indicators that change depending on how the practice compared with the RSC average in every section and overall.

### Study period

The data went back till International Organization for Standardization (ISO) week-1 of 2019. People who were registered for at least 12 weeks before the ISO week start date were included, except for influenza vaccination where that restriction was not applied. The study index week was ISO week (Monday to Sunday) 10, 6–12th March for year 2023. A March week was used because asthma is part of an English national chronic disease management programme called the QOF. This has its year up to 31st March. Therefore, a March week would see data as good as it gets in the study and comparator practices.

### Near-real time

Near real time refers to the cycle of dashboard refresh. Data are refreshed on a Wednesday from up to the previous Saturday, and on a Friday that were collected on a Tuesday. The dashboard is at best five days in arrears. The feedback from practices was this was the interval that they wanted, as clinicians thought they might remember the details of an admission or exacerbation the previous week. Due to quality control measures in place, the asthma dashboard has a time lag of a week i.e. on Monday 27th March 2023, data available for the latest week was for the week Monday, March 13, 2023.

### Data analyses

Data reported were from observational data only and have not been part of any trial. Cross-sectional analyses were undertaken using Systematized Nomenclature of Medicine -clinical terms (SNOMED-CT) for asthma (Supplement 1)^27^. Structured query language (SQL) was used to process these data and Tableau was used to create the dashboard. Data processing was automated to present weekly data. An incident case of asthma was defined as a new case of ‘active asthma’ during the last 12 weeks before index week. Prevalence was provided by age-groups, as well as age-standardised. The number of asthma reviews conducted over the last 12 months preceding the index week was reported. Asthma self-management plans were for ISO week-1 in 2019 till end of index week. Since PPV vaccinations are given once to 65-year-olds-and-above^25^, data extraction were for any event dates until end of index week. Prescribed inhaled relievers, preventers and asthma exacerbation events were measured for the index week. Prednisolone prescriptions were found using NHS England’s Dictionary of Medicines and Devices (DM + D) codes. Counts of prednisolone prescriptions and GP-recorded asthma exacerbation events were within the last 4 weeks from index week, with GP-recorded hospital admissions and GP-recorded emergency department attendances +/– 7 days from either the prednisolone prescription issue date or asthma exacerbation event date. Since prednisolone may also have been prescribed on repeat, for asthma exacerbations, repeat prescriptions were excluded from the analysis. Annual asthma prevalence and influenza vaccination was summarised by age-groups. Summary measures of all other practices have been provided as RSC. Reporting of measures for a particular practice, called here as ‘study practice’ has been compared to all other participating practices combined (RSC). Missing data were excluded from analyses. Data are presented by numerator, denominator, percentages and lower and upper 95% confidence intervals and visualised in the dashboard. The denominators of asthma modifiable factors were people with asthma, for the respective age-groups where relevant. Ratio of inhaled relievers to preventers were presented with standard deviation (SD) and 95% CI. For data confidentiality, numbers less than 5 were withheld from reporting.

The REporting of studies Conducted using Observational Routinely-collected health Data (RECORD) statement (RECORD) guidelines have been followed for the reporting here^28^.

### Ethical approval

Patients and practices were informed of this study and the option available to them to ‘opt-out’ of sharing data. All current research activities using pseudonymised data from the RCGP RSC network of general practices are listed on the RCGP RSC webpage (http://www.rcgp.org.uk/rsc) and practices are informed via the monthly newsletter. The Health Research Authority (HRA) decision tool was used^29^, to confirm that NHS Research Ethics Committee (REC) approval for the study was not a requirement. Edinburgh Medical School Research Ethics Committee (EMREC) approval was sought regarding using the data (ref: 21:-EMREC-032).

### Reporting summary

Further information on research design is available in the Nature Research Reporting Summary linked to this article.

## Results

Snapshots of the dashboard are in Supplement 2—Fig. 1.

### Epidemiology

There were 7.6 million people registered in the study period (Table 1). Weekly incidence in the study practice vs RSC were 0.4% vs 0.4%, whereas annual prevalence was 6.1% vs 6.7% during the study week (Table 1). Annual prevalence of asthma in 0–4-years-olds was 0% in the study practice and 0.7% in RSC, whereas in 65-year-olds-and-above was 9.7% and 11.2%, respectively.

**Table 1:** Incidence in last 12 weeks and prevalence of asthma in last 12 months by age-groups for the study week (6–12/3/2023) in RSC.

	Study practice			RSC		
	People with asthma (*n*)	People registered (*N*)	% (95% CI)	People with asthma (*n*)	People registered (*N*)	% (95% CI)
Incidence in last 12 weeks	57	13,943	0.4 (0.3–0.5)	27,641	7,581,138	0.4 (0.4–0.4)
Prevalence in last 12 months						
0–4 years	0	555	0 (0–0)	2498	354,933	0.7 (0.7–0.7)
5–15 years	74	1515	4.9 (3.8–6.0)	46,875	982,072	4.8 (4.7–4.8)
16–64 years	582	10,424	5.6 (5.1–6.0)	336,626	4,855,362	6.9 (6.9–7.0)
>=65 years	163	1449	11.2 (9.6–12.9)	134,070	1,388,771	9.7 (9.6–9.7)
Age-standardised rate	819	13,943	6.1 (5.7–6.5)	520,069	7,581,138	6.7 (6.7–6.7)

### Modifiable factors

The inhaled reliever to preventer ratio for the study week in the study practice was 1.2 (95% CI 0.5–1.8) (SD 0.4) (*n*/*N* = 45/39) and in RSC was 1.1 (95% CI 0.4–1.8) (SD 0.4) (*n/N* = 40,085/35,397). Ratios of inhaled relievers to preventers were over one in the study practice and RSC in the study week. The other modifiable factors are in Table 2. Recording of the issuing of asthma self-management plans was higher in the study practice than in RSC (83.4% vs 60.8%), while it was the reverse for asthma annual reviews (36.8% vs 57.3%) (Table 2). In RSC, 39.2% people with asthma did not have asthma self-management plan and 42.7% did not have asthma review in the last 12 months. In the study practice, 16.6% people with asthma did not have an asthma self-management plan and 63.2% did not have an asthma review in the last 12 months. In RSC, 44.5% people with asthma did not have influenza vaccination compared to 43.4% in the study practice.

**Table 2:** Asthma modifiable factors for the study week (6-12/3/2023) in RSC.

	Study practice		RSC	
	*n/N*	% (95% CI)	*n/N*	% (95% CI)
Asthma self-management plan ever given	683/819	83.4 (80.8–85.9)	316,079/520,069	60.8 (60.6–60.9)
Asthma annual reviews in last 12 months	301/819	36.8 (33.5–40.1)	298,194/520,069	57.3 (57.2–57.5)
Influenza vaccination	535/945	56.6 (53.5–59.8)	332,650/599,834	55.5 (55.3–55.6)
2–3 years	0	0 (0–0)	754/1872	40.3 (38.1–42.5)
4–17 years	54/99	54.5 (44.7–64.4)	28,824/69,269	41.6 (41.2–42.0)
50–64 years	157/223	70.4 (64.4–76.4)	96,880/147,816	65.5 (65.3–65.8)
≥65 years	152/179	84.9 (79.7–90.2)	123,289/150,187	82.1 (81.9–82.3)
Others	172/444	38.7 (34.2–43.3)	82,903/230,690	35.9 (35.7–36.1)
Pneumococcal vaccination ever >=65 years	147/163	90.2 (85.6–94.8)	112,732/134,070	84.1 (83.9–84.3)
Active smokers	122/819	14.9 (12.5–17.3)	77,137/520,069	14.8 (14.7–14.9)

### Asthma exacerbations

There were a low number of asthma exacerbations in the study practice and these data therefore had to be suppressed for disclosure control reasons. In RSC, 0.1% of people with asthma had an asthma exacerbation and attended A&E in the study week (Table 3). Hospitalisations in RSC in the study week were 0.02% (95% CI: 0.02–0.03).

**Table 3:** Asthma exacerbation events in the study week (6–12/3/2023) in RSC.

	Study practice (*N* = 819)		RSC (*N* = 520,069)	
	*n*	% (95% CI)	*n*	% (95% CI)
Asthma exacerbations	^a^	^a^	519	0.1 (0.1–0.1)
Prednisolone prescriptions	16	2.0 (1.0–-2.9)	16,542	3.2 (3.1–3.2)
A&E attendances	^a^	^a^	626	0.1 (0.1–0.1)
Hospitalisations	^a^	^a^		0.02 (0.02–0.03)

^a^Counts <5 are withheld.

## Discussion

We demonstrated the mobilisation of 7.6 million people’s data to report asthma incidence, prevalence, care process /modifiable factors and outcomes in near-real time on a dashboard, which compared individual GP practices to the average of other practices in the RSC network. The dashboard data illustrates that inhaled relievers to preventers were over one in the study week and almost 40% in RSC did not have an asthma self-management plan from the dashboard inception and asthma review in the last 12 months. About 45% people with asthma did not have influenza vaccination. The dashboard provides a vehicle for A&F, and this paper reports the feasibility of doing this. The A&F topics would be around differences in incidence (Table 1 data), process of care measures (Table 2), and outcomes (Table 3). Thus, the dashboard can provide a helpful insight for busy GP practices of how the care and outcomes for their patients compare with reference standards and other practices in the network.

The asthma pyramid described here in England with a wide base of 520,069 people and a sharp peak of 0.02% hospitalisations is very similar to another cross-sectional study in Scotland^30^. Annual asthma prevalence in RSC of 6.7% is comparable to 6.5% in QOF in England for 2022–2023^31^. Seasonal flu uptake in RSC of 55.5% in people with asthma is also comparable to 55.0% uptake across clinical risk groups and eligible age-groups in England for 2022–2023^32^. There is huge room for improvement in our study population since the current advice is that people with asthma should have an annual review and asthma self-management plan^33–36^, and about 40% of RSC population with asthma did not have one. Since pay-for-performance incentives are not always effective, a dashboard updated frequently with potential for A&F as reported in this study, with perhaps the addition of goals and recommendations^35,36^, could be another way of motivating HCPs in primary care^11^. An interdisciplinary approach with HCPs, content and IT experts, to co-develop, review, modify and implement the dashboard was suggested^18,37^. Our dashboard prototype has A&F features which can provide objective data on current practice and can also compare other HCPs^9,10^, should HCPs feedback be useful to them. It was reported that implementation of asthma clinical practice guidelines in primary care could be improved by teamwork and assisting HCPs, especially nurses and pharmacists^7,38–40^. Furthermore it was found that asthma education and self-management programs when delivered by an integrated team of clinicians and allied HCPs are both clinically and cost effective^41^.

Also comparison of practice data with peers in a randomised control trial was found to be highly motivating^42^. Similar algorithms by the research team have been used for dashboards for atrial fibrillation^43^, influenza vaccine effectiveness^26^, and virological surveillance^44^, which have been peer-reviewed. The same research team also recently reported that their respective dashboards, which are economical and easy to scale-up, had been effective in increasing flu vaccination, medicine optimisation, improving diabetes care and impact on primary care quality^45^. Thus although this piece of work is not validated for asthma yet, we have evidence from similar exercise in RSC for other disease areas that such a RSC-dashboard has led to improvement.

To monitor and change HCPs behaviour, both to increase accountability and to improve quality of care, A&F is used widely in healthcare by a range of stakeholders, including research funders and health system payers, delivery organisations, professional groups and researchers. Three Cochrane systematic reviews between 2003 and 2012 on A&F interventions reported A&F led to small, but potentially important improvements in care and outcomes^46–48^. Later evidence reported that feedback was most effective when it was delivered by a supervisor or respected colleague, presented frequently, included both specific goals and action-plans in writing, aimed to change the targeted behaviour that needs addressed, focused on a problem where there was substantial scope for improvement and when the recipients were non-physicians^35,36^. The latest systematic review reported that even after 140 RCTs of A&F, it has remained difficult to identify how to optimise A&F, since of the 32 studies conducted after 2002, feedback was delivered only 19% of times by a supervisor or respected colleague and none of the studies included feedback with both explicit goals and action plans as recommended by previous systematic reviews^35,36,48^. The 2017 systematic review on electronic A&F, which is more common in recent times due to increased use of EHR, reported unreliable average effects, due to high heterogeneity and medium to high risk of bias in few studies (*n* = 7)^49^. Four statistically significant features were identified as independent predictors of improved clinical practice from 70 trials in a systematic review: automatic provision of decision support as part of clinician workflow, provision of recommendations rather than just assessments, provision of decision support at the time and location of decision making and computer based decision support^50^.

Timing of e-A&F is a big issue in its delivery^51^. e-A&F often provided by dashboards^49,52^, provide relevant and timely information via data visualisations of clinical performance summaries to healthcare professionals^17,53^. A review reported timely or near real-time e-A&F systems were imperative for proactive management of clinical risks, which resulted in increased participation and increased likelihood of reporting favourable outcomes^54^. Users found promptness of the feedback beneficial, insightful, made the data appear more reliable and performance-representative^54^. Less prompt feedback was frequently perceived as additional work needed and seemed to have taken place outside of the established workflow^54^. Absence of prompt feedback resulted in delayed effective action^54^. Furthermore, a barrier to usage of e-A&F systems was absence of real-time feedback^54^. Recent evidence suggests that e-A&F system implementation is effective within a highly stretched healthcare system when feedback is provided at near real-time, specific to user roles with an action plan embedded^55^. Near-real time feedback in dashboard was seen particularly effective during the COVID-19 pandemic to monitor the situation, assist in making clinical decisions and public health policies^56^. In contrast to near real-time notification, point-of-care notifications were more effective when data were about screening than lifestyle^57^. This was attributed to clinicians prioritising clinical information or which could be resolved comparatively quickly with less effort^57^. Alert fatigue from point-of-care notification was found in several studies, which resulted in distraction in workflow, clinician ignored content of message and which might have affected patient safety^58^. In summary, we conclude that both approaches have merits and limitations, and that these are best regarded as complementary approaches to driving forward quality improvement initiatives. The key strengths of point-of-care decision support capability is the potential for information availability to guide decision making during the process of care. The key limitations include presentation of what is perceived as irrelevant information by busy clinicians, presentation of information at a time that it interrupts workflows and high rates of over-riding of this information with associated risks. In contrast A&F offers the opportunity for assessing trends over time, benchmarking and assessing the impact of quality improvement initiatives. The main challenges with this are the presentation of data outside of the clinical EHR, which is an important barrier to access for busy clinicians and the non-contemporaneous nature of the data, which is something we have sought to overcome.

This study used a large primary care population database to create a comparative dashboard co-designed by multi-disciplinary team, on asthma at GP practice level and for all the other participating RSC practices. Thus, the findings on epidemiology, modifiable factors and outcomes can be generalised in the UK context. The feasibility of the dashboard has largely been technical, automating data flows to produce a dynamic contemporary data display across many practices. Additionally, we have had feedback on how we might develop the dashboard further. Generalisability on the A&F will come from a larger pilot where a larger group of practices are getting involved in A&F and we can look at engagement and change related to A&F. The A&F element would involve educational sessions with the intervention practices to explore how they might improve the processes of care (Table 2) and to provide a forum to discuss the outcomes of care (Table 3) with the goal of reducing adverse outcomes. Generally, the A&F cycle is a three-to-four-month cycle of review, then plan, practice activities, implement and then review again. Furthermore, these are possible to review by looking at the cumulative reporting in Tables 2 and 3 to get an indication of quality over the previous year as well as events in the last week.

The e-A&F is at practice-level. It is therefore not part of EHR. Rather, healthcare professionals have to log into asthma dashboard to view the e-A&F data. The dashboard though available using secure login, has not yet been promoted to HCPs due to lack of resources. Thus, we do not know if it is being used or what would be further useful to HCPs. Although the data existed to be able to categorise patients by mild, moderate or severe asthma, this was not attempted as our objective was to provide a snapshot at aggregated practice-level, as a technical feasibility exercise. The count of asthma self-management plan given and PPV in the dashboard are high since the look back period were long for both. People’s asthma symptoms vary over time and not all deteriorations lead to GP, out-of-hours or hospital visits. Such self-managed exacerbations of asthma will therefore not be recorded in GP records leading to a systematic under-estimate of the true prevalence of asthma exacerbations. Clinicians wanted data for previous week since they thought they might remember the details of an admission or exacerbation the previous week. However, the weakness of this approach is that it does not provide enough data for infrequent events such as asthma admissions. We recognise we may have to display exacerbations and admissions over a longer period.

A systematic review of e-A&F found that feedback displays were often graphical displays of individual practice performance and benchmarking, presented in dashboards^49^. While the feedback is not a written statement currently to economise on space in the dashboard, the graphs and tables have been kept simple for busy clinicians to quickly interpret. Thus, the smileys are just a quick indicator but not the only feedback. Dashboard algorithms are run in the background when new data are generated to do all calculations for tables, graphs and smileys. Whether tables, graphs and smileys were representing the data have been checked internally, before publishing on the web. The change in the smiley face is programmatic and highly reliable.

Since asthma is highly prevalent, the relatively low rates of asthma exacerbations still translate into a high number of A&E and hospital visits in a week. This may be considered a failure of asthma management and thus a bell-weather of the general management of asthma. This trend can be changed if we investigate the asthma modifiable factors and address them when required. Data on asthma modifiable factors are not routinely available, but insight of those data has the potential to alter asthma severity in people by timely intervention. The asthma dashboard with epidemiology, modifiable factors and severity measures could be an important CDSS tool in this respect.

Building on this foundational work, there is the potential to embark on a collaborative learning model involving a multi-disciplinary team of doctors, nurses, pharmacists, patient and public involvement members, behavioural health scientists, clinical informaticians, to better engage with people with asthma, aided by near-real time information as provided in the dashboard. Together we could develop methods to elicit what information HCPs would find most useful and how HCPs could be further motivated and based on their feedback we can improve the development and implementation of the asthma dashboard in primary care. Given the current system in place in RSC, there is an opportunity to implement A&F cycles in clinical practice and do a mixed method evaluation study of implementation effectiveness, by taking insights from HCPs on the ground interacting with the dashboard prototype and accordingly improve the dashboard iteratively, adopting a phased evaluation strategy^52^, with both explicit goals and action plans and find out what would aid scalability^18,34–36,39,40,48^. Multiple interventions were found to be more effective in asthma management than single interventions in primary care^38^. Given the recommendations from systematic reviews, we propose comparing GP practices to the high performing quintile for that parameter and set that as target for improvement, in near-real time.

This technical feasibility study found that an interactive, weekly, dashboard on asthma, with actionable insights for quality improvement, could be created with potential to support national A&F efforts, through a platform that is easily accessible online using primary care data. There are now opportunities to build on this foundational work through national experimental studies of A&F interventions to improve asthma care processes and outcomes.

### Supplementary information

Reporting Summary

Supplement

## Data Availability

Access to confidential Oxford-RCGP RSC data can be requested by submitting a written protocol and data request form to Primary Care Hosted Research Datasets Independent Scientific Committee (PrimDISC) (https://www.phc.ox.ac.uk/intranet/better-workplace-groups-committees-open-meetings/primdisc-committee). The data obtained from the TRE are presented in the Tables.
