# Supplementary material for: Deploying an asthma dashboard to support quality improvement across a nationally representative sentinel network of 7.6 million people in England
Source: NPJ Prim Care Respir Med. 2024 Jun 29;34:18. doi: 10.1038/s41533-024-00377-8 (PMC11217285; doi:10.1038/s41533-024-00377-8)
Supplement: Supplementary file 2 — Supplement [file 41533_2024_377_MOESM2_ESM.docx]

**Supplement**

**Codes for asthma and modifiable factors and screenshot of asthma dashboard in Oxford-RCGP RSC**

**Table of contents**

**Pages**

**Table S1: SNOMED CT codes for asthma 2-76**

**Table S2: SNOMED CT Expression Constraint Language (ECL) codes for asthma 77-80**

**Table S3: DM+D codes for prescriptions for asthma 80-128**

**Figure S1 Screenshots of asthma dashboard in RCGP RSC 129-130**

**Table S1: SNOMED CT codes for asthma**

| **Condition Name** | **Condition ID** | **Concept ID** | **Primary Term** |
| --- | --- | --- | --- |
| AsthmaExacerbation | 4204 | 30352005 | Allergic-infective asthma |
| AsthmaExacerbation | 4204 | 57546000 | Asthma with status asthmaticus |
| AsthmaExacerbation | 4204 | 59327009 | Intrinsic asthma with status asthmaticus |
| AsthmaExacerbation | 4204 | 91340006 | Extrinsic asthma with status asthmaticus |
| AsthmaExacerbation | 4204 | 233681001 | Extrinsic asthma with asthma attack |
| AsthmaExacerbation | 4204 | 233685005 | Intrinsic asthma with asthma attack |
| AsthmaExacerbation | 4204 | 233686006 | Aspirin-sensitive asthma with nasal polyps |
| AsthmaExacerbation | 4204 | 266363006 | Status asthmaticus NOS |
| AsthmaExacerbation | 4204 | 266364000 | Asthma attack |
| AsthmaExacerbation | 4204 | 274105009 | Asthma attack NOS |
| AsthmaExacerbation | 4204 | 281239006 | Acute asthma |
| AsthmaExacerbation | 4204 | 304527002 | Acute asthma |
| AsthmaExacerbation | 4204 | 407674008 | Aspirin-induced asthma |
| AsthmaExacerbation | 4204 | 425969006 | Exacerbation of intermittent asthma |
| AsthmaExacerbation | 4204 | 427354000 | Exacerbation of persistent asthma |
| AsthmaExacerbation | 4204 | 442025000 | Acute exacerbation of chronic asthmatic bronchitis |
| AsthmaExacerbation | 4204 | 707445000 | Exacerbation of mild persistent asthma |
| AsthmaExacerbation | 4204 | 707446004 | Exacerbation of moderate persistent asthma |
| AsthmaExacerbation | 4204 | 707447008 | Exacerbation of severe persistent asthma |
| AsthmaExacerbation | 4204 | 707979007 | Acute severe exacerbation of severe persistent asthma |
| AsthmaExacerbation | 4204 | 707980005 | Acute severe exacerbation of moderate persistent asthma |
| AsthmaExacerbation | 4204 | 707981009 | Acute severe exacerbation of mild persistent asthma |
| AsthmaExacerbation | 4204 | 708038006 | Acute exacerbation of asthma |
| AsthmaExacerbation | 4204 | 708090002 | Acute severe exacerbation of asthma |
| AsthmaExacerbation | 4204 | 708093000 | Acute exacerbation of allergic asthma |
| AsthmaExacerbation | 4204 | 708094006 | Acute exacerbation of intrinsic asthma |
| AsthmaExacerbation | 4204 | 708095007 | Acute severe exacerbation of immunoglobin E-mediated allergic asthma |
| AsthmaExacerbation | 4204 | 708096008 | Acute severe exacerbation of intrinsic asthma |
| AsthmaExacerbation | 4204 | 733858005 | Acute severe refractory exacerbation of asthma |
| AsthmaExacerbation | 4204 | 734904007 | Life threatening acute exacerbation of asthma |
| AsthmaExacerbation | 4204 | 734905008 | Moderate acute exacerbation of asthma |
| AsthmaExacerbation | 4204 | 735587000 | Acute severe exacerbation of asthma co-occurrent and due to allergic asthma |
| AsthmaExacerbation | 4204 | 762521001 | Exacerbation of allergic asthma |
| AsthmaExacerbation | 4204 | 782513000 | Acute severe exacerbation of allergic asthma |
| AsthmaExacerbation | 4204 | 782520007 | Exacerbation of allergic asthma due to infection |
| AsthmaExacerbation | 4204 | 786836003 | Near fatal asthma |
| AsthmaExacerbation | 4204 | 829976001 | Thunderstorm asthma |
| AsthmaExacerbation | 4204 | 1751000119100 | Acute exacerbation of chronic obstructive airways disease with asthma |
| AsthmaExacerbation | 4204 | 99031000119107 | Acute exacerbation of asthma co-occurrent with allergic rhinitis |
| AsthmaExacerbation | 4204 | 135171000119106 | Acute exacerbation of moderate persistent asthma |
| AsthmaExacerbation | 4204 | 135181000119109 | Acute exacerbation of mild persistent asthma |
| AsthmaExacerbation | 4204 | 653751000000109 | Asthma attack NOS |
| AsthmaExacerbation | 4204 | 689421000000104 | Status asthmaticus NOS |
| AsthmaExacerbation | 4204 | 1064771000000103 | Acute severe exacerbation of asthma |
| AsthmaExacerbation | 4204 | 1064811000000103 | Moderate acute exacerbation of asthma |
| AsthmaExacerbation | 4204 | 1064821000000109 | Life threatening acute exacerbation of asthma |
| AsthmaExacerbation | 4204 | 1086701000000102 | Life threatening acute exacerbation of allergic asthma |
| AsthmaExacerbation | 4204 | 1086711000000100 | Life threatening acute exacerbation of intrinsic asthma |
| AsthmaExacerbation | 4204 | 10674711000119105 | Acute severe exacerbation of asthma co-occurrent with allergic rhinitis |
| AsthmaExacerbation | 4204 | 10675471000119109 | Acute severe exacerbation of severe persistent allergic asthma |
| AsthmaExacerbation | 4204 | 10675551000119104 | Acute severe exacerbation of severe persistent asthma co-occurrent with allergic rhinitis |
| AsthmaExacerbation | 4204 | 10675911000119109 | Acute severe exacerbation of mild persistent allergic asthma |
| AsthmaExacerbation | 4204 | 10675991000119100 | Acute severe exacerbation of mild persistent allergic asthma co-occurrent with allergic rhinitis |
| AsthmaExacerbation | 4204 | 10676431000119103 | Acute severe exacerbation of moderate persistent allergic asthma |
| AsthmaExacerbation | 4204 | 10676511000119109 | Acute severe exacerbation of moderate persistent asthma co-occurrent with allergic rhinitis |
| AsthmaExacerbation | 4204 | 10692681000119108 | Aspirin exacerbated respiratory disease |
| AsthmaExacerbation | 4204 | 10692721000119102 | Chronic obstructive asthma co-occurrent with acute exacerbation of asthma |
| AsthmaManagementNoPlan | 4210 | 169591000000104 | Does not have asthma management plan |
| AsthmaManagementNoPlan | 4210 | 174171000000100 | Does not have asthma management plan |
| AsthmaManagementNoPlan | 4210 | 176711000000100 | Does not have asthma management plan |
| AsthmaManagementNoPlan | 4210 | 892301000000100 | Asthma management plan declined |
| AsthmaManagementNoPlan | 4210 | 916481000000109 | Asthma management plan declined |
| AsthmaManagementPlan | 4209 | 390872009 | Change in asthma management plan |
| AsthmaManagementPlan | 4209 | 390877003 | Step up change in asthma management plan |
| AsthmaManagementPlan | 4209 | 390878008 | Step down change in asthma management plan |
| AsthmaManagementPlan | 4209 | 412775002 | Asthma clinical management plan |
| AsthmaManagementPlan | 4209 | 736056000 | Asthma clinical management plan |
| AsthmaManagementPlan | 4209 | 116961000000106 | Asthma clinical management plan |
| AsthmaManagementPlan | 4209 | 121701000000107 | Asthma clinical management plan |
| AsthmaManagementPlan | 4209 | 811921000000103 | Asthma self-management plan agreed |
| AsthmaManagementPlan | 4209 | 811931000000101 | Asthma self-management plan agreed |
| AsthmaReview | 4208 | 393030008 | Asthma annual review |
| AsthmaReview | 4208 | 393082005 | Asthma medication review |
| AsthmaReview | 4208 | 393986005 | Asthma annual review |
| AsthmaReview | 4208 | 394033000 | Asthma medication review |
| AsthmaReview | 4208 | 394700004 | Asthma annual review |
| AsthmaReview | 4208 | 394720003 | Asthma medication review |
| AsthmaReview | 4208 | 754061000000100 | Asthma review using Royal College of Physicians three questions |
| AsthmaReview | 4208 | 754071000000107 | Asthma review (three Royal College of Pysicisan questions) |
| HospitalAdmission | 7163 | 417005 | Hospital re-admission |
| HospitalAdmission | 7163 | 1505002 | Hospital admission for isolation |
| HospitalAdmission | 7163 | 2252009 | Hospital admission, urgent, 48 hours |
| HospitalAdmission | 7163 | 2876009 | Hospital admission, type unclassified, explain by report |
| HospitalAdmission | 7163 | 4563007 | Hospital admission, transfer from other hospital or health care facility |
| HospitalAdmission | 7163 | 5161006 | Speciality clinic admission |
| HospitalAdmission | 7163 | 8715000 | Hospital admission, elective |
| HospitalAdmission | 7163 | 10378005 | Hospital admission, emergency, from emergency room, accidental injury |
| HospitalAdmission | 7163 | 11545006 | Emergency room admission, dead on arrival (DOA) |
| HospitalAdmission | 7163 | 15584006 | Hospital admission, elective, with partial pre-admission work-up |
| HospitalAdmission | 7163 | 18083007 | Hospital admission, emergency, indirect |
| HospitalAdmission | 7163 | 19951005 | Hospital admission, emergency, from emergency room, medical nature |
| HospitalAdmission | 7163 | 23473000 | Hospital admission, for research investigation |
| HospitalAdmission | 7163 | 25986004 | Hospital admission, under police custody |
| HospitalAdmission | 7163 | 32485007 | Hospital admission |
| HospitalAdmission | 7163 | 36723004 | Hospital admission, pre-nursing home placement |
| HospitalAdmission | 7163 | 40274000 | General outpatient clinic admission |
| HospitalAdmission | 7163 | 45702004 | Hospital admission, precertified by medical audit action |
| HospitalAdmission | 7163 | 47348005 | Hospital admission, mother, for observation, delivered outside of hospital |
| HospitalAdmission | 7163 | 48183000 | Hospital admission, special |
| HospitalAdmission | 7163 | 50331008 | Emergency room admission, followed by release |
| HospitalAdmission | 7163 | 50699000 | Hospital admission, short-term |
| HospitalAdmission | 7163 | 50849002 | Emergency room admission |
| HospitalAdmission | 7163 | 51032003 | Hospital admission, donor for transplant organ |
| HospitalAdmission | 7163 | 51501005 | Hospital admission, parent, for in-hospital child care |
| HospitalAdmission | 7163 | 52748007 | Hospital admission, involuntary |
| HospitalAdmission | 7163 | 55402005 | Hospital admission, for laboratory work-up, radiography, etc. |
| HospitalAdmission | 7163 | 60059000 | Hospital admission, infant, for observation, delivered outside of hospital |
| HospitalAdmission | 7163 | 63551005 | Hospital admission, from remote area, by means of special transportation |
| HospitalAdmission | 7163 | 65043002 | Hospital admission, short-term, day care |
| HospitalAdmission | 7163 | 70755000 | Hospital admission, by legal authority (commitment) |
| HospitalAdmission | 7163 | 71290004 | Hospital admission, limited to designated procedures |
| HospitalAdmission | 7163 | 73607007 | Hospital admission, emergency, from emergency room |
| HospitalAdmission | 7163 | 74857009 | Hospital admission, short-term, 24 hours |
| HospitalAdmission | 7163 | 75004002 | Emergency room admission, died in emergency room |
| HospitalAdmission | 7163 | 76464004 | Hospital admission, for observation |
| HospitalAdmission | 7163 | 78680009 | Hospital admission, emergency, direct |
| HospitalAdmission | 7163 | 81672003 | Hospital admission, elective, without pre-admission work-up |
| HospitalAdmission | 7163 | 82942009 | Hospital admission, blood donor |
| HospitalAdmission | 7163 | 112689000 | Hospital admission, elective, with complete pre-admission work-up |
| HospitalAdmission | 7163 | 112690009 | Hospital admission, boarder, for social reasons |
| HospitalAdmission | 7163 | 183430001 | Holiday relief admission |
| HospitalAdmission | 7163 | 183452005 | Emergency hospital admission |
| HospitalAdmission | 7163 | 183453000 | Admit medical emergency unspecified |
| HospitalAdmission | 7163 | 183454006 | Admit surgical emergency unspecified |
| HospitalAdmission | 7163 | 183455007 | Psychiatric emergency hospital admission |
| HospitalAdmission | 7163 | 183456008 | Emergency psychiatric admission MHA |
| HospitalAdmission | 7163 | 183457004 | Geriatric emergency hospital admission |
| HospitalAdmission | 7163 | 183458009 | Paediatric emergency hospital admission |
| HospitalAdmission | 7163 | 183459001 | Gynaecological emergency hospital admission |
| HospitalAdmission | 7163 | 183460006 | Obstetric emergency hospital admission |
| HospitalAdmission | 7163 | 183461005 | Orthopaedic emergency hospital admission |
| HospitalAdmission | 7163 | 183462003 | ENT emergency hospital admission |
| HospitalAdmission | 7163 | 183463008 | Trauma emergency hospital admission |
| HospitalAdmission | 7163 | 183464002 | Ophthalmological emergency hospital admission |
| HospitalAdmission | 7163 | 183465001 | Rheumatology emergency hospital admission |
| HospitalAdmission | 7163 | 183466000 | Dermatology emergency hospital admission |
| HospitalAdmission | 7163 | 183467009 | Neurology emergency hospital admission |
| HospitalAdmission | 7163 | 183468004 | Urology emergency hospital admission |
| HospitalAdmission | 7163 | 183469007 | Radiotherapy emergency hospital admission |
| HospitalAdmission | 7163 | 183470008 | Haematology emergency hospital admission |
| HospitalAdmission | 7163 | 183471007 | Plastic surgery emergency hospital admission |
| HospitalAdmission | 7163 | 183472000 | Diabetic emergency hospital admission |
| HospitalAdmission | 7163 | 183473005 | Oral surgical emergency hospital admission |
| HospitalAdmission | 7163 | 183474004 | Psychogeriatric emergency hospital admission |
| HospitalAdmission | 7163 | 183475003 | Renal medicine emergency hospital admission |
| HospitalAdmission | 7163 | 183476002 | Neurosurgical emergency hospital admission |
| HospitalAdmission | 7163 | 183477006 | Cardiothoracic emergency hospital admission |
| HospitalAdmission | 7163 | 183478001 | Emergency hospital admission for asthma |
| HospitalAdmission | 7163 | 183480007 | Admit hospital emergency NOS |
| HospitalAdmission | 7163 | 183481006 | Non-urgent hospital admission |
| HospitalAdmission | 7163 | 183482004 | Non-urgent hospital admission unspecified |
| HospitalAdmission | 7163 | 183497001 | Non-urgent trauma admission |
| HospitalAdmission | 7163 | 183514007 | Other hospital admission NOS |
| HospitalAdmission | 7163 | 299964000 | [V] Admission for bladder training |
| HospitalAdmission | 7163 | 302187006 | [V]Admission for instruction of self-catheterisation |
| HospitalAdmission | 7163 | 302987007 | [V]Acquired absence of organs |
| HospitalAdmission | 7163 | 304568006 | Admission for respite care |
| HospitalAdmission | 7163 | 305337004 | Admission to community hospital |
| HospitalAdmission | 7163 | 305338009 | Admission to GP hospital |
| HospitalAdmission | 7163 | 305339001 | Admission to private hospital |
| HospitalAdmission | 7163 | 305340004 | Admission to long stay hospital |
| HospitalAdmission | 7163 | 305341000 | Admission to tertiary referral hospital |
| HospitalAdmission | 7163 | 305342007 | Admission to ward |
| HospitalAdmission | 7163 | 305343002 | Admission to day ward |
| HospitalAdmission | 7163 | 305344008 | Admission to day hospital |
| HospitalAdmission | 7163 | 305345009 | Admission to psychiatric day hospital |
| HospitalAdmission | 7163 | 305346005 | Admission to psychogeriatric day hospital |
| HospitalAdmission | 7163 | 305347001 | Admission to elderly severely mentally ill day hospital |
| HospitalAdmission | 7163 | 305348006 | Admission to care of the elderly day hospital |
| HospitalAdmission | 7163 | 308162006 | [V]Admission for respite care |
| HospitalAdmission | 7163 | 308163001 | [V]Problems in relationship with parents and in-laws |
| HospitalAdmission | 7163 | 308164007 | [V]Problem was normal state |
| HospitalAdmission | 7163 | 309561003 | [V]Procreative/fertility counselling |
| HospitalAdmission | 7163 | 309788005 | [V]Admission for instruction in the use of a nebuliser |
| HospitalAdmission | 7163 | 313265008 | [V]Procedure cancelled due to non-availability of anaesthetist |
| HospitalAdmission | 7163 | 313266009 | [V]Procedure cancelled due to lack of theatre time |
| HospitalAdmission | 7163 | 313285007 | [V]Admitted for conversion to insulin |
| HospitalAdmission | 7163 | 313286008 | [V]Admitted for commencement of insulin |
| HospitalAdmission | 7163 | 313385005 | Cardiology emergency hospital admission |
| HospitalAdmission | 7163 | 315949001 | [V]Genetic counselling |
| HospitalAdmission | 7163 | 316361005 | [V]Housing, household and economic circumstances as reason for encounter |
| HospitalAdmission | 7163 | 316362003 | [V]Lack of housing |
| HospitalAdmission | 7163 | 316363008 | [V]Inadequate housing |
| HospitalAdmission | 7163 | 316364002 | [V]Inadequate material resources |
| HospitalAdmission | 7163 | 316365001 | [V]Person living alone |
| HospitalAdmission | 7163 | 316366000 | [V]No able carer in household |
| HospitalAdmission | 7163 | 316367009 | [V]Holiday relief care |
| HospitalAdmission | 7163 | 316368004 | [V]Institution resident |
| HospitalAdmission | 7163 | 316369007 | [V]Sheltered housing |
| HospitalAdmission | 7163 | 316370008 | [V]Carer unable to cope |
| HospitalAdmission | 7163 | 316372000 | [V]Other social reason for encounter |
| HospitalAdmission | 7163 | 316373005 | [V]Unspecified social reason for encounter |
| HospitalAdmission | 7163 | 316374004 | [V]Other family reason for encounter |
| HospitalAdmission | 7163 | 316375003 | [V]Family disruption |
| HospitalAdmission | 7163 | 316376002 | [V]Marital problems |
| HospitalAdmission | 7163 | 316377006 | [V]Child abuse |
| HospitalAdmission | 7163 | 316378001 | [V]Other parent-child problems |
| HospitalAdmission | 7163 | 316379009 | [V]Aged parents or in-law problems |
| HospitalAdmission | 7163 | 316380007 | [V]Health problems in family |
| HospitalAdmission | 7163 | 316381006 | [V]Multiparity |
| HospitalAdmission | 7163 | 316382004 | [V]Illegitimacy |
| HospitalAdmission | 7163 | 316383009 | [V]Illegitimate pregnancy |
| HospitalAdmission | 7163 | 316385002 | [V]Other unwanted pregnancy |
| HospitalAdmission | 7163 | 316387005 | [V]Other specified family reason for encounter |
| HospitalAdmission | 7163 | 316388000 | [V]Unspecified family reason for encounter |
| HospitalAdmission | 7163 | 316408003 | [V]Procedure planned but not carried out |
| HospitalAdmission | 7163 | 316409006 | [V]Vaccination not carried out because of contraindication |
| HospitalAdmission | 7163 | 316410001 | [V]Surgical or other procedure not carried out because of contraindication |
| HospitalAdmission | 7163 | 316411002 | [V]Surgical or other procedure not carried out because of patient decision |
| HospitalAdmission | 7163 | 316412009 | [V]No procedure - not indicated |
| HospitalAdmission | 7163 | 316413004 | [V]Operation not carried out as condition resolved |
| HospitalAdmission | 7163 | 316414005 | [V]Immunisation not carried out because of patient's decision for reasons of belief or group pressure |
| HospitalAdmission | 7163 | 316415006 | [V]Immunisation not carried out because of patient's decision for other unspecified reasons |
| HospitalAdmission | 7163 | 316416007 | [V]Immunisation not carried out for other reasons |
| HospitalAdmission | 7163 | 316417003 | [V]Immunisation not carried out for unspecified reason |
| HospitalAdmission | 7163 | 316420006 | [V]Procedure not carried out for other specified reason |
| HospitalAdmission | 7163 | 316421005 | [V]Procedure not carried out for unspecified reason |
| HospitalAdmission | 7163 | 316427009 | [V]Other counselling NEC |
| HospitalAdmission | 7163 | 316428004 | [V]Person with feared complaint, no diagnosis made |
| HospitalAdmission | 7163 | 316435007 | [V]Other specified encounter with person who has no complaint or sickness |
| HospitalAdmission | 7163 | 316436008 | [V]Unspecified encounter with person who has no complaint or sickness |
| HospitalAdmission | 7163 | 316487006 | [V]Person consulting for counselling or advice |
| HospitalAdmission | 7163 | 316488001 | [V]Counselling related to sexual attitude |
| HospitalAdmission | 7163 | 316489009 | [V]Counselling related to patient's sexual behaviour and orientation |
| HospitalAdmission | 7163 | 316490000 | [V]Counselling related to sexual behaviour and orientation of third party |
| HospitalAdmission | 7163 | 316491001 | [V]Counselling related to combined concerns regarding sexual attitude, behaviour and orientation |
| HospitalAdmission | 7163 | 316492008 | [V]Human immunodefiency virus counselling |
| HospitalAdmission | 7163 | 316493003 | [V]Person consulting for explanation of investigation findings |
| HospitalAdmission | 7163 | 316494009 | [V]Alcohol abuse counselling and surveillance |
| HospitalAdmission | 7163 | 316495005 | [V]Drug abuse counselling and surveillance |
| HospitalAdmission | 7163 | 316496006 | [V]Tobacco abuse counselling |
| HospitalAdmission | 7163 | 316497002 | [V]Other sex counselling |
| HospitalAdmission | 7163 | 316500008 | [V]Problems related to medical facilities and other health care |
| HospitalAdmission | 7163 | 316501007 | [V]Person awaiting admission to adequate facility elsewhere |
| HospitalAdmission | 7163 | 316502000 | [V]Unavailability and inaccessibility of other helping agencies |
| HospitalAdmission | 7163 | 316503005 | [V]Other problems related to medical facilities and other health care |
| HospitalAdmission | 7163 | 316504004 | [V]Unspecified problem related to medical facilities and other health care |
| HospitalAdmission | 7163 | 316515009 | [V]Acquired absence of organs NEC |
| HospitalAdmission | 7163 | 316516005 | [V]Acquired absence of part of head and neck |
| HospitalAdmission | 7163 | 316517001 | [V]Acquired absence of breast(s) |
| HospitalAdmission | 7163 | 316518006 | [V]Acquired absence of part of lung |
| HospitalAdmission | 7163 | 316519003 | [V]Acquired absence of part of stomach |
| HospitalAdmission | 7163 | 316520009 | [V]Acquired absence of other parts of digestive tract |
| HospitalAdmission | 7163 | 316521008 | [V]Acquired absence of kidney |
| HospitalAdmission | 7163 | 316522001 | [V]Acquired absence of other organs of urinary tract |
| HospitalAdmission | 7163 | 316523006 | [V]Acquired absence of genital organ(s) |
| HospitalAdmission | 7163 | 316524000 | [V]Acquired absence of other organs |
| HospitalAdmission | 7163 | 405614004 | Unexpected hospital admission |
| HospitalAdmission | 7163 | 408489005 | Respiratory emergency hospital admission |
| HospitalAdmission | 7163 | 408501008 | Emergency hospital admission for chronic obstructive pulmonary disease |
| HospitalAdmission | 7163 | 416683003 | Emergency hospital admission for heart failure |
| HospitalAdmission | 7163 | 699122005 | Vascular surgery emergency hospital admission |
| HospitalAdmission | 7163 | 699245006 | Emergency hospital admission for ischaemic heart disease |
| HospitalAdmission | 7163 | 3241000175106 | Hospital admission from non-health care facility |
| HospitalAdmission | 7163 | 188151000000107 | Admit heart failure emergency |
| HospitalAdmission | 7163 | 191651000000108 | Admit heart failure emergency |
| HospitalAdmission | 7163 | 313891000000105 | Emergency voluntary psychiatric admission under Mental Health Act |
| HospitalAdmission | 7163 | 313901000000106 | Emergency informal psychiatric admission |
| HospitalAdmission | 7163 | 313911000000108 | Emergency informal psychiatric admission |
| HospitalAdmission | 7163 | 320351000000105 | Admit ischaemic heart disease emergency |
| HospitalAdmission | 7163 | 320361000000108 | Emergency admission - ischaemic heart disease |
| HospitalAdmission | 7163 | 320371000000101 | Emergency admission - ischaemic heart disease |
| HospitalAdmission | 7163 | 369261000000102 | Admission to community hospital |
| HospitalAdmission | 7163 | 370181000000103 | Admission to community hospital |
| HospitalAdmission | 7163 | 395661000000100 | Emergency psychiatric admission under Mental Health Act 1983 (England and Wales) |
| HospitalAdmission | 7163 | 397941000000102 | [V]Other reasons for encounter |
| HospitalAdmission | 7163 | 398041000000100 | [V]Admitted for commencement of insulin |
| HospitalAdmission | 7163 | 398341000000102 | [V]Lack of housing |
| HospitalAdmission | 7163 | 399421000000108 | [V]Inadequate material resources |
| HospitalAdmission | 7163 | 400151000000105 | [V]Other counselling NEC |
| HospitalAdmission | 7163 | 400161000000108 | [V]Person with feared complaint, no diagnosis made |
| HospitalAdmission | 7163 | 400791000000103 | [V]Other parent-child problems |
| HospitalAdmission | 7163 | 401051000000100 | [V]Other social reason for encounter |
| HospitalAdmission | 7163 | 401171000000108 | [V] Admission for bladder training |
| HospitalAdmission | 7163 | 401651000000107 | [V]Procedure cancelled due to lack of theatre time |
| HospitalAdmission | 7163 | 402131000000102 | [V]Other unwanted pregnancy |
| HospitalAdmission | 7163 | 402291000000106 | [V]No able carer in household |
| HospitalAdmission | 7163 | 409891000000104 | [V]Other reasons for encounter OS |
| HospitalAdmission | 7163 | 410171000000104 | [V]Procedure cancelled due to non-availability of anaesthetist |
| HospitalAdmission | 7163 | 410371000000101 | [V]Housing, household and economic circumstances as reason for encounter |
| HospitalAdmission | 7163 | 410391000000102 | [V]Admission for respite care |
| HospitalAdmission | 7163 | 410801000000102 | [V]Illegitimate pregnancy |
| HospitalAdmission | 7163 | 411151000000102 | [V]Unspecified encounter with person who has no complaint or sickness |
| HospitalAdmission | 7163 | 411851000000109 | [X]Other boarder in health care facility |
| HospitalAdmission | 7163 | 412211000000100 | [V]Acquired absence of other parts of digestive tract |
| HospitalAdmission | 7163 | 413571000000101 | [V]Problems in relationship with parents and in-laws |
| HospitalAdmission | 7163 | 413761000000102 | [V]No procedure - not indicated |
| HospitalAdmission | 7163 | 414181000000105 | [V]Vaccination not carried out because of contraindication |
| HospitalAdmission | 7163 | 416411000000100 | [V]Procedure planned but not carried out |
| HospitalAdmission | 7163 | 416681000000109 | [V]Other specified reasons for encounter |
| HospitalAdmission | 7163 | 418621000000107 | [V]Admitted for commencement of insulin |
| HospitalAdmission | 7163 | 418871000000107 | [V]Institution resident |
| HospitalAdmission | 7163 | 418881000000109 | [V]Other sex counselling |
| HospitalAdmission | 7163 | 419371000000105 | [V]Person living alone |
| HospitalAdmission | 7163 | 420171000000106 | [V]Unspecified problem related to medical facilities and other health care |
| HospitalAdmission | 7163 | 426401000000108 | [V]Counselling related to patient's sexual behaviour and orientation |
| HospitalAdmission | 7163 | 427471000000102 | [V]Admission for instruction of self-catheterisation |
| HospitalAdmission | 7163 | 427601000000108 | [V]Procedure not carried out for unspecified reason |
| HospitalAdmission | 7163 | 427611000000105 | [V]Encounter with person who has no complaint or sickness |
| HospitalAdmission | 7163 | 427981000000109 | [V]Acquired absence of organs |
| HospitalAdmission | 7163 | 428231000000104 | [V]Other boarder in health-care facility |
| HospitalAdmission | 7163 | 428401000000109 | [V]Problems in relationship with parents and in-laws |
| HospitalAdmission | 7163 | 428511000000105 | [V]Illegitimacy |
| HospitalAdmission | 7163 | 429351000000109 | [V]Inadequate housing |
| HospitalAdmission | 7163 | 429841000000103 | [V]Acquired absence of other organs of urinary tract |
| HospitalAdmission | 7163 | 430251000000105 | [V]Unspecified reasons for encounter |
| HospitalAdmission | 7163 | 430531000000101 | [V]Child abuse |
| HospitalAdmission | 7163 | 430541000000105 | [V]Immunisation not carried out for unspecified reason |
| HospitalAdmission | 7163 | 430751000000103 | [V]Other family reason for encounter |
| HospitalAdmission | 7163 | 431101000000109 | [V]Admission for instruction in the use of a nebuliser |
| HospitalAdmission | 7163 | 431521000000106 | [V]Sheltered housing |
| HospitalAdmission | 7163 | 431921000000103 | [V]Unspecified family reason for encounter |
| HospitalAdmission | 7163 | 432621000124105 | Hospital admission from dialysis facility |
| HospitalAdmission | 7163 | 432661000000106 | [V]Marital problems |
| HospitalAdmission | 7163 | 440111000000104 | [V]Person consulting for counselling or advice |
| HospitalAdmission | 7163 | 440431000000103 | [V]Person with other specified health problems |
| HospitalAdmission | 7163 | 440731000000106 | [V]Health problems in family |
| HospitalAdmission | 7163 | 440741000000102 | [V]Healthy person accompanying sick person |
| HospitalAdmission | 7163 | 441901000000108 | [V]Dietary surveillance and counselling |
| HospitalAdmission | 7163 | 442701000000101 | [V]Unspecified social reason for encounter |
| HospitalAdmission | 7163 | 442711000000104 | [V]Immunisation not carried out because of patient's decision for reasons of belief or group pressure |
| HospitalAdmission | 7163 | 442721000000105 | [V]Problems related to medical facilities and other health care |
| HospitalAdmission | 7163 | 443421000000107 | [V]Carer unable to cope |
| HospitalAdmission | 7163 | 443861000000102 | [V]Holiday relief care |
| HospitalAdmission | 7163 | 443871000000109 | [V]Surgical or other procedure not carried out because of contraindication |
| HospitalAdmission | 7163 | 443901000000109 | [V]Drug abuse counselling and surveillance |
| HospitalAdmission | 7163 | 444741000000107 | [V]Family disruption |
| HospitalAdmission | 7163 | 451501000000101 | [V]Counselling related to sexual attitude |
| HospitalAdmission | 7163 | 451961000000105 | [V]Other reasons for encounter NOS |
| HospitalAdmission | 7163 | 451991000000104 | [V]Other specified encounter with person who has no complaint or sickness |
| HospitalAdmission | 7163 | 452011000000104 | [V]Acquired absence of other organs |
| HospitalAdmission | 7163 | 452111000000103 | [V]Problem was normal state |
| HospitalAdmission | 7163 | 452221000000100 | [V]Multiparity |
| HospitalAdmission | 7163 | 452441000000106 | [V]Procedure cancelled due to lack of theatre time |
| HospitalAdmission | 7163 | 452491000000101 | [V]Aged parents or in-law problems |
| HospitalAdmission | 7163 | 452561000000106 | [V]Genetic counselling |
| HospitalAdmission | 7163 | 453041000000106 | [V] Admission for bladder training |
| HospitalAdmission | 7163 | 454911000000106 | [V]Problem was normal state |
| HospitalAdmission | 7163 | 455051000000105 | [V]Admitted for conversion to insulin |
| HospitalAdmission | 7163 | 455711000000108 | [V]Acquired absence of organs NEC |
| HospitalAdmission | 7163 | 456641000000105 | [V]Other problems related to medical facilities and other health care |
| HospitalAdmission | 7163 | 463631000000103 | [V]Admitted for conversion to insulin |
| HospitalAdmission | 7163 | 464391000000106 | [V]Unspecified health problems |
| HospitalAdmission | 7163 | 464741000000102 | [V]Person consulting for explanation of investigation findings |
| HospitalAdmission | 7163 | 465741000000103 | [V]Admission for respite care |
| HospitalAdmission | 7163 | 465751000000100 | [V]Procedure cancelled due to non-availability of anaesthetist |
| HospitalAdmission | 7163 | 467311000000108 | [V]Acquired absence of part of head and neck |
| HospitalAdmission | 7163 | 469761000000100 | [V]Admission for instruction of self-catheterisation |
| HospitalAdmission | 7163 | 469991000000109 | [V]Procedure not carried out for other specified reason |
| HospitalAdmission | 7163 | 470201000000106 | [V]Operation not carried out as condition resolved |
| HospitalAdmission | 7163 | 470891000000100 | [V]Surgical or other procedure not carried out because of patient decision |
| HospitalAdmission | 7163 | 471181000000101 | [V]Other specified family reason for encounter |
| HospitalAdmission | 7163 | 472141000000100 | [V]Immunisation not carried out because of patient's decision for other unspecified reasons |
| HospitalAdmission | 7163 | 472151000000102 | [V]Immunisation not carried out for other reasons |
| HospitalAdmission | 7163 | 479091000000102 | [X]Persons encountering health services in other specified circumstances |
| HospitalAdmission | 7163 | 479851000000104 | [V]Counselling related to sexual behaviour and orientation of third party |
| HospitalAdmission | 7163 | 479861000000101 | [V]Admission for instruction in the use of a nebuliser |
| HospitalAdmission | 7163 | 480041000000107 | [V]Counselling related to combined concerns regarding sexual attitude, behaviour and orientation |
| HospitalAdmission | 7163 | 511661000000100 | Admit vascular surgery emergency |
| HospitalAdmission | 7163 | 511671000000107 | Admit vascular surgery emergency |
| HospitalAdmission | 7163 | 623231000000108 | Other hospital admission NOS |
| HospitalAdmission | 7163 | 629551000000101 | Admit surgical emergency unspecified |
| HospitalAdmission | 7163 | 646311000000102 | Admit hospital emergency NOS |
| HospitalAdmission | 7163 | 646321000000108 | Non-urgent hospital admission unspecified |
| HospitalAdmission | 7163 | 656151000000103 | Admit medical emergency unspecified |
| HospitalAdmission | 7163 | 780831000000103 | Admission to accident and emergency department |
| HospitalAdmission | 7163 | 814141000000105 | Hospital re-admission |
| HospitalAdmission | 7163 | 814151000000108 | Hospital re-admission |
| HospitalAdmission | 7163 | 831421000000102 | Admission to day ward |
| HospitalAdmission | 7163 | 842951000000109 | Emergency hospital admission from walk-in centre |
| HospitalAdmission | 7163 | 842961000000107 | Emergency admission from walk in centre |
| HospitalAdmission | 7163 | 848101000000108 | Admit anticoagulation emergency |
| HospitalAdmission | 7163 | 848111000000105 | Admit anticoagulation emergency |
| HospitalAdmission | 7163 | 851901000006112 | Elderly psychiatric emergency admission |
| HospitalAdmission | 7163 | 894881000000108 | Admission to observation ward |
| HospitalAdmission | 7163 | 894891000000105 | Admission to observation ward |
| HospitalAdmission | 7163 | 1077941000000106 | Emergency hospital admission to maxillofacial surgery service |
| HospitalAdmission | 7163 | 1077981000000103 | Emergency hospital admission to cardiac surgery service |
| HospitalAdmission | 7163 | 1077991000000101 | Emergency hospital admission to thoracic surgery service |
| HospitalAdmission | 7163 | 1078031000000104 | Emergency hospital admission to community paediatric service |
| HospitalAdmission | 7163 | 1078041000000108 | Emergency hospital admission to general surgical service |
| HospitalAdmission | 7163 | 1078051000000106 | Emergency hospital admission to transplantation surgery service |
| HospitalAdmission | 7163 | 1078061000000109 | Emergency hospital admission to breast surgery service |
| HospitalAdmission | 7163 | 1078071000000102 | Emergency hospital admission to colorectal surgery service |
| HospitalAdmission | 7163 | 1078081000000100 | Emergency hospital admission to cardiothoracic transplantation surgery service |
| HospitalAdmission | 7163 | 1078091000000103 | Emergency hospital admission to pain management service |
| HospitalAdmission | 7163 | 1078101000000106 | Emergency hospital admission to paediatric urology service |
| HospitalAdmission | 7163 | 1078111000000108 | Emergency hospital admission to paediatric gastrointestinal surgery service |
| HospitalAdmission | 7163 | 1078121000000102 | Emergency hospital admission to paediatric ear, nose and throat service |
| HospitalAdmission | 7163 | 1078131000000100 | Emergency hospital admission to paediatric ophthalmology service |
| HospitalAdmission | 7163 | 1078141000000109 | Emergency hospital admission to paediatric maxillofacial surgery service |
| HospitalAdmission | 7163 | 1078151000000107 | Emergency hospital admission to paediatric neurosurgery service |
| HospitalAdmission | 7163 | 1078161000000105 | Emergency hospital admission to paediatric plastic surgery service |
| HospitalAdmission | 7163 | 1078171000000103 | Emergency hospital admission to paediatric cardiac surgery service |
| HospitalAdmission | 7163 | 1078181000000101 | Emergency hospital admission to paediatric thoracic surgery service |
| HospitalAdmission | 7163 | 1078191000000104 | Emergency hospital admission to paediatric pain management service |
| HospitalAdmission | 7163 | 1078201000000102 | Emergency hospital admission to paediatric gastroenterology service |
| HospitalAdmission | 7163 | 1078211000000100 | Emergency hospital admission to paediatric endocrinology service |
| HospitalAdmission | 7163 | 1078221000000106 | Emergency hospital admission to paediatric dermatology service |
| HospitalAdmission | 7163 | 1078231000000108 | Emergency hospital admission to paediatric respiratory medicine service |
| HospitalAdmission | 7163 | 1078241000000104 | Emergency hospital admission to paediatric nephrology service |
| HospitalAdmission | 7163 | 1078251000000101 | Emergency hospital admission to paediatric rheumatology service |
| HospitalAdmission | 7163 | 1078261000000103 | Emergency hospital admission to gastroenterology service |
| HospitalAdmission | 7163 | 1078271000000105 | Emergency hospital admission to endocrinology service |
| HospitalAdmission | 7163 | 1078281000000107 | Emergency hospital admission to critical care medicine service |
| HospitalAdmission | 7163 | 1078291000000109 | Emergency hospital admission to general medical service |
| HospitalAdmission | 7163 | 1078301000000108 | Emergency hospital admission to hepatology service |
| HospitalAdmission | 7163 | 1078311000000105 | Emergency hospital admission to palliative medicine service |
| HospitalAdmission | 7163 | 1078321000000104 | Emergency hospital admission to respite care service |
| HospitalAdmission | 7163 | 1078331000000102 | Emergency hospital admission to intermediate care service |
| HospitalAdmission | 7163 | 1078341000000106 | Emergency hospital admission to paediatric cardiology service |
| HospitalAdmission | 7163 | 1078351000000109 | Emergency hospital admission to cardiac rehabilitation service |
| HospitalAdmission | 7163 | 1078531000000105 | Emergency hospital admission to paediatric dentistry service |
| HospitalAdmission | 7163 | 1078541000000101 | Emergency hospital admission to orthodontics service |
| HospitalAdmission | 7163 | 1078551000000103 | Emergency hospital admission to paediatric surgical service |
| HospitalAdmission | 7163 | 1078561000000100 | Emergency hospital admission to paediatric intensive care service |
| HospitalAdmission | 7163 | 1078571000000107 | Emergency hospital admission to trauma and orthopaedics service |
| HospitalAdmission | 7163 | 1078581000000109 | Emergency hospital admission to paediatric transplantation surgery service |
| HospitalAdmission | 7163 | 1078591000000106 | Emergency hospital admission to paediatric trauma and orthopaedics surgery service |
| HospitalAdmission | 7163 | 1078601000000100 | Emergency hospital admission to paediatric medical oncology service |
| HospitalAdmission | 7163 | 1078611000000103 | Emergency hospital admission to paediatric diabetes service |
| HospitalAdmission | 7163 | 1078621000000109 | Emergency hospital admission to paediatric interventional radiology service |
| HospitalAdmission | 7163 | 1078631000000106 | Emergency hospital admission to paediatric neurodisability service |
| HospitalAdmission | 7163 | 1078641000000102 | Emergency hospital admission to clinical pharmacology service |
| HospitalAdmission | 7163 | 1078651000000104 | Emergency hospital admission to haemophilia service |
| HospitalAdmission | 7163 | 1078661000000101 | Emergency hospital admission to clinical immunology and allergy service |
| HospitalAdmission | 7163 | 1078671000000108 | Emergency hospital admission to stroke service |
| HospitalAdmission | 7163 | 1078681000000105 | Emergency hospital admission to genitourinary medicine service |
| HospitalAdmission | 7163 | 1078691000000107 | Emergency hospital admission to clinical neurophysiology service |
| HospitalAdmission | 7163 | 1078701000000107 | Emergency hospital admission to podiatric surgery service |
| HospitalAdmission | 7163 | 1078711000000109 | Emergency hospital admission to midwifery service |
| HospitalAdmission | 7163 | 1078721000000103 | Emergency hospital admission to forensic psychiatry service |
| HospitalAdmission | 7163 | 1078731000000101 | Emergency hospital admission to eating disorders service |
| HospitalAdmission | 7163 | 1078741000000105 | Emergency hospital admission to clinical oncology service |
| HospitalAdmission | 7163 | 1078751000000108 | Emergency hospital admission to interventional radiology service |
| HospitalAdmission | 7163 | 1078761000000106 | Emergency hospital admission to upper gastrointestinal surgery service |
| HospitalAdmission | 7163 | 1078771000000104 | Emergency hospital admission to restorative dentistry service |
| HospitalAdmission | 7163 | 1078781000000102 | Emergency hospital admission to tropical medicine service |
| HospitalAdmission | 7163 | 1078791000000100 | Emergency hospital admission to medical oncology service |
| HospitalAdmission | 7163 | 1078801000000101 | Emergency hospital admission to nuclear medicine service |
| HospitalAdmission | 7163 | 1078811000000104 | Emergency hospital admission to psychiatric intensive care service |
| HospitalAdmission | 7163 | 1078821000000105 | Emergency hospital admission to child and adolescent psychiatry service |
| HospitalAdmission | 7163 | 1078831000000107 | Emergency hospital admission to gynaecological oncology service |
| HospitalAdmission | 7163 | 1078841000000103 | Emergency hospital admission to medical ophthalmology service |
| HospitalAdmission | 7163 | 1078851000000100 | Emergency hospital admission to paediatric burn care service |
| HospitalAdmission | 7163 | 1078861000000102 | Emergency hospital admission to clinical immunology service |
| HospitalAdmission | 7163 | 1078871000000109 | Emergency hospital admission to allergy service |
| HospitalAdmission | 7163 | 1078881000000106 | Emergency hospital admission to spinal injuries service |
| HospitalAdmission | 7163 | 1078891000000108 | Emergency hospital admission to respiratory physiology service |
| HospitalAdmission | 7163 | 1079141000000103 | Emergency hospital admission to addiction service |
| HospitalAdmission | 7163 | 1079151000000100 | Emergency hospital admission to burn care service |
| HospitalAdmission | 7163 | 1079161000000102 | Emergency hospital admission to paediatric clinical haematology service |
| HospitalAdmission | 7163 | 1079171000000109 | Emergency hospital admission to paediatric infectious diseases service |
| HospitalAdmission | 7163 | 1079181000000106 | Emergency hospital admission to paediatric metabolic disease service |
| HospitalAdmission | 7163 | 1079191000000108 | Emergency hospital admission to paediatric cystic fibrosis service |
| HospitalAdmission | 7163 | 1079201000000105 | Emergency hospital admission to transient ischaemic attack service |
| HospitalAdmission | 7163 | 1079211000000107 | Emergency hospital admission to adult cystic fibrosis service |
| HospitalAdmission | 7163 | 1079221000000101 | Emergency hospital admission to infectious diseases service |
| HospitalAdmission | 7163 | 1079231000000104 | Emergency hospital admission to paediatric neurology service |
| HospitalAdmission | 7163 | 1079241000000108 | Emergency hospital admission to neonatal critical care service |
| HospitalAdmission | 7163 | 1079251000000106 | Emergency hospital admission to psychotherapy service |
| HospitalAdmission | 7163 | 1079261000000109 | Emergency hospital admission to perinatal psychiatry service |
| HospitalAdmission | 7163 | 1079271000000102 | Emergency hospital admission to hepatobiliary and pancreatic surgery service |
| HospitalAdmission | 7163 | 1079281000000100 | Emergency hospital admission to spinal surgery service |
| HospitalAdmission | 7163 | 1079291000000103 | Emergency hospital admission to paediatric epilepsy service |
| HospitalAdmission | 7163 | 1079301000000104 | Emergency hospital admission to paediatric audiological medicine service |
| HospitalAdmission | 7163 | 1079311000000102 | Emergency hospital admission to paediatric clinical immunology and allergy service |
| HospitalAdmission | 7163 | 1079321000000108 | Emergency hospital admission to blood and marrow transplantation service |
| HospitalAdmission | 7163 | 1079331000000105 | Emergency hospital admission to audiological medicine service |
| HospitalAdmission | 7163 | 1079341000000101 | Emergency hospital admission to clinical genetics service |
| HospitalAdmission | 7163 | 1079351000000103 | Emergency hospital admission to rehabilitation service |
| HospitalAdmission | 7163 | 1079361000000100 | Emergency hospital admission to sport and exercise medicine service |
| HospitalAdmission | 7163 | 1079371000000107 | Emergency hospital admission to congenital heart disease service |
| HospitalAdmission | 7163 | 1082351000000104 | Emergency hospital admission to adult mental health service |
| HospitalAdmission | 7163 | 1082361000000101 | Emergency hospital admission to complex specialised rehabilitation service |
| HospitalAdmission | 7163 | 1082371000000108 | Emergency hospital admission to dental medicine specialties service |
| HospitalAdmission | 7163 | 1082381000000105 | Emergency hospital admission to learning disability service |
| HospitalAdmission | 7163 | 1082391000000107 | Emergency hospital admission to local specialist rehabilitation service |
| HospitalAdmission | 7163 | 1082401000000105 | Emergency hospital admission to programmed pulmonary rehabilitation service |
| HospitalAdmission | 7163 | 1082411000000107 | Emergency hospital admission to well babies specialty |
| HospitalAdmission | 7163 | 1082421000000101 | Emergency hospital admission to accident and emergency service |
| HospitalAdmission | 7163 | 1823531000006111 | Hospital admission, emergency, from walk-in centre |
| HospitalAdmission | 7163 | 1823541000006118 | Admission by own GP |
| HospitalAdmission | 7163 | 1823551000006116 | Admission by GP partner |
| HospitalAdmission | 7163 | 1880401000006119 | Admission to observation ward |
| Influenza-likeIllness-WRpt | 1020 | 6142004 | Influenza |
| Influenza-likeIllness-WRpt | 1020 | 24662006 | Influenza due to Influenza B virus |
| Influenza-likeIllness-WRpt | 1020 | 41269000 | Influenzal bronchopneumonia |
| Influenza-likeIllness-WRpt | 1020 | 42964004 | Influenza with pneumonia |
| Influenza-likeIllness-WRpt | 1020 | 43692000 | Influenzal acute upper respiratory infection |
| Influenza-likeIllness-WRpt | 1020 | 46171006 | Influenza due to Influenza virus, type A, porcine |
| Influenza-likeIllness-WRpt | 1020 | 55604004 | Avian influenza |
| Influenza-likeIllness-WRpt | 1020 | 61700007 | Influenza with non-respiratory manifestation |
| Influenza-likeIllness-WRpt | 1020 | 63039003 | Influenza with respiratory manifestation other than pneumonia |
| Influenza-likeIllness-WRpt | 1020 | 74644004 | Influenza with encephalopathy |
| Influenza-likeIllness-WRpt | 1020 | 78046005 | Myocarditis due to influenza virus |
| Influenza-likeIllness-WRpt | 1020 | 78431007 | Influenza due to Influenza virus, type A, human |
| Influenza-likeIllness-WRpt | 1020 | 81524006 | Influenza due to Influenza C virus |
| Influenza-likeIllness-WRpt | 1020 | 84037004 | Swine influenza |
| Influenza-likeIllness-WRpt | 1020 | 95891005 | Influenza-like illness |
| Influenza-likeIllness-WRpt | 1020 | 139168000 | Influenza-like symptoms |
| Influenza-likeIllness-WRpt | 1020 | 161913008 | Influenza-like symptoms |
| Influenza-likeIllness-WRpt | 1020 | 194946005 | Acute myocarditis - influenzal |
| Influenza-likeIllness-WRpt | 1020 | 195878008 | Pneumonia and influenza |
| Influenza-likeIllness-WRpt | 1020 | 195920000 | Influenza with pneumonia, influenza virus identified |
| Influenza-likeIllness-WRpt | 1020 | 195921001 | Influenza with pneumonia NOS |
| Influenza-likeIllness-WRpt | 1020 | 195922008 | Influenza with other respiratory manifestation |
| Influenza-likeIllness-WRpt | 1020 | 195923003 | Influenza with laryngitis |
| Influenza-likeIllness-WRpt | 1020 | 195924009 | Influenza with pharyngitis |
| Influenza-likeIllness-WRpt | 1020 | 195925005 | Influenza with respiratory manifestations NOS |
| Influenza-likeIllness-WRpt | 1020 | 195927002 | Influenza with other manifestations |
| Influenza-likeIllness-WRpt | 1020 | 195929004 | Influenza with gastrointestinal tract involvement |
| Influenza-likeIllness-WRpt | 1020 | 195930009 | Influenza with other manifestations NOS |
| Influenza-likeIllness-WRpt | 1020 | 195933006 | Other specified pneumonia or influenza |
| Influenza-likeIllness-WRpt | 1020 | 195934000 | Pneumonia or influenza NOS |
| Influenza-likeIllness-WRpt | 1020 | 196200002 | [X]Influenza with other respiratory manifestations, influenza virus identified |
| Influenza-likeIllness-WRpt | 1020 | 196201003 | [X]Influenza with other manifestations, influenza virus identified |
| Influenza-likeIllness-WRpt | 1020 | 196202005 | [X]Influenza with other respiratory manifestations, virus not identified |
| Influenza-likeIllness-WRpt | 1020 | 196203000 | [X]Influenza with other manifestations, virus not identified |
| Influenza-likeIllness-WRpt | 1020 | 266353003 | Influenza NOS |
| Influenza-likeIllness-WRpt | 1020 | 274104008 | Flu-like illness NOS |
| Influenza-likeIllness-WRpt | 1020 | 309789002 | Encephalitis due to influenza |
| Influenza-likeIllness-WRpt | 1020 | 309806000 | Encephalitis due to influenza-virus identified |
| Influenza-likeIllness-WRpt | 1020 | 313251006 | Encephalitis due to influenza-specific virus not identified |
| Influenza-likeIllness-WRpt | 1020 | 315642008 | Influenza-like symptoms |
| Influenza-likeIllness-WRpt | 1020 | 408687004 | Healthcare associated influenza disease |
| Influenza-likeIllness-WRpt | 1020 | 427873006 | Influenza due to influenza virus type A, avian, H5N1 strain |
| Influenza-likeIllness-WRpt | 1020 | 442438000 | Influenza due to Influenza A virus |
| Influenza-likeIllness-WRpt | 1020 | 442696006 | Influenza due to Influenza A virus subtype H1N1 |
| Influenza-likeIllness-WRpt | 1020 | 450715004 | Influenza due to Influenza A virus subtype H7 |
| Influenza-likeIllness-WRpt | 1020 | 450716003 | Influenza due to Influenza A virus subtype H9 |
| Influenza-likeIllness-WRpt | 1020 | 707448003 | Influenza due to Influenza A virus subtype H7N9 |
| Influenza-likeIllness-WRpt | 1020 | 711128004 | Influenza due to influenza virus type A, avian, H3N2 strain |
| Influenza-likeIllness-WRpt | 1020 | 713083002 | Influenza caused by Influenza A virus subtype H5 |
| Influenza-likeIllness-WRpt | 1020 | 719590007 | Influenza caused by seasonal influenza virus |
| Influenza-likeIllness-WRpt | 1020 | 719865001 | Influenza caused by pandemic influenza virus |
| Influenza-likeIllness-WRpt | 1020 | 772810003 | Influenza caused by Influenza A virus subtype H3N2 |
| Influenza-likeIllness-WRpt | 1020 | 772828001 | Influenza caused by Influenza A virus subtype H5N1 |
| Influenza-likeIllness-WRpt | 1020 | 772839003 | Pneumonia caused by Influenza A virus |
| Influenza-likeIllness-WRpt | 1020 | 1149091008 | Influenza caused by Influenza A virus subtype H2 |
| Influenza-likeIllness-WRpt | 1020 | 16311000119108 | Pneumonia due to influenza |
| Influenza-likeIllness-WRpt | 1020 | 142921000119103 | Upper respiratory tract infection due to avian influenza |
| Influenza-likeIllness-WRpt | 1020 | 142931000119100 | Pneumonia due to H1N1 influenza |
| Influenza-likeIllness-WRpt | 1020 | 142941000119109 | Upper respiratory tract infection due to H1N1 influenza |
| Influenza-likeIllness-WRpt | 1020 | 142951000119106 | Myocarditis due to Influenza A virus subtype H1N1 |
| Influenza-likeIllness-WRpt | 1020 | 142961000119108 | Gastroenteritis due to H1N1 influenza |
| Influenza-likeIllness-WRpt | 1020 | 142981000119104 | Myocarditis due to avian influenza |
| Influenza-likeIllness-WRpt | 1020 | 142991000119101 | Gastroenteritis due to avian influenza |
| Influenza-likeIllness-WRpt | 1020 | 143111000119103 | Pneumonia due to avian influenza |
| Influenza-likeIllness-WRpt | 1020 | 280331000000102 | Avian influenza |
| Influenza-likeIllness-WRpt | 1020 | 292631000000106 | Avian influenza |
| Influenza-likeIllness-WRpt | 1020 | 328531000119104 | Upper respiratory tract infection due to Influenza A |
| Influenza-likeIllness-WRpt | 1020 | 418181000000104 | [X]Influenza with other respiratory manifestations, influenza virus identified |
| Influenza-likeIllness-WRpt | 1020 | 418191000000102 | [X]Influenza with other manifestations, influenza virus identified |
| Influenza-likeIllness-WRpt | 1020 | 430891000000103 | [X]Influenza with other respiratory manifestations, virus not identified |
| Influenza-likeIllness-WRpt | 1020 | 441131000000104 | [X]Influenza with other manifestations, virus not identified |
| Influenza-likeIllness-WRpt | 1020 | 505131000000104 | Influenza due to Influenza A virus subtype H1N1 |
| Influenza-likeIllness-WRpt | 1020 | 510671000000104 | Influenza due to Influenza A virus subtype H1N1 |
| Influenza-likeIllness-WRpt | 1020 | 540121000000103 | Influenza with other manifestations |
| Influenza-likeIllness-WRpt | 1020 | 540131000000101 | Influenza with other manifestations NOS |
| Influenza-likeIllness-WRpt | 1020 | 540141000000105 | Other specified pneumonia or influenza |
| Influenza-likeIllness-WRpt | 1020 | 540151000000108 | Pneumonia or influenza NOS |
| Influenza-likeIllness-WRpt | 1020 | 616161000000107 | Influenza with pneumonia NOS |
| Influenza-likeIllness-WRpt | 1020 | 616171000000100 | Influenza with other respiratory manifestation |
| Influenza-likeIllness-WRpt | 1020 | 616181000000103 | Influenza with respiratory manifestations NOS |
| Influenza-likeIllness-WRpt | 1020 | 670551000000108 | Influenza NOS |
| Influenza-likeIllness-WRpt | 1020 | 677811000000106 | Flu-like illness NOS |
| Influenza-likeIllness-WRpt | 1020 | 856211000006111 | Post influenzal debility |
| Influenza-likeIllness-WRpt | 1020 | 1033051000000101 | Influenza due to zoonotic influenza virus |
| Influenza-likeIllness-WRpt | 1020 | 1033061000000103 | Influenza due to zoonotic influenza virus |
| Influenza-likeIllness-WRpt | 1020 | 1033071000000105 | Influenza due to pandemic influenza virus |
| Influenza-likeIllness-WRpt | 1020 | 1033081000000107 | Influenza due to pandemic influenza virus |
| Influenza-likeIllness-WRpt | 1020 | 1033091000000109 | Influenza due to seasonal influenza virus |
| Influenza-likeIllness-WRpt | 1020 | 1033101000000101 | Influenza due to seasonal influenza virus |
| Influenza-likeIllness-WRpt | 1020 | 1033111000000104 | Influenza with pneumonia due to seasonal influenza virus |
| Influenza-likeIllness-WRpt | 1020 | 1033121000000105 | Influenzal bronchopneumonia due to seasonal influenza virus |
| Influenza-likeIllness-WRpt | 1020 | 1050601000000101 | Influenza due to seasonal influenza virus |
| Influenza-likeIllness-WRpt | 1020 | 1050981000000100 | Influenza due to seasonal influenza virus |
| Influenza-likeIllness-WRpt | 1020 | 1787121000006116 | Community acquired pneumonia |
| Influenza-likeIllness-WRpt | 1020 | 1787131000006118 | Hospital acquired pneumonia |
| Influenza-likeIllness-WRpt | 1020 | 10628871000119101 | Gastroenteritis due to influenza |
| Influenza-likeIllness-WRpt | 1020 | 10628911000119103 | Gastroenteritis due to Influenza A virus |
| Influenza-likeIllness-WRpt | 1020 | 10629191000119100 | Bronchiolitis caused by influenza virus |
| Influenza-likeIllness-WRpt | 1020 | 10629351000119108 | Myocarditis due to Influenza A virus |
| Influenza-likeIllness-WRpt | 1020 | 10685111000119102 | Upper respiratory tract infection due to Influenza |
| LowerRespiratoryTractInfection-WRpt | 1055 | 181007 | Haemorrhagic bronchopneumonia |
| LowerRespiratoryTractInfection-WRpt | 1055 | 718004 | Acute bronchiolitis with obstruction |
| LowerRespiratoryTractInfection-WRpt | 1055 | 2087000 | Pulmonary nocardiosis |
| LowerRespiratoryTractInfection-WRpt | 1055 | 2523007 | Salmonella pneumonia |
| LowerRespiratoryTractInfection-WRpt | 1055 | 2585002 | Pneumococcal pleurisy |
| LowerRespiratoryTractInfection-WRpt | 1055 | 3144005 | Staphylococcal pleurisy |
| LowerRespiratoryTractInfection-WRpt | 1055 | 3214003 | Invasive pulmonary aspergillosis |
| LowerRespiratoryTractInfection-WRpt | 1055 | 3487004 | Candidiasis of lung |
| LowerRespiratoryTractInfection-WRpt | 1055 | 4120002 | Bronchiolitis |
| LowerRespiratoryTractInfection-WRpt | 1055 | 5505005 | Acute bronchiolitis |
| LowerRespiratoryTractInfection-WRpt | 1055 | 5875001 | Acute bronchitis with obstruction |
| LowerRespiratoryTractInfection-WRpt | 1055 | 6042001 | Pulmonary aspergillosis |
| LowerRespiratoryTractInfection-WRpt | 1055 | 7063008 | Gangrenous pneumonia |
| LowerRespiratoryTractInfection-WRpt | 1055 | 7097001 | Streptococcal pleurisy with effusion |
| LowerRespiratoryTractInfection-WRpt | 1055 | 7238003 | Jaagziekte |
| LowerRespiratoryTractInfection-WRpt | 1055 | 7548000 | Rheumatic pneumonia |
| LowerRespiratoryTractInfection-WRpt | 1055 | 7678002 | Cytomegaloviral pneumonia |
| LowerRespiratoryTractInfection-WRpt | 1055 | 8555001 | Syphilis of lung |
| LowerRespiratoryTractInfection-WRpt | 1055 | 9095002 | Adiaspiromycosis due to Emmonsia crescens |
| LowerRespiratoryTractInfection-WRpt | 1055 | 9228003 | Pulmonary schistosomiasis |
| LowerRespiratoryTractInfection-WRpt | 1055 | 9505000 | Infestation by Pneumonyssus simicola |
| LowerRespiratoryTractInfection-WRpt | 1055 | 10446001 | Aspergilloma |
| LowerRespiratoryTractInfection-WRpt | 1055 | 10509002 | Acute bronchitis |
| LowerRespiratoryTractInfection-WRpt | 1055 | 11389007 | Inhalational anthrax |
| LowerRespiratoryTractInfection-WRpt | 1055 | 11885007 | Corynebacterial pneumonia of foals |
| LowerRespiratoryTractInfection-WRpt | 1055 | 13089009 | Adenoviral bronchiolitis |
| LowerRespiratoryTractInfection-WRpt | 1055 | 14527007 | Tuberculous empyema |
| LowerRespiratoryTractInfection-WRpt | 1055 | 15199004 | Acute bronchiolitis with bronchospasm |
| LowerRespiratoryTractInfection-WRpt | 1055 | 15341009 | Atypical interstitial pneumonia of cattle |
| LowerRespiratoryTractInfection-WRpt | 1055 | 16146001 | Viral bronchitis |
| LowerRespiratoryTractInfection-WRpt | 1055 | 16810008 | AIDS with viral pneumonia |
| LowerRespiratoryTractInfection-WRpt | 1055 | 18988001 | Septic pleurisy |
| LowerRespiratoryTractInfection-WRpt | 1055 | 19287005 | North American pulmonary blastomycosis |
| LowerRespiratoryTractInfection-WRpt | 1055 | 20953001 | Pulmonary cryptococcosis |
| LowerRespiratoryTractInfection-WRpt | 1055 | 21846001 | Pulmonary actinomycosis |
| LowerRespiratoryTractInfection-WRpt | 1055 | 22754005 | Staphylococcal pneumonia |
| LowerRespiratoryTractInfection-WRpt | 1055 | 23698009 | Staphylococcal pleurisy with effusion |
| LowerRespiratoryTractInfection-WRpt | 1055 | 24235005 | Tuberculous hydrothorax |
| LowerRespiratoryTractInfection-WRpt | 1055 | 24302002 | Pneumococcal pleurisy with effusion |
| LowerRespiratoryTractInfection-WRpt | 1055 | 25042006 | Tracheostomy sepsis |
| LowerRespiratoryTractInfection-WRpt | 1055 | 26427008 | Chronic pulmonary histoplasmosis |
| LowerRespiratoryTractInfection-WRpt | 1055 | 27475006 | Parainfluenza virus bronchitis |
| LowerRespiratoryTractInfection-WRpt | 1055 | 27757009 | Encysted pleurisy |
| LowerRespiratoryTractInfection-WRpt | 1055 | 27836007 | Pertussis |
| LowerRespiratoryTractInfection-WRpt | 1055 | 28085001 | Empyema with bronchopleural fistula |
| LowerRespiratoryTractInfection-WRpt | 1055 | 29591002 | Purulent bronchitis |
| LowerRespiratoryTractInfection-WRpt | 1055 | 29731002 | Tuberculous pneumothorax |
| LowerRespiratoryTractInfection-WRpt | 1055 | 30437004 | Empyema with bronchocutaneous fistula |
| LowerRespiratoryTractInfection-WRpt | 1055 | 31561003 | Hypostatic bronchopneumonia |
| LowerRespiratoryTractInfection-WRpt | 1055 | 31920006 | Haemorrhagic varicella pneumonitis |
| LowerRespiratoryTractInfection-WRpt | 1055 | 32204007 | Pulmonary actinobacillosis |
| LowerRespiratoryTractInfection-WRpt | 1055 | 32286006 | Pneumonia in Q fever |
| LowerRespiratoryTractInfection-WRpt | 1055 | 33631007 | Pyopneumothorax |
| LowerRespiratoryTractInfection-WRpt | 1055 | 34020007 | Pneumonia due to Streptococcus |
| LowerRespiratoryTractInfection-WRpt | 1055 | 34286007 | Empyema with hepatopleural fistula |
| LowerRespiratoryTractInfection-WRpt | 1055 | 34290009 | Pulmonary nematodiasis |
| LowerRespiratoryTractInfection-WRpt | 1055 | 35037009 | Primary atypical interstitial pneumonia |
| LowerRespiratoryTractInfection-WRpt | 1055 | 35339003 | Primary pneumonic plague |
| LowerRespiratoryTractInfection-WRpt | 1055 | 36426008 | Subacute bronchitis |
| LowerRespiratoryTractInfection-WRpt | 1055 | 37721008 | AIDS with pneumococcal pneumonia |
| LowerRespiratoryTractInfection-WRpt | 1055 | 38699009 | Pneumonia due to Histoplasma capsulatum |
| LowerRespiratoryTractInfection-WRpt | 1055 | 38976008 | Pneumonic plague |
| LowerRespiratoryTractInfection-WRpt | 1055 | 39172002 | Pneumonia due to Proteus mirabilis |
| LowerRespiratoryTractInfection-WRpt | 1055 | 40600002 | Pneumococcal bronchitis |
| LowerRespiratoryTractInfection-WRpt | 1055 | 41207000 | Adenoviral pneumonia |
| LowerRespiratoryTractInfection-WRpt | 1055 | 41269000 | Influenzal bronchopneumonia |
| LowerRespiratoryTractInfection-WRpt | 1055 | 41381004 | Pneumonia due to Pseudomonas |
| LowerRespiratoryTractInfection-WRpt | 1055 | 42002000 | Avian infectious bronchitis |
| LowerRespiratoryTractInfection-WRpt | 1055 | 45263007 | Pulmonary sporotrichosis |
| LowerRespiratoryTractInfection-WRpt | 1055 | 45312009 | Pneumonia in typhoid fever |
| LowerRespiratoryTractInfection-WRpt | 1055 | 45556008 | Pulmonary tularaemia |
| LowerRespiratoryTractInfection-WRpt | 1055 | 46207001 | Pneumonitis due to acquired toxoplasmosis |
| LowerRespiratoryTractInfection-WRpt | 1055 | 46970008 | Mycoplasma pneumonia |
| LowerRespiratoryTractInfection-WRpt | 1055 | 47082005 | Congenital rubella pneumonitis |
| LowerRespiratoryTractInfection-WRpt | 1055 | 48722001 | Progressive pneumonia of sheep |
| LowerRespiratoryTractInfection-WRpt | 1055 | 50417007 | Lower respiratory tract infection |
| LowerRespiratoryTractInfection-WRpt | 1055 | 50648007 | Tropical eosinophilia |
| LowerRespiratoryTractInfection-WRpt | 1055 | 50694005 | Empyema with mediastinal fistula |
| LowerRespiratoryTractInfection-WRpt | 1055 | 50997000 | Pulmonary dirofilariasis |
| LowerRespiratoryTractInfection-WRpt | 1055 | 51530003 | Pneumonia due to Escherichia coli |
| LowerRespiratoryTractInfection-WRpt | 1055 | 52409006 | Bronchiolitis exudativa |
| LowerRespiratoryTractInfection-WRpt | 1055 | 53084003 | Bacterial pneumonia |
| LowerRespiratoryTractInfection-WRpt | 1055 | 56507008 | Tea-tasters' disease |
| LowerRespiratoryTractInfection-WRpt | 1055 | 57086000 | Enzootic pneumonia of calves |
| LowerRespiratoryTractInfection-WRpt | 1055 | 57089007 | Respiratory syncytial virus bronchiolitis |
| LowerRespiratoryTractInfection-WRpt | 1055 | 57541005 | Pulmonary pneumocystosis |
| LowerRespiratoryTractInfection-WRpt | 1055 | 57716000 | Brooder pneumonia |
| LowerRespiratoryTractInfection-WRpt | 1055 | 58524006 | Acute pulmonary histoplasmosis |
| LowerRespiratoryTractInfection-WRpt | 1055 | 58554001 | Empyema of pleura |
| LowerRespiratoryTractInfection-WRpt | 1055 | 58890000 | Adenoviral bronchopneumonia |
| LowerRespiratoryTractInfection-WRpt | 1055 | 59475000 | Pneumonia in pertussis |
| LowerRespiratoryTractInfection-WRpt | 1055 | 60363000 | Pneumonia |
| LowerRespiratoryTractInfection-WRpt | 1055 | 60485005 | Pleurobronchopneumonia |
| LowerRespiratoryTractInfection-WRpt | 1055 | 60696005 | AIDS with bacterial pneumonia |
| LowerRespiratoryTractInfection-WRpt | 1055 | 60837001 | Contagious bovine pleuropneumonia |
| LowerRespiratoryTractInfection-WRpt | 1055 | 60916008 | Cardiopulmonary schistosomiasis |
| LowerRespiratoryTractInfection-WRpt | 1055 | 61532009 | Enzootic pneumonia of sheep |
| LowerRespiratoryTractInfection-WRpt | 1055 | 61884008 | Achromobacter pneumonia |
| LowerRespiratoryTractInfection-WRpt | 1055 | 63741006 | Fungal infection of lung |
| LowerRespiratoryTractInfection-WRpt | 1055 | 64479007 | Pneumonia due to Klebsiella pneumoniae |
| LowerRespiratoryTractInfection-WRpt | 1055 | 64667001 | Interstitial pneumonia |
| LowerRespiratoryTractInfection-WRpt | 1055 | 64703005 | Terminal bronchopneumonia |
| LowerRespiratoryTractInfection-WRpt | 1055 | 64880000 | Parainfluenza virus bronchopneumonia |
| LowerRespiratoryTractInfection-WRpt | 1055 | 64912000 | Enzootic mycoplasmal pneumonia of swine |
| LowerRespiratoryTractInfection-WRpt | 1055 | 64917006 | Parainfluenza virus pneumonia |
| LowerRespiratoryTractInfection-WRpt | 1055 | 65095005 | Amoebic lung abscess |
| LowerRespiratoryTractInfection-WRpt | 1055 | 65102004 | Adiaspiromycosis due to Emmonsia parva |
| LowerRespiratoryTractInfection-WRpt | 1055 | 65878001 | Septic bronchitis |
| LowerRespiratoryTractInfection-WRpt | 1055 | 66429007 | Unresolved lobar pneumonia |
| LowerRespiratoryTractInfection-WRpt | 1055 | 67525007 | Secondary pneumonic plague |
| LowerRespiratoryTractInfection-WRpt | 1055 | 70036007 | Haemophilus influenzae pneumonia |
| LowerRespiratoryTractInfection-WRpt | 1055 | 71186008 | Croup |
| LowerRespiratoryTractInfection-WRpt | 1055 | 71255007 | Adenoviral laryngotracheobronchitis |
| LowerRespiratoryTractInfection-WRpt | 1055 | 71435009 | Chronic obstructive bronchitis |
| LowerRespiratoryTractInfection-WRpt | 1055 | 71926009 | Infective pneumonia acquired prenatally |
| LowerRespiratoryTractInfection-WRpt | 1055 | 73198007 | Bacterial pleurisy |
| LowerRespiratoryTractInfection-WRpt | 1055 | 73414003 | Haemophilus influenzae laryngotracheobronchitis |
| LowerRespiratoryTractInfection-WRpt | 1055 | 73452002 | Abscess of lung |
| LowerRespiratoryTractInfection-WRpt | 1055 | 73995006 | Pulmonary paracoccidioidomycosis |
| LowerRespiratoryTractInfection-WRpt | 1055 | 74387008 | Tuberculosis of hilar lymph nodes |
| LowerRespiratoryTractInfection-WRpt | 1055 | 74417001 | Mucopurulent chronic bronchitis |
| LowerRespiratoryTractInfection-WRpt | 1055 | 75388006 | Echinococcus granulosus infection of lung |
| LowerRespiratoryTractInfection-WRpt | 1055 | 75570004 | Viral pneumonia |
| LowerRespiratoryTractInfection-WRpt | 1055 | 75642009 | Bovine pneumonic pasteurellosis |
| LowerRespiratoryTractInfection-WRpt | 1055 | 76090006 | Pittsburgh pneumonia |
| LowerRespiratoryTractInfection-WRpt | 1055 | 76630008 | Fibrinopurulent pleurisy |
| LowerRespiratoryTractInfection-WRpt | 1055 | 78887005 | Seropurulent pleurisy |
| LowerRespiratoryTractInfection-WRpt | 1055 | 78895009 | Congenital pneumonia |
| LowerRespiratoryTractInfection-WRpt | 1055 | 79479005 | Respiratory syncytial virus bronchitis |
| LowerRespiratoryTractInfection-WRpt | 1055 | 80003002 | Tuberculous pneumonia |
| LowerRespiratoryTractInfection-WRpt | 1055 | 80010008 | Isolated bronchial tuberculosis |
| LowerRespiratoryTractInfection-WRpt | 1055 | 80257001 | Acute bronchitis with bronchospasm |
| LowerRespiratoryTractInfection-WRpt | 1055 | 80602006 | Nodular tuberculosis of lung |
| LowerRespiratoryTractInfection-WRpt | 1055 | 81164001 | Ornithosis with pneumonia |
| LowerRespiratoryTractInfection-WRpt | 1055 | 81554001 | Tuberculosis of lung with involvement of bronchus |
| LowerRespiratoryTractInfection-WRpt | 1055 | 81638006 | Contagious caprine pleuropneumonia |
| LowerRespiratoryTractInfection-WRpt | 1055 | 82670009 | Whooping cough due to organism other than Bordetella pertussis |
| LowerRespiratoryTractInfection-WRpt | 1055 | 84353005 | Pulmonary disease due to Mycobacteria |
| LowerRespiratoryTractInfection-WRpt | 1055 | 84659003 | Feline pneumonitis |
| LowerRespiratoryTractInfection-WRpt | 1055 | 84753008 | Pneumonia in systemic mycosis |
| LowerRespiratoryTractInfection-WRpt | 1055 | 85420008 | Streptococcal pleurisy |
| LowerRespiratoryTractInfection-WRpt | 1055 | 85426002 | Bacterial pleurisy with effusion |
| LowerRespiratoryTractInfection-WRpt | 1055 | 85469005 | Hypostatic pneumonia |
| LowerRespiratoryTractInfection-WRpt | 1055 | 85915003 | Laryngotracheobronchitis |
| LowerRespiratoryTractInfection-WRpt | 1055 | 86853002 | Feline infectious peritonitis AND pleuritis |
| LowerRespiratoryTractInfection-WRpt | 1055 | 87695000 | Necrotising bronchiolitis |
| LowerRespiratoryTractInfection-WRpt | 1055 | 88036000 | Primary pulmonary coccidioidomycosis |
| LowerRespiratoryTractInfection-WRpt | 1055 | 88693009 | AIDS with candidiasis of lung |
| LowerRespiratoryTractInfection-WRpt | 1055 | 89087009 | Porcine contagious pleuropneumonia |
| LowerRespiratoryTractInfection-WRpt | 1055 | 90117007 | Tuberculous fibrosis of lung |
| LowerRespiratoryTractInfection-WRpt | 1055 | 111849006 | Adenoviral bronchitis |
| LowerRespiratoryTractInfection-WRpt | 1055 | 111900000 | Pneumonia in aspergillosis |
| LowerRespiratoryTractInfection-WRpt | 1055 | 120639003 | Hantavirus pulmonary syndrome |
| LowerRespiratoryTractInfection-WRpt | 1055 | 123587001 | Acute bronchopneumonia |
| LowerRespiratoryTractInfection-WRpt | 1055 | 123588006 | Confluent bronchopneumonia with abscess formation |
| LowerRespiratoryTractInfection-WRpt | 1055 | 123589003 | Necrotising bronchopneumonia |
| LowerRespiratoryTractInfection-WRpt | 1055 | 123620007 | Hantanvirus pulmonary syndrome |
| LowerRespiratoryTractInfection-WRpt | 1055 | 128601007 | Infectious disease of lung |
| LowerRespiratoryTractInfection-WRpt | 1055 | 128940004 | Parasitic infection of lung |
| LowerRespiratoryTractInfection-WRpt | 1055 | 154283005 | Pulmonary tuberculosis |
| LowerRespiratoryTractInfection-WRpt | 1055 | 155510007 | Acute laryngotracheobronchitis |
| LowerRespiratoryTractInfection-WRpt | 1055 | 155548002 | Pneumonia and influenza &/or pneumonia |
| LowerRespiratoryTractInfection-WRpt | 1055 | 155552002 | Pneumonia NOS |
| LowerRespiratoryTractInfection-WRpt | 1055 | 155558003 | Pneumonia NOS |
| LowerRespiratoryTractInfection-WRpt | 1055 | 155570004 | Acute exacerbation of chronic obstructive airways disease |
| LowerRespiratoryTractInfection-WRpt | 1055 | 155571000 | Acute exacerbation of chronic obstructive airways disease |
| LowerRespiratoryTractInfection-WRpt | 1055 | 171699006 | Tuberculous pleuritis |
| LowerRespiratoryTractInfection-WRpt | 1055 | 186172004 | Tuberculous pleurisy in primary progressive tuberculosis |
| LowerRespiratoryTractInfection-WRpt | 1055 | 186175002 | Infiltrative lung tuberculosis |
| LowerRespiratoryTractInfection-WRpt | 1055 | 186177005 | Tuberculosis of lung with cavitation |
| LowerRespiratoryTractInfection-WRpt | 1055 | 186178000 | Tuberculosis of bronchus |
| LowerRespiratoryTractInfection-WRpt | 1055 | 186179008 | Other specified pulmonary tuberculosis |
| LowerRespiratoryTractInfection-WRpt | 1055 | 186180006 | Pulmonary tuberculosis NOS |
| LowerRespiratoryTractInfection-WRpt | 1055 | 186181005 | Other respiratory tuberculosis |
| LowerRespiratoryTractInfection-WRpt | 1055 | 186182003 | Tuberculosis of pleura |
| LowerRespiratoryTractInfection-WRpt | 1055 | 186186000 | Tuberculous pleurisy NOS |
| LowerRespiratoryTractInfection-WRpt | 1055 | 186188004 | Isolated tracheal or bronchial tuberculosis |
| LowerRespiratoryTractInfection-WRpt | 1055 | 186191004 | Isolated tracheal or bronchial tuberculosis NOS |
| LowerRespiratoryTractInfection-WRpt | 1055 | 186193001 | Tuberculosis of lung, confirmed by sputum microscopy with or without culture |
| LowerRespiratoryTractInfection-WRpt | 1055 | 186194007 | Tuberculosis of lung, confirmed by culture only |
| LowerRespiratoryTractInfection-WRpt | 1055 | 186195008 | Tuberculosis of lung, confirmed histologically |
| LowerRespiratoryTractInfection-WRpt | 1055 | 186197000 | Tuberculosis of lung, confirmed by unspecified means |
| LowerRespiratoryTractInfection-WRpt | 1055 | 186200004 | Tuberculous pleurisy, confirmed bacteriologically and histologically |
| LowerRespiratoryTractInfection-WRpt | 1055 | 186203002 | Tuberculosis of lung, bacteriologically and histologically negative |
| LowerRespiratoryTractInfection-WRpt | 1055 | 186204008 | Tuberculosis of lung, bacteriological and histological examination not done |
| LowerRespiratoryTractInfection-WRpt | 1055 | 186207001 | Other specified respiratory tuberculosis |
| LowerRespiratoryTractInfection-WRpt | 1055 | 186212000 | Other specified respiratory tuberculosis NOS |
| LowerRespiratoryTractInfection-WRpt | 1055 | 186283004 | Pneumonic plague, unspecified |
| LowerRespiratoryTractInfection-WRpt | 1055 | 186342000 | Pulmonary Mycobacterium avium complex infection |
| LowerRespiratoryTractInfection-WRpt | 1055 | 186353006 | Whooping cough - other specified organism |
| LowerRespiratoryTractInfection-WRpt | 1055 | 186355004 | Other whooping cough NOS |
| LowerRespiratoryTractInfection-WRpt | 1055 | 186356003 | Whooping cough NOS |
| LowerRespiratoryTractInfection-WRpt | 1055 | 187027001 | Acute pulmonary coccidioidomycosis |
| LowerRespiratoryTractInfection-WRpt | 1055 | 187042003 | Histoplasma capsulatum with pneumonia |
| LowerRespiratoryTractInfection-WRpt | 1055 | 187052004 | Pulmonary African histoplasmosis |
| LowerRespiratoryTractInfection-WRpt | 1055 | 187054003 | Pulmonary histoplasmosis |
| LowerRespiratoryTractInfection-WRpt | 1055 | 187061004 | Histoplasmosis with pneumonia |
| LowerRespiratoryTractInfection-WRpt | 1055 | 187066009 | Primary pulmonary blastomycosis |
| LowerRespiratoryTractInfection-WRpt | 1055 | 187069002 | Acute pulmonary blastomycosis |
| LowerRespiratoryTractInfection-WRpt | 1055 | 187134002 | Lung echinococcus granulosus |
| LowerRespiratoryTractInfection-WRpt | 1055 | 187196002 | Toxoplasma pneumonitis |
| LowerRespiratoryTractInfection-WRpt | 1055 | 187324001 | [X]Whooping cough due to other Bordetella species |
| LowerRespiratoryTractInfection-WRpt | 1055 | 187325000 | [X]Whooping cough, unspecified |
| LowerRespiratoryTractInfection-WRpt | 1055 | 187499004 | [X]Pulmonary histoplasmosis capsulati, unspecified |
| LowerRespiratoryTractInfection-WRpt | 1055 | 187500008 | [X]Other pulmonary aspergillosis |
| LowerRespiratoryTractInfection-WRpt | 1055 | 195702002 | Acute laryngotracheobronchitis |
| LowerRespiratoryTractInfection-WRpt | 1055 | 195712009 | Acute bronchitis and/or bronchiolitis |
| LowerRespiratoryTractInfection-WRpt | 1055 | 195714005 | Acute fibrinous bronchitis |
| LowerRespiratoryTractInfection-WRpt | 1055 | 195715006 | Acute membranous bronchitis |
| LowerRespiratoryTractInfection-WRpt | 1055 | 195717003 | Acute purulent bronchitis |
| LowerRespiratoryTractInfection-WRpt | 1055 | 195719000 | Acute pneumococcal bronchitis |
| LowerRespiratoryTractInfection-WRpt | 1055 | 195720006 | Acute streptococcal bronchitis |
| LowerRespiratoryTractInfection-WRpt | 1055 | 195721005 | Acute bronchitis caused by Haemophilus influenzae |
| LowerRespiratoryTractInfection-WRpt | 1055 | 195722003 | Acute Moraxella catarrhalis bronchitis |
| LowerRespiratoryTractInfection-WRpt | 1055 | 195725001 | Acute coxsackievirus bronchitis |
| LowerRespiratoryTractInfection-WRpt | 1055 | 195726000 | Acute parainfluenza virus bronchitis |
| LowerRespiratoryTractInfection-WRpt | 1055 | 195727009 | Acute respiratory syncytial virus bronchitis |
| LowerRespiratoryTractInfection-WRpt | 1055 | 195728004 | Acute bronchitis due to rhinovirus |
| LowerRespiratoryTractInfection-WRpt | 1055 | 195729007 | Acute echovirus bronchitis |
| LowerRespiratoryTractInfection-WRpt | 1055 | 195730002 | Subacute bronchitis unspecified |
| LowerRespiratoryTractInfection-WRpt | 1055 | 195731003 | Acute viral bronchitis unspecified |
| LowerRespiratoryTractInfection-WRpt | 1055 | 195732005 | Acute bacterial bronchitis unspecified |
| LowerRespiratoryTractInfection-WRpt | 1055 | 195733000 | Acute bronchitis NOS |
| LowerRespiratoryTractInfection-WRpt | 1055 | 195737004 | Acute exudative bronchiolitis |
| LowerRespiratoryTractInfection-WRpt | 1055 | 195739001 | Acute bronchiolitis due to respiratory syncytial virus |
| LowerRespiratoryTractInfection-WRpt | 1055 | 195740004 | Acute bronchiolitis due to other specified organisms |
| LowerRespiratoryTractInfection-WRpt | 1055 | 195741000 | Acute bronchiolitis NOS |
| LowerRespiratoryTractInfection-WRpt | 1055 | 195742007 | Acute lower respiratory tract infection |
| LowerRespiratoryTractInfection-WRpt | 1055 | 195743002 | Acute bronchitis or bronchiolitis NOS |
| LowerRespiratoryTractInfection-WRpt | 1055 | 195747001 | Chest cold |
| LowerRespiratoryTractInfection-WRpt | 1055 | 195881003 | Pneumonia due to respiratory syncytial virus |
| LowerRespiratoryTractInfection-WRpt | 1055 | 195882005 | Viral pneumonia NEC |
| LowerRespiratoryTractInfection-WRpt | 1055 | 195883000 | Viral pneumonia NOS |
| LowerRespiratoryTractInfection-WRpt | 1055 | 195885007 | Other bacterial pneumonia |
| LowerRespiratoryTractInfection-WRpt | 1055 | 195886008 | Group B streptococcal pneumonia |
| LowerRespiratoryTractInfection-WRpt | 1055 | 195887004 | Pneumonia due to other specified bacteria |
| LowerRespiratoryTractInfection-WRpt | 1055 | 195888009 | Proteus pneumonia |
| LowerRespiratoryTractInfection-WRpt | 1055 | 195889001 | Legionella pneumonia |
| LowerRespiratoryTractInfection-WRpt | 1055 | 195891009 | Pneumonia due to bacteria NOS |
| LowerRespiratoryTractInfection-WRpt | 1055 | 195892002 | Bacterial pneumonia NOS |
| LowerRespiratoryTractInfection-WRpt | 1055 | 195893007 | Pneumonia due to other specified organisms |
| LowerRespiratoryTractInfection-WRpt | 1055 | 195896004 | Pneumonia due to pleuropneumonia-like organism |
| LowerRespiratoryTractInfection-WRpt | 1055 | 195898003 | Pneumonia due to specified organism NOS |
| LowerRespiratoryTractInfection-WRpt | 1055 | 195900001 | Pneumonia due to measles |
| LowerRespiratoryTractInfection-WRpt | 1055 | 195902009 | Anthrax pneumonia |
| LowerRespiratoryTractInfection-WRpt | 1055 | 195903004 | Pneumonia with other systemic mycoses |
| LowerRespiratoryTractInfection-WRpt | 1055 | 195904005 | Pneumonia with coccidioidomycosis |
| LowerRespiratoryTractInfection-WRpt | 1055 | 195905006 | Pneumonia with histoplasmosis |
| LowerRespiratoryTractInfection-WRpt | 1055 | 195906007 | Pneumonia with systemic mycosis NOS |
| LowerRespiratoryTractInfection-WRpt | 1055 | 195907003 | Pneumonia with other infectious diseases EC |
| LowerRespiratoryTractInfection-WRpt | 1055 | 195908008 | Actinomycotic pneumonia |
| LowerRespiratoryTractInfection-WRpt | 1055 | 195909000 | Nocardial pneumonia |
| LowerRespiratoryTractInfection-WRpt | 1055 | 195911009 | Chickenpox pneumonia |
| LowerRespiratoryTractInfection-WRpt | 1055 | 195912002 | Pneumonia with other infectious diseases EC NOS |
| LowerRespiratoryTractInfection-WRpt | 1055 | 195913007 | Pneumonia with infectious diseases EC NOS |
| LowerRespiratoryTractInfection-WRpt | 1055 | 195915000 | Pneumonia due to unspecified organism |
| LowerRespiratoryTractInfection-WRpt | 1055 | 195916004 | Lobar pneumonia due to unspecified organism |
| LowerRespiratoryTractInfection-WRpt | 1055 | 195919006 | Postoperative pneumonia |
| LowerRespiratoryTractInfection-WRpt | 1055 | 195936003 | Bronchitis: [unspecified (& chest infection)] or [recurrent wheezy] |
| LowerRespiratoryTractInfection-WRpt | 1055 | 195938002 | Laryngotracheobronchitis |
| LowerRespiratoryTractInfection-WRpt | 1055 | 195947005 | Mucopurulent chronic bronchitis NOS |
| LowerRespiratoryTractInfection-WRpt | 1055 | 195949008 | Chronic asthmatic bronchitis |
| LowerRespiratoryTractInfection-WRpt | 1055 | 195951007 | Acute exacerbation of chronic obstructive airways disease |
| LowerRespiratoryTractInfection-WRpt | 1055 | 196001008 | Chronic obstructive pulmonary disease with acute lower respiratory infection |
| LowerRespiratoryTractInfection-WRpt | 1055 | 196002001 | Chronic obstructive pulmonary disease with acute exacerbation, unspecified |
| LowerRespiratoryTractInfection-WRpt | 1055 | 196063008 | Empyema with pleural fistula NOS |
| LowerRespiratoryTractInfection-WRpt | 1055 | 196064002 | Empyema with thoracic fistula NOS |
| LowerRespiratoryTractInfection-WRpt | 1055 | 196067009 | Loculated empyema |
| LowerRespiratoryTractInfection-WRpt | 1055 | 196070008 | Lung empyema NOS |
| LowerRespiratoryTractInfection-WRpt | 1055 | 196090004 | Bacterial pleurisy with effusion NOS |
| LowerRespiratoryTractInfection-WRpt | 1055 | 196092007 | Encysted pleurisy |
| LowerRespiratoryTractInfection-WRpt | 1055 | 196107009 | Abscess of lung and mediastinum |
| LowerRespiratoryTractInfection-WRpt | 1055 | 196108004 | Single lung abscess |
| LowerRespiratoryTractInfection-WRpt | 1055 | 196109007 | Multiple lung abscesses |
| LowerRespiratoryTractInfection-WRpt | 1055 | 196110002 | Gangrenous pneumonia |
| LowerRespiratoryTractInfection-WRpt | 1055 | 196112005 | Abscess of lung with pneumonia |
| LowerRespiratoryTractInfection-WRpt | 1055 | 196113000 | Abscess of lung NOS |
| LowerRespiratoryTractInfection-WRpt | 1055 | 196114006 | Abscess of lung and mediastinum NOS |
| LowerRespiratoryTractInfection-WRpt | 1055 | 196204006 | [X]Other viral pneumonia |
| LowerRespiratoryTractInfection-WRpt | 1055 | 196205007 | [X]Pneumonia due to other aerobic gram-negative bacteria |
| LowerRespiratoryTractInfection-WRpt | 1055 | 196206008 | [X]Other bacterial pneumonia |
| LowerRespiratoryTractInfection-WRpt | 1055 | 196207004 | [X]Pneumonia due to other specified infectious organisms |
| LowerRespiratoryTractInfection-WRpt | 1055 | 196208009 | [X]Pneumonia in bacterial diseases classified elsewhere |
| LowerRespiratoryTractInfection-WRpt | 1055 | 196209001 | [X]Pneumonia in viral diseases classified elsewhere |
| LowerRespiratoryTractInfection-WRpt | 1055 | 196210006 | [X]Pneumonia in mycoses classified elsewhere |
| LowerRespiratoryTractInfection-WRpt | 1055 | 196211005 | [X]Pneumonia in parasitic diseases classified elsewhere |
| LowerRespiratoryTractInfection-WRpt | 1055 | 196212003 | [X]Pneumonia in other diseases classified elsewhere |
| LowerRespiratoryTractInfection-WRpt | 1055 | 196213008 | [X]Other pneumonia, organism unspecified |
| LowerRespiratoryTractInfection-WRpt | 1055 | 196214002 | [X]Other acute lower respiratory infections |
| LowerRespiratoryTractInfection-WRpt | 1055 | 196215001 | [X]Acute bronchitis due to other specified organisms |
| LowerRespiratoryTractInfection-WRpt | 1055 | 196216000 | [X]Acute bronchiolitis due to other specified organisms |
| LowerRespiratoryTractInfection-WRpt | 1055 | 196248001 | [X]Suppurative and necrotic conditions of the lower respiratory tract |
| LowerRespiratoryTractInfection-WRpt | 1055 | 206283000 | Congenital staphylococcal pneumonia |
| LowerRespiratoryTractInfection-WRpt | 1055 | 206284006 | Congenital group A haemolytic streptococcal pneumonia |
| LowerRespiratoryTractInfection-WRpt | 1055 | 206285007 | Congenital group B haemolytic streptococcal pneumonia |
| LowerRespiratoryTractInfection-WRpt | 1055 | 206286008 | Congenital Escherichia coli pneumonia |
| LowerRespiratoryTractInfection-WRpt | 1055 | 206287004 | Congenital pseudomonal pneumonia |
| LowerRespiratoryTractInfection-WRpt | 1055 | 206289001 | Congenital chlamydial pneumonia |
| LowerRespiratoryTractInfection-WRpt | 1055 | 206290005 | Other specified congenital pneumonia |
| LowerRespiratoryTractInfection-WRpt | 1055 | 206291009 | Congenital pneumonia NOS |
| LowerRespiratoryTractInfection-WRpt | 1055 | 206359006 | Neonatal candidiasis of lung |
| LowerRespiratoryTractInfection-WRpt | 1055 | 206634009 | [X]Congenital pneumonia due to other bacterial agents |
| LowerRespiratoryTractInfection-WRpt | 1055 | 206635005 | [X]Congenital pneumonia due to other organisms |
| LowerRespiratoryTractInfection-WRpt | 1055 | 213225005 | Postoperative chest infection |
| LowerRespiratoryTractInfection-WRpt | 1055 | 233597004 | Chest infection - unspecified bronchitis |
| LowerRespiratoryTractInfection-WRpt | 1055 | 233598009 | Acute bacterial bronchitis |
| LowerRespiratoryTractInfection-WRpt | 1055 | 233599001 | Acute mycoplasmal bronchitis |
| LowerRespiratoryTractInfection-WRpt | 1055 | 233600003 | Acute chlamydial bronchitis |
| LowerRespiratoryTractInfection-WRpt | 1055 | 233601004 | Acute viral bronchitis |
| LowerRespiratoryTractInfection-WRpt | 1055 | 233602006 | Acute viral bronchiolitis |
| LowerRespiratoryTractInfection-WRpt | 1055 | 233603001 | Acute bronchiolitis due to adenovirus |
| LowerRespiratoryTractInfection-WRpt | 1055 | 233604007 | Pneumonia |
| LowerRespiratoryTractInfection-WRpt | 1055 | 233606009 | Atypical pneumonia |
| LowerRespiratoryTractInfection-WRpt | 1055 | 233607000 | Pneumococcal pneumonia |
| LowerRespiratoryTractInfection-WRpt | 1055 | 233608005 | Meningococcal pneumonia |
| LowerRespiratoryTractInfection-WRpt | 1055 | 233609002 | Chlamydial pneumonia |
| LowerRespiratoryTractInfection-WRpt | 1055 | 233610007 | Neonatal chlamydial pneumonia |
| LowerRespiratoryTractInfection-WRpt | 1055 | 233613009 | Fungal pneumonia |
| LowerRespiratoryTractInfection-WRpt | 1055 | 233614003 | Pulmonary mucormycosis |
| LowerRespiratoryTractInfection-WRpt | 1055 | 233615002 | Chronic pulmonary coccidioidomycosis |
| LowerRespiratoryTractInfection-WRpt | 1055 | 233616001 | Pulmonary blastomycosis |
| LowerRespiratoryTractInfection-WRpt | 1055 | 233617005 | Haemorrhagic pneumonia |
| LowerRespiratoryTractInfection-WRpt | 1055 | 233618000 | Mycobacterial pneumonia |
| LowerRespiratoryTractInfection-WRpt | 1055 | 233619008 | Neonatal pneumonia |
| LowerRespiratoryTractInfection-WRpt | 1055 | 233620002 | Pneumonia due to parasitic infestation |
| LowerRespiratoryTractInfection-WRpt | 1055 | 233621003 | Rickettsial pneumonia |
| LowerRespiratoryTractInfection-WRpt | 1055 | 233622005 | Infectious mononucleosis pneumonia |
| LowerRespiratoryTractInfection-WRpt | 1055 | 233623000 | Mononuclear interstitial pneumonia |
| LowerRespiratoryTractInfection-WRpt | 1055 | 233624006 | Herpes simplex pneumonia |
| LowerRespiratoryTractInfection-WRpt | 1055 | 233625007 | Giant cell pneumonia |
| LowerRespiratoryTractInfection-WRpt | 1055 | 233626008 | Chronic bronchial sepsis |
| LowerRespiratoryTractInfection-WRpt | 1055 | 233649005 | Tuberculous chylothorax |
| LowerRespiratoryTractInfection-WRpt | 1055 | 233671000 | Chronic obstructive pulmonary disease with acute exacerbation, unspecified |
| LowerRespiratoryTractInfection-WRpt | 1055 | 233785003 | Infectious disorder of trachea |
| LowerRespiratoryTractInfection-WRpt | 1055 | 233794009 | Tuberculous bronchopleural fistula |
| LowerRespiratoryTractInfection-WRpt | 1055 | 233795005 | Infectious disorder of bronchus |
| LowerRespiratoryTractInfection-WRpt | 1055 | 233797002 | Infected bronchogenic cyst |
| LowerRespiratoryTractInfection-WRpt | 1055 | 240387006 | Pulmonary glanders |
| LowerRespiratoryTractInfection-WRpt | 1055 | 240391001 | Pulmonary melioidosis |
| LowerRespiratoryTractInfection-WRpt | 1055 | 240422004 | Tracheobronchial diphtheria |
| LowerRespiratoryTractInfection-WRpt | 1055 | 240629003 | Malarial shock lung |
| LowerRespiratoryTractInfection-WRpt | 1055 | 240635003 | Leishmanial pneumonia |
| LowerRespiratoryTractInfection-WRpt | 1055 | 240702004 | Chronic necrotising pulmonary aspergillosis |
| LowerRespiratoryTractInfection-WRpt | 1055 | 240705002 | Candidiasis of trachea |
| LowerRespiratoryTractInfection-WRpt | 1055 | 240741002 | Acute pulmonary African histoplasmosis |
| LowerRespiratoryTractInfection-WRpt | 1055 | 240742009 | Chronic pulmonary African histoplasmosis |
| LowerRespiratoryTractInfection-WRpt | 1055 | 240747003 | Chronic pulmonary blastomycosis |
| LowerRespiratoryTractInfection-WRpt | 1055 | 266339003 | Chest infection NOS |
| LowerRespiratoryTractInfection-WRpt | 1055 | 266350000 | Pneumococcal lobar pneumonia |
| LowerRespiratoryTractInfection-WRpt | 1055 | 266351001 | Pneumonia with infectious diseases EC |
| LowerRespiratoryTractInfection-WRpt | 1055 | 266352008 | Bronchopneumonia due to unspecified organism |
| LowerRespiratoryTractInfection-WRpt | 1055 | 266391003 | Pneumonia and influenza &/or pneumonia |
| LowerRespiratoryTractInfection-WRpt | 1055 | 271503005 | Empyema with fistula |
| LowerRespiratoryTractInfection-WRpt | 1055 | 271504004 | Pleural empyema with no fistula |
| LowerRespiratoryTractInfection-WRpt | 1055 | 271506002 | Pleural empyema NOS |
| LowerRespiratoryTractInfection-WRpt | 1055 | 271567008 | Whooping cough-like syndrome |
| LowerRespiratoryTractInfection-WRpt | 1055 | 274103002 | Pneumonia NOS |
| LowerRespiratoryTractInfection-WRpt | 1055 | 275497007 | Infection of lower respiratory tract and mediastinum |
| LowerRespiratoryTractInfection-WRpt | 1055 | 275499005 | Acute wheezy bronchitis |
| LowerRespiratoryTractInfection-WRpt | 1055 | 276692000 | Congenital viral pneumonia |
| LowerRespiratoryTractInfection-WRpt | 1055 | 276693005 | Congenital bacterial pneumonia |
| LowerRespiratoryTractInfection-WRpt | 1055 | 276694004 | Acquired neonatal pneumonia |
| LowerRespiratoryTractInfection-WRpt | 1055 | 277869007 | Non-tuberculous mycobacterial pneumonia |
| LowerRespiratoryTractInfection-WRpt | 1055 | 278484009 | Tropical pulmonary eosinophilia |
| LowerRespiratoryTractInfection-WRpt | 1055 | 278516003 | Lobar pneumonia |
| LowerRespiratoryTractInfection-WRpt | 1055 | 285381006 | Acute infective exacerbation of chronic obstructive airways disease |
| LowerRespiratoryTractInfection-WRpt | 1055 | 300999006 | Basal pneumonia |
| LowerRespiratoryTractInfection-WRpt | 1055 | 301000005 | Left lower zone pneumonia |
| LowerRespiratoryTractInfection-WRpt | 1055 | 301001009 | Right lower zone pneumonia |
| LowerRespiratoryTractInfection-WRpt | 1055 | 301002002 | Left upper zone pneumonia |
| LowerRespiratoryTractInfection-WRpt | 1055 | 301003007 | Right middle zone pneumonia |
| LowerRespiratoryTractInfection-WRpt | 1055 | 301004001 | Right upper zone pneumonia |
| LowerRespiratoryTractInfection-WRpt | 1055 | 307763005 | Basal pneumonia due to unspecified organism |
| LowerRespiratoryTractInfection-WRpt | 1055 | 308130008 | Recurrent chest infection |
| LowerRespiratoryTractInfection-WRpt | 1055 | 308906005 | Secondary bacterial pneumonia |
| LowerRespiratoryTractInfection-WRpt | 1055 | 312119006 | Bacterial lower respiratory infection |
| LowerRespiratoryTractInfection-WRpt | 1055 | 312134000 | Viral lower respiratory infection |
| LowerRespiratoryTractInfection-WRpt | 1055 | 312342009 | Infective pneumonia |
| LowerRespiratoryTractInfection-WRpt | 1055 | 312371005 | Acute infective bronchitis |
| LowerRespiratoryTractInfection-WRpt | 1055 | 312403005 | Legionnaire's disease |
| LowerRespiratoryTractInfection-WRpt | 1055 | 313433007 | Tuberculous cavity of lung |
| LowerRespiratoryTractInfection-WRpt | 1055 | 314042000 | Infective pleurisy |
| LowerRespiratoryTractInfection-WRpt | 1055 | 314043005 | Viral pleurisy |
| LowerRespiratoryTractInfection-WRpt | 1055 | 314978007 | Postoperative pneumonia |
| LowerRespiratoryTractInfection-WRpt | 1055 | 373435003 | Battey disease |
| LowerRespiratoryTractInfection-WRpt | 1055 | 385093006 | Community acquired pneumonia |
| LowerRespiratoryTractInfection-WRpt | 1055 | 385479009 | Follicular bronchiolitis |
| LowerRespiratoryTractInfection-WRpt | 1055 | 389075004 | Tuberculous pneumonia of humans |
| LowerRespiratoryTractInfection-WRpt | 1055 | 389077007 | Tuberculous pneumonia of animals |
| LowerRespiratoryTractInfection-WRpt | 1055 | 396284006 | Lobular pneumonia |
| LowerRespiratoryTractInfection-WRpt | 1055 | 396285007 | Bronchopneumonia |
| LowerRespiratoryTractInfection-WRpt | 1055 | 396286008 | Bilateral bronchopneumonia |
| LowerRespiratoryTractInfection-WRpt | 1055 | 397190009 | Respiratory tuberculosis |
| LowerRespiratoryTractInfection-WRpt | 1055 | 398447004 | Severe acute respiratory syndrome |
| LowerRespiratoryTractInfection-WRpt | 1055 | 407671000 | Bilateral pneumonia |
| LowerRespiratoryTractInfection-WRpt | 1055 | 408679000 | Healthcare associated pneumonia |
| LowerRespiratoryTractInfection-WRpt | 1055 | 408680002 | Healthcare associated bacterial pneumonia |
| LowerRespiratoryTractInfection-WRpt | 1055 | 408681003 | Healthcare associated legionnaire's disease |
| LowerRespiratoryTractInfection-WRpt | 1055 | 408682005 | Healthcare associated pertussis |
| LowerRespiratoryTractInfection-WRpt | 1055 | 408683000 | Healthcare associated pulmonary aspergillosis |
| LowerRespiratoryTractInfection-WRpt | 1055 | 408688009 | Healthcare associated severe acute respiratory syndrome |
| LowerRespiratoryTractInfection-WRpt | 1055 | 409664000 | Pneumonia due to anaerobic bacteria |
| LowerRespiratoryTractInfection-WRpt | 1055 | 409665004 | Pneumonia due to aerobic bacteria |
| LowerRespiratoryTractInfection-WRpt | 1055 | 415125002 | Pneumocystosis pneumonia |
| LowerRespiratoryTractInfection-WRpt | 1055 | 417018008 | Pulmonary coccidioidomycosis |
| LowerRespiratoryTractInfection-WRpt | 1055 | 417688002 | Chronic progressive coccidioidal pneumonia |
| LowerRespiratoryTractInfection-WRpt | 1055 | 418122003 | Bronchomoniliasis |
| LowerRespiratoryTractInfection-WRpt | 1055 | 419502003 | Chest infection |
| LowerRespiratoryTractInfection-WRpt | 1055 | 420245002 | Pneumonia due to Mannheimia haemolytica |
| LowerRespiratoryTractInfection-WRpt | 1055 | 420544002 | Bacterial pneumonia with AIDS (acquired immunodeficiency syndrome) |
| LowerRespiratoryTractInfection-WRpt | 1055 | 420787001 | Pneumococcal pneumonia with AIDS (acquired immunodeficiency syndrome) |
| LowerRespiratoryTractInfection-WRpt | 1055 | 421047005 | Candidiasis of lung with AIDS (acquired immunodeficiency syndrome) |
| LowerRespiratoryTractInfection-WRpt | 1055 | 421217001 | Enzootic pneumonia of sheep |
| LowerRespiratoryTractInfection-WRpt | 1055 | 421508002 | Viral pneumonia with AIDS (acquired immunodeficiency syndrome) |
| LowerRespiratoryTractInfection-WRpt | 1055 | 421724004 | Bovine respiratory disease complex |
| LowerRespiratoryTractInfection-WRpt | 1055 | 425464007 | Hospital acquired pneumonia |
| LowerRespiratoryTractInfection-WRpt | 1055 | 425996009 | Bilateral basal pneumonia |
| LowerRespiratoryTractInfection-WRpt | 1055 | 426696003 | Lingular pneumonia |
| LowerRespiratoryTractInfection-WRpt | 1055 | 428697002 | Inactive tuberculosis of lung |
| LowerRespiratoryTractInfection-WRpt | 1055 | 430395005 | Pneumonia due to Gram negative bacteria |
| LowerRespiratoryTractInfection-WRpt | 1055 | 430476004 | Diffuse panbronchiolitis |
| LowerRespiratoryTractInfection-WRpt | 1055 | 438764004 | Postoperative aspiration pneumonia |
| LowerRespiratoryTractInfection-WRpt | 1055 | 441590008 | Pneumonia due to Severe acute respiratory syndrome coronavirus |
| LowerRespiratoryTractInfection-WRpt | 1055 | 441658007 | Pneumonia due to Staphylococcus aureus |
| LowerRespiratoryTractInfection-WRpt | 1055 | 441942006 | Pneumonia due to infection by Streptococcus pyogenes |
| LowerRespiratoryTractInfection-WRpt | 1055 | 442094008 | Pneumonia due to Histoplasma |
| LowerRespiratoryTractInfection-WRpt | 1055 | 443378001 | Lady Windermere syndrome |
| LowerRespiratoryTractInfection-WRpt | 1055 | 445058002 | Aspergillus bronchitis |
| LowerRespiratoryTractInfection-WRpt | 1055 | 445096001 | Pneumonia due to Human metapneumovirus |
| LowerRespiratoryTractInfection-WRpt | 1055 | 445102008 | Bronchiolitis due to Human metapneumovirus |
| LowerRespiratoryTractInfection-WRpt | 1055 | 446543007 | Tuberculous abscess of lung |
| LowerRespiratoryTractInfection-WRpt | 1055 | 446946005 | Reinfection pulmonary tuberculosis |
| LowerRespiratoryTractInfection-WRpt | 1055 | 446986002 | Tuberculous pleural effusion |
| LowerRespiratoryTractInfection-WRpt | 1055 | 447006007 | Relapse pulmonary tuberculosis |
| LowerRespiratoryTractInfection-WRpt | 1055 | 448719004 | Postoperative lower respiratory tract infection |
| LowerRespiratoryTractInfection-WRpt | 1055 | 448739000 | Recurrent lower respiratory tract infection |
| LowerRespiratoryTractInfection-WRpt | 1055 | 700273003 | Isolated tracheobronchial tuberculosis |
| LowerRespiratoryTractInfection-WRpt | 1055 | 704345008 | Chronic interstitial pneumonia |
| LowerRespiratoryTractInfection-WRpt | 1055 | 707503004 | Pneumonia due to Schistosoma mansoni |
| LowerRespiratoryTractInfection-WRpt | 1055 | 707507003 | Pneumonia due to Schistosoma japonicum |
| LowerRespiratoryTractInfection-WRpt | 1055 | 707508008 | Pneumonia due to Schistosoma haematobium |
| LowerRespiratoryTractInfection-WRpt | 1055 | 708025003 | Pyopneumothorax following infection by Coccidioides |
| LowerRespiratoryTractInfection-WRpt | 1055 | 713084008 | Pneumonia caused by Human coronavirus |
| LowerRespiratoryTractInfection-WRpt | 1055 | 713525001 | Recurrent bacterial pneumonia |
| LowerRespiratoryTractInfection-WRpt | 1055 | 713526000 | Recurrent bacterial pneumonia co-occurrent with human immunodeficiency virus infection |
| LowerRespiratoryTractInfection-WRpt | 1055 | 713544008 | Bacterial pneumonia co-occurrent with human immunodeficiency virus infection |
| LowerRespiratoryTractInfection-WRpt | 1055 | 714203003 | Acute bronchitis co-occurrent with bronchiectasis |
| LowerRespiratoryTractInfection-WRpt | 1055 | 715882005 | Severe acute respiratory syndrome of upper respiratory tract |
| LowerRespiratoryTractInfection-WRpt | 1055 | 721804002 | Infection of lung caused by Pneumocystis |
| LowerRespiratoryTractInfection-WRpt | 1055 | 724498004 | Pneumonia caused by Chlamydia pneumoniae |
| LowerRespiratoryTractInfection-WRpt | 1055 | 733051000 | Pneumonia caused by Gram positive bacteria |
| LowerRespiratoryTractInfection-WRpt | 1055 | 733171006 | Chronic pulmonary aspergillosis |
| LowerRespiratoryTractInfection-WRpt | 1055 | 733497009 | Chronic suppuration of bronchus |
| LowerRespiratoryTractInfection-WRpt | 1055 | 735464006 | Acute noninfective bronchitis |
| LowerRespiratoryTractInfection-WRpt | 1055 | 735465007 | Protracted bronchitis caused by bacterium |
| LowerRespiratoryTractInfection-WRpt | 1055 | 735532001 | Infection of lung caused by Echinococcus |
| LowerRespiratoryTractInfection-WRpt | 1055 | 737180005 | Chronic bronchiolitis |
| LowerRespiratoryTractInfection-WRpt | 1055 | 763888005 | Necrotising pneumonia caused by Panton-Valentine leukocidin producing Staphylococcus aureus |
| LowerRespiratoryTractInfection-WRpt | 1055 | 770674007 | Ghon complex |
| LowerRespiratoryTractInfection-WRpt | 1055 | 772839003 | Pneumonia caused by Influenza A virus |
| LowerRespiratoryTractInfection-WRpt | 1055 | 782761005 | Subacute invasive pulmonary aspergillosis |
| LowerRespiratoryTractInfection-WRpt | 1055 | 785745000 | Acute bronchitis co-occurrent with wheeze |
| LowerRespiratoryTractInfection-WRpt | 1055 | 788997004 | Obstructing Aspergillus tracheobronchitis |
| LowerRespiratoryTractInfection-WRpt | 1055 | 870573008 | Interstitial pneumonia with autoimmune features |
| LowerRespiratoryTractInfection-WRpt | 1055 | 1010615002 | Late syphilis of lung |
| LowerRespiratoryTractInfection-WRpt | 1055 | 1010620002 | Infection of lung caused by Mycobacterium malmoense |
| LowerRespiratoryTractInfection-WRpt | 1055 | 1010622005 | Infection of lung caused by Mycobacterium xenopi |
| LowerRespiratoryTractInfection-WRpt | 1055 | 1010634002 | Pneumonia caused by Acinetobacter |
| LowerRespiratoryTractInfection-WRpt | 1055 | 1010662009 | Infection of lung caused by Mycobacterium kansasii |
| LowerRespiratoryTractInfection-WRpt | 1055 | 1149093006 | Pneumonia caused by vancomycin resistant Enterococcus |
| LowerRespiratoryTractInfection-WRpt | 1055 | 1163147006 | Chronic fibrosing pulmonary aspergillosis |
| LowerRespiratoryTractInfection-WRpt | 1055 | 1163150009 | Chronic cavitary pulmonary aspergillosis |
| LowerRespiratoryTractInfection-WRpt | 1055 | 1163489008 | Human metapneumovirus bronchitis |
| LowerRespiratoryTractInfection-WRpt | 1055 | 1176988004 | Enterobacter pneumonia |
| LowerRespiratoryTractInfection-WRpt | 1055 | 1177000004 | Pulmonary nodule caused by Pneumocystis |
| LowerRespiratoryTractInfection-WRpt | 1055 | 1177059007 | Pneumocystis jirovecii lung cyst |
| LowerRespiratoryTractInfection-WRpt | 1055 | 1187256004 | Viral pneumonia due to Epstein-Barr virus infectious mononucleosis |
| LowerRespiratoryTractInfection-WRpt | 1055 | 1208602000 | Pneumonia caused by Pseudomonas aeruginosa |
| LowerRespiratoryTractInfection-WRpt | 1055 | 1731000119106 | Atypical mycobacterial infection of lung |
| LowerRespiratoryTractInfection-WRpt | 1055 | 12571000132104 | Pneumonitis due to Herpes zoster |
| LowerRespiratoryTractInfection-WRpt | 1055 | 16311000119108 | Pneumonia due to influenza |
| LowerRespiratoryTractInfection-WRpt | 1055 | 28791000119105 | Chronic coccidioidomycotic pneumonia |
| LowerRespiratoryTractInfection-WRpt | 1055 | 33601000087105 | Acute bronchiolitis caused by chemical fumes |
| LowerRespiratoryTractInfection-WRpt | 1055 | 106001000119101 | Chronic obstructive lung disease co-occurrent with acute bronchitis |
| LowerRespiratoryTractInfection-WRpt | 1055 | 124691000119101 | Pneumonia due to methicillin resistant Staphylococcus aureus |
| LowerRespiratoryTractInfection-WRpt | 1055 | 128711000119106 | Pneumonia due to methicillin susceptible Staphylococcus aureus |
| LowerRespiratoryTractInfection-WRpt | 1055 | 142931000119100 | Pneumonia due to H1N1 influenza |
| LowerRespiratoryTractInfection-WRpt | 1055 | 143111000119103 | Pneumonia due to avian influenza |
| LowerRespiratoryTractInfection-WRpt | 1055 | 184431000119108 | Acute pneumonia due to coccidioidomycosis |
| LowerRespiratoryTractInfection-WRpt | 1055 | 243981000119109 | Pulmonary filariasis |
| LowerRespiratoryTractInfection-WRpt | 1055 | 309421000000105 | Pulmonary blastomycosis |
| LowerRespiratoryTractInfection-WRpt | 1055 | 400631000000109 | [X]Other pneumonia, organism unspecified |
| LowerRespiratoryTractInfection-WRpt | 1055 | 402451000000104 | [X]Pulmonary histoplasmosis capsulati, unspecified |
| LowerRespiratoryTractInfection-WRpt | 1055 | 402461000000101 | [X]Other pulmonary aspergillosis |
| LowerRespiratoryTractInfection-WRpt | 1055 | 409081000000107 | [X]Congenital pneumonia due to other bacterial agents |
| LowerRespiratoryTractInfection-WRpt | 1055 | 412671000000108 | [X]Other acute lower respiratory infections |
| LowerRespiratoryTractInfection-WRpt | 1055 | 414341000000100 | [X]Pneumonia in viral diseases classified elsewhere |
| LowerRespiratoryTractInfection-WRpt | 1055 | 418961000000106 | [X]Acute bronchiolitis due to other specified organisms |
| LowerRespiratoryTractInfection-WRpt | 1055 | 429381000000103 | [X]Other viral pneumonia |
| LowerRespiratoryTractInfection-WRpt | 1055 | 431681000000108 | [X]Acute bronchitis due to other specified organisms |
| LowerRespiratoryTractInfection-WRpt | 1055 | 432151000000100 | [X]Pneumonia in bacterial diseases classified elsewhere |
| LowerRespiratoryTractInfection-WRpt | 1055 | 441141000000108 | [X]Pneumonia due to other aerobic gram-negative bacteria |
| LowerRespiratoryTractInfection-WRpt | 1055 | 442341000000102 | [X]Pneumonia in other diseases classified elsewhere |
| LowerRespiratoryTractInfection-WRpt | 1055 | 444061000000106 | [X]Whooping cough due to other Bordetella species |
| LowerRespiratoryTractInfection-WRpt | 1055 | 454431000000109 | [X]Pneumonia due to other aerobic gram-negative bacteria |
| LowerRespiratoryTractInfection-WRpt | 1055 | 456031000000108 | [X]Pneumonia in mycoses classified elsewhere |
| LowerRespiratoryTractInfection-WRpt | 1055 | 456041000000104 | [X]Whooping cough, unspecified |
| LowerRespiratoryTractInfection-WRpt | 1055 | 468671000000107 | [X]Other bacterial pneumonia |
| LowerRespiratoryTractInfection-WRpt | 1055 | 469801000000105 | [X]Pneumonia in parasitic diseases classified elsewhere |
| LowerRespiratoryTractInfection-WRpt | 1055 | 471471000000106 | [X]Pneumonia due to other specified infectious organisms |
| LowerRespiratoryTractInfection-WRpt | 1055 | 478351000000102 | [X]Congenital pneumonia due to other organisms |
| LowerRespiratoryTractInfection-WRpt | 1055 | 526041000000105 | Aspergillus bronchitis |
| LowerRespiratoryTractInfection-WRpt | 1055 | 532291000000105 | Pneumonia with infectious diseases EC |
| LowerRespiratoryTractInfection-WRpt | 1055 | 546371000000104 | Whooping cough NOS |
| LowerRespiratoryTractInfection-WRpt | 1055 | 554071000000106 | Isolated tracheal or bronchial tuberculosis NOS |
| LowerRespiratoryTractInfection-WRpt | 1055 | 554081000000108 | Tuberculosis of lung, confirmed by unspecified means |
| LowerRespiratoryTractInfection-WRpt | 1055 | 555361000000102 | Other whooping cough NOS |
| LowerRespiratoryTractInfection-WRpt | 1055 | 564541000000102 | Other specified pulmonary tuberculosis |
| LowerRespiratoryTractInfection-WRpt | 1055 | 564611000000104 | Other specified respiratory tuberculosis |
| LowerRespiratoryTractInfection-WRpt | 1055 | 564621000000105 | Other specified respiratory tuberculosis NOS |
| LowerRespiratoryTractInfection-WRpt | 1055 | 566801000000107 | Other specified congenital pneumonia |
| LowerRespiratoryTractInfection-WRpt | 1055 | 570791000000100 | Whooping cough - other specified organism |
| LowerRespiratoryTractInfection-WRpt | 1055 | 579411000000108 | Pulmonary tuberculosis NOS |
| LowerRespiratoryTractInfection-WRpt | 1055 | 579421000000102 | Other respiratory tuberculosis |
| LowerRespiratoryTractInfection-WRpt | 1055 | 579431000000100 | Tuberculous pleurisy NOS |
| LowerRespiratoryTractInfection-WRpt | 1055 | 603071000000106 | Pneumonia due to specified organism NOS |
| LowerRespiratoryTractInfection-WRpt | 1055 | 603111000000100 | Pneumonia with other infectious diseases EC |
| LowerRespiratoryTractInfection-WRpt | 1055 | 603201000000106 | Congenital pneumonia NOS |
| LowerRespiratoryTractInfection-WRpt | 1055 | 616081000000102 | Pneumonia due to other specified organisms |
| LowerRespiratoryTractInfection-WRpt | 1055 | 616091000000100 | Pneumonia with other systemic mycoses |
| LowerRespiratoryTractInfection-WRpt | 1055 | 616101000000108 | Pneumonia with systemic mycosis NOS |
| LowerRespiratoryTractInfection-WRpt | 1055 | 616111000000105 | Pneumonia with other infectious diseases EC NOS |
| LowerRespiratoryTractInfection-WRpt | 1055 | 616121000000104 | Lung empyema NOS |
| LowerRespiratoryTractInfection-WRpt | 1055 | 616131000000102 | Pneumonia with infectious diseases EC NOS |
| LowerRespiratoryTractInfection-WRpt | 1055 | 616141000000106 | Pneumonia due to unspecified organism |
| LowerRespiratoryTractInfection-WRpt | 1055 | 617941000000107 | Viral pneumonia NEC |
| LowerRespiratoryTractInfection-WRpt | 1055 | 617951000000105 | Viral pneumonia NOS |
| LowerRespiratoryTractInfection-WRpt | 1055 | 617961000000108 | Pneumonia due to other specified bacteria |
| LowerRespiratoryTractInfection-WRpt | 1055 | 617971000000101 | Pneumonia due to bacteria NOS |
| LowerRespiratoryTractInfection-WRpt | 1055 | 617981000000104 | Bacterial pneumonia NOS |
| LowerRespiratoryTractInfection-WRpt | 1055 | 621011000000106 | Acute bronchitis NOS |
| LowerRespiratoryTractInfection-WRpt | 1055 | 621021000000100 | Acute bronchiolitis due to other specified organisms |
| LowerRespiratoryTractInfection-WRpt | 1055 | 621031000000103 | Acute bronchiolitis NOS |
| LowerRespiratoryTractInfection-WRpt | 1055 | 623451000000102 | Other bacterial pneumonia |
| LowerRespiratoryTractInfection-WRpt | 1055 | 627061000000102 | Empyema with pleural fistula NOS |
| LowerRespiratoryTractInfection-WRpt | 1055 | 627071000000109 | Empyema with thoracic fistula NOS |
| LowerRespiratoryTractInfection-WRpt | 1055 | 627101000000100 | Bacterial pleurisy with effusion NOS |
| LowerRespiratoryTractInfection-WRpt | 1055 | 644261000000103 | Chest infection - unspecified bronchitis |
| LowerRespiratoryTractInfection-WRpt | 1055 | 644271000000105 | Chronic obstructive pulmonary disease with acute exacerbation, unspecified |
| LowerRespiratoryTractInfection-WRpt | 1055 | 649141000000105 | Mucopurulent chronic bronchitis NOS |
| LowerRespiratoryTractInfection-WRpt | 1055 | 651931000000103 | Subacute bronchitis unspecified |
| LowerRespiratoryTractInfection-WRpt | 1055 | 651941000000107 | Acute viral bronchitis unspecified |
| LowerRespiratoryTractInfection-WRpt | 1055 | 651951000000105 | Acute bacterial bronchitis unspecified |
| LowerRespiratoryTractInfection-WRpt | 1055 | 652331000000107 | Basal pneumonia due to unspecified organism |
| LowerRespiratoryTractInfection-WRpt | 1055 | 654461000000102 | Pneumonic plague, unspecified |
| LowerRespiratoryTractInfection-WRpt | 1055 | 656891000000102 | Lobar pneumonia due to unspecified organism |
| LowerRespiratoryTractInfection-WRpt | 1055 | 677801000000109 | Pneumonia NOS |
| LowerRespiratoryTractInfection-WRpt | 1055 | 686531000000105 | Abscess of lung NOS |
| LowerRespiratoryTractInfection-WRpt | 1055 | 686541000000101 | Abscess of lung and mediastinum NOS |
| LowerRespiratoryTractInfection-WRpt | 1055 | 689401000000108 | Bronchopneumonia due to unspecified organism |
| LowerRespiratoryTractInfection-WRpt | 1055 | 701361000000105 | Chest infection NOS |
| LowerRespiratoryTractInfection-WRpt | 1055 | 701461000000102 | Pleural empyema NOS |
| LowerRespiratoryTractInfection-WRpt | 1055 | 705871000000109 | Acute bronchitis or bronchiolitis NOS |
| LowerRespiratoryTractInfection-WRpt | 1055 | 706961000000100 | Aspergillus bronchitis |
| LowerRespiratoryTractInfection-WRpt | 1055 | 707151000000103 | Aspergilloma |
| LowerRespiratoryTractInfection-WRpt | 1055 | 810711000000102 | Acute bronchiolitis due to human metapneumovirus |
| LowerRespiratoryTractInfection-WRpt | 1055 | 810721000000108 | Pneumonia due to Human metapneumovirus |
| LowerRespiratoryTractInfection-WRpt | 1055 | 812311000000102 | Hantavirus pulmonary syndrome |
| LowerRespiratoryTractInfection-WRpt | 1055 | 823141000000101 | Community acquired pneumonia |
| LowerRespiratoryTractInfection-WRpt | 1055 | 823151000000103 | Hospital acquired pneumonia |
| LowerRespiratoryTractInfection-WRpt | 1055 | 831801000000108 | Acute bronchiolitis due to human metapneumovirus |
| LowerRespiratoryTractInfection-WRpt | 1055 | 831811000000105 | Pneumonia due to human metapneumovirus |
| LowerRespiratoryTractInfection-WRpt | 1055 | 832441000000100 | Hantavirus pulmonary syndrome |
| LowerRespiratoryTractInfection-WRpt | 1055 | 834851000000106 | Community acquired pneumonia |
| LowerRespiratoryTractInfection-WRpt | 1055 | 834861000000109 | Hospital acquired pneumonia |
| LowerRespiratoryTractInfection-WRpt | 1055 | 1033131000000107 | Pneumonia due to Gram negative bacteria |
| LowerRespiratoryTractInfection-WRpt | 1055 | 1033141000000103 | Pneumonia due to Gram negative bacteria |
| LowerRespiratoryTractInfection-WRpt | 1055 | 1082721000119101 | Pneumonia due to Ascaris |
| LowerRespiratoryTractInfection-WRpt | 1055 | 1087061000119106 | Gonococcal pneumonia |
| LowerRespiratoryTractInfection-WRpt | 1055 | 1092361000119109 | Rubella pneumonia |
| LowerRespiratoryTractInfection-WRpt | 1055 | 1092951000119106 | Pneumonia due to Bordetella parapertussis |
| LowerRespiratoryTractInfection-WRpt | 1055 | 1240541000000107 | Upper respiratory tract infection caused by SARS-CoV-2 (severe acute respiratory syndrome coronavirus 2) |
| LowerRespiratoryTractInfection-WRpt | 1055 | 1240551000000105 | Pneumonia caused by SARS-CoV-2 (severe acute respiratory syndrome coronavirus 2) |
| LowerRespiratoryTractInfection-WRpt | 1055 | 10624991000119103 | Bronchopneumonia due to Achromobacter |
| LowerRespiratoryTractInfection-WRpt | 1055 | 10625031000119102 | Bronchopneumonia due to anaerobic bacteria |
| LowerRespiratoryTractInfection-WRpt | 1055 | 10625071000119104 | Bronchopneumonia due to bacteria |
| LowerRespiratoryTractInfection-WRpt | 1055 | 10625111000119106 | Bronchopneumonia due to Escherichia coli |
| LowerRespiratoryTractInfection-WRpt | 1055 | 10625151000119107 | Bronchopneumonia due to Group A Streptococcus |
| LowerRespiratoryTractInfection-WRpt | 1055 | 10625191000119102 | Bronchopneumonia due to Group B Streptococcus |
| LowerRespiratoryTractInfection-WRpt | 1055 | 10625231000119106 | Bronchopneumonia due to Haemophilus influenzae |
| LowerRespiratoryTractInfection-WRpt | 1055 | 10625271000119109 | Bronchopneumonia due to Human metapneumovirus |
| LowerRespiratoryTractInfection-WRpt | 1055 | 10625311000119109 | Bronchopneumonia due to Klebsiella pneumoniae |
| LowerRespiratoryTractInfection-WRpt | 1055 | 10625351000119105 | Bronchopneumonia due to methicillin resistant Staphylococcus aureus |
| LowerRespiratoryTractInfection-WRpt | 1055 | 10625391000119100 | Bronchopneumonia due to methicillin susceptible Staphylococcus aureus |
| LowerRespiratoryTractInfection-WRpt | 1055 | 10625431000119105 | Bronchopneumonia due to Mycoplasma pneumoniae |
| LowerRespiratoryTractInfection-WRpt | 1055 | 10625471000119108 | Bronchopneumonia due to Proteus mirabilis |
| LowerRespiratoryTractInfection-WRpt | 1055 | 10625511000119104 | Bronchopneumonia due to Pseudomonas |
| LowerRespiratoryTractInfection-WRpt | 1055 | 10625551000119103 | Bronchopneumonia due to respiratory syncytial virus |
| LowerRespiratoryTractInfection-WRpt | 1055 | 10625591000119108 | Bronchopneumonia due to Staphylococcus |
| LowerRespiratoryTractInfection-WRpt | 1055 | 10625631000119108 | Bronchopneumonia due to Staphylococcus aureus |
| LowerRespiratoryTractInfection-WRpt | 1055 | 10625671000119106 | Bronchopneumonia due to Streptococcus |
| LowerRespiratoryTractInfection-WRpt | 1055 | 10625711000119105 | Bronchopneumonia due to Streptococcus pneumoniae |
| LowerRespiratoryTractInfection-WRpt | 1055 | 10625751000119106 | Bronchopneumonia due to virus |
| LowerRespiratoryTractInfection-WRpt | 1055 | 10629151000119105 | Acute bronchitis caused by coxsackievirus |
| LowerRespiratoryTractInfection-WRpt | 1055 | 10629191000119100 | Bronchiolitis caused by influenza virus |
| LowerRespiratoryTractInfection-WRpt | 1055 | 138389411000119105 | Acute bronchitis caused by SARS-CoV-2 (severe acute respiratory syndrome coronavirus 2) |
| LowerRespiratoryTractInfection-WRpt | 1055 | 880529761000119102 | Lower respiratory infection caused by SARS-CoV-2 (severe acute respiratory syndrome coronavirus 2) |
| LowerRespiratoryTractInfection-WRpt | 1055 | 882784691000119100 | Pneumonia caused by SARS-CoV-2 (severe acute respiratory syndrome coronavirus 2) |
| SeenInHospitalCasualty | 7183 | 4525004 | Emergency department patient visit |
| SeenInHospitalCasualty | 7183 | 11545006 | Emergency room admission, dead on arrival (DOA) |
| SeenInHospitalCasualty | 7183 | 12586001 | Physician direction of emergency medical systems |
| SeenInHospitalCasualty | 7183 | 50331008 | Emergency room admission, followed by release |
| SeenInHospitalCasualty | 7183 | 50849002 | Emergency room admission |
| SeenInHospitalCasualty | 7183 | 75004002 | Emergency room admission, died in emergency room |
| SeenInHospitalCasualty | 7183 | 183497001 | Non-urgent trauma admission |
| SeenInHospitalCasualty | 7183 | 185210004 | Seen in hospital casualty |
| SeenInHospitalCasualty | 7183 | 305226003 | Admission by Accident and Emergency doctor |
| SeenInHospitalCasualty | 7183 | 305451000 | Under care of Accident and Emergency doctor |
| SeenInHospitalCasualty | 7183 | 305633005 | Seen by Accident and Emergency doctor |
| SeenInHospitalCasualty | 7183 | 305925007 | Referral by Accident and Emergency doctor |
| SeenInHospitalCasualty | 7183 | 306390007 | Discharge by Accident and Emergency doctor |
| SeenInHospitalCasualty | 7183 | 306563004 | Discharge from Accident and Emergency service |
| SeenInHospitalCasualty | 7183 | 397721007 | Referral by Accident and Emergency |
| SeenInHospitalCasualty | 7183 | 413845009 | Chronic obstructive pulmonary disease accident and emergency attendance since last visit |
| SeenInHospitalCasualty | 7183 | 417119002 | Discharged from accident and emergency |
| SeenInHospitalCasualty | 7183 | 92291000000100 | Referral by accident and emergency |
| SeenInHospitalCasualty | 7183 | 169631000000104 | Chronic obstructive pulmonary disease accident and emergency attendance since last visit |
| SeenInHospitalCasualty | 7183 | 174211000000102 | Chronic obstructive pulmonary disease accident and emergency attendance since last visit |
| SeenInHospitalCasualty | 7183 | 188161000000105 | Discharged from accident and emergency |
| SeenInHospitalCasualty | 7183 | 191661000000106 | Discharged from accident and emergency |
| SeenInHospitalCasualty | 7183 | 353401000000109 | Seen in eye casualty department |
| SeenInHospitalCasualty | 7183 | 353411000000106 | Seen in eye casualty department |
| SeenInHospitalCasualty | 7183 | 353421000000100 | Seen in eye casualty department |
| SeenInHospitalCasualty | 7183 | 507291000000100 | Self-referral to accident and emergency department |
| SeenInHospitalCasualty | 7183 | 510051000000101 | Self-referral to accident and emergency department |
| SeenInHospitalCasualty | 7183 | 510061000000103 | Self-referral to accident and emergency department |
| SeenInHospitalCasualty | 7183 | 780781000000108 | Admission by accident and emergency doctor |
| SeenInHospitalCasualty | 7183 | 780831000000103 | Admission to accident and emergency department |
| SeenInHospitalCasualty | 7183 | 812481000000104 | Dead on arrival in accident and emergency department |
| SeenInHospitalCasualty | 7183 | 812491000000102 | Died in accident and emergency department |
| SeenInHospitalCasualty | 7183 | 826931000000104 | Frequent attender of accident and emergency department |
| SeenInHospitalCasualty | 7183 | 826941000000108 | Frequent attendance of accident and emergency |
| SeenInHospitalCasualty | 7183 | 831631000000103 | Referral by accident and emergency |
| SeenInHospitalCasualty | 7183 | 963261000000108 | Emergency department attendance related to personal alcohol consumption |
| SeenInHospitalCasualty | 7183 | 963271000000101 | Emergency department attendance related to personal alcohol consumption |
| SeenInHospitalCasualty | 7183 | 980491000000106 | Removed from Accident and Emergency department by force |
| SeenInHospitalCasualty | 7183 | 989501000000106 | Discharge from Accident and Emergency service with advice for follow up treatment by general practitioner |
| SeenInHospitalCasualty | 7183 | 989531000000100 | Left Accident and Emergency department having refused treatment |
| SeenInHospitalCasualty | 7183 | 1066331000000109 | Emergency department discharge to emergency department short stay ward |
| SeenInHospitalCasualty | 7183 | 1066341000000100 | Emergency department discharge to ambulatory emergency care service |
| SeenInHospitalCasualty | 7183 | 1066361000000104 | Emergency department discharge to high dependency unit |
| SeenInHospitalCasualty | 7183 | 1066371000000106 | Emergency department discharge to coronary care unit |
| SeenInHospitalCasualty | 7183 | 1066381000000108 | Emergency department discharge to special care baby unit |
| SeenInHospitalCasualty | 7183 | 1066391000000105 | Emergency department discharge to intensive care unit |
| SeenInHospitalCasualty | 7183 | 1066401000000108 | Emergency department discharge to neonatal intensive care unit |
| SeenInHospitalCasualty | 7183 | 1077011000000106 | Streamed from emergency department following initial assessment |
| SeenInHospitalCasualty | 7183 | 1077021000000100 | Streamed from emergency department to general practitioner following initial assessment |
| SeenInHospitalCasualty | 7183 | 1077031000000103 | Streamed from emergency department to urgent care service following initial assessment |
| SeenInHospitalCasualty | 7183 | 1077041000000107 | Streamed from emergency department to mental health service following initial assessment |
| SeenInHospitalCasualty | 7183 | 1077051000000105 | Streamed from emergency department to dental service following initial assessment |
| SeenInHospitalCasualty | 7183 | 1077061000000108 | Streamed from emergency department to ophthalmology service following initial assessment |
| SeenInHospitalCasualty | 7183 | 1077071000000101 | Streamed from emergency department to pharmacy service following initial assessment |
| SeenInHospitalCasualty | 7183 | 1077081000000104 | Streamed from emergency department to ambulatory emergency care service following initial assessment |
| SeenInHospitalCasualty | 7183 | 1077091000000102 | Streamed from emergency department to falls service following initial assessment |
| SeenInHospitalCasualty | 7183 | 1077101000000105 | Streamed from emergency department to frailty service following initial assessment |
| SeenInHospitalCasualty | 7183 | 1082421000000101 | Emergency hospital admission to accident and emergency service |
| SeenInHospitalCasualty | 7183 | 1324201000000109 | Streamed from emergency department to inpatient unit following initial assessment |
| SeenInHospitalCasualty | 7183 | 1656561000006119 | Seen in eye casualty |
| SeenInHospitalCasualty | 7183 | 1779101000006110 | Seen in gynaecology accident and emergency department |
| SeenInHospitalCasualty | 7183 | 1779111000006113 | Seen in general medicine accident and emergency department |
| SeenInHospitalCasualty | 7183 | 1779121000006117 | Seen in ENT accident and emergency department |
| SeenInHospitalCasualty | 7183 | 1779131000006119 | Seen in paediatric accident and emergency department |
| SeenInHospitalCasualty | 7183 | 1779141000006112 | Seen in surgical accident and emergency department |
| SeenInHospitalCasualty | 7183 | 1779151000006114 | Seen in orthopaedic accident and emergency department |
| UpperRespiratoryInfection-WRpt | 1007 | 140004 | Chronic pharyngitis |
| UpperRespiratoryInfection-WRpt | 1007 | 222008 | Acute epiglottitis with obstruction |
| UpperRespiratoryInfection-WRpt | 1007 | 297009 | Acute myringitis |
| UpperRespiratoryInfection-WRpt | 1007 | 652005 | Gangrenous tonsillitis |
| UpperRespiratoryInfection-WRpt | 1007 | 1532007 | Viral pharyngitis |
| UpperRespiratoryInfection-WRpt | 1007 | 1980003 | Seromucinous otitis media |
| UpperRespiratoryInfection-WRpt | 1007 | 2365002 | Chronic granular pharyngitis |
| UpperRespiratoryInfection-WRpt | 1007 | 3110003 | Acute otitis media |
| UpperRespiratoryInfection-WRpt | 1007 | 4225003 | Tuberculosis of nose |
| UpperRespiratoryInfection-WRpt | 1007 | 5028002 | Acute pansinusitis |
| UpperRespiratoryInfection-WRpt | 1007 | 6142004 | Influenza |
| UpperRespiratoryInfection-WRpt | 1007 | 6655004 | Acute laryngitis |
| UpperRespiratoryInfection-WRpt | 1007 | 7457009 | Chronic tracheitis |
| UpperRespiratoryInfection-WRpt | 1007 | 8304007 | Acute seromucinous otitis media |
| UpperRespiratoryInfection-WRpt | 1007 | 8326008 | Acute allergic mucoid otitis media |
| UpperRespiratoryInfection-WRpt | 1007 | 8442000 | Purulent rhinitis |
| UpperRespiratoryInfection-WRpt | 1007 | 8519009 | Acute tracheitis with obstruction |
| UpperRespiratoryInfection-WRpt | 1007 | 9312005 | Necrotic rhinitis |
| UpperRespiratoryInfection-WRpt | 1007 | 10345003 | Primary syphilis of tonsils |
| UpperRespiratoryInfection-WRpt | 1007 | 10351008 | Suppurative tonsillitis |
| UpperRespiratoryInfection-WRpt | 1007 | 10809006 | Parainfluenza virus laryngotracheitis |
| UpperRespiratoryInfection-WRpt | 1007 | 11134001 | Acute suppuration of sphenoidal sinus |
| UpperRespiratoryInfection-WRpt | 1007 | 11316005 | Granuloma of vocal cords |
| UpperRespiratoryInfection-WRpt | 1007 | 11461005 | Staphylococcal tonsillitis |
| UpperRespiratoryInfection-WRpt | 1007 | 13177009 | Cellulitis of nasopharynx |
| UpperRespiratoryInfection-WRpt | 1007 | 13266007 | Sphenoidal sinusitis |
| UpperRespiratoryInfection-WRpt | 1007 | 13420004 | Post measles otitis media |
| UpperRespiratoryInfection-WRpt | 1007 | 13617004 | Tracheobronchitis |
| UpperRespiratoryInfection-WRpt | 1007 | 13933003 | Feline viral rhinotracheitis |
| UpperRespiratoryInfection-WRpt | 1007 | 14310000 | Purulent nasal discharge |
| UpperRespiratoryInfection-WRpt | 1007 | 14465002 | Ulcerative tonsillitis |
| UpperRespiratoryInfection-WRpt | 1007 | 14948001 | Acute suppurative otitis media without spontaneous rupture of ear drum |
| UpperRespiratoryInfection-WRpt | 1007 | 14969004 | Catarrhal laryngitis |
| UpperRespiratoryInfection-WRpt | 1007 | 15033003 | Peritonsillar abscess |
| UpperRespiratoryInfection-WRpt | 1007 | 15682004 | Anterior nasal diphtheria |
| UpperRespiratoryInfection-WRpt | 1007 | 15805002 | Acute sinusitis |
| UpperRespiratoryInfection-WRpt | 1007 | 16036000 | Acute empyema of frontal sinus |
| UpperRespiratoryInfection-WRpt | 1007 | 16664009 | Malignant otitis media |
| UpperRespiratoryInfection-WRpt | 1007 | 17357005 | Acute suppuration of frontal sinus |
| UpperRespiratoryInfection-WRpt | 1007 | 17741008 | Acute tonsillitis |
| UpperRespiratoryInfection-WRpt | 1007 | 17866004 | Acute allergic sanguinous otitis media |
| UpperRespiratoryInfection-WRpt | 1007 | 18643000 | Ethmoidal sinusitis |
| UpperRespiratoryInfection-WRpt | 1007 | 19021002 | Haemophilus influenzae otitis media |
| UpperRespiratoryInfection-WRpt | 1007 | 19399000 | Acute exudative otitis media |
| UpperRespiratoryInfection-WRpt | 1007 | 20906004 | Mycosis leptothrica |
| UpperRespiratoryInfection-WRpt | 1007 | 21060003 | Acute bacterial epiglottitis |
| UpperRespiratoryInfection-WRpt | 1007 | 23166004 | Cellulitis of pharynx |
| UpperRespiratoryInfection-WRpt | 1007 | 23884004 | Acute suppuration of maxillary sinus |
| UpperRespiratoryInfection-WRpt | 1007 | 24078009 | Gangosa of yaws |
| UpperRespiratoryInfection-WRpt | 1007 | 24347001 | Cellulitis of vocal cords |
| UpperRespiratoryInfection-WRpt | 1007 | 24662006 | Influenza due to Influenza B virus |
| UpperRespiratoryInfection-WRpt | 1007 | 25764005 | Acute abscess of frontal sinus |
| UpperRespiratoryInfection-WRpt | 1007 | 25831001 | Tuberculosis of nasal septum |
| UpperRespiratoryInfection-WRpt | 1007 | 26650005 | Acute tracheitis |
| UpperRespiratoryInfection-WRpt | 1007 | 26808007 | Polypoid sinus degeneration |
| UpperRespiratoryInfection-WRpt | 1007 | 27278006 | Acute empyema of sphenoidal sinus |
| UpperRespiratoryInfection-WRpt | 1007 | 27836007 | Pertussis |
| UpperRespiratoryInfection-WRpt | 1007 | 27878001 | Follicular tonsillitis |
| UpperRespiratoryInfection-WRpt | 1007 | 28709001 | Cellulitis of larynx |
| UpperRespiratoryInfection-WRpt | 1007 | 29608009 | Acute epiglottitis |
| UpperRespiratoryInfection-WRpt | 1007 | 29951006 | Chronic laryngitis |
| UpperRespiratoryInfection-WRpt | 1007 | 30239003 | Acute abscess of sphenoidal sinus |
| UpperRespiratoryInfection-WRpt | 1007 | 31309002 | Respiratory syncytial virus pharyngitis |
| UpperRespiratoryInfection-WRpt | 1007 | 32179007 | Furuncle of nasal septum |
| UpperRespiratoryInfection-WRpt | 1007 | 32904004 | Pneumococcal laryngitis |
| UpperRespiratoryInfection-WRpt | 1007 | 33261009 | Abscess of tonsil |
| UpperRespiratoryInfection-WRpt | 1007 | 33924006 | Abscess of larynx |
| UpperRespiratoryInfection-WRpt | 1007 | 34790005 | Chronic tubotympanic disease with anterior perforation of ear drum |
| UpperRespiratoryInfection-WRpt | 1007 | 35168006 | Acute empyema of ethmoidal sinus |
| UpperRespiratoryInfection-WRpt | 1007 | 35183001 | Acute transudative otitis media |
| UpperRespiratoryInfection-WRpt | 1007 | 35301006 | Acute tracheobronchitis |
| UpperRespiratoryInfection-WRpt | 1007 | 35377009 | Parainfluenza virus laryngotracheobronchitis |
| UpperRespiratoryInfection-WRpt | 1007 | 35923002 | Chronic maxillary sinusitis |
| UpperRespiratoryInfection-WRpt | 1007 | 36971009 | Sinusitis |
| UpperRespiratoryInfection-WRpt | 1007 | 37426002 | Ulcerative laryngitis |
| UpperRespiratoryInfection-WRpt | 1007 | 37948003 | Acute laryngotracheitis without obstruction |
| UpperRespiratoryInfection-WRpt | 1007 | 38394007 | Chronic purulent otitis media |
| UpperRespiratoryInfection-WRpt | 1007 | 38961000 | Chronic sphenoidal sinusitis |
| UpperRespiratoryInfection-WRpt | 1007 | 39271004 | Ulcerative pharyngitis |
| UpperRespiratoryInfection-WRpt | 1007 | 39288006 | Purulent otitis media |
| UpperRespiratoryInfection-WRpt | 1007 | 40055000 | Chronic sinusitis |
| UpperRespiratoryInfection-WRpt | 1007 | 40766000 | Enteroviral lymphonodular pharyngitis |
| UpperRespiratoryInfection-WRpt | 1007 | 41048006 | Haemophilus influenzae laryngitis |
| UpperRespiratoryInfection-WRpt | 1007 | 41269000 | Influenzal bronchopneumonia |
| UpperRespiratoryInfection-WRpt | 1007 | 41582007 | Streptococcal tonsillitis |
| UpperRespiratoryInfection-WRpt | 1007 | 41954005 | Chronic atticoantral suppurative otitis media |
| UpperRespiratoryInfection-WRpt | 1007 | 42402006 | Kartagener syndrome |
| UpperRespiratoryInfection-WRpt | 1007 | 42964004 | Influenza with pneumonia |
| UpperRespiratoryInfection-WRpt | 1007 | 43692000 | Influenzal acute upper respiratory infection |
| UpperRespiratoryInfection-WRpt | 1007 | 43878008 | Streptococcal sore throat |
| UpperRespiratoryInfection-WRpt | 1007 | 45629003 | Abscess of nasopharynx |
| UpperRespiratoryInfection-WRpt | 1007 | 45913009 | Laryngitis |
| UpperRespiratoryInfection-WRpt | 1007 | 46171006 | Influenza due to Influenza virus, type A, porcine |
| UpperRespiratoryInfection-WRpt | 1007 | 47841006 | Chronic nasopharyngitis |
| UpperRespiratoryInfection-WRpt | 1007 | 49908003 | Acute epiglottitis without obstruction |
| UpperRespiratoryInfection-WRpt | 1007 | 50211006 | Catarrhal tracheitis |
| UpperRespiratoryInfection-WRpt | 1007 | 50215002 | Laryngeal diphtheria |
| UpperRespiratoryInfection-WRpt | 1007 | 51152000 | Abscess of vocal cords |
| UpperRespiratoryInfection-WRpt | 1007 | 51209006 | Viral tonsillitis |
| UpperRespiratoryInfection-WRpt | 1007 | 51476001 | Nasopharyngitis |
| UpperRespiratoryInfection-WRpt | 1007 | 51960003 | Secondary syphilis of pharynx |
| UpperRespiratoryInfection-WRpt | 1007 | 52353000 | Acute mucoid otitis media |
| UpperRespiratoryInfection-WRpt | 1007 | 52571006 | Chronic tracheobronchitis |
| UpperRespiratoryInfection-WRpt | 1007 | 52721006 | Tuberculosis of nasal sinus |
| UpperRespiratoryInfection-WRpt | 1007 | 54150009 | Upper respiratory infection |
| UpperRespiratoryInfection-WRpt | 1007 | 54383008 | Inclusion body rhinitis of swine |
| UpperRespiratoryInfection-WRpt | 1007 | 54398005 | Acute upper respiratory infection |
| UpperRespiratoryInfection-WRpt | 1007 | 55130001 | Laryngotracheitis |
| UpperRespiratoryInfection-WRpt | 1007 | 55355000 | Acute laryngopharyngitis |
| UpperRespiratoryInfection-WRpt | 1007 | 55419007 | Tuberculosis of glottis |
| UpperRespiratoryInfection-WRpt | 1007 | 55604004 | Avian influenza |
| UpperRespiratoryInfection-WRpt | 1007 | 56498009 | Tuberculosis of nasopharynx |
| UpperRespiratoryInfection-WRpt | 1007 | 57713008 | Chorditis |
| UpperRespiratoryInfection-WRpt | 1007 | 58031004 | Suppurative pharyngitis |
| UpperRespiratoryInfection-WRpt | 1007 | 58194007 | Chronic seromucinous otitis media |
| UpperRespiratoryInfection-WRpt | 1007 | 58576005 | Haemophilus influenzae epiglottitis |
| UpperRespiratoryInfection-WRpt | 1007 | 58763001 | Acute empyema of nasal sinus |
| UpperRespiratoryInfection-WRpt | 1007 | 59221008 | Parainfluenza virus rhinopharyngitis |
| UpperRespiratoryInfection-WRpt | 1007 | 59275002 | Acute allergic serous otitis media |
| UpperRespiratoryInfection-WRpt | 1007 | 59707005 | Abscess of pharynx |
| UpperRespiratoryInfection-WRpt | 1007 | 59967003 | Acute laryngotracheitis with obstruction |
| UpperRespiratoryInfection-WRpt | 1007 | 60130002 | Chronic frontal sinusitis |
| UpperRespiratoryInfection-WRpt | 1007 | 61437000 | Furuncle of nose |
| UpperRespiratoryInfection-WRpt | 1007 | 61700007 | Influenza with non-respiratory manifestation |
| UpperRespiratoryInfection-WRpt | 1007 | 61711004 | Acute abscess of ethmoidal sinus |
| UpperRespiratoryInfection-WRpt | 1007 | 62877002 | Infectious canine tracheobronchitis |
| UpperRespiratoryInfection-WRpt | 1007 | 62994001 | Tracheitis |
| UpperRespiratoryInfection-WRpt | 1007 | 63039003 | Influenza with respiratory manifestation other than pneumonia |
| UpperRespiratoryInfection-WRpt | 1007 | 63140003 | Acute suppuration of ethmoidal sinus |
| UpperRespiratoryInfection-WRpt | 1007 | 63866002 | Atrophic pharyngitis |
| UpperRespiratoryInfection-WRpt | 1007 | 64369009 | Acute tracheitis without obstruction |
| UpperRespiratoryInfection-WRpt | 1007 | 64375000 | Acute laryngotracheitis |
| UpperRespiratoryInfection-WRpt | 1007 | 64531003 | Nasal discharge |
| UpperRespiratoryInfection-WRpt | 1007 | 64611009 | Catarrhal nasal discharge |
| UpperRespiratoryInfection-WRpt | 1007 | 65363002 | Otitis media |
| UpperRespiratoryInfection-WRpt | 1007 | 66011008 | Viral tracheitis |
| UpperRespiratoryInfection-WRpt | 1007 | 67832005 | Acute ethmoidal sinusitis |
| UpperRespiratoryInfection-WRpt | 1007 | 68272006 | Acute maxillary sinusitis |
| UpperRespiratoryInfection-WRpt | 1007 | 68686002 | Infectious bovine rhinotracheitis |
| UpperRespiratoryInfection-WRpt | 1007 | 70020005 | Adenoiditis |
| UpperRespiratoryInfection-WRpt | 1007 | 70341005 | Tuberculous laryngitis |
| UpperRespiratoryInfection-WRpt | 1007 | 70385007 | Adenoviral pharyngoconjunctivitis |
| UpperRespiratoryInfection-WRpt | 1007 | 70976000 | Viral epiglottitis |
| UpperRespiratoryInfection-WRpt | 1007 | 71186008 | Croup |
| UpperRespiratoryInfection-WRpt | 1007 | 71255007 | Adenoviral laryngotracheobronchitis |
| UpperRespiratoryInfection-WRpt | 1007 | 72189003 | Haemorrhagic nasal discharge |
| UpperRespiratoryInfection-WRpt | 1007 | 72204002 | Respiratory syncytial virus laryngotracheobronchitis |
| UpperRespiratoryInfection-WRpt | 1007 | 72211003 | Laryngeal granuloma |
| UpperRespiratoryInfection-WRpt | 1007 | 72409005 | Rhinoscleroma |
| UpperRespiratoryInfection-WRpt | 1007 | 72430001 | Gangrenous pharyngitis |
| UpperRespiratoryInfection-WRpt | 1007 | 73237007 | Chronic ethmoidal sinusitis |
| UpperRespiratoryInfection-WRpt | 1007 | 73414003 | Haemophilus influenzae laryngotracheobronchitis |
| UpperRespiratoryInfection-WRpt | 1007 | 74372003 | Gonorrhoea of pharynx |
| UpperRespiratoryInfection-WRpt | 1007 | 74644004 | Influenza with encephalopathy |
| UpperRespiratoryInfection-WRpt | 1007 | 75498004 | Acute bacterial sinusitis |
| UpperRespiratoryInfection-WRpt | 1007 | 75589004 | Nasopharyngeal diphtheria |
| UpperRespiratoryInfection-WRpt | 1007 | 75803007 | Posterior rhinorrhoea |
| UpperRespiratoryInfection-WRpt | 1007 | 76651006 | Pneumococcal pharyngitis |
| UpperRespiratoryInfection-WRpt | 1007 | 76653009 | Acute empyema of maxillary sinus |
| UpperRespiratoryInfection-WRpt | 1007 | 77478005 | Acute sanguinous otitis media |
| UpperRespiratoryInfection-WRpt | 1007 | 77668003 | Isolated tracheal tuberculosis |
| UpperRespiratoryInfection-WRpt | 1007 | 77919000 | Acute sphenoidal sinusitis |
| UpperRespiratoryInfection-WRpt | 1007 | 78337007 | Acute upper respiratory infection of multiple sites |
| UpperRespiratoryInfection-WRpt | 1007 | 78430008 | Adenoviral pharyngitis |
| UpperRespiratoryInfection-WRpt | 1007 | 78431007 | Influenza due to Influenza virus, type A, human |
| UpperRespiratoryInfection-WRpt | 1007 | 78737005 | Frontal sinusitis |
| UpperRespiratoryInfection-WRpt | 1007 | 78911000 | Parainfluenza virus pharyngitis |
| UpperRespiratoryInfection-WRpt | 1007 | 80327007 | Serous otitis media |
| UpperRespiratoryInfection-WRpt | 1007 | 80384002 | Epiglottitis |
| UpperRespiratoryInfection-WRpt | 1007 | 80600003 | Acute suppuration of nasal sinus |
| UpperRespiratoryInfection-WRpt | 1007 | 81339006 | Secondary syphilis of tonsil |
| UpperRespiratoryInfection-WRpt | 1007 | 81524006 | Influenza due to Influenza C virus |
| UpperRespiratoryInfection-WRpt | 1007 | 81564005 | Chronic serous otitis media |
| UpperRespiratoryInfection-WRpt | 1007 | 82228008 | Staphylococcal pharyngitis |
| UpperRespiratoryInfection-WRpt | 1007 | 82272006 | Common cold |
| UpperRespiratoryInfection-WRpt | 1007 | 82454002 | Carbuncle of nasal septum |
| UpperRespiratoryInfection-WRpt | 1007 | 82670009 | Whooping cough due to organism other than Bordetella pertussis |
| UpperRespiratoryInfection-WRpt | 1007 | 82690000 | Suppurative laryngitis |
| UpperRespiratoryInfection-WRpt | 1007 | 83271005 | Chronic laryngotracheitis |
| UpperRespiratoryInfection-WRpt | 1007 | 83492008 | Congenital syphilitic coryza |
| UpperRespiratoryInfection-WRpt | 1007 | 84037004 | Swine influenza |
| UpperRespiratoryInfection-WRpt | 1007 | 85083002 | Streptococcal laryngitis |
| UpperRespiratoryInfection-WRpt | 1007 | 85638002 | Cerebrospinal fluid rhinorrhoea |
| UpperRespiratoryInfection-WRpt | 1007 | 85832003 | Parainfluenza virus laryngitis |
| UpperRespiratoryInfection-WRpt | 1007 | 85915003 | Laryngotracheobronchitis |
| UpperRespiratoryInfection-WRpt | 1007 | 85940005 | Tuberculous otitis media |
| UpperRespiratoryInfection-WRpt | 1007 | 86279000 | Acute suppurative otitis media with spontaneous rupture of ear drum |
| UpperRespiratoryInfection-WRpt | 1007 | 86359006 | Subacute transudative otitis media |
| UpperRespiratoryInfection-WRpt | 1007 | 86850004 | Serosanguineous chronic otitis media |
| UpperRespiratoryInfection-WRpt | 1007 | 87665008 | Chronic tubotympanic suppurative otitis media |
| UpperRespiratoryInfection-WRpt | 1007 | 88348008 | Maxillary sinusitis |
| UpperRespiratoryInfection-WRpt | 1007 | 88850006 | Chronic pansinusitis |
| UpperRespiratoryInfection-WRpt | 1007 | 89194009 | Acute abscess of maxillary sinus |
| UpperRespiratoryInfection-WRpt | 1007 | 90176007 | Tonsillitis |
| UpperRespiratoryInfection-WRpt | 1007 | 90347002 | Infectious avian laryngotracheitis |
| UpperRespiratoryInfection-WRpt | 1007 | 90979004 | Chronic tonsillitis |
| UpperRespiratoryInfection-WRpt | 1007 | 91038008 | Acute frontal sinusitis |
| UpperRespiratoryInfection-WRpt | 1007 | 95885008 | Mycoplasmal pharyngitis |
| UpperRespiratoryInfection-WRpt | 1007 | 95886009 | Mycoplasmal tracheobronchitis |
| UpperRespiratoryInfection-WRpt | 1007 | 102453009 | Peritonsillar cellulitis |
| UpperRespiratoryInfection-WRpt | 1007 | 111274000 | Acute abscess of nasal sinus |
| UpperRespiratoryInfection-WRpt | 1007 | 111275004 | Abscess of nasal septum |
| UpperRespiratoryInfection-WRpt | 1007 | 111816002 | Pneumococcal tonsillitis |
| UpperRespiratoryInfection-WRpt | 1007 | 126664009 | Exudative pharyngitis |
| UpperRespiratoryInfection-WRpt | 1007 | 126665005 | Oropharyngeal mucositis |
| UpperRespiratoryInfection-WRpt | 1007 | 139168000 | Influenza-like symptoms |
| UpperRespiratoryInfection-WRpt | 1007 | 154341002 | Postmeasles otitis media |
| UpperRespiratoryInfection-WRpt | 1007 | 155222006 | Otitis media NOS |
| UpperRespiratoryInfection-WRpt | 1007 | 155505008 | Acute laryngitis and tracheitis |
| UpperRespiratoryInfection-WRpt | 1007 | 161913008 | Influenza-like symptoms |
| UpperRespiratoryInfection-WRpt | 1007 | 164189000 | O/E - nasal discharge NOS |
| UpperRespiratoryInfection-WRpt | 1007 | 186353006 | Whooping cough - other specified organism |
| UpperRespiratoryInfection-WRpt | 1007 | 186355004 | Other whooping cough NOS |
| UpperRespiratoryInfection-WRpt | 1007 | 186356003 | Whooping cough NOS |
| UpperRespiratoryInfection-WRpt | 1007 | 186361001 | Streptococcal sore throat NOS |
| UpperRespiratoryInfection-WRpt | 1007 | 186560001 | Postmeasles otitis media |
| UpperRespiratoryInfection-WRpt | 1007 | 186659004 | Herpangina |
| UpperRespiratoryInfection-WRpt | 1007 | 186675001 | Viral pharyngoconjunctivitis |
| UpperRespiratoryInfection-WRpt | 1007 | 186963008 | Vincent's angina |
| UpperRespiratoryInfection-WRpt | 1007 | 187084006 | Tonsillar aspergillosis |
| UpperRespiratoryInfection-WRpt | 1007 | 187100003 | Rhinocerebral mucormycosis |
| UpperRespiratoryInfection-WRpt | 1007 | 187222000 | Nasopharyngeal myiasis |
| UpperRespiratoryInfection-WRpt | 1007 | 187324001 | [X]Whooping cough due to other Bordetella species |
| UpperRespiratoryInfection-WRpt | 1007 | 187325000 | [X]Whooping cough, unspecified |
| UpperRespiratoryInfection-WRpt | 1007 | 194202008 | Infective otitis externa due to erysipelas |
| UpperRespiratoryInfection-WRpt | 1007 | 194203003 | Infective otitis externa due to impetigo |
| UpperRespiratoryInfection-WRpt | 1007 | 194240006 | Acute non-suppurative serous otitis media |
| UpperRespiratoryInfection-WRpt | 1007 | 194244002 | Acute non-suppurative otitis media NOS |
| UpperRespiratoryInfection-WRpt | 1007 | 194248004 | Bilateral chronic serous otitis |
| UpperRespiratoryInfection-WRpt | 1007 | 194249007 | Unilateral chronic serous otitis |
| UpperRespiratoryInfection-WRpt | 1007 | 194250007 | Chronic serous otitis media NOS |
| UpperRespiratoryInfection-WRpt | 1007 | 194252004 | Glue ear, unspecified |
| UpperRespiratoryInfection-WRpt | 1007 | 194254003 | Chronic mucoid otitis media NOS |
| UpperRespiratoryInfection-WRpt | 1007 | 194256001 | Chronic otitis media with effusion, other |
| UpperRespiratoryInfection-WRpt | 1007 | 194260003 | Unspecified non-suppurative otitis media |
| UpperRespiratoryInfection-WRpt | 1007 | 194261004 | Allergic otitis media NOS |
| UpperRespiratoryInfection-WRpt | 1007 | 194262006 | Serous otitis media NOS |
| UpperRespiratoryInfection-WRpt | 1007 | 194263001 | Catarrhal otitis media NOS |
| UpperRespiratoryInfection-WRpt | 1007 | 194264007 | Mucoid otitis media NOS |
| UpperRespiratoryInfection-WRpt | 1007 | 194265008 | Non-suppurative otitis media NOS |
| UpperRespiratoryInfection-WRpt | 1007 | 194276007 | Chronic otitis media with effusion, unspecified |
| UpperRespiratoryInfection-WRpt | 1007 | 194281003 | Acute suppurative otitis media |
| UpperRespiratoryInfection-WRpt | 1007 | 194282005 | Acute suppurative otitis media due to another disease |
| UpperRespiratoryInfection-WRpt | 1007 | 194283000 | Acute suppurative otitis media NOS |
| UpperRespiratoryInfection-WRpt | 1007 | 194284006 | Chronic suppurative otitis media NOS |
| UpperRespiratoryInfection-WRpt | 1007 | 194286008 | Bilateral suppurative otitis media |
| UpperRespiratoryInfection-WRpt | 1007 | 194287004 | Recurrent acute otitis media |
| UpperRespiratoryInfection-WRpt | 1007 | 194288009 | Acute left otitis media |
| UpperRespiratoryInfection-WRpt | 1007 | 194289001 | Acute right otitis media |
| UpperRespiratoryInfection-WRpt | 1007 | 194290005 | Acute bilateral otitis media |
| UpperRespiratoryInfection-WRpt | 1007 | 194311006 | Acute myringitis without otitis media |
| UpperRespiratoryInfection-WRpt | 1007 | 194312004 | Unspecified acute tympanitis |
| UpperRespiratoryInfection-WRpt | 1007 | 194313009 | Acute myringitis NOS |
| UpperRespiratoryInfection-WRpt | 1007 | 194675002 | [X]Other acute non-suppurative otitis media |
| UpperRespiratoryInfection-WRpt | 1007 | 194677005 | [X]Other chronic suppurative otitis media |
| UpperRespiratoryInfection-WRpt | 1007 | 194680006 | [X]Otitis media in other diseases classified elsewhere |
| UpperRespiratoryInfection-WRpt | 1007 | 195651009 | Other acute sinusitis |
| UpperRespiratoryInfection-WRpt | 1007 | 195652002 | Other acute sinusitis NOS |
| UpperRespiratoryInfection-WRpt | 1007 | 195653007 | Acute sinusitis NOS |
| UpperRespiratoryInfection-WRpt | 1007 | 195655000 | Acute gangrenous pharyngitis |
| UpperRespiratoryInfection-WRpt | 1007 | 195656004 | Acute phlegmonous pharyngitis |
| UpperRespiratoryInfection-WRpt | 1007 | 195657008 | Acute ulcerative pharyngitis |
| UpperRespiratoryInfection-WRpt | 1007 | 195658003 | Acute bacterial pharyngitis |
| UpperRespiratoryInfection-WRpt | 1007 | 195659006 | Acute pneumococcal pharyngitis |
| UpperRespiratoryInfection-WRpt | 1007 | 195660001 | Acute staphylococcal pharyngitis |
| UpperRespiratoryInfection-WRpt | 1007 | 195661002 | Acute bacterial pharyngitis NOS |
| UpperRespiratoryInfection-WRpt | 1007 | 195662009 | Acute viral pharyngitis |
| UpperRespiratoryInfection-WRpt | 1007 | 195664005 | Acute pharyngitis NOS |
| UpperRespiratoryInfection-WRpt | 1007 | 195666007 | Acute erythematous tonsillitis |
| UpperRespiratoryInfection-WRpt | 1007 | 195667003 | Acute follicular tonsillitis |
| UpperRespiratoryInfection-WRpt | 1007 | 195668008 | Acute ulcerative tonsillitis |
| UpperRespiratoryInfection-WRpt | 1007 | 195669000 | Acute catarrhal tonsillitis |
| UpperRespiratoryInfection-WRpt | 1007 | 195670004 | Acute gangrenous tonsillitis |
| UpperRespiratoryInfection-WRpt | 1007 | 195671000 | Acute bacterial tonsillitis |
| UpperRespiratoryInfection-WRpt | 1007 | 195672007 | Acute pneumococcal tonsillitis |
| UpperRespiratoryInfection-WRpt | 1007 | 195673002 | Acute staphylococcal tonsillitis |
| UpperRespiratoryInfection-WRpt | 1007 | 195674008 | Acute bacterial tonsillitis NOS |
| UpperRespiratoryInfection-WRpt | 1007 | 195676005 | Acute viral tonsillitis |
| UpperRespiratoryInfection-WRpt | 1007 | 195677001 | Recurrent acute tonsillitis |
| UpperRespiratoryInfection-WRpt | 1007 | 195678006 | Acute tonsillitis NOS |
| UpperRespiratoryInfection-WRpt | 1007 | 195679003 | Acute laryngitis and tracheitis |
| UpperRespiratoryInfection-WRpt | 1007 | 195680000 | Acute oedematous laryngitis |
| UpperRespiratoryInfection-WRpt | 1007 | 195681001 | Acute ulcerative laryngitis |
| UpperRespiratoryInfection-WRpt | 1007 | 195682008 | Acute catarrhal laryngitis |
| UpperRespiratoryInfection-WRpt | 1007 | 195683003 | Acute phlegmonous laryngitis |
| UpperRespiratoryInfection-WRpt | 1007 | 195684009 | Acute laryngitis caused by Haemophilus influenzae |
| UpperRespiratoryInfection-WRpt | 1007 | 195685005 | Acute pneumococcal laryngitis |
| UpperRespiratoryInfection-WRpt | 1007 | 195686006 | Acute suppurative laryngitis |
| UpperRespiratoryInfection-WRpt | 1007 | 195688007 | Acute viral laryngitis unspecified |
| UpperRespiratoryInfection-WRpt | 1007 | 195689004 | Acute bacterial laryngitis unspecified |
| UpperRespiratoryInfection-WRpt | 1007 | 195690008 | Acute laryngitis NOS |
| UpperRespiratoryInfection-WRpt | 1007 | 195693005 | Acute tracheitis NOS |
| UpperRespiratoryInfection-WRpt | 1007 | 195697006 | Acute laryngotracheitis NOS |
| UpperRespiratoryInfection-WRpt | 1007 | 195701009 | Acute epiglottitis NOS |
| UpperRespiratoryInfection-WRpt | 1007 | 195703007 | Acute laryngitis and tracheitis NOS |
| UpperRespiratoryInfection-WRpt | 1007 | 195704001 | Other acute upper respiratory infections |
| UpperRespiratoryInfection-WRpt | 1007 | 195707008 | Tracheopharyngitis |
| UpperRespiratoryInfection-WRpt | 1007 | 195708003 | Recurrent upper respiratory tract infection |
| UpperRespiratoryInfection-WRpt | 1007 | 195709006 | Pharyngolaryngitis |
| UpperRespiratoryInfection-WRpt | 1007 | 195710001 | Other upper respiratory infections of multiple sites |
| UpperRespiratoryInfection-WRpt | 1007 | 195756009 | Woakes' ethmoiditis |
| UpperRespiratoryInfection-WRpt | 1007 | 195757000 | Polypoid sinus degeneration NOS |
| UpperRespiratoryInfection-WRpt | 1007 | 195770009 | Chronic infective rhinitis |
| UpperRespiratoryInfection-WRpt | 1007 | 195779005 | Pharyngitis keratosa |
| UpperRespiratoryInfection-WRpt | 1007 | 195780008 | Pharyngitis sicca |
| UpperRespiratoryInfection-WRpt | 1007 | 195782000 | Chronic follicular pharyngitis |
| UpperRespiratoryInfection-WRpt | 1007 | 195783005 | Chronic pharyngitis NOS |
| UpperRespiratoryInfection-WRpt | 1007 | 195784004 | Chronic pharyngitis and nasopharyngitis NOS |
| UpperRespiratoryInfection-WRpt | 1007 | 195788001 | Recurrent sinusitis |
| UpperRespiratoryInfection-WRpt | 1007 | 195789009 | Other chronic sinusitis |
| UpperRespiratoryInfection-WRpt | 1007 | 195790000 | Pansinusitis |
| UpperRespiratoryInfection-WRpt | 1007 | 195791001 | Other chronic sinusitis NOS |
| UpperRespiratoryInfection-WRpt | 1007 | 195792008 | Chronic sinusitis NOS |
| UpperRespiratoryInfection-WRpt | 1007 | 195798007 | Chronic adenotonsillitis |
| UpperRespiratoryInfection-WRpt | 1007 | 195803003 | Caseous tonsillitis |
| UpperRespiratoryInfection-WRpt | 1007 | 195804009 | Lingular tonsillitis |
| UpperRespiratoryInfection-WRpt | 1007 | 195810009 | Chronic laryngitis and laryngotracheitis |
| UpperRespiratoryInfection-WRpt | 1007 | 195811008 | Chronic simple laryngitis |
| UpperRespiratoryInfection-WRpt | 1007 | 195812001 | Chronic catarrhal laryngitis |
| UpperRespiratoryInfection-WRpt | 1007 | 195816003 | Chronic laryngitis NOS |
| UpperRespiratoryInfection-WRpt | 1007 | 195836002 | Pharynx or nasopharynx abscess |
| UpperRespiratoryInfection-WRpt | 1007 | 195853009 | Singers' chorditis |
| UpperRespiratoryInfection-WRpt | 1007 | 195854003 | Fibrinous chorditis |
| UpperRespiratoryInfection-WRpt | 1007 | 195855002 | Chorditis vocalis inferior |
| UpperRespiratoryInfection-WRpt | 1007 | 195878008 | Pneumonia and influenza |
| UpperRespiratoryInfection-WRpt | 1007 | 195920000 | Influenza with pneumonia, influenza virus identified |
| UpperRespiratoryInfection-WRpt | 1007 | 195921001 | Influenza with pneumonia NOS |
| UpperRespiratoryInfection-WRpt | 1007 | 195922008 | Influenza with other respiratory manifestation |
| UpperRespiratoryInfection-WRpt | 1007 | 195923003 | Influenza with laryngitis |
| UpperRespiratoryInfection-WRpt | 1007 | 195924009 | Influenza with pharyngitis |
| UpperRespiratoryInfection-WRpt | 1007 | 195925005 | Influenza with respiratory manifestations NOS |
| UpperRespiratoryInfection-WRpt | 1007 | 195927002 | Influenza with other manifestations |
| UpperRespiratoryInfection-WRpt | 1007 | 195930009 | Influenza with other manifestations NOS |
| UpperRespiratoryInfection-WRpt | 1007 | 195933006 | Other specified pneumonia or influenza |
| UpperRespiratoryInfection-WRpt | 1007 | 195934000 | Pneumonia or influenza NOS |
| UpperRespiratoryInfection-WRpt | 1007 | 195937007 | Tracheobronchitis NOS |
| UpperRespiratoryInfection-WRpt | 1007 | 196196007 | [X]Other acute sinusitis |
| UpperRespiratoryInfection-WRpt | 1007 | 196197003 | [X]Acute pharyngitis due to other specified organisms |
| UpperRespiratoryInfection-WRpt | 1007 | 196198008 | [X]Acute tonsillitis due to other specified organisms |
| UpperRespiratoryInfection-WRpt | 1007 | 196199000 | [X]Other acute upper respiratory infections of multiple sites |
| UpperRespiratoryInfection-WRpt | 1007 | 196200002 | [X]Influenza with other respiratory manifestations, influenza virus identified |
| UpperRespiratoryInfection-WRpt | 1007 | 196201003 | [X]Influenza with other manifestations, influenza virus identified |
| UpperRespiratoryInfection-WRpt | 1007 | 196202005 | [X]Influenza with other respiratory manifestations, virus not identified |
| UpperRespiratoryInfection-WRpt | 1007 | 196203000 | [X]Influenza with other manifestations, virus not identified |
| UpperRespiratoryInfection-WRpt | 1007 | 196220001 | [X]Other chronic sinusitis |
| UpperRespiratoryInfection-WRpt | 1007 | 196226007 | [X]Other abscess of pharynx |
| UpperRespiratoryInfection-WRpt | 1007 | 232251007 | Recurrent acute suppurative otitis media |
| UpperRespiratoryInfection-WRpt | 1007 | 232252000 | Recurrent acute non-suppurative otitis media |
| UpperRespiratoryInfection-WRpt | 1007 | 232311007 | Endocochlear cytomegalovirus infection |
| UpperRespiratoryInfection-WRpt | 1007 | 232363000 | Abscess of nasal cavity |
| UpperRespiratoryInfection-WRpt | 1007 | 232367004 | Nasal syphilis |
| UpperRespiratoryInfection-WRpt | 1007 | 232390009 | Suppurative sinusitis with complications |
| UpperRespiratoryInfection-WRpt | 1007 | 232391008 | Recurrent acute sinusitis |
| UpperRespiratoryInfection-WRpt | 1007 | 232393006 | Chronic anterior ethmoidal sinusitis |
| UpperRespiratoryInfection-WRpt | 1007 | 232394000 | Chronic posterior ethmoidal sinusitis |
| UpperRespiratoryInfection-WRpt | 1007 | 232395004 | Chronic panethmoidal sinusitis |
| UpperRespiratoryInfection-WRpt | 1007 | 232396003 | Chronic osteomeatal disease |
| UpperRespiratoryInfection-WRpt | 1007 | 232397007 | Chronic frontoethmoidal sinusitis |
| UpperRespiratoryInfection-WRpt | 1007 | 232399005 | Acute herpes simplex pharyngitis |
| UpperRespiratoryInfection-WRpt | 1007 | 232400003 | Acute herpes zoster pharyngitis |
| UpperRespiratoryInfection-WRpt | 1007 | 232401004 | Glandular fever pharyngitis |
| UpperRespiratoryInfection-WRpt | 1007 | 232402006 | Meningococcal pharyngitis |
| UpperRespiratoryInfection-WRpt | 1007 | 232403001 | Chlamydial pharyngitis |
| UpperRespiratoryInfection-WRpt | 1007 | 232404007 | Acute pharyngeal candidiasis |
| UpperRespiratoryInfection-WRpt | 1007 | 232405008 | Chronic ulcerative pharyngitis |
| UpperRespiratoryInfection-WRpt | 1007 | 232406009 | Chronic pharyngeal candidiasis |
| UpperRespiratoryInfection-WRpt | 1007 | 232417005 | Vincent's tonsillitis |
| UpperRespiratoryInfection-WRpt | 1007 | 232418000 | Acute infection of tonsillar remnant |
| UpperRespiratoryInfection-WRpt | 1007 | 232420002 | Chronic adenoiditis |
| UpperRespiratoryInfection-WRpt | 1007 | 232426008 | Acute simple laryngitis |
| UpperRespiratoryInfection-WRpt | 1007 | 232428009 | Acute membranous laryngitis |
| UpperRespiratoryInfection-WRpt | 1007 | 232429001 | Acute subglottic laryngitis |
| UpperRespiratoryInfection-WRpt | 1007 | 232430006 | Recurrent allergic croup |
| UpperRespiratoryInfection-WRpt | 1007 | 232432003 | Paediatric acute epiglottitis and supraglottitis |
| UpperRespiratoryInfection-WRpt | 1007 | 232433008 | Adult acute epiglottitis and supraglottitis |
| UpperRespiratoryInfection-WRpt | 1007 | 232434002 | Chronic fungal laryngitis |
| UpperRespiratoryInfection-WRpt | 1007 | 232439007 | Relapsing polychondritis of larynx |
| UpperRespiratoryInfection-WRpt | 1007 | 232460001 | Granulomatosis with polyangiitis of larynx |
| UpperRespiratoryInfection-WRpt | 1007 | 233596008 | Viral upper respiratory tract infection NOS |
| UpperRespiratoryInfection-WRpt | 1007 | 233799004 | Acute toxic tracheobronchitis |
| UpperRespiratoryInfection-WRpt | 1007 | 234528007 | Nasopharyngeal sarcoidosis |
| UpperRespiratoryInfection-WRpt | 1007 | 240444009 | Fusobacterial necrotising tonsillitis |
| UpperRespiratoryInfection-WRpt | 1007 | 240547000 | Lymphonodular coxsackie pharyngitis |
| UpperRespiratoryInfection-WRpt | 1007 | 240704003 | Pharyngeal candidiasis |
| UpperRespiratoryInfection-WRpt | 1007 | 240735009 | Classical histoplasmosis nasal ulceration |
| UpperRespiratoryInfection-WRpt | 1007 | 240913009 | Halzoun |
| UpperRespiratoryInfection-WRpt | 1007 | 249458000 | Supraglottic abscess |
| UpperRespiratoryInfection-WRpt | 1007 | 249463001 | Subglottic abscess |
| UpperRespiratoryInfection-WRpt | 1007 | 266146001 | Vincent's angina NOS |
| UpperRespiratoryInfection-WRpt | 1007 | 266337001 | Acute epiglottitis (non-streptococcal) |
| UpperRespiratoryInfection-WRpt | 1007 | 266338006 | Upper respiratory infection NOS |
| UpperRespiratoryInfection-WRpt | 1007 | 266353003 | Influenza NOS |
| UpperRespiratoryInfection-WRpt | 1007 | 267669008 | Otitis media NOS |
| UpperRespiratoryInfection-WRpt | 1007 | 267759006 | Chronic atticoantral disease |
| UpperRespiratoryInfection-WRpt | 1007 | 270490007 | Acute otitis media with effusion |
| UpperRespiratoryInfection-WRpt | 1007 | 271567008 | Whooping cough-like syndrome |
| UpperRespiratoryInfection-WRpt | 1007 | 275281000 | Catarrh |
| UpperRespiratoryInfection-WRpt | 1007 | 275376007 | Congenital syphilitic chronic coryza |
| UpperRespiratoryInfection-WRpt | 1007 | 275495004 | Acute fibrinous laryngotracheobronchitis |
| UpperRespiratoryInfection-WRpt | 1007 | 276443001 | Acute laryngitis and/or tracheitis |
| UpperRespiratoryInfection-WRpt | 1007 | 276700005 | Congenital syphilitic rhinitis |
| UpperRespiratoryInfection-WRpt | 1007 | 281794004 | Viral upper respiratory tract infection |
| UpperRespiratoryInfection-WRpt | 1007 | 301824001 | Acute viral laryngotracheitis |
| UpperRespiratoryInfection-WRpt | 1007 | 302911003 | Acute lingual tonsillitis |
| UpperRespiratoryInfection-WRpt | 1007 | 312118003 | Bacterial upper respiratory infection |
| UpperRespiratoryInfection-WRpt | 1007 | 312218008 | Infective otitis media |
| UpperRespiratoryInfection-WRpt | 1007 | 312400008 | Acute infective tracheobronchitis |
| UpperRespiratoryInfection-WRpt | 1007 | 312422001 | Infective pharyngitis |
| UpperRespiratoryInfection-WRpt | 1007 | 312423006 | Infective laryngitis |
| UpperRespiratoryInfection-WRpt | 1007 | 315642008 | Influenza-like symptoms |
| UpperRespiratoryInfection-WRpt | 1007 | 359609001 | Acute secretory otitis media |
| UpperRespiratoryInfection-WRpt | 1007 | 360595002 | Acute necrotising otitis media |
| UpperRespiratoryInfection-WRpt | 1007 | 363746003 | Acute pharyngitis |
| UpperRespiratoryInfection-WRpt | 1007 | 371103000 | Granulomatous epiglottitis |
| UpperRespiratoryInfection-WRpt | 1007 | 371127003 | Obstructive sinusitis |
| UpperRespiratoryInfection-WRpt | 1007 | 399095008 | Fusospirochaetal pharyngitis |
| UpperRespiratoryInfection-WRpt | 1007 | 405737000 | Pharyngitis |
| UpperRespiratoryInfection-WRpt | 1007 | 408669002 | Acute laryngitis with obstruction |
| UpperRespiratoryInfection-WRpt | 1007 | 408682005 | Healthcare associated pertussis |
| UpperRespiratoryInfection-WRpt | 1007 | 408687004 | Healthcare associated influenza disease |
| UpperRespiratoryInfection-WRpt | 1007 | 414304001 | Furuncle of nasal cavity |
| UpperRespiratoryInfection-WRpt | 1007 | 415724006 | Tonsillitis due to Gram negative bacteria |
| UpperRespiratoryInfection-WRpt | 1007 | 420706008 | Nasopharyngeal fascioliasis |
| UpperRespiratoryInfection-WRpt | 1007 | 424327005 | Tonsillar actinomycosis |
| UpperRespiratoryInfection-WRpt | 1007 | 427873006 | Influenza due to influenza virus type A, avian, H5N1 strain |
| UpperRespiratoryInfection-WRpt | 1007 | 427909005 | Chronic recurrent sinusitis |
| UpperRespiratoryInfection-WRpt | 1007 | 429427008 | Invasive fungal sinusitis |
| UpperRespiratoryInfection-WRpt | 1007 | 429759002 | Cerebrospinal fluid leak from nose and mouth |
| UpperRespiratoryInfection-WRpt | 1007 | 431231008 | Acute rhinosinusitis |
| UpperRespiratoryInfection-WRpt | 1007 | 432381000 | Human papilloma virus infection of vocal cord |
| UpperRespiratoryInfection-WRpt | 1007 | 441551009 | Viral laryngitis |
| UpperRespiratoryInfection-WRpt | 1007 | 442438000 | Influenza due to Influenza A virus |
| UpperRespiratoryInfection-WRpt | 1007 | 442696006 | Influenza due to Influenza A virus subtype H1N1 |
| UpperRespiratoryInfection-WRpt | 1007 | 444745000 | Infection of mucous cyst of nasal sinus |
| UpperRespiratoryInfection-WRpt | 1007 | 444814009 | Viral sinusitis |
| UpperRespiratoryInfection-WRpt | 1007 | 445130008 | Acute infective adenoiditis |
| UpperRespiratoryInfection-WRpt | 1007 | 446594000 | Infection of pharynx due to Chlamydia trachomatis |
| UpperRespiratoryInfection-WRpt | 1007 | 446753005 | Tuberculosis of oropharynx |
| UpperRespiratoryInfection-WRpt | 1007 | 450715004 | Influenza due to Influenza A virus subtype H7 |
| UpperRespiratoryInfection-WRpt | 1007 | 450716003 | Influenza due to Influenza A virus subtype H9 |
| UpperRespiratoryInfection-WRpt | 1007 | 703468005 | Bacterial tonsillitis |
| UpperRespiratoryInfection-WRpt | 1007 | 703469002 | Bacterial otitis media |
| UpperRespiratoryInfection-WRpt | 1007 | 703470001 | Bacterial sinusitis |
| UpperRespiratoryInfection-WRpt | 1007 | 707448003 | Influenza due to Influenza A virus subtype H7N9 |
| UpperRespiratoryInfection-WRpt | 1007 | 707509000 | Tracheobronchitis due to Aspergillus |
| UpperRespiratoryInfection-WRpt | 1007 | 709663002 | Supraglottitis |
| UpperRespiratoryInfection-WRpt | 1007 | 711128004 | Influenza due to influenza virus type A, avian, H3N2 strain |
| UpperRespiratoryInfection-WRpt | 1007 | 713083002 | Influenza caused by Influenza A virus subtype H5 |
| UpperRespiratoryInfection-WRpt | 1007 | 715593000 | Candidiasis of nose |
| UpperRespiratoryInfection-WRpt | 1007 | 715882005 | Severe acute respiratory syndrome of upper respiratory tract |
| UpperRespiratoryInfection-WRpt | 1007 | 716673006 | Infection of upper respiratory tract caused by fungus |
| UpperRespiratoryInfection-WRpt | 1007 | 717231003 | PFAPA syndrome |
| UpperRespiratoryInfection-WRpt | 1007 | 719522009 | Candidiasis of upper respiratory tract co-occurrent with human immunodeficiency virus infection |
| UpperRespiratoryInfection-WRpt | 1007 | 719590007 | Influenza caused by seasonal influenza virus |
| UpperRespiratoryInfection-WRpt | 1007 | 719865001 | Influenza caused by pandemic influenza virus |
| UpperRespiratoryInfection-WRpt | 1007 | 721586007 | Pharyngotonsillitis caused by Human herpes simplex virus |
| UpperRespiratoryInfection-WRpt | 1007 | 721741006 | Sinusitis caused by Streptococcus pneumoniae |
| UpperRespiratoryInfection-WRpt | 1007 | 721742004 | Otitis media caused by Streptococcus pneumoniae |
| UpperRespiratoryInfection-WRpt | 1007 | 721755003 | Sinusitis caused by Haemophilus influenzae |
| UpperRespiratoryInfection-WRpt | 1007 | 723880004 | Abscess of upper respiratory tract |
| UpperRespiratoryInfection-WRpt | 1007 | 725916003 | Acute sinusitis caused by fungus |
| UpperRespiratoryInfection-WRpt | 1007 | 725917007 | Acute sinusitis caused by virus |
| UpperRespiratoryInfection-WRpt | 1007 | 733170007 | Chronic aspergillosis of paranasal sinus |
| UpperRespiratoryInfection-WRpt | 1007 | 735463000 | Perichondritis of trachea |
| UpperRespiratoryInfection-WRpt | 1007 | 735740009 | Infection causing tracheitis in neonate |
| UpperRespiratoryInfection-WRpt | 1007 | 765152004 | Chronic serous otitis media of left ear |
| UpperRespiratoryInfection-WRpt | 1007 | 765153009 | Chronic serous otitis media of right ear |
| UpperRespiratoryInfection-WRpt | 1007 | 772810003 | Influenza caused by Influenza A virus subtype H3N2 |
| UpperRespiratoryInfection-WRpt | 1007 | 772828001 | Influenza caused by Influenza A virus subtype H5N1 |
| UpperRespiratoryInfection-WRpt | 1007 | 772839003 | Pneumonia caused by Influenza A virus |
| UpperRespiratoryInfection-WRpt | 1007 | 788964000 | Laryngeal myiasis |
| UpperRespiratoryInfection-WRpt | 1007 | 789057009 | Tuberculosis of middle ear |
| UpperRespiratoryInfection-WRpt | 1007 | 827090008 | Foul smelling discharge from nose |
| UpperRespiratoryInfection-WRpt | 1007 | 836475004 | Mucopurulent discharge from nose |
| UpperRespiratoryInfection-WRpt | 1007 | 838367000 | Allergic rhinosinusitis caused by Aspergillus |
| UpperRespiratoryInfection-WRpt | 1007 | 878818001 | Pharyngotonsillitis |
| UpperRespiratoryInfection-WRpt | 1007 | 897656009 | Rhinosinusitis |
| UpperRespiratoryInfection-WRpt | 1007 | 897657000 | Chronic rhinosinusitis |
| UpperRespiratoryInfection-WRpt | 1007 | 1010619008 | Inflammatory tracheobronchial papilloma |
| UpperRespiratoryInfection-WRpt | 1007 | 1010626008 | Recurrent peritonsillar abscess |
| UpperRespiratoryInfection-WRpt | 1007 | 1149091008 | Influenza caused by Influenza A virus subtype H2 |
| UpperRespiratoryInfection-WRpt | 1007 | 1163148001 | Chronic suppurative otitis media caused by Aspergillus |
| UpperRespiratoryInfection-WRpt | 1007 | 1163523005 | Fibrosis of upper respiratory tract due to paracoccidioidomycosis |
| UpperRespiratoryInfection-WRpt | 1007 | 1186932001 | Leakage of cerebrospinal fluid from nose following surgical procedure |
| UpperRespiratoryInfection-WRpt | 1007 | 7271000119107 | Acute bilateral otitis media with effusion |
| UpperRespiratoryInfection-WRpt | 1007 | 12181000119103 | Contact granuloma of larynx |
| UpperRespiratoryInfection-WRpt | 1007 | 16311000119108 | Pneumonia due to influenza |
| UpperRespiratoryInfection-WRpt | 1007 | 22951000119104 | Recurrent croup |
| UpperRespiratoryInfection-WRpt | 1007 | 41931000119102 | Sinusitis co-occurrent with nasal polyps |
| UpperRespiratoryInfection-WRpt | 1007 | 84261000119106 | Acute persistent otitis media |
| UpperRespiratoryInfection-WRpt | 1007 | 88171000119100 | Acute adenoiditis |
| UpperRespiratoryInfection-WRpt | 1007 | 104041000119108 | Fungal sinusitis |
| UpperRespiratoryInfection-WRpt | 1007 | 133171000119105 | Chronic pharyngolaryngitis |
| UpperRespiratoryInfection-WRpt | 1007 | 142921000119103 | Upper respiratory tract infection due to avian influenza |
| UpperRespiratoryInfection-WRpt | 1007 | 142931000119100 | Pneumonia due to H1N1 influenza |
| UpperRespiratoryInfection-WRpt | 1007 | 142941000119109 | Upper respiratory tract infection due to H1N1 influenza |
| UpperRespiratoryInfection-WRpt | 1007 | 143111000119103 | Pneumonia due to avian influenza |
| UpperRespiratoryInfection-WRpt | 1007 | 198691000000108 | Acute obstructive laryngitis |
| UpperRespiratoryInfection-WRpt | 1007 | 214081000000107 | Acute obstructive laryngitis |
| UpperRespiratoryInfection-WRpt | 1007 | 280331000000102 | Avian influenza |
| UpperRespiratoryInfection-WRpt | 1007 | 290051000119103 | Bilateral recurrent acute suppurative otitis media of middle ears |
| UpperRespiratoryInfection-WRpt | 1007 | 292631000000106 | Avian influenza |
| UpperRespiratoryInfection-WRpt | 1007 | 297831000000109 | Cerebrospinal fluid leak from nose and mouth |
| UpperRespiratoryInfection-WRpt | 1007 | 313281000000109 | Acute rhinosinusitis |
| UpperRespiratoryInfection-WRpt | 1007 | 328531000119104 | Upper respiratory tract infection due to Influenza A |
| UpperRespiratoryInfection-WRpt | 1007 | 338111000000109 | Acute rhinosinusitis |
| UpperRespiratoryInfection-WRpt | 1007 | 402101000000108 | [X]Other abscess of pharynx |
| UpperRespiratoryInfection-WRpt | 1007 | 402501000000101 | [X]Other chronic suppurative otitis media |
| UpperRespiratoryInfection-WRpt | 1007 | 411271000000104 | [X]Other acute upper respiratory infections of multiple sites |
| UpperRespiratoryInfection-WRpt | 1007 | 418181000000104 | [X]Influenza with other respiratory manifestations, influenza virus identified |
| UpperRespiratoryInfection-WRpt | 1007 | 418191000000102 | [X]Influenza with other manifestations, influenza virus identified |
| UpperRespiratoryInfection-WRpt | 1007 | 420141000000100 | [X]Otitis media in other diseases classified elsewhere |
| UpperRespiratoryInfection-WRpt | 1007 | 427821000000103 | [X]Acute pharyngitis due to other specified organisms |
| UpperRespiratoryInfection-WRpt | 1007 | 430891000000103 | [X]Influenza with other respiratory manifestations, virus not identified |
| UpperRespiratoryInfection-WRpt | 1007 | 441131000000104 | [X]Influenza with other manifestations, virus not identified |
| UpperRespiratoryInfection-WRpt | 1007 | 444061000000106 | [X]Whooping cough due to other Bordetella species |
| UpperRespiratoryInfection-WRpt | 1007 | 444121000000104 | [X]Other acute non-suppurative otitis media |
| UpperRespiratoryInfection-WRpt | 1007 | 452761000000100 | [X]Acute tonsillitis due to other specified organisms |
| UpperRespiratoryInfection-WRpt | 1007 | 454211000000100 | [X]Other acute sinusitis |
| UpperRespiratoryInfection-WRpt | 1007 | 455311000000109 | [X]Other chronic sinusitis |
| UpperRespiratoryInfection-WRpt | 1007 | 456041000000104 | [X]Whooping cough, unspecified |
| UpperRespiratoryInfection-WRpt | 1007 | 505131000000104 | Influenza due to Influenza A virus subtype H1N1 |
| UpperRespiratoryInfection-WRpt | 1007 | 510671000000104 | Influenza due to Influenza A virus subtype H1N1 |
| UpperRespiratoryInfection-WRpt | 1007 | 538321000000103 | Streptococcal sore throat NOS |
| UpperRespiratoryInfection-WRpt | 1007 | 539811000000107 | Chronic serous otitis media NOS |
| UpperRespiratoryInfection-WRpt | 1007 | 539871000000102 | Acute suppurative otitis media NOS |
| UpperRespiratoryInfection-WRpt | 1007 | 540121000000103 | Influenza with other manifestations |
| UpperRespiratoryInfection-WRpt | 1007 | 540131000000101 | Influenza with other manifestations NOS |
| UpperRespiratoryInfection-WRpt | 1007 | 540141000000105 | Other specified pneumonia or influenza |
| UpperRespiratoryInfection-WRpt | 1007 | 540151000000108 | Pneumonia or influenza NOS |
| UpperRespiratoryInfection-WRpt | 1007 | 546371000000104 | Whooping cough NOS |
| UpperRespiratoryInfection-WRpt | 1007 | 551571000000104 | Glue ear, unspecified |
| UpperRespiratoryInfection-WRpt | 1007 | 555361000000102 | Other whooping cough NOS |
| UpperRespiratoryInfection-WRpt | 1007 | 570791000000100 | Whooping cough - other specified organism |
| UpperRespiratoryInfection-WRpt | 1007 | 576581000000107 | Acute viral laryngitis unspecified |
| UpperRespiratoryInfection-WRpt | 1007 | 579671000000101 | Other chronic sinusitis |
| UpperRespiratoryInfection-WRpt | 1007 | 579681000000104 | Other chronic sinusitis NOS |
| UpperRespiratoryInfection-WRpt | 1007 | 598921000000106 | Unspecified acute tympanitis |
| UpperRespiratoryInfection-WRpt | 1007 | 598931000000108 | Acute myringitis NOS |
| UpperRespiratoryInfection-WRpt | 1007 | 600041000000109 | Catarrhal otitis media NOS |
| UpperRespiratoryInfection-WRpt | 1007 | 600051000000107 | Non-suppurative otitis media NOS |
| UpperRespiratoryInfection-WRpt | 1007 | 600981000000100 | Other acute sinusitis NOS |
| UpperRespiratoryInfection-WRpt | 1007 | 600991000000103 | Acute sinusitis NOS |
| UpperRespiratoryInfection-WRpt | 1007 | 603081000000108 | Acute bacterial laryngitis unspecified |
| UpperRespiratoryInfection-WRpt | 1007 | 603091000000105 | Acute laryngitis NOS |
| UpperRespiratoryInfection-WRpt | 1007 | 603101000000102 | Acute tracheitis NOS |
| UpperRespiratoryInfection-WRpt | 1007 | 603121000000106 | Acute epiglottitis NOS |
| UpperRespiratoryInfection-WRpt | 1007 | 603131000000108 | Acute laryngitis and tracheitis NOS |
| UpperRespiratoryInfection-WRpt | 1007 | 603141000000104 | Other acute upper respiratory infections |
| UpperRespiratoryInfection-WRpt | 1007 | 605921000000107 | Chronic laryngitis NOS |
| UpperRespiratoryInfection-WRpt | 1007 | 606871000000108 | Unspecified non-suppurative otitis media |
| UpperRespiratoryInfection-WRpt | 1007 | 606881000000105 | Allergic otitis media NOS |
| UpperRespiratoryInfection-WRpt | 1007 | 607911000000108 | Other acute sinusitis |
| UpperRespiratoryInfection-WRpt | 1007 | 607921000000102 | Acute pharyngitis NOS |
| UpperRespiratoryInfection-WRpt | 1007 | 607931000000100 | Acute laryngotracheitis NOS |
| UpperRespiratoryInfection-WRpt | 1007 | 611481000000104 | Chronic sinusitis NOS |
| UpperRespiratoryInfection-WRpt | 1007 | 616161000000107 | Influenza with pneumonia NOS |
| UpperRespiratoryInfection-WRpt | 1007 | 616171000000100 | Influenza with other respiratory manifestation |
| UpperRespiratoryInfection-WRpt | 1007 | 616181000000103 | Influenza with respiratory manifestations NOS |
| UpperRespiratoryInfection-WRpt | 1007 | 619661000000107 | Chronic otitis media with effusion, unspecified |
| UpperRespiratoryInfection-WRpt | 1007 | 621131000000104 | Acute non-suppurative otitis media NOS |
| UpperRespiratoryInfection-WRpt | 1007 | 621141000000108 | Chronic mucoid otitis media NOS |
| UpperRespiratoryInfection-WRpt | 1007 | 621151000000106 | Chronic otitis media with effusion, other |
| UpperRespiratoryInfection-WRpt | 1007 | 622321000000109 | O/E - nasal discharge NOS |
| UpperRespiratoryInfection-WRpt | 1007 | 623441000000100 | Acute bacterial pharyngitis NOS |
| UpperRespiratoryInfection-WRpt | 1007 | 623461000000104 | Acute bacterial tonsillitis NOS |
| UpperRespiratoryInfection-WRpt | 1007 | 623471000000106 | Acute tonsillitis NOS |
| UpperRespiratoryInfection-WRpt | 1007 | 623611000000103 | Polypoid sinus degeneration NOS |
| UpperRespiratoryInfection-WRpt | 1007 | 623621000000109 | Chronic pharyngitis NOS |
| UpperRespiratoryInfection-WRpt | 1007 | 623631000000106 | Chronic pharyngitis and nasopharyngitis NOS |
| UpperRespiratoryInfection-WRpt | 1007 | 643741000000108 | Serous otitis media NOS |
| UpperRespiratoryInfection-WRpt | 1007 | 643751000000106 | Mucoid otitis media NOS |
| UpperRespiratoryInfection-WRpt | 1007 | 644251000000101 | Viral upper respiratory tract infection NOS |
| UpperRespiratoryInfection-WRpt | 1007 | 645631000000105 | Other upper respiratory infections of multiple sites |
| UpperRespiratoryInfection-WRpt | 1007 | 647141000000100 | Otitis media NOS |
| UpperRespiratoryInfection-WRpt | 1007 | 649111000000109 | Tracheobronchitis NOS |
| UpperRespiratoryInfection-WRpt | 1007 | 670551000000108 | Influenza NOS |
| UpperRespiratoryInfection-WRpt | 1007 | 686051000000106 | Vincent's angina NOS |
| UpperRespiratoryInfection-WRpt | 1007 | 693581000000101 | Upper respiratory infection NOS |
| UpperRespiratoryInfection-WRpt | 1007 | 694131000000108 | Chronic suppurative otitis media NOS |
| UpperRespiratoryInfection-WRpt | 1007 | 849571000000102 | Acute bacterial laryngitis |
| UpperRespiratoryInfection-WRpt | 1007 | 856211000006111 | Post influenzal debility |
| UpperRespiratoryInfection-WRpt | 1007 | 877891000000108 | Pharyngeal lymphogranuloma venereum |
| UpperRespiratoryInfection-WRpt | 1007 | 877901000000109 | Pharyngeal lymphogranuloma venereum |
| UpperRespiratoryInfection-WRpt | 1007 | 1033051000000101 | Influenza due to zoonotic influenza virus |
| UpperRespiratoryInfection-WRpt | 1007 | 1033061000000103 | Influenza due to zoonotic influenza virus |
| UpperRespiratoryInfection-WRpt | 1007 | 1033071000000105 | Influenza due to pandemic influenza virus |
| UpperRespiratoryInfection-WRpt | 1007 | 1033081000000107 | Influenza due to pandemic influenza virus |
| UpperRespiratoryInfection-WRpt | 1007 | 1033091000000109 | Influenza due to seasonal influenza virus |
| UpperRespiratoryInfection-WRpt | 1007 | 1033101000000101 | Influenza due to seasonal influenza virus |
| UpperRespiratoryInfection-WRpt | 1007 | 1033111000000104 | Influenza with pneumonia due to seasonal influenza virus |
| UpperRespiratoryInfection-WRpt | 1007 | 1033121000000105 | Influenzal bronchopneumonia due to seasonal influenza virus |
| UpperRespiratoryInfection-WRpt | 1007 | 1050601000000101 | Influenza due to seasonal influenza virus |
| UpperRespiratoryInfection-WRpt | 1007 | 1050981000000100 | Influenza due to seasonal influenza virus |
| UpperRespiratoryInfection-WRpt | 1007 | 1082561000119104 | Recurrent acute suppurative otitis media with spontaneous rupture of ear drum |
| UpperRespiratoryInfection-WRpt | 1007 | 1082871000119108 | Bilateral acute allergic otitis media of middle ears |
| UpperRespiratoryInfection-WRpt | 1007 | 1082911000119106 | Bilateral acute eustachian salpingitis |
| UpperRespiratoryInfection-WRpt | 1007 | 1082951000119107 | Bilateral mucoid otitis media of middle ears |
| UpperRespiratoryInfection-WRpt | 1007 | 1083051000119102 | Bilateral recurrent acute serous otitis media of middle ears |
| UpperRespiratoryInfection-WRpt | 1007 | 1083061000119100 | Bilateral spontaneous rupture of tympanic membranes of ears co-occurrent and due to recurrent acute suppurative otitis media |
| UpperRespiratoryInfection-WRpt | 1007 | 1083421000119103 | Bilateral chronic serosanguineous otitis media of middle ears |
| UpperRespiratoryInfection-WRpt | 1007 | 1083431000119100 | Bilateral chronic suppurative otitis media of middle ears |
| UpperRespiratoryInfection-WRpt | 1007 | 1087981000119103 | Acute allergic otitis media of left middle ear |
| UpperRespiratoryInfection-WRpt | 1007 | 1088021000119100 | Left acute eustachian salpingitis |
| UpperRespiratoryInfection-WRpt | 1007 | 1088061000119105 | Acute mucoid otitis media of left middle ear |
| UpperRespiratoryInfection-WRpt | 1007 | 1088181000119100 | Recurrent acute suppurative otitis media of left ear with spontaneous rupture of tympanic membrane |
| UpperRespiratoryInfection-WRpt | 1007 | 1088401000119103 | Chronic atticoantral suppurative otitis media of left middle ear |
| UpperRespiratoryInfection-WRpt | 1007 | 1088551000119103 | Left chronic serosanguineous otitis media |
| UpperRespiratoryInfection-WRpt | 1007 | 1088571000119107 | Chronic suppurative otitis media of left middle ear |
| UpperRespiratoryInfection-WRpt | 1007 | 1088581000119105 | Chronic tubotympanic suppurative otitis media of left middle ear |
| UpperRespiratoryInfection-WRpt | 1007 | 1090001000119105 | Otitis media due to scarlet fever |
| UpperRespiratoryInfection-WRpt | 1007 | 1090211000119102 | Pharyngeal diphtheria |
| UpperRespiratoryInfection-WRpt | 1007 | 1090601000119102 | Acute allergic otitis media of right middle ear |
| UpperRespiratoryInfection-WRpt | 1007 | 1090641000119100 | Right acute eustachian salpingitis |
| UpperRespiratoryInfection-WRpt | 1007 | 1090681000119105 | Acute mucoid otitis media of right middle ear |
| UpperRespiratoryInfection-WRpt | 1007 | 1090791000119102 | Recurrent acute suppurative otitis media of right ear with spontaneous rupture of tympanic membrane |
| UpperRespiratoryInfection-WRpt | 1007 | 1091011000119101 | Chronic atticoantral suppurative otitis media of right middle ear |
| UpperRespiratoryInfection-WRpt | 1007 | 1091161000119104 | Right chronic serosanguineous otitis media |
| UpperRespiratoryInfection-WRpt | 1007 | 1091181000119108 | Chronic suppurative otitis media of right middle ear |
| UpperRespiratoryInfection-WRpt | 1007 | 1091191000119106 | Chronic tubotympanic suppurative otitis media of right middle ear |
| UpperRespiratoryInfection-WRpt | 1007 | 1240521000000100 | Otitis media due to disease caused by SARS-CoV-2 (severe acute respiratory syndrome coronavirus 2) |
| UpperRespiratoryInfection-WRpt | 1007 | 1240541000000107 | Upper respiratory tract infection caused by SARS-CoV-2 (severe acute respiratory syndrome coronavirus 2) |
| UpperRespiratoryInfection-WRpt | 1007 | 1787121000006116 | Community acquired pneumonia |
| UpperRespiratoryInfection-WRpt | 1007 | 1787131000006118 | Hospital acquired pneumonia |
| UpperRespiratoryInfection-WRpt | 1007 | 10624911000119107 | Otitis media due to H1N1 influenza |
| UpperRespiratoryInfection-WRpt | 1007 | 10624951000119108 | Otitis media due to influenza |
| UpperRespiratoryInfection-WRpt | 1007 | 10629191000119100 | Bronchiolitis caused by influenza virus |
| UpperRespiratoryInfection-WRpt | 1007 | 10629231000119109 | Recurrent acute streptococcal tonsillitis |
| UpperRespiratoryInfection-WRpt | 1007 | 10629551000119101 | Recurrent acute sphenoid sinusitis |
| UpperRespiratoryInfection-WRpt | 1007 | 10629591000119106 | Recurrent acute frontal sinusitis |
| UpperRespiratoryInfection-WRpt | 1007 | 10629631000119106 | Recurrent acute pansinusitis |
| UpperRespiratoryInfection-WRpt | 1007 | 10629671000119109 | Recurrent acute maxillary sinusitis |
| UpperRespiratoryInfection-WRpt | 1007 | 10629711000119108 | Recurrent acute ethmoid sinusitis |
| UpperRespiratoryInfection-WRpt | 1007 | 10674911000119108 | Otitis media due to Influenza A virus |
| UpperRespiratoryInfection-WRpt | 1007 | 10685111000119102 | Upper respiratory tract infection due to Influenza |
| UpperRespiratoryInfection-WRpt | 1007 | 12366661000119100 | Recurrent croup |

**Table S2: SNOMED CT Expression Constraint Language (ECL) codes for asthma**

| **Condition ID** | **Condition Name** | **ECL Element** | **ConceptID** |
| --- | --- | --- | --- |
| 4204 | AsthmaExacerbation | AddSupertype | 281239006 |
| 4210 | AsthmaManagementNoPlan | AddSubtype | 176711000000100 |
| 4210 | AsthmaManagementNoPlan | AddSubtype | 892301000000100 |
| 4209 | AsthmaManagementPlan | AddSubtype | 736056000 |
| 4209 | AsthmaManagementPlan | AddSubtype | 811921000000103 |
| 4209 | AsthmaManagementPlan | AddSupertype | 390872009 |
| 4208 | AsthmaReview | AddSubtype | 394700004 |
| 4208 | AsthmaReview | AddSubtype | 394720003 |
| 4208 | AsthmaReview | AddSubtype | 754061000000100 |
| 7163 | HospitalAdmission | AddSupertype | 32485007 |
| 1020 | Influenza-likeIllness | AddSubtype | 315642008 |
| 1020 | Influenza-likeIllness | AddSupertype | 6142004 |
| 1020 | Influenza-likeIllness | AddSupertype | 78046005 |
| 1020 | Influenza-likeIllness | AddSupertype | 95891005 |
| 1020 | Influenza-likeIllness | AddSupertype | 195929004 |
| 1020 | Influenza-likeIllness | AddSupertype | 309789002 |
| 1055 | LowerRespiratoryTractInfection | AddSubtype | 7063008 |
| 1055 | LowerRespiratoryTractInfection | AddSubtype | 18988001 |
| 1055 | LowerRespiratoryTractInfection | AddSubtype | 27757009 |
| 1055 | LowerRespiratoryTractInfection | AddSubtype | 29731002 |
| 1055 | LowerRespiratoryTractInfection | AddSubtype | 36426008 |
| 1055 | LowerRespiratoryTractInfection | AddSubtype | 71186008 |
| 1055 | LowerRespiratoryTractInfection | AddSubtype | 71255007 |
| 1055 | LowerRespiratoryTractInfection | AddSubtype | 73414003 |
| 1055 | LowerRespiratoryTractInfection | AddSubtype | 85469005 |
| 1055 | LowerRespiratoryTractInfection | AddSubtype | 85915003 |
| 1055 | LowerRespiratoryTractInfection | AddSubtype | 187196002 |
| 1055 | LowerRespiratoryTractInfection | AddSubtype | 195949008 |
| 1055 | LowerRespiratoryTractInfection | AddSubtype | 195951007 |
| 1055 | LowerRespiratoryTractInfection | AddSubtype | 233604007 |
| 1055 | LowerRespiratoryTractInfection | AddSubtype | 233617005 |
| 1055 | LowerRespiratoryTractInfection | AddSubtype | 233619008 |
| 1055 | LowerRespiratoryTractInfection | AddSubtype | 314978007 |
| 1055 | LowerRespiratoryTractInfection | AddSubtype | 385093006 |
| 1055 | LowerRespiratoryTractInfection | AddSubtype | 1092951000119106 |
| 1055 | LowerRespiratoryTractInfection | AddSupertype | 4120002 |
| 1055 | LowerRespiratoryTractInfection | AddSupertype | 10509002 |
| 1055 | LowerRespiratoryTractInfection | AddSupertype | 13617004 |
| 1055 | LowerRespiratoryTractInfection | AddSupertype | 50417007 |
| 1055 | LowerRespiratoryTractInfection | AddSupertype | 58554001 |
| 1055 | LowerRespiratoryTractInfection | AddSupertype | 64667001 |
| 1055 | LowerRespiratoryTractInfection | AddSupertype | 73452002 |
| 1055 | LowerRespiratoryTractInfection | AddSupertype | 78895009 |
| 1055 | LowerRespiratoryTractInfection | AddSupertype | 278516003 |
| 1055 | LowerRespiratoryTractInfection | AddSupertype | 312134000 |
| 1055 | LowerRespiratoryTractInfection | AddSupertype | 314042000 |
| 1055 | LowerRespiratoryTractInfection | AddSupertype | 396285007 |
| 1055 | LowerRespiratoryTractInfection | AddSupertype | 398447004 |
| 1055 | LowerRespiratoryTractInfection | AddSupertype | 407671000 |
| 1055 | LowerRespiratoryTractInfection | AddSupertype | 417018008 |
| 1055 | LowerRespiratoryTractInfection | AddSupertype | 446986002 |
| 1055 | LowerRespiratoryTractInfection | MinusSubtype | 196019004 |
| 1055 | LowerRespiratoryTractInfection | MinusSubtype | 846639005 |
| 1055 | LowerRespiratoryTractInfection | MinusSubtype | 866901000000103 |
| 1055 | LowerRespiratoryTractInfection | MinusSupertype | 40100001 |
| 1055 | LowerRespiratoryTractInfection | MinusSupertype | 62994001 |
| 1055 | LowerRespiratoryTractInfection | MinusSupertype | 66011008 |
| 1055 | LowerRespiratoryTractInfection | MinusSupertype | 405720007 |
| 1055 | LowerRespiratoryTractInfection | MinusSupertype | 405944004 |
| 1055 | LowerRespiratoryTractInfection | MinusSupertype | 700249006 |
| 1055 | LowerRespiratoryTractInfection | MinusSupertype | 10625791000119101 |
| 7183 | SeenInHospitalCasualty | AddSubtype | 4525004 |
| 7183 | SeenInHospitalCasualty | AddSubtype | 305226003 |
| 7183 | SeenInHospitalCasualty | AddSubtype | 305451000 |
| 7183 | SeenInHospitalCasualty | AddSubtype | 305633005 |
| 7183 | SeenInHospitalCasualty | AddSubtype | 305925007 |
| 7183 | SeenInHospitalCasualty | AddSubtype | 306390007 |
| 7183 | SeenInHospitalCasualty | AddSubtype | 397721007 |
| 7183 | SeenInHospitalCasualty | AddSubtype | 413845009 |
| 7183 | SeenInHospitalCasualty | AddSubtype | 417119002 |
| 7183 | SeenInHospitalCasualty | AddSubtype | 507291000000100 |
| 7183 | SeenInHospitalCasualty | AddSubtype | 812481000000104 |
| 7183 | SeenInHospitalCasualty | AddSubtype | 812491000000102 |
| 7183 | SeenInHospitalCasualty | AddSubtype | 826931000000104 |
| 7183 | SeenInHospitalCasualty | AddSubtype | 963261000000108 |
| 7183 | SeenInHospitalCasualty | AddSubtype | 980491000000106 |
| 7183 | SeenInHospitalCasualty | AddSubtype | 989531000000100 |
| 7183 | SeenInHospitalCasualty | AddSubtype | 1066331000000109 |
| 7183 | SeenInHospitalCasualty | AddSubtype | 1066341000000100 |
| 7183 | SeenInHospitalCasualty | AddSubtype | 1066361000000104 |
| 7183 | SeenInHospitalCasualty | AddSubtype | 1066371000000106 |
| 7183 | SeenInHospitalCasualty | AddSubtype | 1066381000000108 |
| 7183 | SeenInHospitalCasualty | AddSubtype | 1082421000000101 |
| 7183 | SeenInHospitalCasualty | AddSupertype | 50849002 |
| 7183 | SeenInHospitalCasualty | AddSupertype | 185210004 |
| 7183 | SeenInHospitalCasualty | AddSupertype | 306563004 |
| 7183 | SeenInHospitalCasualty | AddSupertype | 1066391000000105 |
| 7183 | SeenInHospitalCasualty | AddSupertype | 1077011000000106 |
| 1007 | UpperRespiratoryInfection | AddSubtype | 8304007 |
| 1007 | UpperRespiratoryInfection | AddSubtype | 13420004 |
| 1007 | UpperRespiratoryInfection | AddSubtype | 16664009 |
| 1007 | UpperRespiratoryInfection | AddSubtype | 65363002 |
| 1007 | UpperRespiratoryInfection | AddSubtype | 194202008 |
| 1007 | UpperRespiratoryInfection | AddSubtype | 194203003 |
| 1007 | UpperRespiratoryInfection | AddSubtype | 194286008 |
| 1007 | UpperRespiratoryInfection | AddSubtype | 195680000 |
| 1007 | UpperRespiratoryInfection | AddSubtype | 232311007 |
| 1007 | UpperRespiratoryInfection | AddSubtype | 271567008 |
| 1007 | UpperRespiratoryInfection | AddSubtype | 276443001 |
| 1007 | UpperRespiratoryInfection | AddSubtype | 315642008 |
| 1007 | UpperRespiratoryInfection | AddSubtype | 1090001000119105 |
| 1007 | UpperRespiratoryInfection | AddSubtype | 1240521000000100 |
| 1007 | UpperRespiratoryInfection | AddSupertype | 140004 |
| 1007 | UpperRespiratoryInfection | AddSupertype | 297009 |
| 1007 | UpperRespiratoryInfection | AddSupertype | 3110003 |
| 1007 | UpperRespiratoryInfection | AddSupertype | 6142004 |
| 1007 | UpperRespiratoryInfection | AddSupertype | 27836007 |
| 1007 | UpperRespiratoryInfection | AddSupertype | 33924006 |
| 1007 | UpperRespiratoryInfection | AddSupertype | 36971009 |
| 1007 | UpperRespiratoryInfection | AddSupertype | 45913009 |
| 1007 | UpperRespiratoryInfection | AddSupertype | 54150009 |
| 1007 | UpperRespiratoryInfection | AddSupertype | 54398005 |
| 1007 | UpperRespiratoryInfection | AddSupertype | 59707005 |
| 1007 | UpperRespiratoryInfection | AddSupertype | 62994001 |
| 1007 | UpperRespiratoryInfection | AddSupertype | 64531003 |
| 1007 | UpperRespiratoryInfection | AddSupertype | 80327007 |
| 1007 | UpperRespiratoryInfection | AddSupertype | 80384002 |
| 1007 | UpperRespiratoryInfection | AddSupertype | 90176007 |
| 1007 | UpperRespiratoryInfection | AddSupertype | 195709006 |
| 1007 | UpperRespiratoryInfection | AddSupertype | 312218008 |
| 1007 | UpperRespiratoryInfection | AddSupertype | 359609001 |
| 1007 | UpperRespiratoryInfection | AddSupertype | 405737000 |
| 1007 | UpperRespiratoryInfection | AddSupertype | 723880004 |
| 1007 | UpperRespiratoryInfection | MinusSubtype | 1282001 |
| 1007 | UpperRespiratoryInfection | MinusSubtype | 2091005 |
| 1007 | UpperRespiratoryInfection | MinusSubtype | 54287007 |
| 1007 | UpperRespiratoryInfection | MinusSubtype | 59454008 |
| 1007 | UpperRespiratoryInfection | MinusSubtype | 59471009 |
| 1007 | UpperRespiratoryInfection | MinusSubtype | 87326000 |
| 1007 | UpperRespiratoryInfection | MinusSubtype | 111277007 |
| 1007 | UpperRespiratoryInfection | MinusSubtype | 195663004 |
| 1007 | UpperRespiratoryInfection | MinusSubtype | 232389000 |
| 1007 | UpperRespiratoryInfection | MinusSubtype | 232458003 |
| 1007 | UpperRespiratoryInfection | MinusSubtype | 262678008 |
| 1007 | UpperRespiratoryInfection | MinusSubtype | 307500008 |
| 1007 | UpperRespiratoryInfection | MinusSubtype | 425011002 |
| 1007 | UpperRespiratoryInfection | MinusSubtype | 427780002 |
| 1007 | UpperRespiratoryInfection | MinusSubtype | 10692681000119108 |
| 1007 | UpperRespiratoryInfection | MinusSupertype | 17904003 |
| 1007 | UpperRespiratoryInfection | MinusSupertype | 23919004 |
| 1007 | UpperRespiratoryInfection | MinusSupertype | 86773000 |
| 1007 | UpperRespiratoryInfection | MinusSupertype | 232214001 |
| 1007 | UpperRespiratoryInfection | MinusSupertype | 402698005 |

**Table S3: DM+D codes for prescriptions for asthma**

| **ConditionID** | **Condition name** | **DMD code** | **Description** |
| --- | --- | --- | --- |
| 5153 | Antimuscarinics | 320339009 | Ipratropium bromide 40microgram inhalation powder capsules |
| 5153 | Antimuscarinics | 320340006 | Ipratropium bromide 40microgram inhalation powder capsules with device |
| 5153 | Antimuscarinics | 320345001 | Ipratropium bromide 20micrograms/dose breath actuated inhaler |
| 5153 | Antimuscarinics | 320346000 | Ipratropium bromide 250micrograms/1ml nebuliser liquid unit dose vials |
| 5153 | Antimuscarinics | 320347009 | Ipratropium bromide 500micrograms/2ml nebuliser liquid unit dose vials |
| 5153 | Antimuscarinics | 320348004 | Ipratropium bromide 20micrograms/dose inhaler |
| 5153 | Antimuscarinics | 320350007 | Ipratropium bromide 40micrograms/dose inhaler |
| 5153 | Antimuscarinics | 320440005 | Fenoterol 100micrograms/dose / Ipratropium 40micrograms/dose inhaler |
| 5153 | Antimuscarinics | 320441009 | Fenoterol 100micrograms/dose / Ipratropium bromide 40micrograms/dose breath actuated inhaler |
| 5153 | Antimuscarinics | 320442002 | Salbutamol 100micrograms/dose / Ipratropium 20micrograms/dose inhaler |
| 5153 | Antimuscarinics | 320445000 | Salbutamol 2.5mg/2.5ml / Ipratropium bromide 500micrograms/2.5ml nebuliser liquid unit dose vials |
| 5153 | Antimuscarinics | 349394001 | Fenoterol 1.25mg/4ml / Ipratropium 500micrograms/4ml nebuliser liquid unit dose vials |
| 5153 | Antimuscarinics | 414538008 | Ipratropium bromide 20mcg CFC-free inhaler |
| 5153 | Antimuscarinics | 703924000 | Aclidinium bromide 375micrograms inhaler |
| 5153 | Antimuscarinics | 24011000001103 | Atrovent 20micrograms/dose inhaler (Boehringer Ingelheim Ltd) |
| 5153 | Antimuscarinics | 109751000001104 | Fenoterol 1.25mg/4ml / Ipratropium 500micrograms/4ml nebuliser liquid unit dose vials |
| 5153 | Antimuscarinics | 110221000001105 | Ipratropium bromide 500micrograms/2ml nebuliser liquid unit dose vials |
| 5153 | Antimuscarinics | 110481000001107 | Ipratropium bromide 250micrograms/1ml nebuliser liquid unit dose vials |
| 5153 | Antimuscarinics | 110651000001100 | Salbutamol 2.5mg/2.5ml / Ipratropium bromide 500micrograms/2.5ml nebuliser liquid unit dose vials |
| 5153 | Antimuscarinics | 111781000001105 | Ipratropium bromide 40microgram inhalation powder capsules with device |
| 5153 | Antimuscarinics | 112141000001107 | Ipratropium bromide 40microgram inhalation powder capsules |
| 5153 | Antimuscarinics | 347611000001103 | Atrovent Forte 40micrograms/dose inhaler (Boehringer Ingelheim Ltd) |
| 5153 | Antimuscarinics | 351411000001106 | Atrovent 20micrograms/dose Autohaler (Boehringer Ingelheim Ltd) |
| 5153 | Antimuscarinics | 2923111000001107 | Duovent inhaler (Boehringer Ingelheim Ltd) |
| 5153 | Antimuscarinics | 2964011000001104 | Duovent Autohaler (Boehringer Ingelheim Ltd) |
| 5153 | Antimuscarinics | 3221011000001103 | Ipratropium bromide 500micrograms/2ml nebuliser liquid unit dose vials (A A H Pharmaceuticals Ltd) |
| 5153 | Antimuscarinics | 3221911000001104 | Tropiovent 500micrograms/2ml nebuliser liquid unit dose Steripoule vials (Ashbourne Pharmaceuticals Ltd) |
| 5153 | Antimuscarinics | 3222411000001102 | Atrovent 500micrograms/2ml nebuliser liquid UDVs (Boehringer Ingelheim Ltd) |
| 5153 | Antimuscarinics | 3222711000001108 | Ipratropium bromide 500micrograms/2ml nebuliser liquid unit dose vials (The Boots Company Plc) |
| 5153 | Antimuscarinics | 3222911000001105 | Respontin 500micrograms/2ml Nebules (GlaxoSmithKline UK Ltd) |
| 5153 | Antimuscarinics | 3223111000001101 | Ipratropium 500micrograms/2ml nebuliser liquid Steri-Neb unit dose vials (Teva UK Ltd) |
| 5153 | Antimuscarinics | 3223511000001105 | Ipratropium bromide 500micrograms/2ml nebuliser liquid unit dose vials (Alliance Healthcare (Distribution) Ltd) |
| 5153 | Antimuscarinics | 3225311000001103 | Ipratropium bromide 250micrograms/1ml nebuliser liquid unit dose vials (A A H Pharmaceuticals Ltd) |
| 5153 | Antimuscarinics | 3225811000001107 | Tropiovent 250micrograms/1ml nebuliser liquid unit dose Steripoule vials (Ashbourne Pharmaceuticals Ltd) |
| 5153 | Antimuscarinics | 3226411000001101 | Atrovent 250micrograms/1ml nebuliser liquid UDVs (Boehringer Ingelheim Ltd) |
| 5153 | Antimuscarinics | 3226811000001104 | Ipratropium bromide 250micrograms/1ml nebuliser liquid unit dose vials (The Boots Company Plc) |
| 5153 | Antimuscarinics | 3227011000001108 | Respontin 250micrograms/1ml Nebules (GlaxoSmithKline UK Ltd) |
| 5153 | Antimuscarinics | 3227211000001103 | Ipratropium 250micrograms/1ml nebuliser liquid Steri-Neb unit dose vials (Teva UK Ltd) |
| 5153 | Antimuscarinics | 3227511000001100 | Ipratropium bromide 250micrograms/1ml nebuliser liquid unit dose vials (Alliance Healthcare (Distribution) Ltd) |
| 5153 | Antimuscarinics | 3235911000001100 | Atrovent 40microgram Aerocaps (Boehringer Ingelheim Ltd) |
| 5153 | Antimuscarinics | 3238911000001106 | Atrovent 40microgram Aerocaps with Aerohaler (Boehringer Ingelheim Ltd) |
| 5153 | Antimuscarinics | 3348611000001107 | Combivent inhaler (Boehringer Ingelheim Ltd) |
| 5153 | Antimuscarinics | 3378211000001106 | Spiriva 18microgram inhalation powder capsules with HandiHaler (Boehringer Ingelheim Ltd) |
| 5153 | Antimuscarinics | 3380011000001106 | Spiriva 18microgram inhalation powder capsules (Boehringer Ingelheim Ltd) |
| 5153 | Antimuscarinics | 3406011000001104 | Combivent nebuliser liquid 2.5ml UDVs (Boehringer Ingelheim Ltd) |
| 5153 | Antimuscarinics | 4192411000001109 | Duovent UDVs nebuliser liquid 4ml (Boehringer Ingelheim Ltd) |
| 5153 | Antimuscarinics | 5255211000001100 | Atrovent 20micrograms/dose inhaler (Waymade Healthcare Plc) |
| 5153 | Antimuscarinics | 5259111000001108 | Combivent inhaler (Waymade Healthcare Plc) |
| 5153 | Antimuscarinics | 5281611000001108 | Atrovent 20micrograms/dose inhaler (Dowelhurst Ltd) |
| 5153 | Antimuscarinics | 5287111000001107 | Combivent inhaler (Dowelhurst Ltd) |
| 5153 | Antimuscarinics | 5287711000001108 | Combivent nebuliser liquid 2.5ml UDVs (Dowelhurst Ltd) |
| 5153 | Antimuscarinics | 5297911000001106 | Ipratropium 20micrograms/dose inhaler (Dowelhurst Ltd) |
| 5153 | Antimuscarinics | 5319611000001103 | Spiriva 18microgram inhalation powder capsules with HandiHaler (Dowelhurst Ltd) |
| 5153 | Antimuscarinics | 7389911000001105 | Atrovent 20micrograms/dose inhaler CFC free (Boehringer Ingelheim Ltd) |
| 5153 | Antimuscarinics | 8097311000001109 | Ipratropium bromide 250micrograms/1ml nebuliser liquid unit dose vials (Kent Pharma (UK) Ltd) |
| 5153 | Antimuscarinics | 8097811000001100 | Ipratropium bromide 500micrograms/2ml nebuliser liquid unit dose vials (Kent Pharma (UK) Ltd) |
| 5153 | Antimuscarinics | 9039611000001103 | Ipratropium 250micrograms/1ml nebuliser liquid unit dose Steripoule vials (Galen Ltd) |
| 5153 | Antimuscarinics | 9039811000001104 | Ipratropium 500micrograms/2ml nebuliser liquid unit dose Steripoule vials (Galen Ltd) |
| 5153 | Antimuscarinics | 9101911000001108 | Ipratropium bromide 250micrograms/1ml nebuliser liquid unit dose vials (Teva UK Ltd) |
| 5153 | Antimuscarinics | 9102111000001100 | Ipratropium bromide 500micrograms/2ml nebuliser liquid unit dose vials (Teva UK Ltd) |
| 5153 | Antimuscarinics | 9478911000001107 | Tiotropium bromide 18microgram inhalation powder capsules |
| 5153 | Antimuscarinics | 9479011000001103 | Tiotropium bromide 18microgram inhalation powder capsules with device |
| 5153 | Antimuscarinics | 10453411000001106 | Ipratropium bromide 250micrograms/1ml nebuliser liquid unit dose vials (Arrow Generics Ltd) |
| 5153 | Antimuscarinics | 10453611000001109 | Ipratropium bromide 500micrograms/2ml nebuliser liquid unit dose vials (Arrow Generics Ltd) |
| 5153 | Antimuscarinics | 10469711000001108 | Spiriva 18microgram inhalation powder capsules (Dowelhurst Ltd) |
| 5153 | Antimuscarinics | 10509311000001101 | Spiriva 18microgram inhalation powder capsules with HandiHaler (Waymade Healthcare Plc) |
| 5153 | Antimuscarinics | 10776911000001102 | Combivent nebuliser liquid 2.5ml UDVs (Waymade Healthcare Plc) |
| 5153 | Antimuscarinics | 10785611000001108 | Spiriva 18microgram inhalation powder capsules (Waymade Healthcare Plc) |
| 5153 | Antimuscarinics | 10927511000001104 | Ipramol nebuliser solution 2.5ml Steri-Neb unit dose vials (Teva UK Ltd) |
| 5153 | Antimuscarinics | 11006111000001102 | Ipratropium bromide 250micrograms/1ml nebuliser liquid unit dose vials (Accord Healthcare Ltd) |
| 5153 | Antimuscarinics | 11006311000001100 | Ipratropium bromide 500micrograms/2ml nebuliser liquid unit dose vials (Accord Healthcare Ltd) |
| 5153 | Antimuscarinics | 12146911000001103 | Spiriva Respimat 2.5micrograms/dose inhalation solution cartridge with device (Boehringer Ingelheim Ltd) |
| 5153 | Antimuscarinics | 12197411000001102 | Tiotropium bromide 2.5micrograms/dose solution for inhalation cartridge with device CFC free |
| 5153 | Antimuscarinics | 13163001000001106 | Ipratropium bromide 20micrograms/dose breath actuated inhaler |
| 5153 | Antimuscarinics | 13164501000001104 | Fenoterol 100micrograms/dose / Ipratropium 40micrograms/dose inhaler |
| 5153 | Antimuscarinics | 13164601000001100 | Fenoterol 100micrograms/dose / Ipratropium bromide 40micrograms/dose breath actuated inhaler |
| 5153 | Antimuscarinics | 13164701000001105 | Salbutamol 100micrograms/dose / Ipratropium 20micrograms/dose inhaler |
| 5153 | Antimuscarinics | 13266301000001102 | Ipratropium bromide 20micrograms/dose inhaler CFC free |
| 5153 | Antimuscarinics | 13952411000001104 | Spiriva 18microgram inhalation powder capsules with HandiHaler (DE Pharmaceuticals) |
| 5153 | Antimuscarinics | 13953011000001104 | Spiriva 18microgram inhalation powder capsules (DE Pharmaceuticals) |
| 5153 | Antimuscarinics | 14206211000001105 | Atrovent 20micrograms/dose inhaler CFC free (Sigma Pharmaceuticals Plc) |
| 5153 | Antimuscarinics | 14214011000001107 | Duovent UDVs nebuliser liquid 4ml (Sigma Pharmaceuticals Plc) |
| 5153 | Antimuscarinics | 14382411000001103 | Spiriva 18microgram inhalation powder capsules (Sigma Pharmaceuticals Plc) |
| 5153 | Antimuscarinics | 14384011000001101 | Spiriva 18microgram inhalation powder capsules with HandiHaler (Sigma Pharmaceuticals Plc) |
| 5153 | Antimuscarinics | 15213511000001109 | Ipratropium bromide 250micrograms/1ml nebuliser liquid unit dose vials (Sigma Pharmaceuticals Plc) |
| 5153 | Antimuscarinics | 15214311000001101 | Ipratropium bromide 500micrograms/2ml nebuliser liquid unit dose vials (Sigma Pharmaceuticals Plc) |
| 5153 | Antimuscarinics | 15522411000001103 | Salipraneb 0.5mg/2.5mg nebuliser solution 2.5ml ampoules (Arrow Generics Ltd) |
| 5153 | Antimuscarinics | 15534911000001100 | Salbutamol 2.5mg/2.5ml / Ipratropium bromide 500micrograms/2.5ml nebuliser liquid ampoules |
| 5153 | Antimuscarinics | 16733611000001107 | Salbutamol 2.5mg/2.5ml / Ipratropium bromide 500micrograms/2.5ml nebuliser liquid ampoules (A A H Pharmaceuticals Ltd) |
| 5153 | Antimuscarinics | 17454411000001104 | Spiriva 18microgram inhalation powder capsules with HandiHaler (Mawdsley-Brooks & Company Ltd) |
| 5153 | Antimuscarinics | 17454611000001101 | Spiriva Respimat 2.5micrograms/dose solution for inhalation cartridge with device (Mawdsley-Brooks & Company Ltd) |
| 5153 | Antimuscarinics | 17933111000001105 | Ipratropium bromide 250micrograms/1ml nebuliser liquid unit dose vials (Phoenix Healthcare Distribution Ltd) |
| 5153 | Antimuscarinics | 17933311000001107 | Ipratropium bromide 500micrograms/2ml nebuliser liquid unit dose vials (Phoenix Healthcare Distribution Ltd) |
| 5153 | Antimuscarinics | 18071311000001107 | Atrovent 20micrograms/dose inhaler CFC free (Lexon (UK) Ltd) |
| 5153 | Antimuscarinics | 18155011000001100 | Spiriva 18microgram inhalation powder capsules (Mawdsley-Brooks & Company Ltd) |
| 5153 | Antimuscarinics | 18220811000001104 | Combivent nebuliser liquid 2.5ml UDVs (Mawdsley-Brooks & Company Ltd) |
| 5153 | Antimuscarinics | 19541511000001101 | Combivent nebuliser liquid 2.5ml UDVs (DE Pharmaceuticals) |
| 5153 | Antimuscarinics | 19869411000001109 | Combivent nebuliser liquid 2.5ml UDVs (Lexon (UK) Ltd) |
| 5153 | Antimuscarinics | 20164811000001104 | Atrovent 20micrograms/dose inhaler CFC free (DE Pharmaceuticals) |
| 5153 | Antimuscarinics | 20985511000001101 | Eklira 322micrograms/dose Genuair (AstraZeneca UK Ltd) |
| 5153 | Antimuscarinics | 21021211000001107 | Aclidinium bromide 375micrograms/dose dry powder inhaler |
| 5153 | Antimuscarinics | 21495411000001107 | Seebri Breezhaler 44microgram inhalation powder capsules with device (Novartis Pharmaceuticals UK Ltd) |
| 5153 | Antimuscarinics | 21496211000001102 | Glycopyrronium bromide 55microgram inhalation powder capsules with device |
| 5153 | Antimuscarinics | 21508411000001109 | Atrovent 500micrograms/2ml nebuliser liquid UDVs (Waymade Healthcare Plc) |
| 5153 | Antimuscarinics | 21719511000001100 | Ipratropium bromide 250micrograms/1ml nebuliser liquid unit dose vials (Advanz Pharma) |
| 5153 | Antimuscarinics | 21720011000001109 | Ipratropium bromide 500micrograms/2ml nebuliser liquid unit dose vials (Advanz Pharma) |
| 5153 | Antimuscarinics | 22062311000001100 | Ipratropium bromide 250micrograms/1ml nebuliser liquid unit dose vials (Waymade Healthcare Plc) |
| 5153 | Antimuscarinics | 22062511000001106 | Ipratropium bromide 500micrograms/2ml nebuliser liquid unit dose vials (Waymade Healthcare Plc) |
| 5153 | Antimuscarinics | 23433411000001108 | Salbutamol 2.5mg/2.5ml / Ipratropium bromide 500micrograms/2.5ml nebuliser liquid unit dose vials (J M McGill Ltd) |
| 5153 | Antimuscarinics | 23949111000001102 | Ipratropium bromide 250micrograms/1ml nebuliser liquid unit dose vials (DE Pharmaceuticals) |
| 5153 | Antimuscarinics | 23949311000001100 | Ipratropium bromide 500micrograms/2ml nebuliser liquid unit dose vials (DE Pharmaceuticals) |
| 5153 | Antimuscarinics | 23961011000001108 | Salbutamol 2.5mg/2.5ml / Ipratropium bromide 500micrograms/2.5ml nebuliser liquid unit dose vials (Niche Pharma Ltd) |
| 5153 | Antimuscarinics | 24498211000001109 | Spiriva Respimat 2.5micrograms/dose solution for inhalation cartridge with device (Waymade Healthcare Plc) |
| 5153 | Antimuscarinics | 24644611000001108 | Anoro Ellipta 55micrograms/dose / 22micrograms/dose dry powder inhaler (GlaxoSmithKline UK Ltd) |
| 5153 | Antimuscarinics | 24645511000001105 | Umeclidinium bromide 65micrograms/dose / Vilanterol 22micrograms/dose dry powder inhaler |
| 5153 | Antimuscarinics | 27567911000001101 | Incruse Ellipta 55micrograms/dose dry powder inhaler (GlaxoSmithKline UK Ltd) |
| 5153 | Antimuscarinics | 27872911000001109 | Ipratropium bromide 20micrograms/dose inhaler CFC free (A A H Pharmaceuticals Ltd) |
| 5153 | Antimuscarinics | 27890611000001109 | Umeclidinium bromide 65micrograms/dose dry powder inhaler |
| 5153 | Antimuscarinics | 28007211000001102 | Ultibro Breezhaler 85microgram/43microgram inhalation powder capsules with device (Novartis Pharmaceuticals UK Ltd) |
| 5153 | Antimuscarinics | 28049611000001104 | Indacaterol 85micrograms/dose / Glycopyrronium bromide 54micrograms/dose inhalation powder capsules with device |
| 5153 | Antimuscarinics | 28357211000001106 | Duaklir 340micrograms/dose / 12micrograms/dose Genuair (AstraZeneca UK Ltd) |
| 5153 | Antimuscarinics | 28365011000001100 | Aclidinium bromide 396micrograms/dose / Formoterol 11.8micrograms/dose dry powder inhaler |
| 5153 | Antimuscarinics | 28409511000001104 | Tiotropium bromide 2.5micrograms/dose solution for inhalation cartridge with device CFC free (AM Distributions (Yorkshire) Ltd) |
| 5153 | Antimuscarinics | 28410811000001107 | Berodual N inhaler (Imported (Germany)) |
| 5153 | Antimuscarinics | 28422511000001101 | Fenoterol 50micrograms/dose / Ipratropium bromide 20micrograms/dose inhaler CFC free |
| 5153 | Antimuscarinics | 28424911000001104 | Eklira 322micrograms/dose Genuair (Waymade Healthcare Plc) |
| 5153 | Antimuscarinics | 29211411000001103 | Salbutamol 2.5mg/2.5ml / Ipratropium bromide 500micrograms/2.5ml nebuliser liquid unit dose vials (Ennogen Healthcare Ltd) |
| 5153 | Antimuscarinics | 29971311000001100 | Spiolto Respimat 2.5micrograms/dose / 2.5micrograms/dose inhalation solution cartridge with device (Boehringer Ingelheim Ltd) |
| 5153 | Antimuscarinics | 29978611000001102 | Atrovent 250micrograms/1ml nebuliser liquid UDVs (Lexon (UK) Ltd) |
| 5153 | Antimuscarinics | 29978911000001108 | Atrovent 500micrograms/2ml nebuliser liquid UDVs (Lexon (UK) Ltd) |
| 5153 | Antimuscarinics | 29987211000001108 | Tiotropium bromide 2.5micrograms/dose / Olodaterol 2.5micrograms/dose solution for inhalation cartridge with device CFC free |
| 5153 | Antimuscarinics | 30215011000001107 | Ipratropium bromide 500micrograms/2ml nebuliser liquid unit dose vials (Mawdsley-Brooks & Company Ltd) |
| 5153 | Antimuscarinics | 30273611000001108 | Seebri Breezhaler 44microgram inhalation powder capsules with device (Waymade Healthcare Plc) |
| 5153 | Antimuscarinics | 30933811000001108 | Glycopyrronium bromide 55microgram inhalation powder capsules with device (Ennogen Healthcare Ltd) |
| 5153 | Antimuscarinics | 31451611000001103 | Aclidinium bromide 375micrograms/dose dry powder inhaler (J M McGill Ltd) |
| 5153 | Antimuscarinics | 31500911000001102 | Glycopyrronium bromide 55microgram inhalation powder capsules with device (J M McGill Ltd) |
| 5153 | Antimuscarinics | 32180811000001101 | Aclidinium bromide 375micrograms/dose dry powder inhaler (Niche Pharma Ltd) |
| 5153 | Antimuscarinics | 32336011000001106 | Glycopyrronium bromide 55microgram inhalation powder capsules with device (Colorama Pharmaceuticals Ltd) |
| 5153 | Antimuscarinics | 32364511000001104 | Umeclidinium bromide 65micrograms/dose / Vilanterol 22micrograms/dose dry powder inhaler (Ennogen Healthcare Ltd) |
| 5153 | Antimuscarinics | 32408711000001106 | Ipratropium bromide 20micrograms/dose inhaler CFC free (Kent Pharmaceuticals Ltd) |
| 5153 | Antimuscarinics | 32411611000001108 | Aclidinium bromide 375micrograms/dose dry powder inhaler (Colorama Pharmaceuticals Ltd) |
| 5153 | Antimuscarinics | 32480911000001107 | Salbutamol 2.5mg/2.5ml / Ipratropium bromide 500micrograms/2.5ml nebuliser liquid unit dose vials (Colorama Pharmaceuticals Ltd) |
| 5153 | Antimuscarinics | 32629811000001101 | Umeclidinium bromide 65micrograms/dose / Vilanterol 22micrograms/dose dry powder inhaler (J M McGill Ltd) |
| 5153 | Antimuscarinics | 32633011000001106 | Aclidinium bromide 396micrograms/dose / Formoterol 11.8micrograms/dose dry powder inhaler (Colorama Pharmaceuticals Ltd) |
| 5153 | Antimuscarinics | 32898611000001109 | Salipraneb 0.5mg/2.5mg nebuliser solution 2.5ml ampoules (Actavis UK Ltd) |
| 5153 | Antimuscarinics | 33594911000001100 | Braltus 10microgram inhalation powder capsules with Zonda inhaler (Teva UK Ltd) |
| 5153 | Antimuscarinics | 33596311000001107 | Tiotropium bromide 10microgram inhalation powder capsules with device |
| 5153 | Antimuscarinics | 34681611000001100 | Trimbow 87micrograms/dose / 5micrograms/dose / 9micrograms/dose inhaler (Chiesi Ltd) |
| 5153 | Antimuscarinics | 34683311000001106 | Generic Trimbow 87micrograms/dose / 5micrograms/dose / 9micrograms/dose inhaler |
| 5153 | Antimuscarinics | 34952211000001104 | Trelegy Ellipta 92micrograms/dose / 55micrograms/dose / 22micrograms/dose dry powder inhaler (GlaxoSmithKline UK Ltd) |
| 5153 | Antimuscarinics | 34954811000001109 | Inhalvent 20micrograms/dose inhaler (Alissa Healthcare Research Ltd) |
| 5153 | Antimuscarinics | 34955111000001103 | Generic Trelegy Ellipta 92micrograms/dose / 55micrograms/dose / 22micrograms/dose dry powder inhaler |
| 5153 | Antimuscarinics | 35894111000001105 | Aclidinium bromide 375micrograms/dose dry powder inhaler |
| 5153 | Antimuscarinics | 35936311000001102 | Salbutamol 100micrograms/dose / Ipratropium 20micrograms/dose inhaler |
| 5153 | Antimuscarinics | 36049211000001106 | Ipratropium bromide 20micrograms/dose breath actuated inhaler |
| 5153 | Antimuscarinics | 36049311000001103 | Ipratropium bromide 20micrograms/dose inhaler |
| 5153 | Antimuscarinics | 36049411000001105 | Ipratropium bromide 40micrograms/dose inhaler |
| 5153 | Antimuscarinics | 36066611000001106 | Fenoterol 100micrograms/dose / Ipratropium 40micrograms/dose inhaler |
| 5153 | Antimuscarinics | 36066711000001102 | Fenoterol 100micrograms/dose / Ipratropium bromide 40micrograms/dose breath actuated inhaler |
| 5153 | Antimuscarinics | 37344011000001102 | Atrovent 20micrograms/dose inhaler CFC free (Mawdsley-Brooks & Company Ltd) |
| 5153 | Antimuscarinics | 37344411000001106 | Atrovent 250micrograms/1ml nebuliser liquid UDVs (Mawdsley-Brooks & Company Ltd) |
| 5153 | Antimuscarinics | 37344911000001103 | Atrovent 500micrograms/2ml nebuliser liquid UDVs (Mawdsley-Brooks & Company Ltd) |
| 5153 | Antimuscarinics | 37364411000001100 | Combivent nebuliser liquid 2.5ml UDVs (CST Pharma Ltd) |
| 5153 | Antimuscarinics | 37394911000001105 | Spiriva 18microgram inhalation powder capsules (CST Pharma Ltd) |
| 5153 | Antimuscarinics | 37399011000001107 | Eklira 322micrograms/dose Genuair (Mawdsley-Brooks & Company Ltd) |
| 5153 | Antimuscarinics | 37439611000001101 | Incruse Ellipta 55micrograms/dose dry powder inhaler (CST Pharma Ltd) |
| 5153 | Antimuscarinics | 37511511000001102 | Incruse Ellipta 55micrograms/dose dry powder inhaler (Mawdsley-Brooks & Company Ltd) |
| 5153 | Antimuscarinics | 37540311000001105 | Yanimo Respimat 2.5micrograms/dose / 2.5micrograms/dose inhalation solution cartridge with device (Boehringer Ingelheim Ltd) |
| 5153 | Antimuscarinics | 37593111000001108 | Seebri Breezhaler 44microgram inhalation powder capsules with device (CST Pharma Ltd) |
| 5153 | Antimuscarinics | 37596311000001101 | Atrovent 20micrograms/dose inhaler CFC free (CST Pharma Ltd) |
| 5153 | Antimuscarinics | 37625611000001100 | Combivent nebuliser liquid 2.5ml UDVs (Pilsco Ltd) |
| 5153 | Antimuscarinics | 37633411000001104 | Eklira 322micrograms/dose Genuair (Pilsco Ltd) |
| 5153 | Antimuscarinics | 37666611000001102 | Incruse Ellipta 55micrograms/dose dry powder inhaler (Ethigen Ltd) |
| 5153 | Antimuscarinics | 37677711000001102 | Spiriva Respimat 2.5micrograms/dose inhalation solution refill cartridge (Boehringer Ingelheim Ltd) |
| 5153 | Antimuscarinics | 37678011000001103 | Spiolto Respimat 2.5micrograms/dose / 2.5micrograms/dose inhalation solution refill cartridge (Boehringer Ingelheim Ltd) |
| 5153 | Antimuscarinics | 37692511000001100 | Tiotropium bromide 2.5micrograms/dose / Olodaterol 2.5micrograms/dose inhalation solution cartridge CFC free |
| 5153 | Antimuscarinics | 37692711000001105 | Tiotropium bromide 2.5micrograms/dose inhalation solution cartridge CFC free |
| 5153 | Antimuscarinics | 37716011000001104 | Braltus 10microgram inhalation powder capsules with Zonda inhaler (Pilsco Ltd) |
| 5153 | Antimuscarinics | 37728211000001103 | Seebri Breezhaler 44microgram inhalation powder capsules with device (Pilsco Ltd) |
| 5153 | Antimuscarinics | 37730011000001109 | Spiriva 18microgram inhalation powder capsules with HandiHaler (Pilsco Ltd) |
| 5153 | Antimuscarinics | 37743311000001100 | Spiriva 18microgram inhalation powder capsules with HandiHaler (CST Pharma Ltd) |
| 5153 | Antimuscarinics | 37743711000001101 | Braltus 10microgram inhalation powder capsules with Zonda inhaler (CST Pharma Ltd) |
| 5153 | Antimuscarinics | 37842911000001109 | Ipravent 20micrograms/dose inhaler CFC free (Cipla EU Ltd) |
| 5153 | Antimuscarinics | 37979411000001103 | Anoro Ellipta 55micrograms/dose / 22micrograms/dose dry powder inhaler (Pharmaram Ltd) |
| 5153 | Antimuscarinics | 37989411000001108 | Incruse Ellipta 55micrograms/dose dry powder inhaler (Pharmaram Ltd) |
| 5153 | Antimuscarinics | 37996311000001109 | Seebri Breezhaler 44microgram inhalation powder capsules with device (Pharmaram Ltd) |
| 5153 | Antimuscarinics | 38120811000001104 | Braltus 10microgram inhalation powder capsules with Zonda inhaler (DE Pharmaceuticals) |
| 5153 | Antimuscarinics | 38131811000001105 | Eklira 322micrograms/dose Genuair (DE Pharmaceuticals) |
| 5153 | Antimuscarinics | 38140511000001104 | Incruse Ellipta 55micrograms/dose dry powder inhaler (DE Pharmaceuticals) |
| 5153 | Antimuscarinics | 38617811000001107 | Combiprasal 0.5mg/2.5mg nebuliser solution 2.5ml unit dose vials (TriOn Pharma Ltd) |
| 5153 | Antimuscarinics | 38893611000001108 | Aclidinium bromide 375micrograms/dose dry powder inhaler |
| 5153 | Antimuscarinics | 38894511000001107 | Ipratropium bromide 20micrograms/dose inhaler CFC free |
| 5153 | Antimuscarinics | 39001311000001101 | Ipratropium bromide 250micrograms/1ml nebuliser liquid unit dose vials (Medihealth (Northern) Ltd) |
| 5153 | Antimuscarinics | 39001511000001107 | Ipratropium bromide 500micrograms/2ml nebuliser liquid unit dose vials (Medihealth (Northern) Ltd) |
| 5153 | Antimuscarinics | 39134511000001107 | Generic Enerzair Breezhaler 114micrograms/dose / 46micrograms/dose / 136micrograms/dose inhalation powder capsules with device |
| 5153 | Antimuscarinics | 39134711000001102 | Enerzair Breezhaler 114micrograms/dose / 46micrograms/dose / 136micrograms/dose inhalation powder capsules with device (Sandoz Ltd) |
| 5153 | Antimuscarinics | 39167711000001100 | Rolufta Ellipta 55micrograms/dose dry powder inhaler (Ethigen Ltd) |
| 5153 | Antimuscarinics | 39327311000001104 | Trixeo Aerosphere 5micrograms/dose / 7.2micrograms/dose / 160micrograms/dose pressurised inhaler (AstraZeneca UK Ltd) |
| 5153 | Antimuscarinics | 39329111000001107 | Bevespi Aerosphere 7.2micrograms/dose / 5micrograms/dose pressurised inhaler (AstraZeneca UK Ltd) |
| 5153 | Antimuscarinics | 39343511000001103 | Generic Trixeo Aerosphere 5micrograms/dose / 7.2micrograms/dose / 160micrograms/dose inhaler CFC free |
| 5153 | Antimuscarinics | 39343611000001104 | Glycopyrronium 7.2micrograms/dose / Formoterol 5micrograms/dose inhaler CFC free |
| 5153 | Antimuscarinics | 39408811000001105 | Spiriva 18microgram inhalation powder capsules (Pilsco Ltd) |
| 5153 | Antimuscarinics | 39605711000001106 | Tiogiva 18microgram inhalation powder capsules with device (Glenmark Pharmaceuticals Europe Ltd) |
| 5153 | Antimuscarinics | 39606211000001105 | Tiogiva 18microgram inhalation powder capsules (Glenmark Pharmaceuticals Europe Ltd) |
| 5153 | Antimuscarinics | 39666311000001103 | Acopair 18microgram inhalation powder capsules with NeumoHaler (Viatris UK Healthcare Ltd) |
| 5153 | Antimuscarinics | 39710511000001100 | Salbutamol 2.5mg/2.5ml / Ipratropium bromide 500micrograms/2.5ml nebuliser liquid unit dose vials |
| 5153 | Antimuscarinics | 39711511000001107 | Fenoterol 1.25mg/4ml / Ipratropium 500micrograms/4ml nebuliser liquid unit dose vials |
| 5153 | Antimuscarinics | 39717211000001104 | Ipratropium bromide 250micrograms/1ml nebuliser liquid unit dose vials |
| 5153 | Antimuscarinics | 39717311000001107 | Ipratropium bromide 40microgram inhalation powder capsules with device |
| 5153 | Antimuscarinics | 39717411000001100 | Ipratropium bromide 40microgram inhalation powder capsules |
| 5153 | Antimuscarinics | 39717511000001101 | Ipratropium bromide 500micrograms/2ml nebuliser liquid unit dose vials |
| 5153 | Antimuscarinics | 39993311000001105 | Trimbow NEXThaler 88micrograms/dose / 5micrograms/dose / 9micrograms/dose dry powder inhaler (Chiesi Ltd) |
| 5153 | Antimuscarinics | 40087411000001104 | Generic Trimbow NEXThaler 88micrograms/dose / 5micrograms/dose / 9micrograms/dose dry powder inhaler |
| 5153 | Antimuscarinics | 40478511000001100 | Seebri Breezhaler 44microgram inhalation powder capsules with device (DE Pharmaceuticals) |
| 5153 | Antimuscarinics | 40752211000001109 | Trimbow 172micrograms/dose / 5micrograms/dose / 9micrograms/dose inhaler (Chiesi Ltd) |
| 5153 | Antimuscarinics | 40766811000001104 | Generic Trimbow 172micrograms/dose / 5micrograms/dose / 9micrograms/dose inhaler |
| 5154 | Beta2AdrenoceptorAgonistsSelective | 3417111000001102 | Salbutamol 100micrograms/dose inhaler (Alliance Healthcare (Distribution) Ltd) |
| 5154 | Beta2AdrenoceptorAgonistsSelective | 3633911000001104 | Bricanyl 5mg/2ml Respules (AstraZeneca UK Ltd) |
| 5154 | Beta2AdrenoceptorAgonistsSelective | 3654411000001106 | Terbutaline 5mg/2ml nebuliser liquid unit dose vials |
| 5154 | Beta2AdrenoceptorAgonistsSelective | 3926911000001108 | Ventolin 500micrograms/1ml solution for injection ampoules (GlaxoSmithKline UK Ltd) |
| 5154 | Beta2AdrenoceptorAgonistsSelective | 4179611000001107 | Aerocrom Syncroner with spacer (Castlemead Healthcare Ltd) |
| 5154 | Beta2AdrenoceptorAgonistsSelective | 4192411000001109 | Duovent UDVs nebuliser liquid 4ml (Boehringer Ingelheim Ltd) |
| 5154 | Beta2AdrenoceptorAgonistsSelective | 4373811000001100 | Symbicort 400/12 Turbohaler (AstraZeneca UK Ltd) |
| 5154 | Beta2AdrenoceptorAgonistsSelective | 4378111000001103 | Budesonide 400micrograms/dose / Formoterol 12micrograms/dose dry powder inhaler |
| 5154 | Beta2AdrenoceptorAgonistsSelective | 4537611000001107 | Berotec 200micrograms/dose inhaler (Boehringer Ingelheim Ltd) |
| 5154 | Beta2AdrenoceptorAgonistsSelective | 4558411000001107 | Fenoterol 200micrograms/dose inhaler |
| 5154 | Beta2AdrenoceptorAgonistsSelective | 4843911000001107 | Ventolin 5mg/ml respirator solution (GlaxoSmithKline UK Ltd) |
| 5154 | Beta2AdrenoceptorAgonistsSelective | 4889111000001103 | Salbutamol 5mg/50ml solution for infusion vials (Martindale Pharmaceuticals Ltd) |
| 5154 | Beta2AdrenoceptorAgonistsSelective | 5257611000001105 | Bricanyl 250micrograms/dose inhaler (Waymade Healthcare Plc) |
| 5154 | Beta2AdrenoceptorAgonistsSelective | 5257911000001104 | Bricanyl 500micrograms/dose Turbohaler (Waymade Healthcare Plc) |
| 5154 | Beta2AdrenoceptorAgonistsSelective | 5259111000001108 | Combivent inhaler (Waymade Healthcare Plc) |
| 5154 | Beta2AdrenoceptorAgonistsSelective | 5271811000001100 | Oxis 12 Turbohaler (Waymade Healthcare Plc) |
| 5154 | Beta2AdrenoceptorAgonistsSelective | 5272211000001108 | Oxis 6 Turbohaler (Waymade Healthcare Plc) |
| 5154 | Beta2AdrenoceptorAgonistsSelective | 5275011000001108 | Salmeterol 25micrograms/dose inhaler (Waymade Healthcare Plc) |
| 5154 | Beta2AdrenoceptorAgonistsSelective | 5275311000001106 | Serevent 25micrograms/dose inhaler (Waymade Healthcare Plc) |
| 5154 | Beta2AdrenoceptorAgonistsSelective | 5275611000001101 | Seretide 100 Accuhaler (Waymade Healthcare Plc) |
| 5154 | Beta2AdrenoceptorAgonistsSelective | 5276011000001104 | Seretide 250 Accuhaler (Waymade Healthcare Plc) |
| 5154 | Beta2AdrenoceptorAgonistsSelective | 5276211000001109 | Seretide 500 Accuhaler (Waymade Healthcare Plc) |
| 5154 | Beta2AdrenoceptorAgonistsSelective | 5276511000001107 | Serevent 50micrograms/dose Accuhaler (Waymade Healthcare Plc) |
| 5154 | Beta2AdrenoceptorAgonistsSelective | 5276811000001105 | Seretide 250 Evohaler (Waymade Healthcare Plc) |
| 5154 | Beta2AdrenoceptorAgonistsSelective | 5277211000001106 | Symbicort 200/6 Turbohaler (Waymade Healthcare Plc) |
| 5154 | Beta2AdrenoceptorAgonistsSelective | 5277411000001105 | Terbutaline 250micrograms/dose inhaler (Waymade Healthcare Plc) |
| 5154 | Beta2AdrenoceptorAgonistsSelective | 5277911000001102 | Ventolin 100micrograms/dose Evohaler (Waymade Healthcare Plc) |
| 5154 | Beta2AdrenoceptorAgonistsSelective | 5278711000001103 | Ventide inhaler (Waymade Healthcare Plc) |
| 5154 | Beta2AdrenoceptorAgonistsSelective | 5284911000001104 | Bricanyl 250micrograms/dose inhaler (Dowelhurst Ltd) |
| 5154 | Beta2AdrenoceptorAgonistsSelective | 5285911000001100 | Bricanyl 500micrograms/dose Turbohaler (Dowelhurst Ltd) |
| 5154 | Beta2AdrenoceptorAgonistsSelective | 5287111000001107 | Combivent inhaler (Dowelhurst Ltd) |
| 5154 | Beta2AdrenoceptorAgonistsSelective | 5287711000001108 | Combivent nebuliser liquid 2.5ml UDVs (Dowelhurst Ltd) |
| 5154 | Beta2AdrenoceptorAgonistsSelective | 5307111000001103 | Oxis 12 Turbohaler (Dowelhurst Ltd) |
| 5154 | Beta2AdrenoceptorAgonistsSelective | 5307711000001102 | Oxis 6 Turbohaler (Dowelhurst Ltd) |
| 5154 | Beta2AdrenoceptorAgonistsSelective | 5315611000001101 | Salmeterol 25micrograms/dose inhaler (Dowelhurst Ltd) |
| 5154 | Beta2AdrenoceptorAgonistsSelective | 5317111000001100 | Seretide 100 Accuhaler (Dowelhurst Ltd) |
| 5154 | Beta2AdrenoceptorAgonistsSelective | 5317311000001103 | Seretide 250 Accuhaler (Dowelhurst Ltd) |
| 5154 | Beta2AdrenoceptorAgonistsSelective | 5317511000001109 | Seretide 500 Accuhaler (Dowelhurst Ltd) |
| 5154 | Beta2AdrenoceptorAgonistsSelective | 5320411000001102 | Serevent 25micrograms/dose inhaler (Dowelhurst Ltd) |
| 5154 | Beta2AdrenoceptorAgonistsSelective | 5320611000001104 | Serevent 50micrograms/dose Accuhaler (Dowelhurst Ltd) |
| 5154 | Beta2AdrenoceptorAgonistsSelective | 5321011000001102 | Symbicort 200/6 Turbohaler (Dowelhurst Ltd) |
| 5154 | Beta2AdrenoceptorAgonistsSelective | 5321111000001101 | Serevent 50microgram disks with Diskhaler (Dowelhurst Ltd) |
| 5154 | Beta2AdrenoceptorAgonistsSelective | 5321611000001109 | Serevent 50microgram disks (Dowelhurst Ltd) |
| 5154 | Beta2AdrenoceptorAgonistsSelective | 5322811000001104 | Terbutaline 250micrograms/dose inhaler (Dowelhurst Ltd) |
| 5154 | Beta2AdrenoceptorAgonistsSelective | 5323111000001100 | Terbutaline 500micrograms/dose Turbohaler (Dowelhurst Ltd) |
| 5154 | Beta2AdrenoceptorAgonistsSelective | 5323811000001107 | Salmeterol 50microgram Diskhaler (Dowelhurst Ltd) |
| 5154 | Beta2AdrenoceptorAgonistsSelective | 5324211000001109 | Ventide inhaler (Dowelhurst Ltd) |
| 5154 | Beta2AdrenoceptorAgonistsSelective | 5324411000001108 | Ventolin 200micrograms/dose Accuhaler (Dowelhurst Ltd) |
| 5154 | Beta2AdrenoceptorAgonistsSelective | 5324811000001105 | Ventolin 200microgram Rotacaps (Dowelhurst Ltd) |
| 5154 | Beta2AdrenoceptorAgonistsSelective | 5411211000001107 | Salbutamol 8mg modified-release tablets (Waymade Healthcare Plc) |
| 5154 | Beta2AdrenoceptorAgonistsSelective | 5420011000001102 | Salbutamol 4mg modified-release tablets (Waymade Healthcare Plc) |
| 5154 | Beta2AdrenoceptorAgonistsSelective | 5443411000001101 | Volmax 4mg modified-release tablets (Dowelhurst Ltd) |
| 5154 | Beta2AdrenoceptorAgonistsSelective | 5443711000001107 | Volmax 8mg modified-release tablets (Dowelhurst Ltd) |
| 5154 | Beta2AdrenoceptorAgonistsSelective | 5583011000001107 | Ventolin 400microgram Rotacaps (Dowelhurst Ltd) |
| 5154 | Beta2AdrenoceptorAgonistsSelective | 5594511000001100 | Ventolin 100micrograms/dose Evohaler (Dowelhurst Ltd) |
| 5154 | Beta2AdrenoceptorAgonistsSelective | 9040011000001103 | Salbutamol 2.5mg/2.5ml nebuliser liquid unit dose Steripoule vials (Galen Ltd) |
| 5154 | Beta2AdrenoceptorAgonistsSelective | 9040211000001108 | Salbutamol 5mg/2.5ml nebuliser liquid unit dose Steripoule vials (Galen Ltd) |
| 5154 | Beta2AdrenoceptorAgonistsSelective | 9102311000001103 | Salbutamol 2.5mg/2.5ml nebuliser liquid unit dose vials (Teva UK Ltd) |
| 5154 | Beta2AdrenoceptorAgonistsSelective | 9102511000001109 | Salbutamol 5mg/2.5ml nebuliser liquid unit dose vials (Teva UK Ltd) |
| 5154 | Beta2AdrenoceptorAgonistsSelective | 9111011000001106 | Terbutaline 5mg/2ml nebuliser liquid unit dose vials (Galen Ltd) |
| 5154 | Beta2AdrenoceptorAgonistsSelective | 9204911000001109 | Easyhaler Salbutamol sulfate 200micrograms/dose dry powder inhaler (Orion Pharma (UK) Ltd) |
| 5154 | Beta2AdrenoceptorAgonistsSelective | 9205211000001104 | Easyhaler Salbutamol sulfate 100micrograms/dose dry powder inhaler (Orion Pharma (UK) Ltd) |
| 5154 | Beta2AdrenoceptorAgonistsSelective | 9207411000001106 | Salbutamol 100micrograms/dose dry powder inhaler |
| 5154 | Beta2AdrenoceptorAgonistsSelective | 9239411000001108 | Terbutaline 1.5mg/5ml oral solution sugar free (A A H Pharmaceuticals Ltd) |
| 5154 | Beta2AdrenoceptorAgonistsSelective | 9628711000001106 | Atimos Modulite 12micrograms/dose inhaler (Chiesi Ltd) |
| 5154 | Beta2AdrenoceptorAgonistsSelective | 9652711000001107 | Formoterol 12micrograms/dose inhaler CFC free |
| 5154 | Beta2AdrenoceptorAgonistsSelective | 10073011000001107 | Serevent 25micrograms/dose Evohaler (GlaxoSmithKline UK Ltd) |
| 5154 | Beta2AdrenoceptorAgonistsSelective | 10075611000001101 | Salmeterol 25micrograms/dose inhaler CFC free |
| 5154 | Beta2AdrenoceptorAgonistsSelective | 10432311000001108 | Salbutamol 100micrograms/dose inhaler (Arrow Generics Ltd) |
| 5154 | Beta2AdrenoceptorAgonistsSelective | 10453811000001108 | Salbutamol 2.5mg/2.5ml nebuliser liquid unit dose vials (Arrow Generics Ltd) |
| 5154 | Beta2AdrenoceptorAgonistsSelective | 10454011000001100 | Salbutamol 5mg/2.5ml nebuliser liquid unit dose vials (Arrow Generics Ltd) |
| 5154 | Beta2AdrenoceptorAgonistsSelective | 10472811000001102 | Seretide 250 Evohaler (Dowelhurst Ltd) |
| 5154 | Beta2AdrenoceptorAgonistsSelective | 10473211000001109 | Seretide 125 Evohaler (Dowelhurst Ltd) |
| 5154 | Beta2AdrenoceptorAgonistsSelective | 10474211000001107 | Salbutamol 4mg modified-release tablets (Dowelhurst Ltd) |
| 5154 | Beta2AdrenoceptorAgonistsSelective | 10474711000001100 | Salbutamol 8mg modified-release tablets (Dowelhurst Ltd) |
| 5154 | Beta2AdrenoceptorAgonistsSelective | 10493811000001104 | Ventolin 200micrograms/dose Accuhaler (Waymade Healthcare Plc) |
| 5154 | Beta2AdrenoceptorAgonistsSelective | 10506611000001103 | Symbicort 400/12 Turbohaler (Waymade Healthcare Plc) |
| 5154 | Beta2AdrenoceptorAgonistsSelective | 10512011000001109 | Seretide 125 Evohaler (Waymade Healthcare Plc) |
| 5154 | Beta2AdrenoceptorAgonistsSelective | 10776911000001102 | Combivent nebuliser liquid 2.5ml UDVs (Waymade Healthcare Plc) |
| 5154 | Beta2AdrenoceptorAgonistsSelective | 10847711000001102 | Serevent 25micrograms/dose Evohaler (Waymade Healthcare Plc) |
| 5154 | Beta2AdrenoceptorAgonistsSelective | 10927511000001104 | Ipramol nebuliser solution 2.5ml Steri-Neb unit dose vials (Teva UK Ltd) |
| 5154 | Beta2AdrenoceptorAgonistsSelective | 10983311000001107 | Symbicort 100/6 Turbohaler (Waymade Healthcare Plc) |
| 5154 | Beta2AdrenoceptorAgonistsSelective | 11006511000001106 | Salbutamol 2.5mg/2.5ml nebuliser liquid unit dose vials (Accord Healthcare Ltd) |
| 5154 | Beta2AdrenoceptorAgonistsSelective | 11006711000001101 | Salbutamol 5mg/2.5ml nebuliser liquid unit dose vials (Accord Healthcare Ltd) |
| 5154 | Beta2AdrenoceptorAgonistsSelective | 11150411000001106 | Terbutaline 5mg/2ml nebuliser liquid unit dose vials (Accord Healthcare Ltd) |
| 5154 | Beta2AdrenoceptorAgonistsSelective | 11176411000001106 | Formoterol Easyhaler 12micrograms/dose dry powder inhaler (Orion Pharma (UK) Ltd) |
| 5154 | Beta2AdrenoceptorAgonistsSelective | 11256611000001108 | Terbutaline 5mg/2ml nebuliser liquid unit dose vials (A A H Pharmaceuticals Ltd) |
| 5154 | Beta2AdrenoceptorAgonistsSelective | 12062211000001109 | Terbutaline 5mg/2ml nebuliser liquid unit dose vials (Alliance Healthcare (Distribution) Ltd) |
| 5154 | Beta2AdrenoceptorAgonistsSelective | 12626211000001108 | Formoterol 12microgram inhalation powder capsules with device |
| 5154 | Beta2AdrenoceptorAgonistsSelective | 12880611000001109 | Salbutamol 2.5mg/2.5ml nebuliser liquid unit dose vials (Kent Pharma (UK) Ltd) |
| 5154 | Beta2AdrenoceptorAgonistsSelective | 12880811000001108 | Salbutamol 5mg/2.5ml nebuliser liquid unit dose vials (Kent Pharma (UK) Ltd) |
| 5154 | Beta2AdrenoceptorAgonistsSelective | 12906411000001100 | Fostair 100micrograms/dose / 6micrograms/dose inhaler (Chiesi Ltd) |
| 5154 | Beta2AdrenoceptorAgonistsSelective | 12911011000001100 | Beclometasone 100micrograms/dose / Formoterol 6micrograms/dose inhaler CFC free |
| 5154 | Beta2AdrenoceptorAgonistsSelective | 13132801000001101 | Budesonide 200micrograms/dose / Formoterol 6micrograms/dose dry powder inhaler |
| 5154 | Beta2AdrenoceptorAgonistsSelective | 13159001000001101 | Salbutamol 4mg modified-release tablets |
| 5154 | Beta2AdrenoceptorAgonistsSelective | 13159101000001100 | Salbutamol 4mg modified-release capsules |
| 5154 | Beta2AdrenoceptorAgonistsSelective | 13159201000001107 | Salbutamol 8mg modified-release capsules |
| 5154 | Beta2AdrenoceptorAgonistsSelective | 13159401000001106 | Salbutamol 2mg/5ml oral solution sugar free |
| 5154 | Beta2AdrenoceptorAgonistsSelective | 13159601000001109 | Salbutamol 500micrograms/1ml solution for injection ampoules |
| 5154 | Beta2AdrenoceptorAgonistsSelective | 13159701000001104 | Salbutamol 5mg/5ml solution for infusion ampoules |
| 5154 | Beta2AdrenoceptorAgonistsSelective | 13159801000001108 | Salbutamol 200microgram inhalation powder blisters with device |
| 5154 | Beta2AdrenoceptorAgonistsSelective | 13159901000001102 | Salbutamol 400microgram inhalation powder blisters with device |
| 5154 | Beta2AdrenoceptorAgonistsSelective | 13160001000001100 | Salbutamol 200microgram inhalation powder blisters |
| 5154 | Beta2AdrenoceptorAgonistsSelective | 13160101000001104 | Salbutamol 400microgram inhalation powder blisters |
| 5154 | Beta2AdrenoceptorAgonistsSelective | 13160301000001102 | Salbutamol 100micrograms/dose inhaler CFC free |
| 5154 | Beta2AdrenoceptorAgonistsSelective | 13160401000001105 | Salbutamol 200micrograms/dose dry powder inhaler |
| 5154 | Beta2AdrenoceptorAgonistsSelective | 13160601000001108 | Salbutamol 100micrograms/dose breath actuated inhaler CFC free |
| 5154 | Beta2AdrenoceptorAgonistsSelective | 13160701000001103 | Salbutamol 100micrograms/dose inhaler |
| 5154 | Beta2AdrenoceptorAgonistsSelective | 13161101000001106 | Terbutaline 250micrograms/dose inhaler |
| 5154 | Beta2AdrenoceptorAgonistsSelective | 13161201000001104 | Terbutaline 250micrograms/dose inhaler with spacer |
| 5154 | Beta2AdrenoceptorAgonistsSelective | 13161501000001101 | Salmeterol 25micrograms/dose inhaler |
| 5154 | Beta2AdrenoceptorAgonistsSelective | 13161601000001102 | Salmeterol 50microgram inhalation powder blisters with device |
| 5154 | Beta2AdrenoceptorAgonistsSelective | 13161701000001107 | Salmeterol 50microgram inhalation powder blisters |
| 5154 | Beta2AdrenoceptorAgonistsSelective | 13161801000001103 | Salmeterol 50micrograms/dose dry powder inhaler |
| 5154 | Beta2AdrenoceptorAgonistsSelective | 13161901000001109 | Formoterol 6micrograms/dose dry powder inhaler |
| 5154 | Beta2AdrenoceptorAgonistsSelective | 13162001000001101 | Formoterol 12micrograms/dose dry powder inhaler |
| 5154 | Beta2AdrenoceptorAgonistsSelective | 13162101000001100 | Fluticasone 50micrograms/dose / Salmeterol 25micrograms/dose inhaler CFC free |
| 5154 | Beta2AdrenoceptorAgonistsSelective | 13162201000001107 | Fluticasone 125micrograms/dose / Salmeterol 25micrograms/dose inhaler CFC free |
| 5154 | Beta2AdrenoceptorAgonistsSelective | 13162301000001103 | Fluticasone 250micrograms/dose / Salmeterol 25micrograms/dose inhaler CFC free |
| 5154 | Beta2AdrenoceptorAgonistsSelective | 13162401000001106 | Fluticasone propionate 100micrograms/dose / Salmeterol 50micrograms/dose dry powder inhaler |
| 5154 | Beta2AdrenoceptorAgonistsSelective | 13162501000001105 | Fluticasone propionate 250micrograms/dose / Salmeterol 50micrograms/dose dry powder inhaler |
| 5154 | Beta2AdrenoceptorAgonistsSelective | 13162601000001109 | Fluticasone propionate 500micrograms/dose / Salmeterol 50micrograms/dose dry powder inhaler |
| 5154 | Beta2AdrenoceptorAgonistsSelective | 13164501000001104 | Fenoterol 100micrograms/dose / Ipratropium 40micrograms/dose inhaler |
| 5154 | Beta2AdrenoceptorAgonistsSelective | 13164601000001100 | Fenoterol 100micrograms/dose / Ipratropium bromide 40micrograms/dose breath actuated inhaler |
| 5154 | Beta2AdrenoceptorAgonistsSelective | 13164701000001105 | Salbutamol 100micrograms/dose / Ipratropium 20micrograms/dose inhaler |
| 5154 | Beta2AdrenoceptorAgonistsSelective | 13166001000001102 | Salbutamol 100micrograms/dose / Beclometasone 50micrograms/dose inhaler |
| 5154 | Beta2AdrenoceptorAgonistsSelective | 13168701000001101 | Sodium cromoglicate 1mg/dose / Salbutamol 100micrograms/dose inhaler with spacer |
| 5154 | Beta2AdrenoceptorAgonistsSelective | 13168801000001105 | Sodium cromoglicate 1mg/dose / Salbutamol 100micrograms/dose inhaler |
| 5154 | Beta2AdrenoceptorAgonistsSelective | 13203711000001107 | Serevent 25micrograms/dose Evohaler (Dowelhurst Ltd) |
| 5154 | Beta2AdrenoceptorAgonistsSelective | 13206411000001106 | Symbicort 100/6 Turbohaler (Dowelhurst Ltd) |
| 5154 | Beta2AdrenoceptorAgonistsSelective | 13206611000001109 | Symbicort 400/12 Turbohaler (Dowelhurst Ltd) |
| 5154 | Beta2AdrenoceptorAgonistsSelective | 13533511000001104 | Salbulin Novolizer 100micrograms/dose inhalation powder (Viatris UK Healthcare Ltd) |
| 5154 | Beta2AdrenoceptorAgonistsSelective | 13533711000001109 | Salbulin Novolizer 100micrograms/dose inhalation powder refill (Viatris UK Healthcare Ltd) |
| 5154 | Beta2AdrenoceptorAgonistsSelective | 13566111000001109 | Salbutamol 100micrograms/dose dry powder inhalation cartridge |
| 5154 | Beta2AdrenoceptorAgonistsSelective | 13566211000001103 | Salbutamol 100micrograms/dose dry powder inhalation cartridge with device |
| 5154 | Beta2AdrenoceptorAgonistsSelective | 13626311000001105 | Salbutamol 2mg/5ml oral solution sugar free (Pinewood Healthcare) |
| 5154 | Beta2AdrenoceptorAgonistsSelective | 13837711000001100 | Bricanyl 500micrograms/dose Turbohaler (DE Pharmaceuticals) |
| 5154 | Beta2AdrenoceptorAgonistsSelective | 13943811000001108 | Oxis 6 Turbohaler (DE Pharmaceuticals) |
| 5154 | Beta2AdrenoceptorAgonistsSelective | 13944011000001100 | Oxis 12 Turbohaler (DE Pharmaceuticals) |
| 5154 | Beta2AdrenoceptorAgonistsSelective | 13958011000001106 | Symbicort 100/6 Turbohaler (DE Pharmaceuticals) |
| 5154 | Beta2AdrenoceptorAgonistsSelective | 13958611000001104 | Symbicort 200/6 Turbohaler (DE Pharmaceuticals) |
| 5154 | Beta2AdrenoceptorAgonistsSelective | 13959211000001106 | Symbicort 400/12 Turbohaler (DE Pharmaceuticals) |
| 5154 | Beta2AdrenoceptorAgonistsSelective | 13967411000001108 | Ventolin 200micrograms/dose Accuhaler (DE Pharmaceuticals) |
| 5154 | Beta2AdrenoceptorAgonistsSelective | 13968911000001104 | Ventolin 100micrograms/dose Evohaler (DE Pharmaceuticals) |
| 5154 | Beta2AdrenoceptorAgonistsSelective | 13991111000001107 | Terbutaline 500micrograms/dose Turbohaler (DE Pharmaceuticals) |
| 5154 | Beta2AdrenoceptorAgonistsSelective | 13997111000001100 | Seretide 100 Accuhaler (DE Pharmaceuticals) |
| 5154 | Beta2AdrenoceptorAgonistsSelective | 13997511000001109 | Seretide 250 Accuhaler (DE Pharmaceuticals) |
| 5154 | Beta2AdrenoceptorAgonistsSelective | 13997811000001107 | Serevent 50micrograms/dose Accuhaler (DE Pharmaceuticals) |
| 5154 | Beta2AdrenoceptorAgonistsSelective | 13998111000001104 | Seretide 125 Evohaler (DE Pharmaceuticals) |
| 5154 | Beta2AdrenoceptorAgonistsSelective | 13998411000001109 | Seretide 250 Evohaler (DE Pharmaceuticals) |
| 5154 | Beta2AdrenoceptorAgonistsSelective | 14207711000001100 | Bambec 10mg tablets (Sigma Pharmaceuticals Plc) |
| 5154 | Beta2AdrenoceptorAgonistsSelective | 14208011000001101 | Bambec 20mg tablets (Sigma Pharmaceuticals Plc) |
| 5154 | Beta2AdrenoceptorAgonistsSelective | 14214011000001107 | Duovent UDVs nebuliser liquid 4ml (Sigma Pharmaceuticals Plc) |
| 5154 | Beta2AdrenoceptorAgonistsSelective | 14239811000001105 | Foradil 12microgram inhalation powder capsules with device (Sigma Pharmaceuticals Plc) |
| 5154 | Beta2AdrenoceptorAgonistsSelective | 14620511000001104 | Symbicort 100/6 Turbohaler (Sigma Pharmaceuticals Plc) |
| 5154 | Beta2AdrenoceptorAgonistsSelective | 14620711000001109 | Symbicort 200/6 Turbohaler (Sigma Pharmaceuticals Plc) |
| 5154 | Beta2AdrenoceptorAgonistsSelective | 14621111000001102 | Symbicort 400/12 Turbohaler (Sigma Pharmaceuticals Plc) |
| 5154 | Beta2AdrenoceptorAgonistsSelective | 14662411000001107 | Bricanyl 500micrograms/dose Turbohaler (Sigma Pharmaceuticals Plc) |
| 5154 | Beta2AdrenoceptorAgonistsSelective | 14674111000001100 | Seretide 100 Accuhaler (Sigma Pharmaceuticals Plc) |
| 5154 | Beta2AdrenoceptorAgonistsSelective | 14674711000001104 | Seretide 250 Accuhaler (Sigma Pharmaceuticals Plc) |
| 5154 | Beta2AdrenoceptorAgonistsSelective | 14705211000001107 | Seretide 500 Accuhaler (Sigma Pharmaceuticals Plc) |
| 5154 | Beta2AdrenoceptorAgonistsSelective | 14705411000001106 | Seretide 125 Evohaler (Sigma Pharmaceuticals Plc) |
| 5154 | Beta2AdrenoceptorAgonistsSelective | 14705611000001109 | Oxis 12 Turbohaler (Sigma Pharmaceuticals Plc) |
| 5154 | Beta2AdrenoceptorAgonistsSelective | 14705811000001108 | Seretide 250 Evohaler (Sigma Pharmaceuticals Plc) |
| 5154 | Beta2AdrenoceptorAgonistsSelective | 14706011000001106 | Serevent 50micrograms/dose Accuhaler (Sigma Pharmaceuticals Plc) |
| 5154 | Beta2AdrenoceptorAgonistsSelective | 14706211000001101 | Serevent 25micrograms/dose Evohaler (Sigma Pharmaceuticals Plc) |
| 5154 | Beta2AdrenoceptorAgonistsSelective | 14706311000001109 | Oxis 6 Turbohaler (Sigma Pharmaceuticals Plc) |
| 5154 | Beta2AdrenoceptorAgonistsSelective | 14750911000001104 | Ventolin 200micrograms/dose Accuhaler (Sigma Pharmaceuticals Plc) |
| 5154 | Beta2AdrenoceptorAgonistsSelective | 14759211000001101 | Volmax 8mg modified-release tablets (Sigma Pharmaceuticals Plc) |
| 5154 | Beta2AdrenoceptorAgonistsSelective | 15522411000001103 | Salipraneb 0.5mg/2.5mg nebuliser solution 2.5ml ampoules (Arrow Generics Ltd) |
| 5154 | Beta2AdrenoceptorAgonistsSelective | 15534911000001100 | Salbutamol 2.5mg/2.5ml / Ipratropium bromide 500micrograms/2.5ml nebuliser liquid ampoules |
| 5154 | Beta2AdrenoceptorAgonistsSelective | 16219211000001106 | Oxis 6 Turbohaler (Lexon (UK) Ltd) |
| 5154 | Beta2AdrenoceptorAgonistsSelective | 16219411000001105 | Oxis 12 Turbohaler (Lexon (UK) Ltd) |
| 5154 | Beta2AdrenoceptorAgonistsSelective | 16240811000001109 | Seretide 100 Accuhaler (Lexon (UK) Ltd) |
| 5154 | Beta2AdrenoceptorAgonistsSelective | 16241011000001107 | Seretide 250 Accuhaler (Lexon (UK) Ltd) |
| 5154 | Beta2AdrenoceptorAgonistsSelective | 16241111000001108 | Seretide 500 Accuhaler (Lexon (UK) Ltd) |
| 5154 | Beta2AdrenoceptorAgonistsSelective | 16241311000001105 | Seretide 125 Evohaler (Lexon (UK) Ltd) |
| 5154 | Beta2AdrenoceptorAgonistsSelective | 16241511000001104 | Seretide 250 Evohaler (Lexon (UK) Ltd) |
| 5154 | Beta2AdrenoceptorAgonistsSelective | 16241711000001109 | Serevent 25micrograms/dose Evohaler (Lexon (UK) Ltd) |
| 5154 | Beta2AdrenoceptorAgonistsSelective | 16241911000001106 | Serevent 50micrograms/dose Accuhaler (Lexon (UK) Ltd) |
| 5154 | Beta2AdrenoceptorAgonistsSelective | 16545911000001103 | Foradil 12microgram inhalation powder capsules with device (Stephar (U.K.) Ltd) |
| 5154 | Beta2AdrenoceptorAgonistsSelective | 16581611000001101 | Foradil 12microgram inhalation powder capsules with device (Mawdsley-Brooks & Company Ltd) |
| 5154 | Beta2AdrenoceptorAgonistsSelective | 16585911000001109 | Oxis 12 Turbohaler (Stephar (U.K.) Ltd) |
| 5154 | Beta2AdrenoceptorAgonistsSelective | 16587911000001104 | Seretide 100 Accuhaler (Stephar (U.K.) Ltd) |
| 5154 | Beta2AdrenoceptorAgonistsSelective | 16588111000001101 | Seretide 250 Evohaler (Stephar (U.K.) Ltd) |
| 5154 | Beta2AdrenoceptorAgonistsSelective | 16728511000001101 | Seretide 50 Evohaler (Waymade Healthcare Plc) |
| 5154 | Beta2AdrenoceptorAgonistsSelective | 16733611000001107 | Salbutamol 2.5mg/2.5ml / Ipratropium bromide 500micrograms/2.5ml nebuliser liquid ampoules (A A H Pharmaceuticals Ltd) |
| 5154 | Beta2AdrenoceptorAgonistsSelective | 17026511000001100 | Salbutamol 2.5mg/2.5ml nebuliser liquid unit dose vials (Alliance Healthcare (Distribution) Ltd) |
| 5154 | Beta2AdrenoceptorAgonistsSelective | 17026711000001105 | Salbutamol 5mg/2.5ml nebuliser liquid unit dose vials (Alliance Healthcare (Distribution) Ltd) |
| 5154 | Beta2AdrenoceptorAgonistsSelective | 17299911000001102 | Onbrez Breezhaler 300microgram inhalation powder capsules with device (Novartis Pharmaceuticals UK Ltd) |
| 5154 | Beta2AdrenoceptorAgonistsSelective | 17300811000001109 | Onbrez Breezhaler 150microgram inhalation powder capsules with device (Novartis Pharmaceuticals UK Ltd) |
| 5154 | Beta2AdrenoceptorAgonistsSelective | 17313711000001107 | Indacaterol 150microgram inhalation powder capsules with device |
| 5154 | Beta2AdrenoceptorAgonistsSelective | 17313811000001104 | Indacaterol 300microgram inhalation powder capsules with device |
| 5154 | Beta2AdrenoceptorAgonistsSelective | 17344411000001108 | Oxis 6 Turbohaler (Mawdsley-Brooks & Company Ltd) |
| 5154 | Beta2AdrenoceptorAgonistsSelective | 17344611000001106 | Oxis 12 Turbohaler (Mawdsley-Brooks & Company Ltd) |
| 5154 | Beta2AdrenoceptorAgonistsSelective | 17438511000001108 | Salamol 100micrograms/dose Easi-Breathe inhaler (Mawdsley-Brooks & Company Ltd) |
| 5154 | Beta2AdrenoceptorAgonistsSelective | 17440111000001102 | Seretide 100 Accuhaler (Mawdsley-Brooks & Company Ltd) |
| 5154 | Beta2AdrenoceptorAgonistsSelective | 17440311000001100 | Seretide 250 Accuhaler (Mawdsley-Brooks & Company Ltd) |
| 5154 | Beta2AdrenoceptorAgonistsSelective | 17440511000001106 | Seretide 500 Accuhaler (Mawdsley-Brooks & Company Ltd) |
| 5154 | Beta2AdrenoceptorAgonistsSelective | 17440711000001101 | Seretide 125 Evohaler (Mawdsley-Brooks & Company Ltd) |
| 5154 | Beta2AdrenoceptorAgonistsSelective | 17440911000001104 | Seretide 250 Evohaler (Mawdsley-Brooks & Company Ltd) |
| 5154 | Beta2AdrenoceptorAgonistsSelective | 17441111000001108 | Serevent 50micrograms/dose Accuhaler (Mawdsley-Brooks & Company Ltd) |
| 5154 | Beta2AdrenoceptorAgonistsSelective | 17441311000001105 | Serevent 25micrograms/dose Evohaler (Mawdsley-Brooks & Company Ltd) |
| 5154 | Beta2AdrenoceptorAgonistsSelective | 17457311000001102 | Symbicort 100/6 Turbohaler (Mawdsley-Brooks & Company Ltd) |
| 5154 | Beta2AdrenoceptorAgonistsSelective | 17457611000001107 | Symbicort 200/6 Turbohaler (Mawdsley-Brooks & Company Ltd) |
| 5154 | Beta2AdrenoceptorAgonistsSelective | 17457811000001106 | Symbicort 400/12 Turbohaler (Mawdsley-Brooks & Company Ltd) |
| 5154 | Beta2AdrenoceptorAgonistsSelective | 17470911000001100 | Ventolin 200micrograms/dose Accuhaler (Mawdsley-Brooks & Company Ltd) |
| 5154 | Beta2AdrenoceptorAgonistsSelective | 17471111000001109 | Ventolin 100micrograms/dose Evohaler (Mawdsley-Brooks & Company Ltd) |
| 5154 | Beta2AdrenoceptorAgonistsSelective | 17471311000001106 | Ventolin 5mg Nebules (Mawdsley-Brooks & Company Ltd) |
| 5154 | Beta2AdrenoceptorAgonistsSelective | 17471511000001100 | Ventolin 2.5mg Nebules (Mawdsley-Brooks & Company Ltd) |
| 5154 | Beta2AdrenoceptorAgonistsSelective | 17495111000001101 | Bambec 10mg tablets (Mawdsley-Brooks & Company Ltd) |
| 5154 | Beta2AdrenoceptorAgonistsSelective | 17602911000001107 | Bricanyl 500micrograms/dose Turbohaler (Necessity Supplies Ltd) |
| 5154 | Beta2AdrenoceptorAgonistsSelective | 17616311000001108 | Oxis 6 Turbohaler (Necessity Supplies Ltd) |
| 5154 | Beta2AdrenoceptorAgonistsSelective | 17616911000001109 | Oxis 12 Turbohaler (Necessity Supplies Ltd) |
| 5154 | Beta2AdrenoceptorAgonistsSelective | 17661711000001102 | Terbutaline 5mg/2ml nebuliser liquid unit dose vials (Arrow Generics Ltd) |
| 5154 | Beta2AdrenoceptorAgonistsSelective | 17927011000001101 | Salbutamol 100micrograms/dose inhaler CFC free (Phoenix Healthcare Distribution Ltd) |
| 5154 | Beta2AdrenoceptorAgonistsSelective | 17927211000001106 | Salbutamol 2.5mg/2.5ml nebuliser liquid unit dose vials (Phoenix Healthcare Distribution Ltd) |
| 5154 | Beta2AdrenoceptorAgonistsSelective | 17927411000001105 | Salbutamol 5mg/2.5ml nebuliser liquid unit dose vials (Phoenix Healthcare Distribution Ltd) |
| 5154 | Beta2AdrenoceptorAgonistsSelective | 18041311000001106 | Salbutamol 100micrograms/dose inhaler CFC free (Sandoz Ltd) |
| 5154 | Beta2AdrenoceptorAgonistsSelective | 18058411000001109 | Foradil 12microgram inhalation powder capsules with device (Lexon (UK) Ltd) |
| 5154 | Beta2AdrenoceptorAgonistsSelective | 18080711000001105 | Salbutamol 2.5mg/2.5ml nebuliser liquid unit dose vials (Fannin (UK) Ltd) |
| 5154 | Beta2AdrenoceptorAgonistsSelective | 18080911000001107 | Salbutamol 5mg/2.5ml nebuliser liquid unit dose vials (Fannin (UK) Ltd) |
| 5154 | Beta2AdrenoceptorAgonistsSelective | 18148111000001107 | Asmavent 100micrograms/dose inhaler CFC free (Kent Pharma (UK) Ltd) |
| 5154 | Beta2AdrenoceptorAgonistsSelective | 18185011000001106 | Seretide 100 Accuhaler (Necessity Supplies Ltd) |
| 5154 | Beta2AdrenoceptorAgonistsSelective | 18185211000001101 | Seretide 250 Accuhaler (Necessity Supplies Ltd) |
| 5154 | Beta2AdrenoceptorAgonistsSelective | 18185411000001102 | Seretide 500 Accuhaler (Necessity Supplies Ltd) |
| 5154 | Beta2AdrenoceptorAgonistsSelective | 18185611000001104 | Seretide 125 Evohaler (Necessity Supplies Ltd) |
| 5154 | Beta2AdrenoceptorAgonistsSelective | 18185811000001100 | Seretide 250 Evohaler (Necessity Supplies Ltd) |
| 5154 | Beta2AdrenoceptorAgonistsSelective | 18186011000001102 | Serevent 50micrograms/dose Accuhaler (Necessity Supplies Ltd) |
| 5154 | Beta2AdrenoceptorAgonistsSelective | 18186311000001104 | Serevent 25micrograms/dose Evohaler (Necessity Supplies Ltd) |
| 5154 | Beta2AdrenoceptorAgonistsSelective | 18195111000001106 | Symbicort 100/6 Turbohaler (Necessity Supplies Ltd) |
| 5154 | Beta2AdrenoceptorAgonistsSelective | 18195311000001108 | Symbicort 200/6 Turbohaler (Necessity Supplies Ltd) |
| 5154 | Beta2AdrenoceptorAgonistsSelective | 18195511000001102 | Symbicort 400/12 Turbohaler (Necessity Supplies Ltd) |
| 5154 | Beta2AdrenoceptorAgonistsSelective | 18220811000001104 | Combivent nebuliser liquid 2.5ml UDVs (Mawdsley-Brooks & Company Ltd) |
| 5154 | Beta2AdrenoceptorAgonistsSelective | 18488911000001103 | Onbrez Breezhaler 150microgram inhalation powder capsules with device (Waymade Healthcare Plc) |
| 5154 | Beta2AdrenoceptorAgonistsSelective | 18489111000001108 | Onbrez Breezhaler 300microgram inhalation powder capsules with device (Waymade Healthcare Plc) |
| 5154 | Beta2AdrenoceptorAgonistsSelective | 18610611000001106 | Salamol 100micrograms/dose Easi-Breathe inhaler (DE Pharmaceuticals) |
| 5154 | Beta2AdrenoceptorAgonistsSelective | 18611411000001104 | Seretide 500 Accuhaler (DE Pharmaceuticals) |
| 5154 | Beta2AdrenoceptorAgonistsSelective | 18611611000001101 | Serevent 25micrograms/dose Evohaler (DE Pharmaceuticals) |
| 5154 | Beta2AdrenoceptorAgonistsSelective | 19541511000001101 | Combivent nebuliser liquid 2.5ml UDVs (DE Pharmaceuticals) |
| 5154 | Beta2AdrenoceptorAgonistsSelective | 19562111000001107 | Neovent 25micrograms/dose inhaler CFC free (Teva UK Ltd) |
| 5154 | Beta2AdrenoceptorAgonistsSelective | 19568411000001100 | Neovent 25micrograms/dose inhaler CFC free (Kent Pharma (UK) Ltd) |
| 5154 | Beta2AdrenoceptorAgonistsSelective | 19869411000001109 | Combivent nebuliser liquid 2.5ml UDVs (Lexon (UK) Ltd) |
| 5154 | Beta2AdrenoceptorAgonistsSelective | 20031011000001108 | Ventolin 200micrograms/dose Accuhaler (Lexon (UK) Ltd) |
| 5154 | Beta2AdrenoceptorAgonistsSelective | 20434411000001107 | Salamol 100micrograms/dose inhaler CFC free (Arrow Generics Ltd) |
| 5154 | Beta2AdrenoceptorAgonistsSelective | 20638911000001108 | Salmeterol 25micrograms/dose inhaler CFC free (A A H Pharmaceuticals Ltd) |
| 5154 | Beta2AdrenoceptorAgonistsSelective | 21019411000001101 | Flutiform 125micrograms/dose / 5micrograms/dose inhaler (Napp Pharmaceuticals Ltd) |
| 5154 | Beta2AdrenoceptorAgonistsSelective | 21019711000001107 | Flutiform 250micrograms/dose / 10micrograms/dose inhaler (Napp Pharmaceuticals Ltd) |
| 5154 | Beta2AdrenoceptorAgonistsSelective | 21020611000001104 | Flutiform 50micrograms/dose / 5micrograms/dose inhaler (Napp Pharmaceuticals Ltd) |
| 5154 | Beta2AdrenoceptorAgonistsSelective | 21113711000001102 | Fluticasone 125micrograms/dose / Formoterol 5micrograms/dose inhaler CFC free |
| 5154 | Beta2AdrenoceptorAgonistsSelective | 21113811000001105 | Fluticasone 250micrograms/dose / Formoterol 10micrograms/dose inhaler CFC free |
| 5154 | Beta2AdrenoceptorAgonistsSelective | 21113911000001100 | Fluticasone 50micrograms/dose / Formoterol 5micrograms/dose inhaler CFC free |
| 5154 | Beta2AdrenoceptorAgonistsSelective | 21787311000001103 | Terbutaline 5mg/2ml nebuliser liquid unit dose vials (Waymade Healthcare Plc) |
| 5154 | Beta2AdrenoceptorAgonistsSelective | 21866011000001104 | Foradil 12microgram inhalation powder capsules with device (Waymade Healthcare Plc) |
| 5154 | Beta2AdrenoceptorAgonistsSelective | 21895011000001105 | Salbutamol 100micrograms/dose inhaler CFC free (Waymade Healthcare Plc) |
| 5154 | Beta2AdrenoceptorAgonistsSelective | 21895211000001100 | Salbutamol 2mg tablets (Waymade Healthcare Plc) |
| 5154 | Beta2AdrenoceptorAgonistsSelective | 21895411000001101 | Salbutamol 4mg tablets (Waymade Healthcare Plc) |
| 5154 | Beta2AdrenoceptorAgonistsSelective | 22354211000001108 | Salbutamol 100micrograms/dose inhaler CFC free (AM Distributions (Yorkshire) Ltd) |
| 5154 | Beta2AdrenoceptorAgonistsSelective | 22355111000001103 | Terbutaline 5mg/2ml nebuliser liquid unit dose vials (AM Distributions (Yorkshire) Ltd) |
| 5154 | Beta2AdrenoceptorAgonistsSelective | 22503111000001109 | AirSalb 100micrograms/dose inhaler CFC free (Sandoz Ltd) |
| 5154 | Beta2AdrenoceptorAgonistsSelective | 22650111000001100 | Vertine 25micrograms/dose inhaler CFC free (Teva UK Ltd) |
| 5154 | Beta2AdrenoceptorAgonistsSelective | 23433411000001108 | Salbutamol 2.5mg/2.5ml / Ipratropium bromide 500micrograms/2.5ml nebuliser liquid unit dose vials (J M McGill Ltd) |
| 5154 | Beta2AdrenoceptorAgonistsSelective | 23621711000001102 | Relvar Ellipta 184micrograms/dose / 22micrograms/dose dry powder inhaler (GlaxoSmithKline UK Ltd) |
| 5154 | Beta2AdrenoceptorAgonistsSelective | 23622011000001107 | Relvar Ellipta 92micrograms/dose / 22micrograms/dose dry powder inhaler (GlaxoSmithKline UK Ltd) |
| 5154 | Beta2AdrenoceptorAgonistsSelective | 23661311000001105 | Fluticasone furoate 184micrograms/dose / Vilanterol 22micrograms/dose dry powder inhaler |
| 5154 | Beta2AdrenoceptorAgonistsSelective | 23661411000001103 | Fluticasone furoate 92micrograms/dose / Vilanterol 22micrograms/dose dry powder inhaler |
| 5154 | Beta2AdrenoceptorAgonistsSelective | 23961011000001108 | Salbutamol 2.5mg/2.5ml / Ipratropium bromide 500micrograms/2.5ml nebuliser liquid unit dose vials (Niche Pharma Ltd) |
| 5154 | Beta2AdrenoceptorAgonistsSelective | 24608011000001103 | Striverdi Respimat 2.5micrograms/dose inhalation solution cartridge with device (Boehringer Ingelheim Ltd) |
| 5154 | Beta2AdrenoceptorAgonistsSelective | 24644611000001108 | Anoro Ellipta 55micrograms/dose / 22micrograms/dose dry powder inhaler (GlaxoSmithKline UK Ltd) |
| 5154 | Beta2AdrenoceptorAgonistsSelective | 24645511000001105 | Umeclidinium bromide 65micrograms/dose / Vilanterol 22micrograms/dose dry powder inhaler |
| 5154 | Beta2AdrenoceptorAgonistsSelective | 24670111000001108 | Olodaterol 2.5micrograms/dose solution for inhalation cartridge with device CFC free |
| 5154 | Beta2AdrenoceptorAgonistsSelective | 25254111000001105 | DuoResp Spiromax 160micrograms/dose / 4.5micrograms/dose dry powder inhaler (Teva UK Ltd) |
| 5154 | Beta2AdrenoceptorAgonistsSelective | 25254711000001106 | DuoResp Spiromax 320micrograms/dose / 9micrograms/dose dry powder inhaler (Teva UK Ltd) |
| 5154 | Beta2AdrenoceptorAgonistsSelective | 26112111000001106 | Fostair NEXThaler 100micrograms/dose / 6micrograms/dose dry powder inhaler (Chiesi Ltd) |
| 5154 | Beta2AdrenoceptorAgonistsSelective | 26148711000001101 | Beclometasone 100micrograms/dose / Formoterol 6micrograms/dose dry powder inhaler |
| 5154 | Beta2AdrenoceptorAgonistsSelective | 28007211000001102 | Ultibro Breezhaler 85microgram/43microgram inhalation powder capsules with device (Novartis Pharmaceuticals UK Ltd) |
| 5154 | Beta2AdrenoceptorAgonistsSelective | 28049611000001104 | Indacaterol 85micrograms/dose / Glycopyrronium bromide 54micrograms/dose inhalation powder capsules with device |
| 5154 | Beta2AdrenoceptorAgonistsSelective | 28194211000001100 | Terbutaline 7.5mg/15ml solution for infusion pre-filled syringes (Special Order) |
| 5154 | Beta2AdrenoceptorAgonistsSelective | 28279711000001100 | Terbutaline 7.5mg/15ml solution for infusion pre-filled syringes |
| 5154 | Beta2AdrenoceptorAgonistsSelective | 28357211000001106 | Duaklir 340micrograms/dose / 12micrograms/dose Genuair (AstraZeneca UK Ltd) |
| 5154 | Beta2AdrenoceptorAgonistsSelective | 28365011000001100 | Aclidinium bromide 396micrograms/dose / Formoterol 11.8micrograms/dose dry powder inhaler |
| 5154 | Beta2AdrenoceptorAgonistsSelective | 28410811000001107 | Berodual N inhaler (Imported (Germany)) |
| 5154 | Beta2AdrenoceptorAgonistsSelective | 28422511000001101 | Fenoterol 50micrograms/dose / Ipratropium bromide 20micrograms/dose inhaler CFC free |
| 5154 | Beta2AdrenoceptorAgonistsSelective | 29211411000001103 | Salbutamol 2.5mg/2.5ml / Ipratropium bromide 500micrograms/2.5ml nebuliser liquid unit dose vials (Ennogen Healthcare Ltd) |
| 5154 | Beta2AdrenoceptorAgonistsSelective | 29749211000001101 | Flutiform 125micrograms/dose / 5micrograms/dose inhaler (Waymade Healthcare Plc) |
| 5154 | Beta2AdrenoceptorAgonistsSelective | 29749411000001102 | Flutiform 50micrograms/dose / 5micrograms/dose inhaler (Waymade Healthcare Plc) |
| 5154 | Beta2AdrenoceptorAgonistsSelective | 29749611000001104 | Flutiform 250micrograms/dose / 10micrograms/dose inhaler (Waymade Healthcare Plc) |
| 5154 | Beta2AdrenoceptorAgonistsSelective | 29782111000001107 | Sirdupla 25micrograms/dose / 125micrograms/dose inhaler (Viatris UK Healthcare Ltd) |
| 5154 | Beta2AdrenoceptorAgonistsSelective | 29782511000001103 | Sirdupla 25micrograms/dose / 250micrograms/dose inhaler (Viatris UK Healthcare Ltd) |
| 5154 | Beta2AdrenoceptorAgonistsSelective | 29971311000001100 | Spiolto Respimat 2.5micrograms/dose / 2.5micrograms/dose inhalation solution cartridge with device (Boehringer Ingelheim Ltd) |
| 5154 | Beta2AdrenoceptorAgonistsSelective | 29980011000001104 | Bricanyl 500micrograms/dose Turbohaler (Lexon (UK) Ltd) |
| 5154 | Beta2AdrenoceptorAgonistsSelective | 29987211000001108 | Tiotropium bromide 2.5micrograms/dose / Olodaterol 2.5micrograms/dose solution for inhalation cartridge with device CFC free |
| 5154 | Beta2AdrenoceptorAgonistsSelective | 30041511000001100 | Atimos Modulite 12micrograms/dose inhaler (Waymade Healthcare Plc) |
| 5154 | Beta2AdrenoceptorAgonistsSelective | 30094711000001100 | Salbutamol 100micrograms/dose inhaler CFC free (DE Pharmaceuticals) |
| 5154 | Beta2AdrenoceptorAgonistsSelective | 30094911000001103 | Salbutamol 2mg tablets (DE Pharmaceuticals) |
| 5154 | Beta2AdrenoceptorAgonistsSelective | 30095111000001102 | Salbutamol 4mg tablets (DE Pharmaceuticals) |
| 5154 | Beta2AdrenoceptorAgonistsSelective | 30095311000001100 | Salmeterol 25micrograms/dose inhaler CFC free (DE Pharmaceuticals) |
| 5154 | Beta2AdrenoceptorAgonistsSelective | 30253911000001100 | Sirdupla 25micrograms/dose / 250micrograms/dose inhaler (Waymade Healthcare Plc) |
| 5154 | Beta2AdrenoceptorAgonistsSelective | 30273011000001101 | Relvar Ellipta 184micrograms/dose / 22micrograms/dose dry powder inhaler (Waymade Healthcare Plc) |
| 5154 | Beta2AdrenoceptorAgonistsSelective | 30273211000001106 | Relvar Ellipta 92micrograms/dose / 22micrograms/dose dry powder inhaler (Waymade Healthcare Plc) |
| 5154 | Beta2AdrenoceptorAgonistsSelective | 30891711000001102 | Salbutamol 100micrograms/dose inhaler CFC free (Mawdsley-Brooks & Company Ltd) |
| 5154 | Beta2AdrenoceptorAgonistsSelective | 30891911000001100 | Salbutamol 2.5mg/2.5ml nebuliser liquid unit dose vials (Mawdsley-Brooks & Company Ltd) |
| 5154 | Beta2AdrenoceptorAgonistsSelective | 30892111000001108 | Salbutamol 5mg/2.5ml nebuliser liquid unit dose vials (Mawdsley-Brooks & Company Ltd) |
| 5154 | Beta2AdrenoceptorAgonistsSelective | 30924211000001107 | Terbutaline 5mg/2ml nebuliser liquid unit dose vials (Mawdsley-Brooks & Company Ltd) |
| 5154 | Beta2AdrenoceptorAgonistsSelective | 30950311000001106 | AirFluSal Forspiro 50micrograms/dose / 500micrograms/dose dry powder inhaler (Sandoz Ltd) |
| 5154 | Beta2AdrenoceptorAgonistsSelective | 31063111000001106 | Fostair NEXThaler 200micrograms/dose / 6micrograms/dose dry powder inhaler (Chiesi Ltd) |
| 5154 | Beta2AdrenoceptorAgonistsSelective | 31063411000001101 | Fostair 200micrograms/dose / 6micrograms/dose inhaler (Chiesi Ltd) |
| 5154 | Beta2AdrenoceptorAgonistsSelective | 31064411000001103 | Fluticasone 125micrograms/dose / Salmeterol 25micrograms/dose inhaler CFC free (A A H Pharmaceuticals Ltd) |
| 5154 | Beta2AdrenoceptorAgonistsSelective | 31064611000001100 | Fluticasone 250micrograms/dose / Salmeterol 25micrograms/dose inhaler CFC free (A A H Pharmaceuticals Ltd) |
| 5154 | Beta2AdrenoceptorAgonistsSelective | 31064811000001101 | Fluticasone propionate 500micrograms/dose / Salmeterol 50micrograms/dose dry powder inhaler (A A H Pharmaceuticals Ltd) |
| 5154 | Beta2AdrenoceptorAgonistsSelective | 31087411000001106 | Beclometasone 200micrograms/dose / Formoterol 6micrograms/dose inhaler CFC free |
| 5154 | Beta2AdrenoceptorAgonistsSelective | 31087511000001105 | Beclometasone 200micrograms/dose / Formoterol 6micrograms/dose dry powder inhaler |
| 5154 | Beta2AdrenoceptorAgonistsSelective | 31457011000001100 | Fluticasone furoate 184micrograms/dose / Vilanterol 22micrograms/dose dry powder inhaler (J M McGill Ltd) |
| 5154 | Beta2AdrenoceptorAgonistsSelective | 31457411000001109 | Fluticasone furoate 92micrograms/dose / Vilanterol 22micrograms/dose dry powder inhaler (J M McGill Ltd) |
| 5154 | Beta2AdrenoceptorAgonistsSelective | 32333511000001106 | Fluticasone furoate 184micrograms/dose / Vilanterol 22micrograms/dose dry powder inhaler (Colorama Pharmaceuticals Ltd) |
| 5154 | Beta2AdrenoceptorAgonistsSelective | 32333911000001104 | Fluticasone furoate 92micrograms/dose / Vilanterol 22micrograms/dose dry powder inhaler (Colorama Pharmaceuticals Ltd) |
| 5154 | Beta2AdrenoceptorAgonistsSelective | 32364511000001104 | Umeclidinium bromide 65micrograms/dose / Vilanterol 22micrograms/dose dry powder inhaler (Ennogen Healthcare Ltd) |
| 5154 | Beta2AdrenoceptorAgonistsSelective | 32480911000001107 | Salbutamol 2.5mg/2.5ml / Ipratropium bromide 500micrograms/2.5ml nebuliser liquid unit dose vials (Colorama Pharmaceuticals Ltd) |
| 5154 | Beta2AdrenoceptorAgonistsSelective | 32629811000001101 | Umeclidinium bromide 65micrograms/dose / Vilanterol 22micrograms/dose dry powder inhaler (J M McGill Ltd) |
| 5154 | Beta2AdrenoceptorAgonistsSelective | 32633011000001106 | Aclidinium bromide 396micrograms/dose / Formoterol 11.8micrograms/dose dry powder inhaler (Colorama Pharmaceuticals Ltd) |
| 5154 | Beta2AdrenoceptorAgonistsSelective | 32684611000001106 | Fluticasone furoate 184micrograms/dose / Vilanterol 22micrograms/dose dry powder inhaler (DE Pharmaceuticals) |
| 5154 | Beta2AdrenoceptorAgonistsSelective | 32684911000001100 | Fluticasone furoate 92micrograms/dose / Vilanterol 22micrograms/dose dry powder inhaler (DE Pharmaceuticals) |
| 5154 | Beta2AdrenoceptorAgonistsSelective | 32898611000001109 | Salipraneb 0.5mg/2.5mg nebuliser solution 2.5ml ampoules (Actavis UK Ltd) |
| 5154 | Beta2AdrenoceptorAgonistsSelective | 32926011000001100 | Symbicort 200micrograms/dose / 6micrograms/dose pressurised inhaler (AstraZeneca UK Ltd) |
| 5154 | Beta2AdrenoceptorAgonistsSelective | 32960711000001105 | Budesonide 200micrograms/dose / Formoterol 6micrograms/dose inhaler CFC free |
| 5154 | Beta2AdrenoceptorAgonistsSelective | 33561211000001109 | Soltel 25micrograms/dose inhaler CFC free (Cipla EU Ltd) |
| 5154 | Beta2AdrenoceptorAgonistsSelective | 33679711000001103 | Aerivio Spiromax 50micrograms/dose / 500micrograms/dose dry powder inhaler (Teva UK Ltd) |
| 5154 | Beta2AdrenoceptorAgonistsSelective | 34023611000001101 | Sereflo 25micrograms/dose / 125micrograms/dose inhaler (Cipla EU Ltd) |
| 5154 | Beta2AdrenoceptorAgonistsSelective | 34023811000001102 | Sereflo 25micrograms/dose / 250micrograms/dose inhaler (Cipla EU Ltd) |
| 5154 | Beta2AdrenoceptorAgonistsSelective | 34215311000001107 | AirFluSal 25micrograms/dose / 125micrograms/dose inhaler (Sandoz Ltd) |
| 5154 | Beta2AdrenoceptorAgonistsSelective | 34215511000001101 | AirFluSal 25micrograms/dose / 250micrograms/dose inhaler (Sandoz Ltd) |
| 5154 | Beta2AdrenoceptorAgonistsSelective | 34675711000001103 | Aloflute 25micrograms/dose / 250micrograms/dose inhaler (Viatris UK Healthcare Ltd) |
| 5154 | Beta2AdrenoceptorAgonistsSelective | 34677011000001107 | Aloflute 25micrograms/dose / 125micrograms/dose inhaler (Viatris UK Healthcare Ltd) |
| 5154 | Beta2AdrenoceptorAgonistsSelective | 34681611000001100 | Trimbow 87micrograms/dose / 5micrograms/dose / 9micrograms/dose inhaler (Chiesi Ltd) |
| 5154 | Beta2AdrenoceptorAgonistsSelective | 34683311000001106 | Generic Trimbow 87micrograms/dose / 5micrograms/dose / 9micrograms/dose inhaler |
| 5154 | Beta2AdrenoceptorAgonistsSelective | 34812111000001106 | Fobumix Easyhaler 320micrograms/dose / 9micrograms/dose dry powder inhaler (Orion Pharma (UK) Ltd) |
| 5154 | Beta2AdrenoceptorAgonistsSelective | 34950311000001108 | Fobumix Easyhaler 160micrograms/dose / 4.5micrograms/dose dry powder inhaler (Orion Pharma (UK) Ltd) |
| 5154 | Beta2AdrenoceptorAgonistsSelective | 34950611000001103 | Fobumix Easyhaler 80micrograms/dose / 4.5micrograms/dose dry powder inhaler (Orion Pharma (UK) Ltd) |
| 5154 | Beta2AdrenoceptorAgonistsSelective | 34952211000001104 | Trelegy Ellipta 92micrograms/dose / 55micrograms/dose / 22micrograms/dose dry powder inhaler (GlaxoSmithKline UK Ltd) |
| 5154 | Beta2AdrenoceptorAgonistsSelective | 34955111000001103 | Generic Trelegy Ellipta 92micrograms/dose / 55micrograms/dose / 22micrograms/dose dry powder inhaler |
| 5154 | Beta2AdrenoceptorAgonistsSelective | 35369511000001103 | Salbutamol 200microgram / Beclometasone 100microgram inhalation powder capsules |
| 5154 | Beta2AdrenoceptorAgonistsSelective | 35515311000001106 | Fusacomb Easyhaler 50micrograms/dose / 500micrograms/dose dry powder inhaler (Orion Pharma (UK) Ltd) |
| 5154 | Beta2AdrenoceptorAgonistsSelective | 35515511000001100 | Fusacomb Easyhaler 50micrograms/dose / 250micrograms/dose dry powder inhaler (Orion Pharma (UK) Ltd) |
| 5154 | Beta2AdrenoceptorAgonistsSelective | 35594011000001105 | Combisal 25micrograms/dose / 50micrograms/dose inhaler (Aspire Pharma Ltd) |
| 5154 | Beta2AdrenoceptorAgonistsSelective | 35594211000001100 | Combisal 25micrograms/dose / 125micrograms/dose inhaler (Aspire Pharma Ltd) |
| 5154 | Beta2AdrenoceptorAgonistsSelective | 35594411000001101 | Combisal 25micrograms/dose / 250micrograms/dose inhaler (Aspire Pharma Ltd) |
| 5154 | Beta2AdrenoceptorAgonistsSelective | 35647311000001101 | Flutiform K-haler 125micrograms/dose / 5micrograms/dose breath actuated inhaler (Napp Pharmaceuticals Ltd) |
| 5154 | Beta2AdrenoceptorAgonistsSelective | 35647511000001107 | Fluticasone 125micrograms/dose / Formoterol 5micrograms/dose breath actuated inhaler CFC free |
| 5154 | Beta2AdrenoceptorAgonistsSelective | 35647611000001106 | Fluticasone 50micrograms/dose / Formoterol 5micrograms/dose breath actuated inhaler CFC free |
| 5154 | Beta2AdrenoceptorAgonistsSelective | 35650811000001109 | Flutiform K-haler 50micrograms/dose / 5micrograms/dose breath actuated inhaler (Napp Pharmaceuticals Ltd) |
| 5154 | Beta2AdrenoceptorAgonistsSelective | 35912011000001109 | Budesonide 100micrograms/dose / Formoterol 6micrograms/dose dry powder inhaler |
| 5154 | Beta2AdrenoceptorAgonistsSelective | 35915911000001100 | Terbutaline 1.5mg/5ml oral solution sugar free |
| 5154 | Beta2AdrenoceptorAgonistsSelective | 35916011000001108 | Terbutaline 2.5mg/5ml solution for injection ampoules |
| 5154 | Beta2AdrenoceptorAgonistsSelective | 35916111000001109 | Terbutaline 250micrograms/dose inhaler with spacer |
| 5154 | Beta2AdrenoceptorAgonistsSelective | 35916211000001103 | Terbutaline 500micrograms/1ml solution for injection ampoules |
| 5154 | Beta2AdrenoceptorAgonistsSelective | 35916311000001106 | Terbutaline 500micrograms/dose dry powder inhaler |
| 5154 | Beta2AdrenoceptorAgonistsSelective | 35916411000001104 | Terbutaline 7.5mg modified-release tablets |
| 5154 | Beta2AdrenoceptorAgonistsSelective | 35926311000001100 | Sodium cromoglicate 1mg/dose / Salbutamol 100micrograms/dose inhaler |
| 5154 | Beta2AdrenoceptorAgonistsSelective | 35926411000001107 | Sodium cromoglicate 1mg/dose / Salbutamol 100micrograms/dose inhaler with spacer |
| 5154 | Beta2AdrenoceptorAgonistsSelective | 35936211000001105 | Salbutamol 100micrograms/dose / Beclometasone 50micrograms/dose inhaler |
| 5154 | Beta2AdrenoceptorAgonistsSelective | 35936311000001102 | Salbutamol 100micrograms/dose / Ipratropium 20micrograms/dose inhaler |
| 5154 | Beta2AdrenoceptorAgonistsSelective | 35936411000001109 | Salbutamol 100micrograms/dose breath actuated inhaler |
| 5154 | Beta2AdrenoceptorAgonistsSelective | 35936511000001108 | Salbutamol 100micrograms/dose inhaler |
| 5154 | Beta2AdrenoceptorAgonistsSelective | 35936611000001107 | Salbutamol 200microgram inhalation powder blisters |
| 5154 | Beta2AdrenoceptorAgonistsSelective | 35936711000001103 | Salbutamol 200microgram inhalation powder blisters with device |
| 5154 | Beta2AdrenoceptorAgonistsSelective | 35936811000001106 | Salbutamol 400microgram inhalation powder blisters |
| 5154 | Beta2AdrenoceptorAgonistsSelective | 35936911000001101 | Salbutamol 400microgram inhalation powder blisters with device |
| 5154 | Beta2AdrenoceptorAgonistsSelective | 35937011000001102 | Salbutamol 5mg/50ml solution for infusion vials |
| 5154 | Beta2AdrenoceptorAgonistsSelective | 35937111000001101 | Salbutamol 8mg modified-release tablets |
| 5154 | Beta2AdrenoceptorAgonistsSelective | 35937211000001107 | Salbutamol 95micrograms/dose dry powder inhaler |
| 5154 | Beta2AdrenoceptorAgonistsSelective | 35937811000001108 | Salmeterol 50microgram inhalation powder blisters with device |
| 5154 | Beta2AdrenoceptorAgonistsSelective | 35938011000001101 | Salmeterol 50microgram inhalation powder blisters |
| 5154 | Beta2AdrenoceptorAgonistsSelective | 36066611000001106 | Fenoterol 100micrograms/dose / Ipratropium 40micrograms/dose inhaler |
| 5154 | Beta2AdrenoceptorAgonistsSelective | 36066711000001102 | Fenoterol 100micrograms/dose / Ipratropium bromide 40micrograms/dose breath actuated inhaler |
| 5154 | Beta2AdrenoceptorAgonistsSelective | 36604711000001102 | Stalpex 50micrograms/dose / 500micrograms/dose dry powder inhaler (Glenmark Pharmaceuticals Europe Ltd) |
| 5154 | Beta2AdrenoceptorAgonistsSelective | 36889311000001107 | DuoResp Spiromax 160micrograms/dose / 4.5micrograms/dose dry powder inhaler (Pharmaram Ltd) |
| 5154 | Beta2AdrenoceptorAgonistsSelective | 36889511000001101 | DuoResp Spiromax 320micrograms/dose / 9micrograms/dose dry powder inhaler (Pharmaram Ltd) |
| 5154 | Beta2AdrenoceptorAgonistsSelective | 36895211000001101 | Serevent 50micrograms/dose Accuhaler (Originalis B.V.) |
| 5154 | Beta2AdrenoceptorAgonistsSelective | 37350511000001103 | Bricanyl 500micrograms/dose Turbohaler (Mawdsley-Brooks & Company Ltd) |
| 5154 | Beta2AdrenoceptorAgonistsSelective | 37364411000001100 | Combivent nebuliser liquid 2.5ml UDVs (CST Pharma Ltd) |
| 5154 | Beta2AdrenoceptorAgonistsSelective | 37391711000001105 | Flutiform 125micrograms/dose / 5micrograms/dose inhaler (CST Pharma Ltd) |
| 5154 | Beta2AdrenoceptorAgonistsSelective | 37395311000001108 | Salamol 100micrograms/dose Easi-Breathe inhaler (CST Pharma Ltd) |
| 5154 | Beta2AdrenoceptorAgonistsSelective | 37397211000001108 | DuoResp Spiromax 320micrograms/dose / 9micrograms/dose dry powder inhaler (Mawdsley-Brooks & Company Ltd) |
| 5154 | Beta2AdrenoceptorAgonistsSelective | 37397411000001107 | DuoResp Spiromax 160micrograms/dose / 4.5micrograms/dose dry powder inhaler (Mawdsley-Brooks & Company Ltd) |
| 5154 | Beta2AdrenoceptorAgonistsSelective | 37415511000001109 | Serevent 50micrograms/dose Accuhaler (CST Pharma Ltd) |
| 5154 | Beta2AdrenoceptorAgonistsSelective | 37434411000001103 | DuoResp Spiromax 160micrograms/dose / 4.5micrograms/dose dry powder inhaler (CST Pharma Ltd) |
| 5154 | Beta2AdrenoceptorAgonistsSelective | 37434611000001100 | DuoResp Spiromax 320micrograms/dose / 9micrograms/dose dry powder inhaler (CST Pharma Ltd) |
| 5154 | Beta2AdrenoceptorAgonistsSelective | 37443211000001101 | Seretide 250 Accuhaler (CST Pharma Ltd) |
| 5154 | Beta2AdrenoceptorAgonistsSelective | 37443411000001102 | Seretide 500 Accuhaler (CST Pharma Ltd) |
| 5154 | Beta2AdrenoceptorAgonistsSelective | 37443611000001104 | Seretide 250 Evohaler (CST Pharma Ltd) |
| 5154 | Beta2AdrenoceptorAgonistsSelective | 37443811000001100 | Oxis 6 Turbohaler (CST Pharma Ltd) |
| 5154 | Beta2AdrenoceptorAgonistsSelective | 37444011000001108 | Seretide 125 Evohaler (CST Pharma Ltd) |
| 5154 | Beta2AdrenoceptorAgonistsSelective | 37444211000001103 | Symbicort 400/12 Turbohaler (CST Pharma Ltd) |
| 5154 | Beta2AdrenoceptorAgonistsSelective | 37448711000001102 | Flutiform 250micrograms/dose / 10micrograms/dose inhaler (CST Pharma Ltd) |
| 5154 | Beta2AdrenoceptorAgonistsSelective | 37484711000001101 | Flutiform 250micrograms/dose / 10micrograms/dose inhaler (Mawdsley-Brooks & Company Ltd) |
| 5154 | Beta2AdrenoceptorAgonistsSelective | 37484911000001104 | Flutiform 125micrograms/dose / 5micrograms/dose inhaler (Mawdsley-Brooks & Company Ltd) |
| 5154 | Beta2AdrenoceptorAgonistsSelective | 37526111000001109 | Onbrez Breezhaler 150microgram inhalation powder capsules with device (CST Pharma Ltd) |
| 5154 | Beta2AdrenoceptorAgonistsSelective | 37526611000001101 | Onbrez Breezhaler 300microgram inhalation powder capsules with device (CST Pharma Ltd) |
| 5154 | Beta2AdrenoceptorAgonistsSelective | 37532911000001106 | Relvar Ellipta 184micrograms/dose / 22micrograms/dose dry powder inhaler (CST Pharma Ltd) |
| 5154 | Beta2AdrenoceptorAgonistsSelective | 37533111000001102 | Relvar Ellipta 92micrograms/dose / 22micrograms/dose dry powder inhaler (CST Pharma Ltd) |
| 5154 | Beta2AdrenoceptorAgonistsSelective | 37540311000001105 | Yanimo Respimat 2.5micrograms/dose / 2.5micrograms/dose inhalation solution cartridge with device (Boehringer Ingelheim Ltd) |
| 5154 | Beta2AdrenoceptorAgonistsSelective | 37549211000001109 | Onbrez Breezhaler 150microgram inhalation powder capsules with device (Mawdsley-Brooks & Company Ltd) |
| 5154 | Beta2AdrenoceptorAgonistsSelective | 37574711000001102 | Foradil 12microgram inhalation powder capsules with device (CST Pharma Ltd) |
| 5154 | Beta2AdrenoceptorAgonistsSelective | 37575111000001104 | Oxis 12 Turbohaler (CST Pharma Ltd) |
| 5154 | Beta2AdrenoceptorAgonistsSelective | 37576711000001106 | Bricanyl 500micrograms/dose Turbohaler (CST Pharma Ltd) |
| 5154 | Beta2AdrenoceptorAgonistsSelective | 37625611000001100 | Combivent nebuliser liquid 2.5ml UDVs (Pilsco Ltd) |
| 5154 | Beta2AdrenoceptorAgonistsSelective | 37631511000001104 | DuoResp Spiromax 160micrograms/dose / 4.5micrograms/dose dry powder inhaler (Ethigen Ltd) |
| 5154 | Beta2AdrenoceptorAgonistsSelective | 37631711000001109 | DuoResp Spiromax 320micrograms/dose / 9micrograms/dose dry powder inhaler (Ethigen Ltd) |
| 5154 | Beta2AdrenoceptorAgonistsSelective | 37665111000001104 | Flutiform 125micrograms/dose / 5micrograms/dose inhaler (Pilsco Ltd) |
| 5154 | Beta2AdrenoceptorAgonistsSelective | 37665311000001102 | Flutiform 250micrograms/dose / 10micrograms/dose inhaler (Pilsco Ltd) |
| 5154 | Beta2AdrenoceptorAgonistsSelective | 37678011000001103 | Spiolto Respimat 2.5micrograms/dose / 2.5micrograms/dose inhalation solution refill cartridge (Boehringer Ingelheim Ltd) |
| 5154 | Beta2AdrenoceptorAgonistsSelective | 37678311000001100 | Striverdi Respimat 2.5micrograms/dose inhalation solution refill cartridge (Boehringer Ingelheim Ltd) |
| 5154 | Beta2AdrenoceptorAgonistsSelective | 37692311000001106 | Olodaterol 2.5micrograms/dose inhalation solution cartridge CFC free |
| 5154 | Beta2AdrenoceptorAgonistsSelective | 37692511000001100 | Tiotropium bromide 2.5micrograms/dose / Olodaterol 2.5micrograms/dose inhalation solution cartridge CFC free |
| 5154 | Beta2AdrenoceptorAgonistsSelective | 37700511000001108 | Relvar Ellipta 184micrograms/dose / 22micrograms/dose dry powder inhaler (Pilsco Ltd) |
| 5154 | Beta2AdrenoceptorAgonistsSelective | 37700711000001103 | Relvar Ellipta 92micrograms/dose / 22micrograms/dose dry powder inhaler (Pilsco Ltd) |
| 5154 | Beta2AdrenoceptorAgonistsSelective | 37701911000001108 | Salamol 100micrograms/dose Easi-Breathe inhaler (Pilsco Ltd) |
| 5154 | Beta2AdrenoceptorAgonistsSelective | 37702211000001106 | Seretide 250 Accuhaler (Pilsco Ltd) |
| 5154 | Beta2AdrenoceptorAgonistsSelective | 37702411000001105 | Seretide 250 Evohaler (Pilsco Ltd) |
| 5154 | Beta2AdrenoceptorAgonistsSelective | 37702611000001108 | Seretide 500 Accuhaler (Pilsco Ltd) |
| 5154 | Beta2AdrenoceptorAgonistsSelective | 37702811000001107 | Serevent 50micrograms/dose Accuhaler (Pilsco Ltd) |
| 5154 | Beta2AdrenoceptorAgonistsSelective | 37714411000001100 | AirFluSal Forspiro 50micrograms/dose / 500micrograms/dose dry powder inhaler (Pilsco Ltd) |
| 5154 | Beta2AdrenoceptorAgonistsSelective | 37714611000001102 | AirFluSal 25micrograms/dose / 125micrograms/dose inhaler (Pilsco Ltd) |
| 5154 | Beta2AdrenoceptorAgonistsSelective | 37714811000001103 | AirFluSal 25micrograms/dose / 250micrograms/dose inhaler (Pilsco Ltd) |
| 5154 | Beta2AdrenoceptorAgonistsSelective | 37715611000001101 | Atimos Modulite 12micrograms/dose inhaler (Pilsco Ltd) |
| 5154 | Beta2AdrenoceptorAgonistsSelective | 37729411000001107 | Sereflo 25micrograms/dose / 250micrograms/dose inhaler (Pilsco Ltd) |
| 5154 | Beta2AdrenoceptorAgonistsSelective | 37739011000001101 | Symbicort 200/6 Turbohaler (CST Pharma Ltd) |
| 5154 | Beta2AdrenoceptorAgonistsSelective | 37979411000001103 | Anoro Ellipta 55micrograms/dose / 22micrograms/dose dry powder inhaler (Pharmaram Ltd) |
| 5154 | Beta2AdrenoceptorAgonistsSelective | 37994011000001101 | Relvar Ellipta 184micrograms/dose / 22micrograms/dose dry powder inhaler (Pharmaram Ltd) |
| 5154 | Beta2AdrenoceptorAgonistsSelective | 37994211000001106 | Relvar Ellipta 92micrograms/dose / 22micrograms/dose dry powder inhaler (Pharmaram Ltd) |
| 5154 | Beta2AdrenoceptorAgonistsSelective | 37996511000001103 | Seretide 125 Evohaler (Pharmaram Ltd) |
| 5154 | Beta2AdrenoceptorAgonistsSelective | 37996711000001108 | Seretide 250 Evohaler (Pharmaram Ltd) |
| 5154 | Beta2AdrenoceptorAgonistsSelective | 37997311000001107 | Symbicort 100/6 Turbohaler (Pharmaram Ltd) |
| 5154 | Beta2AdrenoceptorAgonistsSelective | 37997511000001101 | Symbicort 200/6 Turbohaler (Pharmaram Ltd) |
| 5154 | Beta2AdrenoceptorAgonistsSelective | 37997711000001106 | Symbicort 400/12 Turbohaler (Pharmaram Ltd) |
| 5154 | Beta2AdrenoceptorAgonistsSelective | 38130011000001108 | DuoResp Spiromax 160micrograms/dose / 4.5micrograms/dose dry powder inhaler (DE Pharmaceuticals) |
| 5154 | Beta2AdrenoceptorAgonistsSelective | 38130211000001103 | DuoResp Spiromax 320micrograms/dose / 9micrograms/dose dry powder inhaler (DE Pharmaceuticals) |
| 5154 | Beta2AdrenoceptorAgonistsSelective | 38131211000001109 | Easyhaler Salbutamol sulfate 100micrograms/dose dry powder inhaler (DE Pharmaceuticals) |
| 5154 | Beta2AdrenoceptorAgonistsSelective | 38131411000001108 | Easyhaler Salbutamol sulfate 200micrograms/dose dry powder inhaler (DE Pharmaceuticals) |
| 5154 | Beta2AdrenoceptorAgonistsSelective | 38134511000001109 | Flutiform 125micrograms/dose / 5micrograms/dose inhaler (DE Pharmaceuticals) |
| 5154 | Beta2AdrenoceptorAgonistsSelective | 38134711000001104 | Flutiform 250micrograms/dose / 10micrograms/dose inhaler (DE Pharmaceuticals) |
| 5154 | Beta2AdrenoceptorAgonistsSelective | 38134911000001102 | Flutiform 50micrograms/dose / 5micrograms/dose inhaler (DE Pharmaceuticals) |
| 5154 | Beta2AdrenoceptorAgonistsSelective | 38135111000001101 | Flutiform K-haler 125micrograms/dose / 5micrograms/dose breath actuated inhaler (DE Pharmaceuticals) |
| 5154 | Beta2AdrenoceptorAgonistsSelective | 38135311000001104 | Flutiform K-haler 50micrograms/dose / 5micrograms/dose breath actuated inhaler (DE Pharmaceuticals) |
| 5154 | Beta2AdrenoceptorAgonistsSelective | 38136011000001106 | Fostair NEXThaler 100micrograms/dose / 6micrograms/dose dry powder inhaler (DE Pharmaceuticals) |
| 5154 | Beta2AdrenoceptorAgonistsSelective | 38157111000001106 | Onbrez Breezhaler 150microgram inhalation powder capsules with device (DE Pharmaceuticals) |
| 5154 | Beta2AdrenoceptorAgonistsSelective | 38157311000001108 | Onbrez Breezhaler 300microgram inhalation powder capsules with device (DE Pharmaceuticals) |
| 5154 | Beta2AdrenoceptorAgonistsSelective | 38165311000001109 | Relvar Ellipta 92micrograms/dose / 22micrograms/dose dry powder inhaler (DE Pharmaceuticals) |
| 5154 | Beta2AdrenoceptorAgonistsSelective | 38165511000001103 | Relvar Ellipta 184micrograms/dose / 22micrograms/dose dry powder inhaler (DE Pharmaceuticals) |
| 5154 | Beta2AdrenoceptorAgonistsSelective | 38166911000001103 | Sirdupla 25micrograms/dose / 125micrograms/dose inhaler (DE Pharmaceuticals) |
| 5154 | Beta2AdrenoceptorAgonistsSelective | 38167111000001103 | Sirdupla 25micrograms/dose / 250micrograms/dose inhaler (DE Pharmaceuticals) |
| 5154 | Beta2AdrenoceptorAgonistsSelective | 38172811000001104 | Ventolin 5mg Nebules (DE Pharmaceuticals) |
| 5154 | Beta2AdrenoceptorAgonistsSelective | 38188711000001104 | Terbutaline 10mg/23ml solution for infusion pre-filled syringes (Special Order) |
| 5154 | Beta2AdrenoceptorAgonistsSelective | 38189211000001101 | Terbutaline 12mg/24ml solution for infusion pre-filled syringes (Special Order) |
| 5154 | Beta2AdrenoceptorAgonistsSelective | 38189511000001103 | Terbutaline 3mg/12ml solution for infusion pre-filled syringes (Special Order) |
| 5154 | Beta2AdrenoceptorAgonistsSelective | 38189911000001105 | Terbutaline 6mg/23ml solution for infusion pre-filled syringes (Special Order) |
| 5154 | Beta2AdrenoceptorAgonistsSelective | 38191511000001103 | Terbutaline 8mg/23ml solution for infusion pre-filled syringes (Special Order) |
| 5154 | Beta2AdrenoceptorAgonistsSelective | 38191811000001100 | Terbutaline 9mg/23ml solution for infusion pre-filled syringes (Special Order) |
| 5154 | Beta2AdrenoceptorAgonistsSelective | 38196411000001108 | Terbutaline 10mg/23ml solution for infusion pre-filled syringes |
| 5154 | Beta2AdrenoceptorAgonistsSelective | 38196511000001107 | Terbutaline 12mg/24ml solution for infusion pre-filled syringes |
| 5154 | Beta2AdrenoceptorAgonistsSelective | 38196611000001106 | Terbutaline 3mg/12ml solution for infusion pre-filled syringes |
| 5154 | Beta2AdrenoceptorAgonistsSelective | 38196711000001102 | Terbutaline 6mg/23ml solution for infusion pre-filled syringes |
| 5154 | Beta2AdrenoceptorAgonistsSelective | 38196811000001105 | Terbutaline 8mg/23ml solution for infusion pre-filled syringes |
| 5154 | Beta2AdrenoceptorAgonistsSelective | 38196911000001100 | Terbutaline 9mg/23ml solution for infusion pre-filled syringes |
| 5154 | Beta2AdrenoceptorAgonistsSelective | 38617811000001107 | Combiprasal 0.5mg/2.5mg nebuliser solution 2.5ml unit dose vials (TriOn Pharma Ltd) |
| 5154 | Beta2AdrenoceptorAgonistsSelective | 38640111000001109 | Sirdupla 25micrograms/dose / 125micrograms/dose inhaler (Pilsco Ltd) |
| 5154 | Beta2AdrenoceptorAgonistsSelective | 38640311000001106 | Sirdupla 25micrograms/dose / 250micrograms/dose inhaler (Pilsco Ltd) |
| 5154 | Beta2AdrenoceptorAgonistsSelective | 38895911000001101 | Salbutamol 500micrograms/1ml solution for injection ampoules |
| 5154 | Beta2AdrenoceptorAgonistsSelective | 38896811000001103 | Budesonide 200micrograms/dose / Formoterol 6micrograms/dose dry powder inhaler |
| 5154 | Beta2AdrenoceptorAgonistsSelective | 38897411000001103 | Fluticasone propionate 500micrograms/dose / Salmeterol 50micrograms/dose dry powder inhaler |
| 5154 | Beta2AdrenoceptorAgonistsSelective | 38897511000001104 | Fluticasone propionate 250micrograms/dose / Salmeterol 50micrograms/dose dry powder inhaler |
| 5154 | Beta2AdrenoceptorAgonistsSelective | 38897611000001100 | Fluticasone propionate 100micrograms/dose / Salmeterol 50micrograms/dose dry powder inhaler |
| 5154 | Beta2AdrenoceptorAgonistsSelective | 39025011000001108 | Salbutamol 4mg modified-release tablets |
| 5154 | Beta2AdrenoceptorAgonistsSelective | 39025111000001109 | Salbutamol 8mg modified-release capsules |
| 5154 | Beta2AdrenoceptorAgonistsSelective | 39025211000001103 | Salbutamol 4mg modified-release capsules |
| 5154 | Beta2AdrenoceptorAgonistsSelective | 39025311000001106 | Salbutamol 5mg/5ml solution for infusion ampoules |
| 5154 | Beta2AdrenoceptorAgonistsSelective | 39089311000001103 | Flutiform K-haler 125micrograms/dose / 5micrograms/dose breath actuated inhaler (Pilsco Ltd) |
| 5154 | Beta2AdrenoceptorAgonistsSelective | 39105811000001102 | Symbicort 100micrograms/dose / 3micrograms/dose pressurised inhaler (AstraZeneca UK Ltd) |
| 5154 | Beta2AdrenoceptorAgonistsSelective | 39109411000001101 | Salbutamol 2mg/5ml oral solution sugar free |
| 5154 | Beta2AdrenoceptorAgonistsSelective | 39110811000001105 | Salmeterol 25micrograms/dose inhaler |
| 5154 | Beta2AdrenoceptorAgonistsSelective | 39111011000001108 | Fluticasone 50micrograms/dose / Salmeterol 25micrograms/dose inhaler CFC free |
| 5154 | Beta2AdrenoceptorAgonistsSelective | 39111111000001109 | Fluticasone 125micrograms/dose / Salmeterol 25micrograms/dose inhaler CFC free |
| 5154 | Beta2AdrenoceptorAgonistsSelective | 39111311000001106 | Fluticasone 250micrograms/dose / Salmeterol 25micrograms/dose inhaler CFC free |
| 5154 | Beta2AdrenoceptorAgonistsSelective | 39112711000001103 | Salbutamol 100micrograms/dose breath actuated inhaler CFC free |
| 5154 | Beta2AdrenoceptorAgonistsSelective | 39112911000001101 | Formoterol 6micrograms/dose dry powder inhaler |
| 5154 | Beta2AdrenoceptorAgonistsSelective | 39113011000001109 | Formoterol 12micrograms/dose dry powder inhaler |
| 5154 | Beta2AdrenoceptorAgonistsSelective | 39113111000001105 | Terbutaline 250micrograms/dose inhaler |
| 5154 | Beta2AdrenoceptorAgonistsSelective | 39113211000001104 | Salmeterol 50micrograms/dose dry powder inhaler |
| 5154 | Beta2AdrenoceptorAgonistsSelective | 39113311000001107 | Salbutamol 200micrograms/dose dry powder inhaler |
| 5154 | Beta2AdrenoceptorAgonistsSelective | 39113611000001102 | Salbutamol 100micrograms/dose inhaler CFC free |
| 5154 | Beta2AdrenoceptorAgonistsSelective | 39114511000001103 | Atectura Breezhaler 125micrograms/62.5micrograms inhalation powder capsules with device (Sandoz Ltd) |
| 5154 | Beta2AdrenoceptorAgonistsSelective | 39115411000001101 | Atectura Breezhaler 125micrograms/127.5micrograms inhalation powder capsules with device (Sandoz Ltd) |
| 5154 | Beta2AdrenoceptorAgonistsSelective | 39115911000001109 | Atectura Breezhaler 125micrograms/260micrograms inhalation powder capsules with device (Sandoz Ltd) |
| 5154 | Beta2AdrenoceptorAgonistsSelective | 39116311000001103 | Indacaterol 125micrograms/dose / Mometasone 127.5micrograms/dose inhalation powder capsules with device |
| 5154 | Beta2AdrenoceptorAgonistsSelective | 39116411000001105 | Indacaterol 125micrograms/dose / Mometasone 260micrograms/dose inhalation powder capsules with device |
| 5154 | Beta2AdrenoceptorAgonistsSelective | 39116511000001109 | Indacaterol 125micrograms/dose / Mometasone 62.5micrograms/dose inhalation powder capsules with device |
| 5154 | Beta2AdrenoceptorAgonistsSelective | 39133611000001108 | Budesonide 100micrograms/dose / Formoterol 3micrograms/dose inhaler CFC free |
| 5154 | Beta2AdrenoceptorAgonistsSelective | 39134511000001107 | Generic Enerzair Breezhaler 114micrograms/dose / 46micrograms/dose / 136micrograms/dose inhalation powder capsules with device |
| 5154 | Beta2AdrenoceptorAgonistsSelective | 39134711000001102 | Enerzair Breezhaler 114micrograms/dose / 46micrograms/dose / 136micrograms/dose inhalation powder capsules with device (Sandoz Ltd) |
| 5154 | Beta2AdrenoceptorAgonistsSelective | 39241911000001100 | Salbutamol 2mg tablets (Medihealth (Northern) Ltd) |
| 5154 | Beta2AdrenoceptorAgonistsSelective | 39242111000001108 | Salbutamol 4mg tablets (Medihealth (Northern) Ltd) |
| 5154 | Beta2AdrenoceptorAgonistsSelective | 39327311000001104 | Trixeo Aerosphere 5micrograms/dose / 7.2micrograms/dose / 160micrograms/dose pressurised inhaler (AstraZeneca UK Ltd) |
| 5154 | Beta2AdrenoceptorAgonistsSelective | 39329111000001107 | Bevespi Aerosphere 7.2micrograms/dose / 5micrograms/dose pressurised inhaler (AstraZeneca UK Ltd) |
| 5154 | Beta2AdrenoceptorAgonistsSelective | 39343511000001103 | Generic Trixeo Aerosphere 5micrograms/dose / 7.2micrograms/dose / 160micrograms/dose inhaler CFC free |
| 5154 | Beta2AdrenoceptorAgonistsSelective | 39343611000001104 | Glycopyrronium 7.2micrograms/dose / Formoterol 5micrograms/dose inhaler CFC free |
| 5154 | Beta2AdrenoceptorAgonistsSelective | 39359011000001107 | Bricanyl 500micrograms/dose Turbohaler (Pilsco Ltd) |
| 5154 | Beta2AdrenoceptorAgonistsSelective | 39360211000001103 | Fostair NEXThaler 100micrograms/dose / 6micrograms/dose dry powder inhaler (Pilsco Ltd) |
| 5154 | Beta2AdrenoceptorAgonistsSelective | 39360411000001104 | Sereflo 25micrograms/dose / 125micrograms/dose inhaler (Pilsco Ltd) |
| 5154 | Beta2AdrenoceptorAgonistsSelective | 39567411000001102 | Fixkoh Airmaster 50micrograms/dose / 500micrograms/dose dry powder inhaler (Thornton & Ross Ltd) |
| 5154 | Beta2AdrenoceptorAgonistsSelective | 39567611000001104 | Fixkoh Airmaster 50micrograms/dose / 250micrograms/dose dry powder inhaler (Thornton & Ross Ltd) |
| 5154 | Beta2AdrenoceptorAgonistsSelective | 39567811000001100 | Fixkoh Airmaster 50micrograms/dose / 100micrograms/dose dry powder inhaler (Thornton & Ross Ltd) |
| 5154 | Beta2AdrenoceptorAgonistsSelective | 39696311000001100 | Salbutamol 400microgram / Beclometasone 200microgram inhalation powder capsules |
| 5154 | Beta2AdrenoceptorAgonistsSelective | 39703611000001107 | Salbutamol 4mg tablets |
| 5154 | Beta2AdrenoceptorAgonistsSelective | 39709511000001105 | Salbutamol 5mg/ml nebuliser liquid |
| 5154 | Beta2AdrenoceptorAgonistsSelective | 39709611000001109 | Salbutamol 2.5mg/2.5ml nebuliser liquid unit dose vials |
| 5154 | Beta2AdrenoceptorAgonistsSelective | 39709711000001100 | Salbutamol 200microgram inhalation powder capsules |
| 5154 | Beta2AdrenoceptorAgonistsSelective | 39709811000001108 | Salbutamol 400microgram inhalation powder capsules |
| 5154 | Beta2AdrenoceptorAgonistsSelective | 39710011000001108 | Salbutamol 5mg/2.5ml nebuliser liquid unit dose vials |
| 5154 | Beta2AdrenoceptorAgonistsSelective | 39710511000001100 | Salbutamol 2.5mg/2.5ml / Ipratropium bromide 500micrograms/2.5ml nebuliser liquid unit dose vials |
| 5154 | Beta2AdrenoceptorAgonistsSelective | 39711511000001107 | Fenoterol 1.25mg/4ml / Ipratropium 500micrograms/4ml nebuliser liquid unit dose vials |
| 5154 | Beta2AdrenoceptorAgonistsSelective | 39714711000001104 | Terbutaline 10mg/ml nebuliser liquid |
| 5154 | Beta2AdrenoceptorAgonistsSelective | 39817511000001103 | Luforbec 100micrograms/dose / 6micrograms/dose inhaler (Lupin Healthcare (UK) Ltd) |
| 5154 | Beta2AdrenoceptorAgonistsSelective | 39939611000001107 | Fostair 100micrograms/dose / 6micrograms/dose inhaler (CST Pharma Ltd) |
| 5154 | Beta2AdrenoceptorAgonistsSelective | 39993311000001105 | Trimbow NEXThaler 88micrograms/dose / 5micrograms/dose / 9micrograms/dose dry powder inhaler (Chiesi Ltd) |
| 5154 | Beta2AdrenoceptorAgonistsSelective | 40034211000001104 | Avenor 25micrograms/dose / 50micrograms/dose inhaler (Zentiva Pharma UK Ltd) |
| 5154 | Beta2AdrenoceptorAgonistsSelective | 40040711000001101 | Avenor 25micrograms/dose / 125micrograms/dose inhaler (Zentiva Pharma UK Ltd) |
| 5154 | Beta2AdrenoceptorAgonistsSelective | 40040911000001104 | Avenor 25micrograms/dose / 250micrograms/dose inhaler (Zentiva Pharma UK Ltd) |
| 5154 | Beta2AdrenoceptorAgonistsSelective | 40087411000001104 | Generic Trimbow NEXThaler 88micrograms/dose / 5micrograms/dose / 9micrograms/dose dry powder inhaler |
| 5154 | Beta2AdrenoceptorAgonistsSelective | 40106011000001102 | WockAIR 160micrograms/dose / 4.5micrograms/dose dry powder inhaler (Wockhardt UK Ltd) |
| 5154 | Beta2AdrenoceptorAgonistsSelective | 40106211000001107 | WockAIR 320micrograms/dose / 9micrograms/dose dry powder inhaler (Wockhardt UK Ltd) |
| 5154 | Beta2AdrenoceptorAgonistsSelective | 40220711000001106 | Seretide 50 Evohaler (CST Pharma Ltd) |
| 5154 | Beta2AdrenoceptorAgonistsSelective | 40220911000001108 | Fostair NEXThaler 100micrograms/dose / 6micrograms/dose dry powder inhaler (CST Pharma Ltd) |
| 5154 | Beta2AdrenoceptorAgonistsSelective | 40444911000001101 | Seffalair Spiromax 12.75micrograms/dose / 202micrograms/dose dry powder inhaler (Teva UK Ltd) |
| 5154 | Beta2AdrenoceptorAgonistsSelective | 40445111000001100 | Seffalair Spiromax 12.75micrograms/dose / 100micrograms/dose dry powder inhaler (Teva UK Ltd) |
| 5154 | Beta2AdrenoceptorAgonistsSelective | 40455711000001101 | Fluticasone propionate 100micrograms/dose / Salmeterol 12.75micrograms/dose dry powder inhaler |
| 5154 | Beta2AdrenoceptorAgonistsSelective | 40455811000001109 | Fluticasone propionate 202micrograms/dose / Salmeterol 12.75micrograms/dose dry powder inhaler |
| 5154 | Beta2AdrenoceptorAgonistsSelective | 40504911000001103 | Sereflo Ciphaler 50micrograms/dose / 250micrograms/dose dry powder inhaler (Cipla EU Ltd) |
| 5154 | Beta2AdrenoceptorAgonistsSelective | 40752211000001109 | Trimbow 172micrograms/dose / 5micrograms/dose / 9micrograms/dose inhaler (Chiesi Ltd) |
| 5154 | Beta2AdrenoceptorAgonistsSelective | 40766811000001104 | Generic Trimbow 172micrograms/dose / 5micrograms/dose / 9micrograms/dose inhaler |
| 5154 | Beta2AdrenoceptorAgonistsSelective | 40852311000001103 | Luforbec 200micrograms/dose / 6micrograms/dose inhaler (Lupin Healthcare (UK) Ltd) |
| 5154 | Beta2AdrenoceptorAgonistsSelective | 134498003 | Budesonide+eformoterol fumarate 200/6mcg breath-actuated dry powder inhaler |
| 5154 | Beta2AdrenoceptorAgonistsSelective | 134499006 | Budesonide 100micrograms/dose / Formoterol 6micrograms/dose dry powder inhaler |
| 5154 | Beta2AdrenoceptorAgonistsSelective | 320096005 | Salbutamol 4mg m/r tablet |
| 5154 | Beta2AdrenoceptorAgonistsSelective | 320099003 | Salbutamol 4mg m/r capsule |
| 5154 | Beta2AdrenoceptorAgonistsSelective | 320100006 | Salbutamol 8mg m/r capsule |
| 5154 | Beta2AdrenoceptorAgonistsSelective | 320104002 | Salbutamol 4mg tablets |
| 5154 | Beta2AdrenoceptorAgonistsSelective | 320106000 | Salbutamol 2mg tablets |
| 5154 | Beta2AdrenoceptorAgonistsSelective | 320107009 | Salbutamol 8mg modified-release tablets |
| 5154 | Beta2AdrenoceptorAgonistsSelective | 320108004 | Salbutamol 2mg/5mL sugar free syrup |
| 5154 | Beta2AdrenoceptorAgonistsSelective | 320116008 | Salbutamol 5mg/50ml solution for infusion vials |
| 5154 | Beta2AdrenoceptorAgonistsSelective | 320118009 | Salbutamol 500micrograms/1mL injection |
| 5154 | Beta2AdrenoceptorAgonistsSelective | 320119001 | Salbutamol 5mg/5mL intravenous infusion |
| 5154 | Beta2AdrenoceptorAgonistsSelective | 320132006 | Salbutamol 200microgram inhalation powder blisters with device |
| 5154 | Beta2AdrenoceptorAgonistsSelective | 320133001 | Salbutamol 400microgram inhalation powder blisters with device |
| 5154 | Beta2AdrenoceptorAgonistsSelective | 320134007 | Salbutamol 200microgram inhalation powder blisters |
| 5154 | Beta2AdrenoceptorAgonistsSelective | 320135008 | Salbutamol 400microgram inhalation powder blisters |
| 5154 | Beta2AdrenoceptorAgonistsSelective | 320136009 | Salbutamol 100micrograms/dose breath actuated inhaler |
| 5154 | Beta2AdrenoceptorAgonistsSelective | 320139002 | Salbutamol 100micrograms CFC-free inhaler |
| 5154 | Beta2AdrenoceptorAgonistsSelective | 320141001 | Salbutamol 200micrograms breath-actuated dry powder inhaler |
| 5154 | Beta2AdrenoceptorAgonistsSelective | 320148007 | Salbutamol 95micrograms/dose dry powder inhaler |
| 5154 | Beta2AdrenoceptorAgonistsSelective | 320151000 | Salbutamol 100micrograms CFC-free breath-actuated aerosol inhaler |
| 5154 | Beta2AdrenoceptorAgonistsSelective | 320171009 | Salbutamol 5mg/2.5ml nebuliser liquid unit dose vials |
| 5154 | Beta2AdrenoceptorAgonistsSelective | 320176004 | Salbutamol 100micrograms/dose inhaler |
| 5154 | Beta2AdrenoceptorAgonistsSelective | 320177008 | Salbutamol 2.5mg/2.5ml nebuliser liquid unit dose vials |
| 5154 | Beta2AdrenoceptorAgonistsSelective | 320178003 | Salbutamol 200microgram inhalation powder capsules |
| 5154 | Beta2AdrenoceptorAgonistsSelective | 320179006 | Salbutamol 400microgram inhalation powder capsules |
| 5154 | Beta2AdrenoceptorAgonistsSelective | 320180009 | Salbutamol 5mg/ml nebuliser liquid |
| 5154 | Beta2AdrenoceptorAgonistsSelective | 320199000 | Terbutaline 2.5mg/5ml solution for injection ampoules |
| 5154 | Beta2AdrenoceptorAgonistsSelective | 320200002 | Terbutaline 10mg/ml nebuliser liquid |
| 5154 | Beta2AdrenoceptorAgonistsSelective | 320201003 | Terbutaline 500micrograms/dose dry powder inhaler |
| 5154 | Beta2AdrenoceptorAgonistsSelective | 320203000 | Terbutaline 5mg tablets |
| 5154 | Beta2AdrenoceptorAgonistsSelective | 320205007 | Terbutaline 500micrograms/1ml solution for injection ampoules |
| 5154 | Beta2AdrenoceptorAgonistsSelective | 320206008 | Terbutaline sulfate 250micrograms inhaler |
| 5154 | Beta2AdrenoceptorAgonistsSelective | 320208009 | Terbutaline 250micrograms/dose inhaler with spacer |
| 5154 | Beta2AdrenoceptorAgonistsSelective | 320211005 | Terbutaline 7.5mg modified-release tablets |
| 5154 | Beta2AdrenoceptorAgonistsSelective | 320212003 | Terbutaline 1.5mg/5ml oral solution sugar free |
| 5154 | Beta2AdrenoceptorAgonistsSelective | 320244004 | Salmeterol 25mcg inhaler |
| 5154 | Beta2AdrenoceptorAgonistsSelective | 320248001 | Salmeterol 50microgram inhalation powder blisters with device |
| 5154 | Beta2AdrenoceptorAgonistsSelective | 320249009 | Salmeterol 50microgram inhalation powder blisters |
| 5154 | Beta2AdrenoceptorAgonistsSelective | 320250009 | Salmeterol 50micrograms breath-actuated dry powder inhaler |
| 5154 | Beta2AdrenoceptorAgonistsSelective | 320258002 | Bambuterol 10mg tablets |
| 5154 | Beta2AdrenoceptorAgonistsSelective | 320259005 | Bambuterol 20mg tablets |
| 5154 | Beta2AdrenoceptorAgonistsSelective | 320263003 | Eformoterol fumarate dihydrate 6micrograms breath-actuated dry powder inhaler |
| 5154 | Beta2AdrenoceptorAgonistsSelective | 320264009 | Eformoterol fumarate dihydrate 12micrograms breath-actuated dry powder inhaler |
| 5154 | Beta2AdrenoceptorAgonistsSelective | 320274007 | Salmeterol+fluticasone propionate 25micrograms/50micrograms CFC-free inhaler |
| 5154 | Beta2AdrenoceptorAgonistsSelective | 320275008 | Salmeterol+fluticasone propionate 25micrograms/125micrograms CFC-free inhaler |
| 5154 | Beta2AdrenoceptorAgonistsSelective | 320276009 | Salmeterol+fluticasone propionate 25micrograms/250micrograms CFC-free inhaler |
| 5154 | Beta2AdrenoceptorAgonistsSelective | 320277000 | Salmeterol+fluticasone propionate 50micrograms/100micrograms breath-actuated dry powder inhaler |
| 5154 | Beta2AdrenoceptorAgonistsSelective | 320279002 | Salmeterol+fluticasone propionate 50micrograms/250micrograms breath-actuated dry powder inhaler |
| 5154 | Beta2AdrenoceptorAgonistsSelective | 320280004 | Salmeterol+fluticasone propionate 50micrograms/500micrograms breath-actuated dry powder inhaler |
| 5154 | Beta2AdrenoceptorAgonistsSelective | 320440005 | Fenoterol 100micrograms/dose / Ipratropium 40micrograms/dose inhaler |
| 5154 | Beta2AdrenoceptorAgonistsSelective | 320441009 | Fenoterol 100micrograms/dose / Ipratropium bromide 40micrograms/dose breath actuated inhaler |
| 5154 | Beta2AdrenoceptorAgonistsSelective | 320442002 | Salbutamol 100micrograms/dose / Ipratropium 20micrograms/dose inhaler |
| 5154 | Beta2AdrenoceptorAgonistsSelective | 320445000 | Salbutamol 2.5mg/2.5ml / Ipratropium bromide 500micrograms/2.5ml nebuliser liquid unit dose vials |
| 5154 | Beta2AdrenoceptorAgonistsSelective | 320543009 | Salbutamol 400microgram / Beclometasone 200microgram inhalation powder capsules |
| 5154 | Beta2AdrenoceptorAgonistsSelective | 320544003 | Salbutamol 200microgram / Beclometasone 100microgram inhalation powder capsules |
| 5154 | Beta2AdrenoceptorAgonistsSelective | 320545002 | Salbutamol 100micrograms/dose / Beclometasone 50micrograms/dose inhaler |
| 5154 | Beta2AdrenoceptorAgonistsSelective | 320660007 | Sodium cromoglicate 1mg/dose / Salbutamol 100micrograms/dose inhaler with spacer |
| 5154 | Beta2AdrenoceptorAgonistsSelective | 320661006 | Sodium cromoglicate 1mg/dose / Salbutamol 100micrograms/dose inhaler |
| 5154 | Beta2AdrenoceptorAgonistsSelective | 349394001 | Fenoterol 1.25mg/4ml / Ipratropium 500micrograms/4ml nebuliser liquid unit dose vials |
| 5154 | Beta2AdrenoceptorAgonistsSelective | 7511000001105 | Salbutamol 100micrograms/dose inhaler CFC free (Actavis UK Ltd) |
| 5154 | Beta2AdrenoceptorAgonistsSelective | 28011000001107 | Monovent 1.5mg/5ml syrup (Sandoz Ltd) |
| 5154 | Beta2AdrenoceptorAgonistsSelective | 45111000001100 | Salbutamol 100micrograms/dose inhaler CFC free (Viatris UK Healthcare Ltd) |
| 5154 | Beta2AdrenoceptorAgonistsSelective | 52811000001102 | Salbutamol 4mg tablets (Alliance Healthcare (Distribution) Ltd) |
| 5154 | Beta2AdrenoceptorAgonistsSelective | 101011000001104 | Bricanyl 2.5mg/5ml solution for injection ampoules (AstraZeneca UK Ltd) |
| 5154 | Beta2AdrenoceptorAgonistsSelective | 106511000001103 | Salamol 100micrograms/dose inhaler CFC free (Teva UK Ltd) |
| 5154 | Beta2AdrenoceptorAgonistsSelective | 109751000001104 | Fenoterol 1.25mg/4ml / Ipratropium 500micrograms/4ml nebuliser liquid unit dose vials |
| 5154 | Beta2AdrenoceptorAgonistsSelective | 110651000001100 | Salbutamol 2.5mg/2.5ml / Ipratropium bromide 500micrograms/2.5ml nebuliser liquid unit dose vials |
| 5154 | Beta2AdrenoceptorAgonistsSelective | 111271000001104 | Salbutamol 5mg/ml nebuliser liquid |
| 5154 | Beta2AdrenoceptorAgonistsSelective | 111351000001100 | Salbutamol 5mg/2.5ml nebuliser liquid unit dose vials |
| 5154 | Beta2AdrenoceptorAgonistsSelective | 111431000001100 | Salbutamol 4mg tablets |
| 5154 | Beta2AdrenoceptorAgonistsSelective | 111961000001101 | Salbutamol 2.5mg/2.5ml nebuliser liquid unit dose vials |
| 5154 | Beta2AdrenoceptorAgonistsSelective | 112121000001102 | Salbutamol 400microgram / Beclometasone 200microgram inhalation powder capsules |
| 5154 | Beta2AdrenoceptorAgonistsSelective | 112651000001104 | Salbutamol 200microgram inhalation powder capsules |
| 5154 | Beta2AdrenoceptorAgonistsSelective | 113271000001103 | Salbutamol 400microgram inhalation powder capsules |
| 5154 | Beta2AdrenoceptorAgonistsSelective | 113751000001100 | Terbutaline 10mg/ml nebuliser liquid |
| 5154 | Beta2AdrenoceptorAgonistsSelective | 161111000001102 | Salbutamol 2mg/5ml oral solution sugar free (A A H Pharmaceuticals Ltd) |
| 5154 | Beta2AdrenoceptorAgonistsSelective | 161811000001109 | Salbutamol 2mg tablets (Kent Pharma (UK) Ltd) |
| 5154 | Beta2AdrenoceptorAgonistsSelective | 189411000001105 | Salbutamol 100micrograms/dose inhaler CFC free (Alliance Healthcare (Distribution) Ltd) |
| 5154 | Beta2AdrenoceptorAgonistsSelective | 194411000001107 | Salbutamol 4mg tablets (Accord Healthcare Ltd) |
| 5154 | Beta2AdrenoceptorAgonistsSelective | 196411000001104 | Volmax 4mg modified-release tablets (GlaxoSmithKline UK Ltd) |
| 5154 | Beta2AdrenoceptorAgonistsSelective | 221511000001100 | Salbutamol 2mg/5ml oral solution sugar free (Sandoz Ltd) |
| 5154 | Beta2AdrenoceptorAgonistsSelective | 222311000001102 | Ventolin 100micrograms/dose Evohaler (GlaxoSmithKline UK Ltd) |
| 5154 | Beta2AdrenoceptorAgonistsSelective | 225511000001102 | Terbutaline 1.5mg/5ml oral solution sugar free (Kent Pharma (UK) Ltd) |
| 5154 | Beta2AdrenoceptorAgonistsSelective | 248411000001109 | Terbutaline 1.5mg/5ml oral solution sugar free (Alliance Healthcare (Distribution) Ltd) |
| 5154 | Beta2AdrenoceptorAgonistsSelective | 258311000001100 | Salbutamol 4mg tablets (Kent Pharma (UK) Ltd) |
| 5154 | Beta2AdrenoceptorAgonistsSelective | 262711000001105 | Salbutamol 4mg tablets (A A H Pharmaceuticals Ltd) |
| 5154 | Beta2AdrenoceptorAgonistsSelective | 273911000001107 | Salbutamol 4mg tablets (Approved Prescription Services Ltd) |
| 5154 | Beta2AdrenoceptorAgonistsSelective | 281511000001105 | Ventmax SR 8mg capsules (Chiesi Ltd) |
| 5154 | Beta2AdrenoceptorAgonistsSelective | 287611000001100 | Salbutamol 2mg tablets (Approved Prescription Services Ltd) |
| 5154 | Beta2AdrenoceptorAgonistsSelective | 364811000001103 | Bricanyl SA 7.5mg tablets (AstraZeneca UK Ltd) |
| 5154 | Beta2AdrenoceptorAgonistsSelective | 375711000001100 | Bambec 10mg tablets (AstraZeneca UK Ltd) |
| 5154 | Beta2AdrenoceptorAgonistsSelective | 408911000001105 | Serevent 25micrograms/dose inhaler (GlaxoSmithKline UK Ltd) |
| 5154 | Beta2AdrenoceptorAgonistsSelective | 453611000001102 | Seretide 50 Evohaler (GlaxoSmithKline UK Ltd) |
| 5154 | Beta2AdrenoceptorAgonistsSelective | 506611000001108 | Salbutamol 2mg tablets (Accord Healthcare Ltd) |
| 5154 | Beta2AdrenoceptorAgonistsSelective | 539811000001106 | Seretide 250 Evohaler (GlaxoSmithKline UK Ltd) |
| 5154 | Beta2AdrenoceptorAgonistsSelective | 571311000001104 | Salbutamol 100micrograms/dose inhaler CFC free (A A H Pharmaceuticals Ltd) |
| 5154 | Beta2AdrenoceptorAgonistsSelective | 597011000001101 | Airomir 100micrograms/dose inhaler (Teva UK Ltd) |
| 5154 | Beta2AdrenoceptorAgonistsSelective | 626411000001107 | Ventmax SR 4mg capsules (Chiesi Ltd) |
| 5154 | Beta2AdrenoceptorAgonistsSelective | 650211000001107 | Salapin 2mg/5ml syrup (Pinewood Healthcare) |
| 5154 | Beta2AdrenoceptorAgonistsSelective | 659111000001106 | Salbutamol 2mg tablets (A A H Pharmaceuticals Ltd) |
| 5154 | Beta2AdrenoceptorAgonistsSelective | 691211000001105 | Bricanyl 500micrograms/1ml solution for injection ampoules (AstraZeneca UK Ltd) |
| 5154 | Beta2AdrenoceptorAgonistsSelective | 726111000001105 | Bricanyl 1.5mg/5ml syrup (AstraZeneca UK Ltd) |
| 5154 | Beta2AdrenoceptorAgonistsSelective | 738811000001100 | Bricanyl 5mg tablets (AstraZeneca UK Ltd) |
| 5154 | Beta2AdrenoceptorAgonistsSelective | 757611000001104 | Salbutamol 100micrograms/dose inhaler CFC free (Teva UK Ltd) |
| 5154 | Beta2AdrenoceptorAgonistsSelective | 803911000001107 | Salbutamol 2mg/5ml oral solution sugar free (Alliance Healthcare (Distribution) Ltd) |
| 5154 | Beta2AdrenoceptorAgonistsSelective | 809411000001102 | Bambec 20mg tablets (AstraZeneca UK Ltd) |
| 5154 | Beta2AdrenoceptorAgonistsSelective | 810211000001105 | Seretide 125 Evohaler (GlaxoSmithKline UK Ltd) |
| 5154 | Beta2AdrenoceptorAgonistsSelective | 831811000001109 | Volmax 8mg modified-release tablets (GlaxoSmithKline UK Ltd) |
| 5154 | Beta2AdrenoceptorAgonistsSelective | 840111000001107 | Salbulin 100micrograms/dose inhaler (3M Health Care Ltd) |
| 5154 | Beta2AdrenoceptorAgonistsSelective | 886111000001103 | Ventolin 5mg/5ml solution for infusion ampoules (GlaxoSmithKline UK Ltd) |
| 5154 | Beta2AdrenoceptorAgonistsSelective | 892011000001102 | Ventolin 2mg/5ml syrup (GlaxoSmithKline UK Ltd) |
| 5154 | Beta2AdrenoceptorAgonistsSelective | 896511000001106 | Salbutamol 2mg/5ml oral solution sugar free (Kent Pharma (UK) Ltd) |
| 5154 | Beta2AdrenoceptorAgonistsSelective | 921311000001107 | Salbutamol 2mg tablets (Alliance Healthcare (Distribution) Ltd) |
| 5154 | Beta2AdrenoceptorAgonistsSelective | 2831711000001102 | Bricanyl 10mg/ml respirator solution (AstraZeneca UK Ltd) |
| 5154 | Beta2AdrenoceptorAgonistsSelective | 2923111000001107 | Duovent inhaler (Boehringer Ingelheim Ltd) |
| 5154 | Beta2AdrenoceptorAgonistsSelective | 2964011000001104 | Duovent Autohaler (Boehringer Ingelheim Ltd) |
| 5154 | Beta2AdrenoceptorAgonistsSelective | 3080411000001101 | Ventodisks 200microgram with Diskhaler (GlaxoSmithKline UK Ltd) |
| 5154 | Beta2AdrenoceptorAgonistsSelective | 3082411000001100 | Serevent 50microgram disks (GlaxoSmithKline UK Ltd) |
| 5154 | Beta2AdrenoceptorAgonistsSelective | 3083011000001100 | Ventodisks 400microgram with Diskhaler (GlaxoSmithKline UK Ltd) |
| 5154 | Beta2AdrenoceptorAgonistsSelective | 3084011000001103 | Serevent 50microgram disks with Diskhaler (GlaxoSmithKline UK Ltd) |
| 5154 | Beta2AdrenoceptorAgonistsSelective | 3086111000001109 | Ventodisks 200microgram (GlaxoSmithKline UK Ltd) |
| 5154 | Beta2AdrenoceptorAgonistsSelective | 3089011000001102 | Ventodisks 400microgram (GlaxoSmithKline UK Ltd) |
| 5154 | Beta2AdrenoceptorAgonistsSelective | 3186011000001104 | Asmasal 95micrograms/dose Clickhaler (Focus Pharmaceuticals Ltd) |
| 5154 | Beta2AdrenoceptorAgonistsSelective | 3186911000001100 | Seretide 100 Accuhaler (GlaxoSmithKline UK Ltd) |
| 5154 | Beta2AdrenoceptorAgonistsSelective | 3187211000001106 | Seretide 250 Accuhaler (GlaxoSmithKline UK Ltd) |
| 5154 | Beta2AdrenoceptorAgonistsSelective | 3188311000001102 | Seretide 500 Accuhaler (GlaxoSmithKline UK Ltd) |
| 5154 | Beta2AdrenoceptorAgonistsSelective | 3200511000001109 | Ventide Paediatric Rotacaps (GlaxoSmithKline UK Ltd) |
| 5154 | Beta2AdrenoceptorAgonistsSelective | 3202211000001107 | Ventide Rotacaps (GlaxoSmithKline UK Ltd) |
| 5154 | Beta2AdrenoceptorAgonistsSelective | 3206811000001109 | Foradil 12microgram inhalation powder capsules with device (Novartis Pharmaceuticals UK Ltd) |
| 5154 | Beta2AdrenoceptorAgonistsSelective | 3214211000001100 | Ventolin 200microgram Rotacaps (GlaxoSmithKline UK Ltd) |
| 5154 | Beta2AdrenoceptorAgonistsSelective | 3214311000001108 | Airomir 100micrograms/dose Autohaler (Teva UK Ltd) |
| 5154 | Beta2AdrenoceptorAgonistsSelective | 3214611000001103 | Salbutamol 200 Cyclocaps (Teva UK Ltd) |
| 5154 | Beta2AdrenoceptorAgonistsSelective | 3215311000001107 | Salamol 100micrograms/dose Easi-Breathe inhaler (Teva UK Ltd) |
| 5154 | Beta2AdrenoceptorAgonistsSelective | 3217611000001109 | Salbutamol 400 Cyclocaps (Teva UK Ltd) |
| 5154 | Beta2AdrenoceptorAgonistsSelective | 3218011000001101 | Ventolin 400microgram Rotacaps (GlaxoSmithKline UK Ltd) |
| 5154 | Beta2AdrenoceptorAgonistsSelective | 3218311000001103 | Bricanyl 500micrograms/dose Turbohaler (AstraZeneca UK Ltd) |
| 5154 | Beta2AdrenoceptorAgonistsSelective | 3235011000001104 | Aerocrom inhaler (Castlemead Healthcare Ltd) |
| 5154 | Beta2AdrenoceptorAgonistsSelective | 3243511000001107 | Oxis 6 Turbohaler (AstraZeneca UK Ltd) |
| 5154 | Beta2AdrenoceptorAgonistsSelective | 3245011000001103 | Oxis 12 Turbohaler (AstraZeneca UK Ltd) |
| 5154 | Beta2AdrenoceptorAgonistsSelective | 3248011000001106 | Bricanyl 250micrograms/dose inhaler (AstraZeneca UK Ltd) |
| 5154 | Beta2AdrenoceptorAgonistsSelective | 3248711000001108 | Bricanyl 250micrograms/dose spacer inhaler (AstraZeneca UK Ltd) |
| 5154 | Beta2AdrenoceptorAgonistsSelective | 3292811000001106 | Ventide inhaler (GlaxoSmithKline UK Ltd) |
| 5154 | Beta2AdrenoceptorAgonistsSelective | 3293111000001105 | Aerolin 100micrograms/dose Autohaler (3M Health Care Ltd) |
| 5154 | Beta2AdrenoceptorAgonistsSelective | 3294211000001101 | Symbicort 100/6 Turbohaler (AstraZeneca UK Ltd) |
| 5154 | Beta2AdrenoceptorAgonistsSelective | 3294611000001104 | Symbicort 200/6 Turbohaler (AstraZeneca UK Ltd) |
| 5154 | Beta2AdrenoceptorAgonistsSelective | 3348611000001107 | Combivent inhaler (Boehringer Ingelheim Ltd) |
| 5154 | Beta2AdrenoceptorAgonistsSelective | 3379611000001104 | Salbutamol 5mg/2.5ml nebuliser liquid unit dose vials (A A H Pharmaceuticals Ltd) |
| 5154 | Beta2AdrenoceptorAgonistsSelective | 3380111000001107 | Serevent 50micrograms/dose Accuhaler (GlaxoSmithKline UK Ltd) |
| 5154 | Beta2AdrenoceptorAgonistsSelective | 3380811000001100 | Maxivent 5mg/2.5ml nebuliser liquid unit dose Steripoule vials (Ashbourne Pharmaceuticals Ltd) |
| 5154 | Beta2AdrenoceptorAgonistsSelective | 3381111000001101 | Salbutamol 5mg/2.5ml nebuliser liquid unit dose vials (Viatris UK Healthcare Ltd) |
| 5154 | Beta2AdrenoceptorAgonistsSelective | 3381811000001108 | Ventolin 5mg Nebules (GlaxoSmithKline UK Ltd) |
| 5154 | Beta2AdrenoceptorAgonistsSelective | 3382711000001107 | Ventolin 200micrograms/dose Accuhaler (GlaxoSmithKline UK Ltd) |
| 5154 | Beta2AdrenoceptorAgonistsSelective | 3383511000001109 | Salamol 5mg/2.5ml nebuliser liquid Steri-Neb unit dose vials (Teva UK Ltd) |
| 5154 | Beta2AdrenoceptorAgonistsSelective | 3384111000001103 | Pulvinal Salbutamol 200micrograms/dose dry powder inhaler (Chiesi Ltd) |
| 5154 | Beta2AdrenoceptorAgonistsSelective | 3385711000001103 | Salbutamol 2.5mg/2.5ml nebuliser liquid unit dose vials (A A H Pharmaceuticals Ltd) |
| 5154 | Beta2AdrenoceptorAgonistsSelective | 3386411000001100 | Maxivent 2.5mg/2.5ml nebuliser liquid unit dose Steripoule vials (Ashbourne Pharmaceuticals Ltd) |
| 5154 | Beta2AdrenoceptorAgonistsSelective | 3386811000001103 | Salbutamol 2.5mg/2.5ml nebuliser liquid unit dose vials (Viatris UK Healthcare Ltd) |
| 5154 | Beta2AdrenoceptorAgonistsSelective | 3387011000001107 | Ventolin 2.5mg Nebules (GlaxoSmithKline UK Ltd) |
| 5154 | Beta2AdrenoceptorAgonistsSelective | 3387511000001104 | Salamol 2.5mg/2.5ml nebuliser liquid Steri-Neb unit dose vials (Teva UK Ltd) |
| 5154 | Beta2AdrenoceptorAgonistsSelective | 3406011000001104 | Combivent nebuliser liquid 2.5ml UDVs (Boehringer Ingelheim Ltd) |
| 5154 | Beta2AdrenoceptorAgonistsSelective | 3408611000001107 | Salbutamol 100micrograms/dose inhaler (A A H Pharmaceuticals Ltd) |
| 5154 | Beta2AdrenoceptorAgonistsSelective | 3410611000001106 | Salbutamol 100micrograms/dose inhaler (Viatris UK Healthcare Ltd) |
| 5154 | Beta2AdrenoceptorAgonistsSelective | 3412611000001107 | Salbutamol 100micrograms/dose inhaler (Kent Pharma (UK) Ltd) |
| 5154 | Beta2AdrenoceptorAgonistsSelective | 3415711000001107 | Salbutamol 100micrograms/dose inhaler (Sandoz Ltd) |
| 5119 | CorticosteroidsInhaled | 134498003 | Budesonide+eformoterol fumarate 200/6mcg breath-actuated dry powder inhaler |
| 5119 | CorticosteroidsInhaled | 134499006 | Budesonide 100micrograms/dose / Formoterol 6micrograms/dose dry powder inhaler |
| 5119 | CorticosteroidsInhaled | 320274007 | Salmeterol+fluticasone propionate 25micrograms/50micrograms CFC-free inhaler |
| 5119 | CorticosteroidsInhaled | 320275008 | Salmeterol+fluticasone propionate 25micrograms/125micrograms CFC-free inhaler |
| 5119 | CorticosteroidsInhaled | 320276009 | Salmeterol+fluticasone propionate 25micrograms/250micrograms CFC-free inhaler |
| 5119 | CorticosteroidsInhaled | 320277000 | Salmeterol+fluticasone propionate 50micrograms/100micrograms breath-actuated dry powder inhaler |
| 5119 | CorticosteroidsInhaled | 320279002 | Salmeterol+fluticasone propionate 50micrograms/250micrograms breath-actuated dry powder inhaler |
| 5119 | CorticosteroidsInhaled | 320280004 | Salmeterol+fluticasone propionate 50micrograms/500micrograms breath-actuated dry powder inhaler |
| 5119 | CorticosteroidsInhaled | 320487003 | Beclometasone 400microgram inhalation powder blisters with device |
| 5119 | CorticosteroidsInhaled | 320488008 | Beclometasone 400microgram inhalation powder blisters |
| 5119 | CorticosteroidsInhaled | 320490009 | Beclometasone 50micrograms/dose breath actuated inhaler |
| 5119 | CorticosteroidsInhaled | 320491008 | Beclometasone 250micrograms/dose breath actuated inhaler |
| 5119 | CorticosteroidsInhaled | 320492001 | Beclometasone 100micrograms/dose breath actuated inhaler |
| 5119 | CorticosteroidsInhaled | 320520000 | Beclometasone 400microgram inhalation powder capsules |
| 5119 | CorticosteroidsInhaled | 320527002 | Beclometasone 100microgram inhalation powder blisters with device |
| 5119 | CorticosteroidsInhaled | 320528007 | Beclometasone 200microgram inhalation powder blisters with device |
| 5119 | CorticosteroidsInhaled | 320529004 | Beclometasone 100microgram inhalation powder blisters |
| 5119 | CorticosteroidsInhaled | 320530009 | Beclometasone 200microgram inhalation powder blisters |
| 5119 | CorticosteroidsInhaled | 320531008 | Beclometasone 250micrograms/dose inhaler |
| 5119 | CorticosteroidsInhaled | 320532001 | Beclometasone 200micrograms/dose inhaler |
| 5119 | CorticosteroidsInhaled | 320533006 | Beclometasone 50micrograms/dose inhaler |
| 5119 | CorticosteroidsInhaled | 320534000 | Beclometasone 100microgram inhalation powder capsules |
| 5119 | CorticosteroidsInhaled | 320535004 | Beclometasone 200microgram inhalation powder capsules |
| 5119 | CorticosteroidsInhaled | 320537007 | Beclometasone dipropionate 100 microgram/actuation pressurised solution for inhalation |
| 5119 | CorticosteroidsInhaled | 320543009 | Salbutamol 400microgram / Beclometasone 200microgram inhalation powder capsules |
| 5119 | CorticosteroidsInhaled | 320544003 | Salbutamol 200microgram / Beclometasone 100microgram inhalation powder capsules |
| 5119 | CorticosteroidsInhaled | 320545002 | Salbutamol 100micrograms/dose / Beclometasone 50micrograms/dose inhaler |
| 5119 | CorticosteroidsInhaled | 320565009 | Budesonide 100micrograms/dose dry powder inhaler |
| 5119 | CorticosteroidsInhaled | 320567001 | Budesonide 200micrograms/dose dry powder inhaler |
| 5119 | CorticosteroidsInhaled | 320568006 | Budesonide 400micrograms/dose dry powder inhaler |
| 5119 | CorticosteroidsInhaled | 320571003 | Budesonide 200micrograms/dose inhaler |
| 5119 | CorticosteroidsInhaled | 320574006 | Budesonide 50micrograms/dose inhaler |
| 5119 | CorticosteroidsInhaled | 320580003 | Fluticasone propionate 50microgram inhalation powder blisters with device |
| 5119 | CorticosteroidsInhaled | 320581004 | Fluticasone propionate 100microgram inhalation powder blisters with device |
| 5119 | CorticosteroidsInhaled | 320582006 | Fluticasone propionate 250microgram inhalation powder blisters with device |
| 5119 | CorticosteroidsInhaled | 320586009 | Fluticasone propionate 50microgram inhalation powder blisters |
| 5119 | CorticosteroidsInhaled | 320587000 | Fluticasone propionate 100microgram inhalation powder blisters |
| 5119 | CorticosteroidsInhaled | 320588005 | Fluticasone propionate 250microgram inhalation powder blisters |
| 5119 | CorticosteroidsInhaled | 320592003 | Fluticasone 25micrograms/dose inhaler |
| 5119 | CorticosteroidsInhaled | 320599007 | Fluticasone propionate 500microgram inhalation powder blisters with device |
| 5119 | CorticosteroidsInhaled | 320600005 | Fluticasone propionate 500microgram inhalation powder blisters |
| 5119 | CorticosteroidsInhaled | 320602002 | Fluticasone propionate 50micrograms breath-actuated dry powder inhaler |
| 5119 | CorticosteroidsInhaled | 320603007 | Fluticasone propionate 100micrograms breath-actuated dry powder inhaler |
| 5119 | CorticosteroidsInhaled | 320604001 | Fluticasone propionate 250micrograms/dose dry powder inhaler |
| 5119 | CorticosteroidsInhaled | 320605000 | Fluticasone propionate 500micrograms breath-actuated dry powder inhaler |
| 5119 | CorticosteroidsInhaled | 320610001 | Fluticasone 500micrograms/2ml nebuliser liquid unit dose vials |
| 5119 | CorticosteroidsInhaled | 320611002 | Fluticasone 2mg/2ml nebuliser liquid unit dose vials |
| 5119 | CorticosteroidsInhaled | 320614005 | Fluticasone propionate 125micrograms CFC-free inhaler |
| 5119 | CorticosteroidsInhaled | 320615006 | Fluticasone propionate 250micrograms CFC-free inhaler |
| 5119 | CorticosteroidsInhaled | 320618008 | Fluticasone propionate 50micrograms CFC-free inhaler |
| 5119 | CorticosteroidsInhaled | 320630002 | Beclometasone 50micrograms/dose dry powder inhaler |
| 5119 | CorticosteroidsInhaled | 320631003 | Beclometasone 100micrograms/dose dry powder inhaler |
| 5119 | CorticosteroidsInhaled | 320632005 | Beclometasone 250micrograms/dose dry powder inhaler |
| 5119 | CorticosteroidsInhaled | 407769008 | Beclometasone 400micrograms/dose dry powder inhaler |
| 5119 | CorticosteroidsInhaled | 407770009 | Beclometasone 200micrograms/dose dry powder inhaler |
| 5119 | CorticosteroidsInhaled | 408013007 | Mometasone furoate 400micrograms breath-actuated dry powder inhaler |
| 5119 | CorticosteroidsInhaled | 408026006 | Mometasone furoate 200micrograms breath-actuated dry powder inhaler |
| 5119 | CorticosteroidsInhaled | 408061000 | Beclometasone 50micrograms/dose inhaler CFC free |
| 5119 | CorticosteroidsInhaled | 408062007 | Beclometasone 50micrograms/dose breath actuated inhaler CFC free |
| 5119 | CorticosteroidsInhaled | 408063002 | Beclometasone 100micrograms/dose inhaler CFC free |
| 5119 | CorticosteroidsInhaled | 408064008 | Beclometasone 100micrograms/dose breath actuated inhaler CFC free |
| 5119 | CorticosteroidsInhaled | 109781000001108 | Beclometasone 400microgram inhalation powder capsules |
| 5119 | CorticosteroidsInhaled | 109941000001100 | Beclometasone 200microgram inhalation powder capsules |
| 5119 | CorticosteroidsInhaled | 111721000001109 | Fluticasone 2mg/2ml nebuliser liquid unit dose vials |
| 5119 | CorticosteroidsInhaled | 112121000001102 | Salbutamol 400microgram / Beclometasone 200microgram inhalation powder capsules |
| 5119 | CorticosteroidsInhaled | 113791000001106 | Fluticasone 500micrograms/2ml nebuliser liquid unit dose vials |
| 5119 | CorticosteroidsInhaled | 113851000001106 | Beclometasone 100microgram inhalation powder capsules |
| 5119 | CorticosteroidsInhaled | 398511000001105 | Flixotide 125micrograms/dose Evohaler (GlaxoSmithKline UK Ltd) |
| 5119 | CorticosteroidsInhaled | 453611000001102 | Seretide 50 Evohaler (GlaxoSmithKline UK Ltd) |
| 5119 | CorticosteroidsInhaled | 539811000001106 | Seretide 250 Evohaler (GlaxoSmithKline UK Ltd) |
| 5119 | CorticosteroidsInhaled | 726611000001102 | Flixotide 50micrograms/dose Evohaler (GlaxoSmithKline UK Ltd) |
| 5119 | CorticosteroidsInhaled | 810211000001105 | Seretide 125 Evohaler (GlaxoSmithKline UK Ltd) |
| 5119 | CorticosteroidsInhaled | 2831211000001109 | Flixotide 250micrograms/dose Evohaler (GlaxoSmithKline UK Ltd) |
| 5119 | CorticosteroidsInhaled | 2924111000001109 | Pulmicort 200micrograms/dose inhaler (AstraZeneca UK Ltd) |
| 5119 | CorticosteroidsInhaled | 3086011000001108 | Becodisks 100microgram (GlaxoSmithKline UK Ltd) |
| 5119 | CorticosteroidsInhaled | 3088611000001100 | Becodisks 100microgram with Diskhaler (GlaxoSmithKline UK Ltd) |
| 5119 | CorticosteroidsInhaled | 3096011000001109 | Becodisks 200microgram with Diskhaler (GlaxoSmithKline UK Ltd) |
| 5119 | CorticosteroidsInhaled | 3097711000001109 | Flixotide 50microgram disks with Diskhaler (GlaxoSmithKline UK Ltd) |
| 5119 | CorticosteroidsInhaled | 3098611000001101 | Flixotide 100microgram disks with Diskhaler (GlaxoSmithKline UK Ltd) |
| 5119 | CorticosteroidsInhaled | 3099611000001105 | Flixotide 100microgram disks (GlaxoSmithKline UK Ltd) |
| 5119 | CorticosteroidsInhaled | 3099811000001109 | Becodisks 200microgram (GlaxoSmithKline UK Ltd) |
| 5119 | CorticosteroidsInhaled | 3100811000001109 | Flixotide 50microgram disks (GlaxoSmithKline UK Ltd) |
| 5119 | CorticosteroidsInhaled | 3102211000001109 | Becodisks 400microgram with Diskhaler (GlaxoSmithKline UK Ltd) |
| 5119 | CorticosteroidsInhaled | 3103211000001103 | Becloforte 400microgram disks with Diskhaler (GlaxoSmithKline UK Ltd) |
| 5119 | CorticosteroidsInhaled | 3103511000001100 | Flixotide 250microgram disks (GlaxoSmithKline UK Ltd) |
| 5119 | CorticosteroidsInhaled | 3104911000001109 | Becodisks 400microgram (GlaxoSmithKline UK Ltd) |
| 5119 | CorticosteroidsInhaled | 3105311000001107 | Becloforte 400microgram disks (GlaxoSmithKline UK Ltd) |
| 5119 | CorticosteroidsInhaled | 3106311000001102 | Flixotide 250microgram disks with Diskhaler (GlaxoSmithKline UK Ltd) |
| 5119 | CorticosteroidsInhaled | 3108411000001105 | Flixotide 500microgram disks with Diskhaler (GlaxoSmithKline UK Ltd) |
| 5119 | CorticosteroidsInhaled | 3110511000001108 | Flixotide 500microgram disks (GlaxoSmithKline UK Ltd) |
| 5119 | CorticosteroidsInhaled | 3111911000001108 | Asmabec 50 Clickhaler (Focus Pharmaceuticals Ltd) |
| 5119 | CorticosteroidsInhaled | 3112411000001105 | Pulmicort 200 Turbohaler (AstraZeneca UK Ltd) |
| 5119 | CorticosteroidsInhaled | 3112511000001109 | Asmabec 100 Clickhaler (Focus Pharmaceuticals Ltd) |
| 5119 | CorticosteroidsInhaled | 3112911000001102 | Pulvinal Beclometasone Dipropionate 100micrograms/dose dry powder inhaler (Chiesi Ltd) |
| 5119 | CorticosteroidsInhaled | 3113111000001106 | Pulmicort 100 Turbohaler (AstraZeneca UK Ltd) |
| 5119 | CorticosteroidsInhaled | 3113411000001101 | Pulvinal Beclometasone Dipropionate 200micrograms/dose dry powder inhaler (Chiesi Ltd) |
| 5119 | CorticosteroidsInhaled | 3174111000001102 | Asmabec 250 Clickhaler (Focus Pharmaceuticals Ltd) |
| 5119 | CorticosteroidsInhaled | 3175111000001103 | Pulvinal Beclometasone Dipropionate 400micrograms/dose dry powder inhaler (Chiesi Ltd) |
| 5119 | CorticosteroidsInhaled | 3175211000001109 | Filair 50 inhaler (Meda Pharmaceuticals Ltd) |
| 5119 | CorticosteroidsInhaled | 3175611000001106 | Qvar 100 inhaler (Teva UK Ltd) |
| 5119 | CorticosteroidsInhaled | 3175711000001102 | Beclometasone 50micrograms/dose inhaler (A A H Pharmaceuticals Ltd) |
| 5119 | CorticosteroidsInhaled | 3175911000001100 | Beclometasone 50micrograms/dose inhaler (Viatris UK Healthcare Ltd) |
| 5119 | CorticosteroidsInhaled | 3176211000001103 | Becotide 50 inhaler (GlaxoSmithKline UK Ltd) |
| 5119 | CorticosteroidsInhaled | 3176411000001104 | Beclazone 50 inhaler (Teva UK Ltd) |
| 5119 | CorticosteroidsInhaled | 3176711000001105 | Beclometasone 50micrograms/dose inhaler (Kent Pharma (UK) Ltd) |
| 5119 | CorticosteroidsInhaled | 3176811000001102 | Qvar 50 inhaler (Teva UK Ltd) |
| 5119 | CorticosteroidsInhaled | 3177111000001107 | Beclometasone 50micrograms/dose inhaler (Alliance Healthcare (Distribution) Ltd) |
| 5119 | CorticosteroidsInhaled | 3177411000001102 | Qvar 50 Autohaler (Teva UK Ltd) |
| 5119 | CorticosteroidsInhaled | 3177711000001108 | Qvar 100 Autohaler (Teva UK Ltd) |
| 5119 | CorticosteroidsInhaled | 3177911000001105 | Filair 100 inhaler (Meda Pharmaceuticals Ltd) |
| 5119 | CorticosteroidsInhaled | 3178211000001102 | Beclometasone 100micrograms/dose inhaler (A A H Pharmaceuticals Ltd) |
| 5119 | CorticosteroidsInhaled | 3178411000001103 | Beclometasone 100micrograms/dose inhaler (Viatris UK Healthcare Ltd) |
| 5119 | CorticosteroidsInhaled | 3178611000001100 | Becotide 100 inhaler (GlaxoSmithKline UK Ltd) |
| 5119 | CorticosteroidsInhaled | 3178811000001101 | Beclazone 100 inhaler (Teva UK Ltd) |
| 5119 | CorticosteroidsInhaled | 3179011000001102 | Beclometasone 100micrograms/dose inhaler (Alliance Healthcare (Distribution) Ltd) |
| 5119 | CorticosteroidsInhaled | 3179311000001104 | Becotide 200 inhaler (GlaxoSmithKline UK Ltd) |
| 5119 | CorticosteroidsInhaled | 3179511000001105 | Beclazone 200 inhaler (Teva UK Ltd) |
| 5119 | CorticosteroidsInhaled | 3179811000001108 | Beclometasone 250micrograms/dose inhaler (A A H Pharmaceuticals Ltd) |
| 5119 | CorticosteroidsInhaled | 3180011000001102 | Beclometasone 250micrograms/dose inhaler (Viatris UK Healthcare Ltd) |
| 5119 | CorticosteroidsInhaled | 3180211000001107 | Becloforte 250micrograms/dose inhaler (GlaxoSmithKline UK Ltd) |
| 5119 | CorticosteroidsInhaled | 3180611000001109 | Beclazone 250 inhaler (Teva UK Ltd) |
| 5119 | CorticosteroidsInhaled | 3180911000001103 | Beclometasone 250micrograms/dose inhaler (Alliance Healthcare (Distribution) Ltd) |
| 5119 | CorticosteroidsInhaled | 3181411000001102 | AeroBec 100 Autohaler (Meda Pharmaceuticals Ltd) |
| 5119 | CorticosteroidsInhaled | 3181711000001108 | Beclazone 100 Easi-Breathe inhaler (Teva UK Ltd) |
| 5119 | CorticosteroidsInhaled | 3182411000001107 | AeroBec Forte 250 Autohaler (Meda Pharmaceuticals Ltd) |
| 5119 | CorticosteroidsInhaled | 3182611000001105 | Beclazone 250 Easi-Breathe inhaler (Teva UK Ltd) |
| 5119 | CorticosteroidsInhaled | 3183811000001101 | Flixotide 50micrograms/dose Accuhaler (GlaxoSmithKline UK Ltd) |
| 5119 | CorticosteroidsInhaled | 3184311000001107 | Flixotide 100micrograms/dose Accuhaler (GlaxoSmithKline UK Ltd) |
| 5119 | CorticosteroidsInhaled | 3184911000001108 | Flixotide 250micrograms/dose Accuhaler (GlaxoSmithKline UK Ltd) |
| 5119 | CorticosteroidsInhaled | 3185211000001103 | Flixotide 500micrograms/dose Accuhaler (GlaxoSmithKline UK Ltd) |
| 5119 | CorticosteroidsInhaled | 3186911000001100 | Seretide 100 Accuhaler (GlaxoSmithKline UK Ltd) |
| 5119 | CorticosteroidsInhaled | 3187211000001106 | Seretide 250 Accuhaler (GlaxoSmithKline UK Ltd) |
| 5119 | CorticosteroidsInhaled | 3188311000001102 | Seretide 500 Accuhaler (GlaxoSmithKline UK Ltd) |
| 5119 | CorticosteroidsInhaled | 3189711000001107 | Beclometasone 100 Cyclocaps (Teva UK Ltd) |
| 5119 | CorticosteroidsInhaled | 3190311000001103 | Becotide 100microgram Rotacaps (GlaxoSmithKline UK Ltd) |
| 5119 | CorticosteroidsInhaled | 3192111000001101 | Becotide 200microgram Rotacaps (GlaxoSmithKline UK Ltd) |
| 5119 | CorticosteroidsInhaled | 3192611000001109 | Beclometasone 200 Cyclocaps (Teva UK Ltd) |
| 5119 | CorticosteroidsInhaled | 3194011000001108 | Becotide 400microgram Rotacaps (GlaxoSmithKline UK Ltd) |
| 5119 | CorticosteroidsInhaled | 3194511000001100 | Beclometasone 400 Cyclocaps (Teva UK Ltd) |
| 5119 | CorticosteroidsInhaled | 3197211000001105 | Budesonide 200 Cyclocaps (Teva UK Ltd) |
| 5119 | CorticosteroidsInhaled | 3198411000001108 | Budesonide 400 Cyclocaps (Teva UK Ltd) |
| 5119 | CorticosteroidsInhaled | 3200511000001109 | Ventide Paediatric Rotacaps (GlaxoSmithKline UK Ltd) |
| 5119 | CorticosteroidsInhaled | 3202211000001107 | Ventide Rotacaps (GlaxoSmithKline UK Ltd) |
| 5119 | CorticosteroidsInhaled | 3228711000001106 | Pulmicort 400 Turbohaler (AstraZeneca UK Ltd) |
| 5119 | CorticosteroidsInhaled | 3240911000001108 | Pulmicort LS 50micrograms/dose inhaler (AstraZeneca UK Ltd) |
| 5119 | CorticosteroidsInhaled | 3292811000001106 | Ventide inhaler (GlaxoSmithKline UK Ltd) |
| 5119 | CorticosteroidsInhaled | 3294211000001101 | Symbicort 100/6 Turbohaler (AstraZeneca UK Ltd) |
| 5119 | CorticosteroidsInhaled | 3294611000001104 | Symbicort 200/6 Turbohaler (AstraZeneca UK Ltd) |
| 5119 | CorticosteroidsInhaled | 3389111000001102 | Flixotide 0.5mg/2ml Nebules (GlaxoSmithKline UK Ltd) |
| 5119 | CorticosteroidsInhaled | 3397211000001103 | Flixotide 2mg/2ml Nebules (GlaxoSmithKline UK Ltd) |
| 5119 | CorticosteroidsInhaled | 3432911000001109 | Budesonide 200microgram inhalation powder capsules |
| 5119 | CorticosteroidsInhaled | 3433011000001101 | Budesonide 400microgram inhalation powder capsules |
| 5119 | CorticosteroidsInhaled | 3604611000001104 | Beclazone 50 Easi-Breathe inhaler (Teva UK Ltd) |
| 5119 | CorticosteroidsInhaled | 3604911000001105 | AeroBec 50 Autohaler (Meda Pharmaceuticals Ltd) |
| 5119 | CorticosteroidsInhaled | 3635411000001106 | Pulmicort 0.5mg Respules (AstraZeneca UK Ltd) |
| 5119 | CorticosteroidsInhaled | 3636511000001103 | Pulmicort 1mg Respules (AstraZeneca UK Ltd) |
| 5119 | CorticosteroidsInhaled | 3654511000001105 | Budesonide 500micrograms/2ml nebuliser liquid unit dose vials |
| 5119 | CorticosteroidsInhaled | 3654611000001109 | Budesonide 1mg/2ml nebuliser liquid unit dose vials |
| 5119 | CorticosteroidsInhaled | 4043811000001103 | Asmanex 400micrograms/dose Twisthaler (Organon Pharma (UK) Ltd) |
| 5119 | CorticosteroidsInhaled | 4045711000001107 | Asmanex 200micrograms/dose Twisthaler (Organon Pharma (UK) Ltd) |
| 5119 | CorticosteroidsInhaled | 4332611000001107 | Beclometasone 200micrograms/dose inhaler (A A H Pharmaceuticals Ltd) |
| 5119 | CorticosteroidsInhaled | 4373811000001100 | Symbicort 400/12 Turbohaler (AstraZeneca UK Ltd) |
| 5119 | CorticosteroidsInhaled | 4378111000001103 | Budesonide 400micrograms/dose / Formoterol 12micrograms/dose dry powder inhaler |
| 5119 | CorticosteroidsInhaled | 4753511000001101 | Filair Forte 250micrograms/dose inhaler (Meda Pharmaceuticals Ltd) |
| 5119 | CorticosteroidsInhaled | 4773611000001100 | Beclometasone 50micrograms/dose inhaler (Teva UK Ltd) |
| 5119 | CorticosteroidsInhaled | 4773811000001101 | Beclometasone 100micrograms/dose inhaler (Teva UK Ltd) |
| 5119 | CorticosteroidsInhaled | 4774011000001109 | Beclometasone 250micrograms/dose inhaler (Teva UK Ltd) |
| 5119 | CorticosteroidsInhaled | 4856011000001103 | Flixotide 25micrograms/dose inhaler (GlaxoSmithKline UK Ltd) |
| 5119 | CorticosteroidsInhaled | 4860811000001104 | Pulmicort 200micrograms/dose inhaler with Nebuchamber (AstraZeneca UK Ltd) |
| 5119 | CorticosteroidsInhaled | 4864911000001104 | Budesonide 200micrograms/dose inhaler with spacer |
| 5119 | CorticosteroidsInhaled | 5256011000001101 | Becloforte 250micrograms/dose inhaler (Waymade Healthcare Plc) |
| 5119 | CorticosteroidsInhaled | 5256411000001105 | Becotide 200microgram Rotacaps (Waymade Healthcare Plc) |
| 5119 | CorticosteroidsInhaled | 5256611000001108 | Becotide 100 inhaler (Waymade Healthcare Plc) |
| 5119 | CorticosteroidsInhaled | 5256811000001107 | Becotide 50 inhaler (Waymade Healthcare Plc) |
| 5119 | CorticosteroidsInhaled | 5258111000001101 | Budesonide 400micrograms/dose Turbohaler (Waymade Healthcare Plc) |
| 5119 | CorticosteroidsInhaled | 5261111000001103 | Flixotide 100micrograms/dose Accuhaler (Waymade Healthcare Plc) |
| 5119 | CorticosteroidsInhaled | 5261411000001108 | Flixotide 250micrograms/dose Accuhaler (Waymade Healthcare Plc) |
| 5119 | CorticosteroidsInhaled | 5262211000001102 | Flixotide 500micrograms/dose Accuhaler (Waymade Healthcare Plc) |
| 5119 | CorticosteroidsInhaled | 5265811000001108 | Flixotide 100microgram disks (Waymade Healthcare Plc) |
| 5119 | CorticosteroidsInhaled | 5266111000001107 | Flixotide 250microgram disks (Waymade Healthcare Plc) |
| 5119 | CorticosteroidsInhaled | 5266711000001108 | Flixotide 500microgram disks (Waymade Healthcare Plc) |
| 5119 | CorticosteroidsInhaled | 5267111000001105 | Flixotide 125micrograms/dose Evohaler (Waymade Healthcare Plc) |
| 5119 | CorticosteroidsInhaled | 5268111000001106 | Flixotide 250micrograms/dose Evohaler (Waymade Healthcare Plc) |
| 5119 | CorticosteroidsInhaled | 5273211000001102 | Pulmicort 100 Turbohaler (Waymade Healthcare Plc) |
| 5119 | CorticosteroidsInhaled | 5273611000001100 | Pulmicort 200 Turbohaler (Waymade Healthcare Plc) |
| 5119 | CorticosteroidsInhaled | 5273911000001106 | Pulmicort 400 Turbohaler (Waymade Healthcare Plc) |
| 5119 | CorticosteroidsInhaled | 5275611000001101 | Seretide 100 Accuhaler (Waymade Healthcare Plc) |
| 5119 | CorticosteroidsInhaled | 5276011000001104 | Seretide 250 Accuhaler (Waymade Healthcare Plc) |
| 5119 | CorticosteroidsInhaled | 5276211000001109 | Seretide 500 Accuhaler (Waymade Healthcare Plc) |
| 5119 | CorticosteroidsInhaled | 5276811000001105 | Seretide 250 Evohaler (Waymade Healthcare Plc) |
| 5119 | CorticosteroidsInhaled | 5277211000001106 | Symbicort 200/6 Turbohaler (Waymade Healthcare Plc) |
| 5119 | CorticosteroidsInhaled | 5278711000001103 | Ventide inhaler (Waymade Healthcare Plc) |
| 5119 | CorticosteroidsInhaled | 5282711000001106 | Becloforte 250micrograms/dose inhaler (Dowelhurst Ltd) |
| 5119 | CorticosteroidsInhaled | 5284411000001107 | Becotide 200microgram Rotacaps (Dowelhurst Ltd) |
| 5119 | CorticosteroidsInhaled | 5284711000001101 | Becotide 50 inhaler (Dowelhurst Ltd) |
| 5119 | CorticosteroidsInhaled | 5290911000001105 | Flixotide 100micrograms/dose Accuhaler (Dowelhurst Ltd) |
| 5119 | CorticosteroidsInhaled | 5292311000001108 | Flixotide 100microgram disks (Dowelhurst Ltd) |
| 5119 | CorticosteroidsInhaled | 5293111000001100 | Flixotide 125micrograms/dose Evohaler (Dowelhurst Ltd) |
| 5119 | CorticosteroidsInhaled | 5293811000001107 | Flixotide 250micrograms/dose Accuhaler (Dowelhurst Ltd) |
| 5119 | CorticosteroidsInhaled | 5294311000001101 | Flixotide 250microgram disks (Dowelhurst Ltd) |
| 5119 | CorticosteroidsInhaled | 5294611000001106 | Flixotide 250micrograms/dose Evohaler (Dowelhurst Ltd) |
| 5119 | CorticosteroidsInhaled | 5294811000001105 | Flixotide 500micrograms/dose Accuhaler (Dowelhurst Ltd) |
| 5119 | CorticosteroidsInhaled | 5295011000001100 | Flixotide 500microgram disks (Dowelhurst Ltd) |
| 5119 | CorticosteroidsInhaled | 5295211000001105 | Flixotide 50micrograms/dose Accuhaler (Dowelhurst Ltd) |
| 5119 | CorticosteroidsInhaled | 5310511000001105 | Pulmicort 0.5mg Respules (Dowelhurst Ltd) |
| 5119 | CorticosteroidsInhaled | 5311011000001106 | Pulmicort 100 Turbohaler (Dowelhurst Ltd) |
| 5119 | CorticosteroidsInhaled | 5312311000001100 | Pulmicort 1mg Respules (Dowelhurst Ltd) |
| 5119 | CorticosteroidsInhaled | 5313011000001107 | Pulmicort 200 Turbohaler (Dowelhurst Ltd) |
| 5119 | CorticosteroidsInhaled | 5313811000001101 | Pulmicort 400 Turbohaler (Dowelhurst Ltd) |
| 5119 | CorticosteroidsInhaled | 5317111000001100 | Seretide 100 Accuhaler (Dowelhurst Ltd) |
| 5119 | CorticosteroidsInhaled | 5317311000001103 | Seretide 250 Accuhaler (Dowelhurst Ltd) |
| 5119 | CorticosteroidsInhaled | 5317511000001109 | Seretide 500 Accuhaler (Dowelhurst Ltd) |
| 5119 | CorticosteroidsInhaled | 5321011000001102 | Symbicort 200/6 Turbohaler (Dowelhurst Ltd) |
| 5119 | CorticosteroidsInhaled | 5324211000001109 | Ventide inhaler (Dowelhurst Ltd) |
| 5119 | CorticosteroidsInhaled | 5350011000001105 | Budesonide 100micrograms/dose Turbohaler (Dowelhurst Ltd) |
| 5119 | CorticosteroidsInhaled | 5350211000001100 | Budesonide 200micrograms/dose Turbohaler (Dowelhurst Ltd) |
| 5119 | CorticosteroidsInhaled | 5350411000001101 | Budesonide 400micrograms/dose Turbohaler (Dowelhurst Ltd) |
| 5119 | CorticosteroidsInhaled | 5350911000001109 | Fluticasone 125micrograms/dose Evohaler (Dowelhurst Ltd) |
| 5119 | CorticosteroidsInhaled | 5351111000001100 | Fluticasone 250micrograms/dose Evohaler (Dowelhurst Ltd) |
| 5119 | CorticosteroidsInhaled | 5404711000001104 | Pulmicort 0.5mg Respules (Waymade Healthcare Plc) |
| 5119 | CorticosteroidsInhaled | 5525411000001107 | Becotide 100microgram Rotacaps (Dowelhurst Ltd) |
| 5119 | CorticosteroidsInhaled | 7379111000001102 | Beclometasone 100micrograms/dose inhaler (Kent Pharma (UK) Ltd) |
| 5119 | CorticosteroidsInhaled | 7379311000001100 | Beclometasone 250micrograms/dose inhaler (Kent Pharma (UK) Ltd) |
| 5119 | CorticosteroidsInhaled | 8024611000001102 | Budesonide 200micrograms/dose dry powder inhalation cartridge with device |
| 5119 | CorticosteroidsInhaled | 8031811000001102 | Budelin Novolizer 200micrograms/dose inhalation powder (Viatris UK Healthcare Ltd) |
| 5119 | CorticosteroidsInhaled | 8159511000001107 | Qvar 50micrograms/dose Easi-Breathe inhaler (Teva UK Ltd) |
| 5119 | CorticosteroidsInhaled | 8159711000001102 | Qvar 100micrograms/dose Easi-Breathe inhaler (Teva UK Ltd) |
| 5119 | CorticosteroidsInhaled | 9003911000001102 | Alvesco 80 inhaler (Covis Pharma Europe B.V.) |
| 5119 | CorticosteroidsInhaled | 9004211000001109 | Alvesco 160 inhaler (Covis Pharma Europe B.V.) |
| 5119 | CorticosteroidsInhaled | 9004411000001108 | Ciclesonide 160micrograms/dose inhaler CFC free |
| 5119 | CorticosteroidsInhaled | 9004511000001107 | Ciclesonide 80micrograms/dose inhaler CFC free |
| 5119 | CorticosteroidsInhaled | 9111811000001100 | Budelin Novolizer 200micrograms/dose inhalation powder refill (Viatris UK Healthcare Ltd) |
| 5119 | CorticosteroidsInhaled | 9117811000001107 | Budesonide 200micrograms/dose dry powder inhalation cartridge |
| 5119 | CorticosteroidsInhaled | 9525111000001105 | Easyhaler Beclometasone 200micrograms/dose dry powder inhaler (Orion Pharma (UK) Ltd) |
| 5119 | CorticosteroidsInhaled | 10073911000001106 | Easyhaler Budesonide 100micrograms/dose dry powder inhaler (Orion Pharma (UK) Ltd) |
| 5119 | CorticosteroidsInhaled | 10074411000001100 | Easyhaler Budesonide 400micrograms/dose dry powder inhaler (Orion Pharma (UK) Ltd) |
| 5119 | CorticosteroidsInhaled | 10074611000001102 | Easyhaler Budesonide 200micrograms/dose dry powder inhaler (Orion Pharma (UK) Ltd) |
| 5119 | CorticosteroidsInhaled | 10272811000001101 | Budesonide 500micrograms/2ml nebuliser liquid unit dose vials (A A H Pharmaceuticals Ltd) |
| 5119 | CorticosteroidsInhaled | 10273211000001108 | Budesonide 1mg/2ml nebuliser liquid unit dose vials (A A H Pharmaceuticals Ltd) |
| 5119 | CorticosteroidsInhaled | 10273611000001105 | Budesonide 500micrograms/2ml nebuliser liquid unit dose vials (Teva UK Ltd) |
| 5119 | CorticosteroidsInhaled | 10274211000001106 | Budesonide 1mg/2ml nebuliser liquid unit dose vials (Teva UK Ltd) |
| 5119 | CorticosteroidsInhaled | 10326511000001109 | Budesonide 500micrograms/2ml nebuliser liquid unit dose vials (Kent Pharma (UK) Ltd) |
| 5119 | CorticosteroidsInhaled | 10326711000001104 | Budesonide 1mg/2ml nebuliser liquid unit dose vials (Kent Pharma (UK) Ltd) |
| 5119 | CorticosteroidsInhaled | 10347711000001104 | Budesonide 500micrograms/2ml nebuliser liquid unit dose vials (Alliance Healthcare (Distribution) Ltd) |
| 5119 | CorticosteroidsInhaled | 10347911000001102 | Budesonide 1mg/2ml nebuliser liquid unit dose vials (Alliance Healthcare (Distribution) Ltd) |
| 5119 | CorticosteroidsInhaled | 10453011000001102 | Budesonide 500micrograms/2ml nebuliser liquid unit dose vials (Arrow Generics Ltd) |
| 5119 | CorticosteroidsInhaled | 10453211000001107 | Budesonide 1mg/2ml nebuliser liquid unit dose vials (Arrow Generics Ltd) |
| 5119 | CorticosteroidsInhaled | 10472811000001102 | Seretide 250 Evohaler (Dowelhurst Ltd) |
| 5119 | CorticosteroidsInhaled | 10473211000001109 | Seretide 125 Evohaler (Dowelhurst Ltd) |
| 5119 | CorticosteroidsInhaled | 10506611000001103 | Symbicort 400/12 Turbohaler (Waymade Healthcare Plc) |
| 5119 | CorticosteroidsInhaled | 10512011000001109 | Seretide 125 Evohaler (Waymade Healthcare Plc) |
| 5119 | CorticosteroidsInhaled | 10527411000001109 | Pulmicort LS 50micrograms/dose inhaler (Waymade Healthcare Plc) |
| 5119 | CorticosteroidsInhaled | 10617711000001103 | Clenil Modulite 50micrograms/dose inhaler (Chiesi Ltd) |
| 5119 | CorticosteroidsInhaled | 10618211000001109 | Clenil Modulite 100micrograms/dose inhaler (Chiesi Ltd) |
| 5119 | CorticosteroidsInhaled | 10619311000001107 | Clenil Modulite 200micrograms/dose inhaler (Chiesi Ltd) |
| 5119 | CorticosteroidsInhaled | 10619611000001102 | Clenil Modulite 250micrograms/dose inhaler (Chiesi Ltd) |
| 5119 | CorticosteroidsInhaled | 10621011000001101 | Beclometasone 200micrograms/dose inhaler CFC free |
| 5119 | CorticosteroidsInhaled | 10621111000001100 | Beclometasone 250micrograms/dose inhaler CFC free |
| 5119 | CorticosteroidsInhaled | 10785011000001101 | Pulmicort 1mg Respules (Waymade Healthcare Plc) |
| 5119 | CorticosteroidsInhaled | 10834011000001106 | Flixotide 2mg/2ml Nebules (Waymade Healthcare Plc) |
| 5119 | CorticosteroidsInhaled | 10835911000001109 | Flixotide 25micrograms/dose inhaler (Waymade Healthcare Plc) |
| 5119 | CorticosteroidsInhaled | 10838111000001101 | Becodisks 200microgram (Waymade Healthcare Plc) |
| 5119 | CorticosteroidsInhaled | 10838311000001104 | Becodisks 400microgram (Waymade Healthcare Plc) |
| 5119 | CorticosteroidsInhaled | 10844511000001104 | Becotide 200 inhaler (Waymade Healthcare Plc) |
| 5119 | CorticosteroidsInhaled | 10855011000001109 | Flixotide 50micrograms/dose Evohaler (Waymade Healthcare Plc) |
| 5119 | CorticosteroidsInhaled | 10983311000001107 | Symbicort 100/6 Turbohaler (Waymade Healthcare Plc) |
| 5119 | CorticosteroidsInhaled | 11005511000001104 | Budesonide 500micrograms/2ml nebuliser liquid unit dose vials (Accord Healthcare Ltd) |
| 5119 | CorticosteroidsInhaled | 11005711000001109 | Budesonide 1mg/2ml nebuliser liquid unit dose vials (Accord Healthcare Ltd) |
| 5119 | CorticosteroidsInhaled | 11400011000001108 | Beclometasone 50micrograms/dose inhaler (Almus Pharmaceuticals Ltd) |
| 5119 | CorticosteroidsInhaled | 11400511000001100 | Beclometasone 100micrograms/dose inhaler (Almus Pharmaceuticals Ltd) |
| 5119 | CorticosteroidsInhaled | 11400811000001102 | Beclometasone 250micrograms/dose inhaler (Almus Pharmaceuticals Ltd) |
| 5119 | CorticosteroidsInhaled | 12888311000001102 | Flixotide 0.5mg/2ml Nebules (Waymade Healthcare Plc) |
| 5119 | CorticosteroidsInhaled | 12906411000001100 | Fostair 100micrograms/dose / 6micrograms/dose inhaler (Chiesi Ltd) |
| 5119 | CorticosteroidsInhaled | 12911011000001100 | Beclometasone 100micrograms/dose / Formoterol 6micrograms/dose inhaler CFC free |
| 5119 | CorticosteroidsInhaled | 13132801000001101 | Budesonide 200micrograms/dose / Formoterol 6micrograms/dose dry powder inhaler |
| 5119 | CorticosteroidsInhaled | 13162101000001100 | Fluticasone 50micrograms/dose / Salmeterol 25micrograms/dose inhaler CFC free |
| 5119 | CorticosteroidsInhaled | 13162201000001107 | Fluticasone 125micrograms/dose / Salmeterol 25micrograms/dose inhaler CFC free |
| 5119 | CorticosteroidsInhaled | 13162301000001103 | Fluticasone 250micrograms/dose / Salmeterol 25micrograms/dose inhaler CFC free |
| 5119 | CorticosteroidsInhaled | 13162401000001106 | Fluticasone propionate 100micrograms/dose / Salmeterol 50micrograms/dose dry powder inhaler |
| 5119 | CorticosteroidsInhaled | 13162501000001105 | Fluticasone propionate 250micrograms/dose / Salmeterol 50micrograms/dose dry powder inhaler |
| 5119 | CorticosteroidsInhaled | 13162601000001109 | Fluticasone propionate 500micrograms/dose / Salmeterol 50micrograms/dose dry powder inhaler |
| 5119 | CorticosteroidsInhaled | 13164801000001101 | Beclometasone 400microgram inhalation powder blisters with device |
| 5119 | CorticosteroidsInhaled | 13164901000001107 | Beclometasone 400microgram inhalation powder blisters |
| 5119 | CorticosteroidsInhaled | 13165001000001107 | Beclometasone 50micrograms/dose breath actuated inhaler |
| 5119 | CorticosteroidsInhaled | 13165101000001108 | Beclometasone 250micrograms/dose breath actuated inhaler |
| 5119 | CorticosteroidsInhaled | 13165201000001101 | Beclometasone 100micrograms/dose breath actuated inhaler |
| 5119 | CorticosteroidsInhaled | 13165301000001105 | Beclometasone 100microgram inhalation powder blisters with device |
| 5119 | CorticosteroidsInhaled | 13165401000001102 | Beclometasone 200microgram inhalation powder blisters with device |
| 5119 | CorticosteroidsInhaled | 13165501000001103 | Beclometasone 100microgram inhalation powder blisters |
| 5119 | CorticosteroidsInhaled | 13165601000001104 | Beclometasone 200microgram inhalation powder blisters |
| 5119 | CorticosteroidsInhaled | 13166001000001102 | Salbutamol 100micrograms/dose / Beclometasone 50micrograms/dose inhaler |
| 5119 | CorticosteroidsInhaled | 13166401000001107 | Budesonide 200micrograms/dose inhaler |
| 5119 | CorticosteroidsInhaled | 13166501000001106 | Budesonide 50micrograms/dose inhaler |
| 5119 | CorticosteroidsInhaled | 13166601000001105 | Fluticasone propionate 50microgram inhalation powder blisters with device |
| 5119 | CorticosteroidsInhaled | 13166701000001100 | Fluticasone propionate 100microgram inhalation powder blisters with device |
| 5119 | CorticosteroidsInhaled | 13166801000001109 | Fluticasone propionate 250microgram inhalation powder blisters with device |
| 5119 | CorticosteroidsInhaled | 13166901000001103 | Fluticasone propionate 50microgram inhalation powder blisters |
| 5119 | CorticosteroidsInhaled | 13167001000001104 | Fluticasone propionate 100microgram inhalation powder blisters |
| 5119 | CorticosteroidsInhaled | 13167101000001103 | Fluticasone propionate 250microgram inhalation powder blisters |
| 5119 | CorticosteroidsInhaled | 13167301000001101 | Fluticasone propionate 500microgram inhalation powder blisters with device |
| 5119 | CorticosteroidsInhaled | 13167401000001109 | Fluticasone propionate 500microgram inhalation powder blisters |
| 5119 | CorticosteroidsInhaled | 13167501000001108 | Fluticasone propionate 50micrograms/dose dry powder inhaler |
| 5119 | CorticosteroidsInhaled | 13167601000001107 | Fluticasone propionate 100micrograms/dose dry powder inhaler |
| 5119 | CorticosteroidsInhaled | 13167801000001106 | Fluticasone propionate 500micrograms/dose dry powder inhaler |
| 5119 | CorticosteroidsInhaled | 13167901000001100 | Fluticasone 125micrograms/dose inhaler CFC free |
| 5119 | CorticosteroidsInhaled | 13168001000001103 | Fluticasone 250micrograms/dose inhaler CFC free |
| 5119 | CorticosteroidsInhaled | 13168101000001102 | Fluticasone 50micrograms/dose inhaler CFC free |
| 5119 | CorticosteroidsInhaled | 13180411000001105 | Beclazone 100 Easi-Breathe inhaler (Dowelhurst Ltd) |
| 5119 | CorticosteroidsInhaled | 13180811000001107 | Beclazone 250 Easi-Breathe inhaler (Dowelhurst Ltd) |
| 5119 | CorticosteroidsInhaled | 13181011000001105 | Beclazone 50 Easi-Breathe inhaler (Dowelhurst Ltd) |
| 5119 | CorticosteroidsInhaled | 13186411000001107 | Budesonide 1mg/2ml nebuliser liquid unit dose vials (Dowelhurst Ltd) |
| 5119 | CorticosteroidsInhaled | 13186811000001109 | Budesonide 500micrograms/2ml nebuliser liquid unit dose vials (Dowelhurst Ltd) |
| 5119 | CorticosteroidsInhaled | 13206411000001106 | Symbicort 100/6 Turbohaler (Dowelhurst Ltd) |
| 5119 | CorticosteroidsInhaled | 13206611000001109 | Symbicort 400/12 Turbohaler (Dowelhurst Ltd) |
| 5119 | CorticosteroidsInhaled | 13261601000001100 | Mometasone 400micrograms/dose dry powder inhaler |
| 5119 | CorticosteroidsInhaled | 13261701000001105 | Mometasone 200micrograms/dose dry powder inhaler |
| 5119 | CorticosteroidsInhaled | 13262201000001105 | Beclometasone 50micrograms/dose breath actuated inhaler CFC free |
| 5119 | CorticosteroidsInhaled | 13262401000001109 | Beclometasone 100micrograms/dose breath actuated inhaler CFC free |
| 5119 | CorticosteroidsInhaled | 13841811000001102 | Flixotide 50micrograms/dose Accuhaler (Waymade Healthcare Plc) |
| 5119 | CorticosteroidsInhaled | 13876511000001105 | Flixotide 100micrograms/dose Accuhaler (DE Pharmaceuticals) |
| 5119 | CorticosteroidsInhaled | 13876711000001100 | Flixotide 250micrograms/dose Accuhaler (DE Pharmaceuticals) |
| 5119 | CorticosteroidsInhaled | 13876911000001103 | Flixotide 500micrograms/dose Accuhaler (DE Pharmaceuticals) |
| 5119 | CorticosteroidsInhaled | 13877111000001103 | Flixotide 250microgram disks (DE Pharmaceuticals) |
| 5119 | CorticosteroidsInhaled | 13877311000001101 | Flixotide 500microgram disks (DE Pharmaceuticals) |
| 5119 | CorticosteroidsInhaled | 13877511000001107 | Flixotide 125micrograms/dose Evohaler (DE Pharmaceuticals) |
| 5119 | CorticosteroidsInhaled | 13877711000001102 | Flixotide 250micrograms/dose Evohaler (DE Pharmaceuticals) |
| 5119 | CorticosteroidsInhaled | 13878311000001100 | Flixotide 0.5mg/2ml Nebules (DE Pharmaceuticals) |
| 5119 | CorticosteroidsInhaled | 13878511000001106 | Flixotide 2mg/2ml Nebules (DE Pharmaceuticals) |
| 5119 | CorticosteroidsInhaled | 13952211000001103 | Pulmicort 0.5mg Respules (DE Pharmaceuticals) |
| 5119 | CorticosteroidsInhaled | 13952511000001100 | Pulmicort 1mg Respules (DE Pharmaceuticals) |
| 5119 | CorticosteroidsInhaled | 13952811000001102 | Pulmicort 100 Turbohaler (DE Pharmaceuticals) |
| 5119 | CorticosteroidsInhaled | 13953111000001103 | Pulmicort 200 Turbohaler (DE Pharmaceuticals) |
| 5119 | CorticosteroidsInhaled | 13953611000001106 | Pulmicort 400 Turbohaler (DE Pharmaceuticals) |
| 5119 | CorticosteroidsInhaled | 13954711000001100 | Qvar 100 inhaler (DE Pharmaceuticals) |
| 5119 | CorticosteroidsInhaled | 13958011000001106 | Symbicort 100/6 Turbohaler (DE Pharmaceuticals) |
| 5119 | CorticosteroidsInhaled | 13958611000001104 | Symbicort 200/6 Turbohaler (DE Pharmaceuticals) |
| 5119 | CorticosteroidsInhaled | 13959211000001106 | Symbicort 400/12 Turbohaler (DE Pharmaceuticals) |
| 5119 | CorticosteroidsInhaled | 13961211000001104 | Pulmicort LS 50micrograms/dose inhaler (DE Pharmaceuticals) |
| 5119 | CorticosteroidsInhaled | 13997111000001100 | Seretide 100 Accuhaler (DE Pharmaceuticals) |
| 5119 | CorticosteroidsInhaled | 13997511000001109 | Seretide 250 Accuhaler (DE Pharmaceuticals) |
| 5119 | CorticosteroidsInhaled | 13998111000001104 | Seretide 125 Evohaler (DE Pharmaceuticals) |
| 5119 | CorticosteroidsInhaled | 13998411000001109 | Seretide 250 Evohaler (DE Pharmaceuticals) |
| 5119 | CorticosteroidsInhaled | 14233711000001100 | Flixotide 100micrograms/dose Accuhaler (Sigma Pharmaceuticals Plc) |
| 5119 | CorticosteroidsInhaled | 14233911000001103 | Flixotide 500micrograms/dose Accuhaler (Sigma Pharmaceuticals Plc) |
| 5119 | CorticosteroidsInhaled | 14234111000001104 | Flixotide 250micrograms/dose Accuhaler (Sigma Pharmaceuticals Plc) |
| 5119 | CorticosteroidsInhaled | 14234311000001102 | Flixotide 50micrograms/dose Accuhaler (Sigma Pharmaceuticals Plc) |
| 5119 | CorticosteroidsInhaled | 14234511000001108 | Flixotide 125micrograms/dose Evohaler (Sigma Pharmaceuticals Plc) |
| 5119 | CorticosteroidsInhaled | 14234711000001103 | Flixotide 250micrograms/dose Evohaler (Sigma Pharmaceuticals Plc) |
| 5119 | CorticosteroidsInhaled | 14234911000001101 | Flixotide 0.5mg/2ml Nebules (Sigma Pharmaceuticals Plc) |
| 5119 | CorticosteroidsInhaled | 14235111000001100 | Flixotide 2mg/2ml Nebules (Sigma Pharmaceuticals Plc) |
| 5119 | CorticosteroidsInhaled | 14235311000001103 | Flixotide 100microgram disks (Sigma Pharmaceuticals Plc) |
| 5119 | CorticosteroidsInhaled | 14235511000001109 | Flixotide 250microgram disks (Sigma Pharmaceuticals Plc) |
| 5119 | CorticosteroidsInhaled | 14235711000001104 | Flixotide 500microgram disks (Sigma Pharmaceuticals Plc) |
| 5119 | CorticosteroidsInhaled | 14238811000001103 | Fluticasone 250micrograms/dose Evohaler (Sigma Pharmaceuticals Plc) |
| 5119 | CorticosteroidsInhaled | 14382711000001109 | Pulmicort 1mg Respules (Sigma Pharmaceuticals Plc) |
| 5119 | CorticosteroidsInhaled | 14386211000001101 | Pulmicort 0.5mg Respules (Sigma Pharmaceuticals Plc) |
| 5119 | CorticosteroidsInhaled | 14387611000001102 | Pulmicort 400 Turbohaler (Sigma Pharmaceuticals Plc) |
| 5119 | CorticosteroidsInhaled | 14390211000001104 | Pulmicort 100 Turbohaler (Sigma Pharmaceuticals Plc) |
| 5119 | CorticosteroidsInhaled | 14390411000001100 | Pulmicort 200 Turbohaler (Sigma Pharmaceuticals Plc) |
| 5119 | CorticosteroidsInhaled | 14616311000001102 | Qvar 100 Autohaler (Sigma Pharmaceuticals Plc) |
| 5119 | CorticosteroidsInhaled | 14616611000001107 | Qvar 100 inhaler (Sigma Pharmaceuticals Plc) |
| 5119 | CorticosteroidsInhaled | 14620511000001104 | Symbicort 100/6 Turbohaler (Sigma Pharmaceuticals Plc) |
| 5119 | CorticosteroidsInhaled | 14620711000001109 | Symbicort 200/6 Turbohaler (Sigma Pharmaceuticals Plc) |
| 5119 | CorticosteroidsInhaled | 14621111000001102 | Symbicort 400/12 Turbohaler (Sigma Pharmaceuticals Plc) |
| 5119 | CorticosteroidsInhaled | 14674111000001100 | Seretide 100 Accuhaler (Sigma Pharmaceuticals Plc) |
| 5119 | CorticosteroidsInhaled | 14674711000001104 | Seretide 250 Accuhaler (Sigma Pharmaceuticals Plc) |
| 5119 | CorticosteroidsInhaled | 14705211000001107 | Seretide 500 Accuhaler (Sigma Pharmaceuticals Plc) |
| 5119 | CorticosteroidsInhaled | 14705411000001106 | Seretide 125 Evohaler (Sigma Pharmaceuticals Plc) |
| 5119 | CorticosteroidsInhaled | 14705811000001108 | Seretide 250 Evohaler (Sigma Pharmaceuticals Plc) |
| 5119 | CorticosteroidsInhaled | 14755711000001106 | Qvar 100 Autohaler (Waymade Healthcare Plc) |
| 5119 | CorticosteroidsInhaled | 14951111000001102 | Pulmicort 100micrograms/dose inhaler CFC free (AstraZeneca UK Ltd) |
| 5119 | CorticosteroidsInhaled | 14959511000001107 | Budesonide 100micrograms/dose inhaler CFC free |
| 5119 | CorticosteroidsInhaled | 15052211000001104 | Beclometasone 200micrograms/dose inhaler (Sigma Pharmaceuticals Plc) |
| 5119 | CorticosteroidsInhaled | 15060511000001109 | Budesonide 1mg/2ml nebuliser liquid unit dose vials (Sigma Pharmaceuticals Plc) |
| 5119 | CorticosteroidsInhaled | 15358411000001102 | Pulmicort 200micrograms/dose inhaler CFC free (AstraZeneca UK Ltd) |
| 5119 | CorticosteroidsInhaled | 15374611000001106 | Budesonide 200micrograms/dose inhaler CFC free |
| 5119 | CorticosteroidsInhaled | 15418911000001106 | Qvar 100micrograms/dose Easi-Breathe inhaler (Waymade Healthcare Plc) |
| 5119 | CorticosteroidsInhaled | 15884311000001105 | Qvar 100 inhaler (Waymade Healthcare Plc) |
| 5119 | CorticosteroidsInhaled | 16140411000001105 | Becodisks 200microgram (Lexon (UK) Ltd) |
| 5119 | CorticosteroidsInhaled | 16140911000001102 | Becodisks 400microgram (Lexon (UK) Ltd) |
| 5119 | CorticosteroidsInhaled | 16179611000001103 | Flixotide 250micrograms/dose Accuhaler (Lexon (UK) Ltd) |
| 5119 | CorticosteroidsInhaled | 16179811000001104 | Flixotide 500micrograms/dose Accuhaler (Lexon (UK) Ltd) |
| 5119 | CorticosteroidsInhaled | 16180011000001105 | Flixotide 125micrograms/dose Evohaler (Lexon (UK) Ltd) |
| 5119 | CorticosteroidsInhaled | 16180211000001100 | Flixotide 250micrograms/dose Evohaler (Lexon (UK) Ltd) |
| 5119 | CorticosteroidsInhaled | 16180411000001101 | Flixotide 0.5mg/2ml Nebules (Lexon (UK) Ltd) |
| 5119 | CorticosteroidsInhaled | 16180611000001103 | Flixotide 2mg/2ml Nebules (Lexon (UK) Ltd) |
| 5119 | CorticosteroidsInhaled | 16230011000001102 | Pulmicort 100 Turbohaler (Lexon (UK) Ltd) |
| 5119 | CorticosteroidsInhaled | 16230211000001107 | Pulmicort 200 Turbohaler (Lexon (UK) Ltd) |
| 5119 | CorticosteroidsInhaled | 16230511000001105 | Pulmicort 400 Turbohaler (Lexon (UK) Ltd) |
| 5119 | CorticosteroidsInhaled | 16230911000001103 | Pulmicort 0.5mg Respules (Lexon (UK) Ltd) |
| 5119 | CorticosteroidsInhaled | 16231411000001102 | Pulmicort 1mg Respules (Lexon (UK) Ltd) |
| 5119 | CorticosteroidsInhaled | 16232011000001103 | Qvar 100 Autohaler (Lexon (UK) Ltd) |
| 5119 | CorticosteroidsInhaled | 16240811000001109 | Seretide 100 Accuhaler (Lexon (UK) Ltd) |
| 5119 | CorticosteroidsInhaled | 16241011000001107 | Seretide 250 Accuhaler (Lexon (UK) Ltd) |
| 5119 | CorticosteroidsInhaled | 16241111000001108 | Seretide 500 Accuhaler (Lexon (UK) Ltd) |
| 5119 | CorticosteroidsInhaled | 16241311000001105 | Seretide 125 Evohaler (Lexon (UK) Ltd) |
| 5119 | CorticosteroidsInhaled | 16241511000001104 | Seretide 250 Evohaler (Lexon (UK) Ltd) |
| 5119 | CorticosteroidsInhaled | 16277711000001101 | Becodisks 200microgram (Mawdsley-Brooks & Company Ltd) |
| 5119 | CorticosteroidsInhaled | 16278211000001107 | Becodisks 400microgram (Mawdsley-Brooks & Company Ltd) |
| 5119 | CorticosteroidsInhaled | 16545111000001101 | Flixotide 250micrograms/dose Accuhaler (Stephar (U.K.) Ltd) |
| 5119 | CorticosteroidsInhaled | 16545311000001104 | Flixotide 125micrograms/dose Evohaler (Stephar (U.K.) Ltd) |
| 5119 | CorticosteroidsInhaled | 16545511000001105 | Flixotide 250micrograms/dose Evohaler (Stephar (U.K.) Ltd) |
| 5119 | CorticosteroidsInhaled | 16579011000001104 | Flixotide 50micrograms/dose Accuhaler (Mawdsley-Brooks & Company Ltd) |
| 5119 | CorticosteroidsInhaled | 16579211000001109 | Flixotide 100micrograms/dose Accuhaler (Mawdsley-Brooks & Company Ltd) |
| 5119 | CorticosteroidsInhaled | 16579411000001108 | Flixotide 250micrograms/dose Accuhaler (Mawdsley-Brooks & Company Ltd) |
| 5119 | CorticosteroidsInhaled | 16579611000001106 | Flixotide 500micrograms/dose Accuhaler (Mawdsley-Brooks & Company Ltd) |
| 5119 | CorticosteroidsInhaled | 16579811000001105 | Flixotide 125micrograms/dose Evohaler (Mawdsley-Brooks & Company Ltd) |
| 5119 | CorticosteroidsInhaled | 16580011000001104 | Flixotide 250micrograms/dose Evohaler (Mawdsley-Brooks & Company Ltd) |
| 5119 | CorticosteroidsInhaled | 16580211000001109 | Flixotide 0.5mg/2ml Nebules (Mawdsley-Brooks & Company Ltd) |
| 5119 | CorticosteroidsInhaled | 16580411000001108 | Flixotide 2mg/2ml Nebules (Mawdsley-Brooks & Company Ltd) |
| 5119 | CorticosteroidsInhaled | 16581211000001103 | Fluticasone 250micrograms/dose Evohaler (Mawdsley-Brooks & Company Ltd) |
| 5119 | CorticosteroidsInhaled | 16586711000001104 | Pulmicort 400 Turbohaler (Stephar (U.K.) Ltd) |
| 5119 | CorticosteroidsInhaled | 16587011000001103 | Pulmicort 0.5mg Respules (Stephar (U.K.) Ltd) |
| 5119 | CorticosteroidsInhaled | 16587211000001108 | Pulmicort 1mg Respules (Stephar (U.K.) Ltd) |
| 5119 | CorticosteroidsInhaled | 16587911000001104 | Seretide 100 Accuhaler (Stephar (U.K.) Ltd) |
| 5119 | CorticosteroidsInhaled | 16588111000001101 | Seretide 250 Evohaler (Stephar (U.K.) Ltd) |
| 5119 | CorticosteroidsInhaled | 16728511000001101 | Seretide 50 Evohaler (Waymade Healthcare Plc) |
| 5119 | CorticosteroidsInhaled | 17425011000001106 | Pulmicort 0.5mg Respules (Mawdsley-Brooks & Company Ltd) |
| 5119 | CorticosteroidsInhaled | 17425311000001109 | Pulmicort 1mg Respules (Mawdsley-Brooks & Company Ltd) |
| 5119 | CorticosteroidsInhaled | 17425611000001104 | Pulmicort 100 Turbohaler (Mawdsley-Brooks & Company Ltd) |
| 5119 | CorticosteroidsInhaled | 17425811000001100 | Pulmicort 200 Turbohaler (Mawdsley-Brooks & Company Ltd) |
| 5119 | CorticosteroidsInhaled | 17426011000001102 | Pulmicort 400 Turbohaler (Mawdsley-Brooks & Company Ltd) |
| 5119 | CorticosteroidsInhaled | 17426811000001108 | Qvar 50 inhaler (Mawdsley-Brooks & Company Ltd) |
| 5119 | CorticosteroidsInhaled | 17427011000001104 | Qvar 100 inhaler (Mawdsley-Brooks & Company Ltd) |
| 5119 | CorticosteroidsInhaled | 17427211000001109 | Qvar 100 Autohaler (Mawdsley-Brooks & Company Ltd) |
| 5119 | CorticosteroidsInhaled | 17440111000001102 | Seretide 100 Accuhaler (Mawdsley-Brooks & Company Ltd) |
| 5119 | CorticosteroidsInhaled | 17440311000001100 | Seretide 250 Accuhaler (Mawdsley-Brooks & Company Ltd) |
| 5119 | CorticosteroidsInhaled | 17440511000001106 | Seretide 500 Accuhaler (Mawdsley-Brooks & Company Ltd) |
| 5119 | CorticosteroidsInhaled | 17440711000001101 | Seretide 125 Evohaler (Mawdsley-Brooks & Company Ltd) |
| 5119 | CorticosteroidsInhaled | 17440911000001104 | Seretide 250 Evohaler (Mawdsley-Brooks & Company Ltd) |
| 5119 | CorticosteroidsInhaled | 17457311000001102 | Symbicort 100/6 Turbohaler (Mawdsley-Brooks & Company Ltd) |
| 5119 | CorticosteroidsInhaled | 17457611000001107 | Symbicort 200/6 Turbohaler (Mawdsley-Brooks & Company Ltd) |
| 5119 | CorticosteroidsInhaled | 17457811000001106 | Symbicort 400/12 Turbohaler (Mawdsley-Brooks & Company Ltd) |
| 5119 | CorticosteroidsInhaled | 17490511000001100 | Alvesco 160 inhaler (Mawdsley-Brooks & Company Ltd) |
| 5119 | CorticosteroidsInhaled | 17505711000001109 | Flixotide 50micrograms/dose Evohaler (Mawdsley-Brooks & Company Ltd) |
| 5119 | CorticosteroidsInhaled | 17600111000001105 | Becodisks 200microgram (Necessity Supplies Ltd) |
| 5119 | CorticosteroidsInhaled | 17600311000001107 | Becodisks 400microgram (Necessity Supplies Ltd) |
| 5119 | CorticosteroidsInhaled | 17793411000001101 | Beclometasone 50micrograms/dose inhaler (Phoenix Healthcare Distribution Ltd) |
| 5119 | CorticosteroidsInhaled | 17794111000001108 | Beclometasone 100micrograms/dose inhaler (Phoenix Healthcare Distribution Ltd) |
| 5119 | CorticosteroidsInhaled | 17809311000001106 | Budesonide 500micrograms/2ml nebuliser liquid unit dose vials (Phoenix Healthcare Distribution Ltd) |
| 5119 | CorticosteroidsInhaled | 17810011000001102 | Budesonide 1mg/2ml nebuliser liquid unit dose vials (Phoenix Healthcare Distribution Ltd) |
| 5119 | CorticosteroidsInhaled | 17833911000001100 | Beclometasone 250micrograms/dose inhaler (Phoenix Healthcare Distribution Ltd) |
| 5119 | CorticosteroidsInhaled | 18050311000001104 | Alvesco 160 inhaler (Lexon (UK) Ltd) |
| 5119 | CorticosteroidsInhaled | 18058211000001105 | Flixotide 50micrograms/dose Evohaler (Lexon (UK) Ltd) |
| 5119 | CorticosteroidsInhaled | 18058811000001106 | Qvar 100micrograms/dose Easi-Breathe inhaler (Sigma Pharmaceuticals Plc) |
| 5119 | CorticosteroidsInhaled | 18059011000001105 | Qvar 50micrograms/dose Easi-Breathe inhaler (Sigma Pharmaceuticals Plc) |
| 5119 | CorticosteroidsInhaled | 18174511000001109 | Pulmicort 1mg Respules (Necessity Supplies Ltd) |
| 5119 | CorticosteroidsInhaled | 18174711000001104 | Pulmicort 0.5mg Respules (Necessity Supplies Ltd) |
| 5119 | CorticosteroidsInhaled | 18174911000001102 | Pulmicort 100 Turbohaler (Necessity Supplies Ltd) |
| 5119 | CorticosteroidsInhaled | 18175111000001101 | Pulmicort 200 Turbohaler (Necessity Supplies Ltd) |
| 5119 | CorticosteroidsInhaled | 18175311000001104 | Pulmicort 400 Turbohaler (Necessity Supplies Ltd) |
| 5119 | CorticosteroidsInhaled | 18185011000001106 | Seretide 100 Accuhaler (Necessity Supplies Ltd) |
| 5119 | CorticosteroidsInhaled | 18185211000001101 | Seretide 250 Accuhaler (Necessity Supplies Ltd) |
| 5119 | CorticosteroidsInhaled | 18185411000001102 | Seretide 500 Accuhaler (Necessity Supplies Ltd) |
| 5119 | CorticosteroidsInhaled | 18185611000001104 | Seretide 125 Evohaler (Necessity Supplies Ltd) |
| 5119 | CorticosteroidsInhaled | 18185811000001100 | Seretide 250 Evohaler (Necessity Supplies Ltd) |
| 5119 | CorticosteroidsInhaled | 18195111000001106 | Symbicort 100/6 Turbohaler (Necessity Supplies Ltd) |
| 5119 | CorticosteroidsInhaled | 18195311000001108 | Symbicort 200/6 Turbohaler (Necessity Supplies Ltd) |
| 5119 | CorticosteroidsInhaled | 18195511000001102 | Symbicort 400/12 Turbohaler (Necessity Supplies Ltd) |
| 5119 | CorticosteroidsInhaled | 18261611000001102 | Flixotide 100micrograms/dose Accuhaler (Necessity Supplies Ltd) |
| 5119 | CorticosteroidsInhaled | 18261811000001103 | Flixotide 250micrograms/dose Accuhaler (Necessity Supplies Ltd) |
| 5119 | CorticosteroidsInhaled | 18262011000001101 | Flixotide 500micrograms/dose Accuhaler (Necessity Supplies Ltd) |
| 5119 | CorticosteroidsInhaled | 18262211000001106 | Flixotide 125micrograms/dose Evohaler (Necessity Supplies Ltd) |
| 5119 | CorticosteroidsInhaled | 18262411000001105 | Flixotide 250micrograms/dose Evohaler (Necessity Supplies Ltd) |
| 5119 | CorticosteroidsInhaled | 18262611000001108 | Flixotide 250microgram disks (Necessity Supplies Ltd) |
| 5119 | CorticosteroidsInhaled | 18263011000001105 | Flixotide 500microgram disks (Necessity Supplies Ltd) |
| 5119 | CorticosteroidsInhaled | 18295511000001100 | Qvar 100 Autohaler (Stephar (U.K.) Ltd) |
| 5119 | CorticosteroidsInhaled | 18507011000001106 | Budesonide 500micrograms/2ml nebuliser liquid unit dose vials (Almus Pharmaceuticals Ltd) |
| 5119 | CorticosteroidsInhaled | 18507211000001101 | Budesonide 1mg/2ml nebuliser liquid unit dose vials (Almus Pharmaceuticals Ltd) |
| 5119 | CorticosteroidsInhaled | 18579211000001101 | Flixotide 50micrograms/dose Evohaler (DE Pharmaceuticals) |
| 5119 | CorticosteroidsInhaled | 18608111000001104 | Qvar 100 Autohaler (DE Pharmaceuticals) |
| 5119 | CorticosteroidsInhaled | 18608311000001102 | Qvar 50 Autohaler (DE Pharmaceuticals) |
| 5119 | CorticosteroidsInhaled | 18608511000001108 | Qvar 100micrograms/dose Easi-Breathe inhaler (DE Pharmaceuticals) |
| 5119 | CorticosteroidsInhaled | 18608711000001103 | Qvar 50micrograms/dose Easi-Breathe inhaler (DE Pharmaceuticals) |
| 5119 | CorticosteroidsInhaled | 18608911000001101 | Qvar 50 inhaler (DE Pharmaceuticals) |
| 5119 | CorticosteroidsInhaled | 18611411000001104 | Seretide 500 Accuhaler (DE Pharmaceuticals) |
| 5119 | CorticosteroidsInhaled | 19612611000001105 | Flixotide 50micrograms/dose Accuhaler (DE Pharmaceuticals) |
| 5119 | CorticosteroidsInhaled | 19726711000001101 | Alvesco 80 inhaler (Mawdsley-Brooks & Company Ltd) |
| 5119 | CorticosteroidsInhaled | 19727911000001101 | Asmanex 400micrograms/dose Twisthaler (Mawdsley-Brooks & Company Ltd) |
| 5119 | CorticosteroidsInhaled | 19737111000001101 | Clenil Modulite 50micrograms/dose inhaler (Mawdsley-Brooks & Company Ltd) |
| 5119 | CorticosteroidsInhaled | 19737311000001104 | Clenil Modulite 100micrograms/dose inhaler (Mawdsley-Brooks & Company Ltd) |
| 5119 | CorticosteroidsInhaled | 19737511000001105 | Clenil Modulite 200micrograms/dose inhaler (Mawdsley-Brooks & Company Ltd) |
| 5119 | CorticosteroidsInhaled | 19737811000001108 | Clenil Modulite 250micrograms/dose inhaler (Mawdsley-Brooks & Company Ltd) |
| 5119 | CorticosteroidsInhaled | 19965011000001108 | Flixotide 100micrograms/dose Accuhaler (Lexon (UK) Ltd) |
| 5119 | CorticosteroidsInhaled | 21019411000001101 | Flutiform 125micrograms/dose / 5micrograms/dose inhaler (Napp Pharmaceuticals Ltd) |
| 5119 | CorticosteroidsInhaled | 21019711000001107 | Flutiform 250micrograms/dose / 10micrograms/dose inhaler (Napp Pharmaceuticals Ltd) |
| 5119 | CorticosteroidsInhaled | 21020611000001104 | Flutiform 50micrograms/dose / 5micrograms/dose inhaler (Napp Pharmaceuticals Ltd) |
| 5119 | CorticosteroidsInhaled | 21113711000001102 | Fluticasone 125micrograms/dose / Formoterol 5micrograms/dose inhaler CFC free |
| 5119 | CorticosteroidsInhaled | 21113811000001105 | Fluticasone 250micrograms/dose / Formoterol 10micrograms/dose inhaler CFC free |
| 5119 | CorticosteroidsInhaled | 21113911000001100 | Fluticasone 50micrograms/dose / Formoterol 5micrograms/dose inhaler CFC free |
| 5119 | CorticosteroidsInhaled | 21690711000001109 | Alvesco 160 inhaler (Waymade Healthcare Plc) |
| 5119 | CorticosteroidsInhaled | 21804411000001104 | Budesonide 500micrograms/2ml nebuliser liquid unit dose vials (Waymade Healthcare Plc) |
| 5119 | CorticosteroidsInhaled | 21804611000001101 | Budesonide 1mg/2ml nebuliser liquid unit dose vials (Waymade Healthcare Plc) |
| 5119 | CorticosteroidsInhaled | 22622411000001105 | Budesonide 500micrograms/2ml nebuliser liquid unit dose vials (DE Pharmaceuticals) |
| 5119 | CorticosteroidsInhaled | 22622611000001108 | Budesonide 1mg/2ml nebuliser liquid unit dose vials (DE Pharmaceuticals) |
| 5119 | CorticosteroidsInhaled | 23438611000001104 | Alvesco 80 inhaler (Waymade Healthcare Plc) |
| 5119 | CorticosteroidsInhaled | 24591711000001104 | Clenil Modulite 200micrograms/dose inhaler (Waymade Healthcare Plc) |
| 5119 | CorticosteroidsInhaled | 24591911000001102 | Clenil Modulite 250micrograms/dose inhaler (Waymade Healthcare Plc) |
| 5119 | CorticosteroidsInhaled | 24660811000001107 | Beclometasone 100micrograms/dose inhaler CFC free (Ennogen Healthcare Ltd) |
| 5119 | CorticosteroidsInhaled | 24667411000001109 | Asmanex 200micrograms/dose Twisthaler (Waymade Healthcare Plc) |
| 5119 | CorticosteroidsInhaled | 25254111000001105 | DuoResp Spiromax 160micrograms/dose / 4.5micrograms/dose dry powder inhaler (Teva UK Ltd) |
| 5119 | CorticosteroidsInhaled | 25254711000001106 | DuoResp Spiromax 320micrograms/dose / 9micrograms/dose dry powder inhaler (Teva UK Ltd) |
| 5119 | CorticosteroidsInhaled | 26112111000001106 | Fostair NEXThaler 100micrograms/dose / 6micrograms/dose dry powder inhaler (Chiesi Ltd) |
| 5119 | CorticosteroidsInhaled | 26148711000001101 | Beclometasone 100micrograms/dose / Formoterol 6micrograms/dose dry powder inhaler |
| 5119 | CorticosteroidsInhaled | 27991211000001105 | Qvar 50micrograms/dose Easi-Breathe inhaler (Waymade Healthcare Plc) |
| 5119 | CorticosteroidsInhaled | 29525711000001109 | Beclometasone 50micrograms/dose inhaler CFC free (Colorama Pharmaceuticals Ltd) |
| 5119 | CorticosteroidsInhaled | 29526111000001102 | Beclometasone 100micrograms/dose inhaler CFC free (Colorama Pharmaceuticals Ltd) |
| 5119 | CorticosteroidsInhaled | 29526411000001107 | Beclometasone 200micrograms/dose inhaler CFC free (Colorama Pharmaceuticals Ltd) |
| 5119 | CorticosteroidsInhaled | 29749211000001101 | Flutiform 125micrograms/dose / 5micrograms/dose inhaler (Waymade Healthcare Plc) |
| 5119 | CorticosteroidsInhaled | 29749411000001102 | Flutiform 50micrograms/dose / 5micrograms/dose inhaler (Waymade Healthcare Plc) |
| 5119 | CorticosteroidsInhaled | 29749611000001104 | Flutiform 250micrograms/dose / 10micrograms/dose inhaler (Waymade Healthcare Plc) |
| 5119 | CorticosteroidsInhaled | 29775411000001101 | Budesonide 500micrograms/2ml nebuliser liquid unit dose vials (Sigma Pharmaceuticals Plc) |
| 5119 | CorticosteroidsInhaled | 29782111000001107 | Sirdupla 25micrograms/dose / 125micrograms/dose inhaler (Viatris UK Healthcare Ltd) |
| 5119 | CorticosteroidsInhaled | 29782511000001103 | Sirdupla 25micrograms/dose / 250micrograms/dose inhaler (Viatris UK Healthcare Ltd) |
| 5119 | CorticosteroidsInhaled | 29959011000001102 | Alvesco 80 inhaler (Lexon (UK) Ltd) |
| 5119 | CorticosteroidsInhaled | 30004311000001102 | Budesonide 500micrograms/2ml nebuliser liquid unit dose vials (Mawdsley-Brooks & Company Ltd) |
| 5119 | CorticosteroidsInhaled | 30004511000001108 | Budesonide 1mg/2ml nebuliser liquid unit dose vials (Mawdsley-Brooks & Company Ltd) |
| 5119 | CorticosteroidsInhaled | 30253911000001100 | Sirdupla 25micrograms/dose / 250micrograms/dose inhaler (Waymade Healthcare Plc) |
| 5119 | CorticosteroidsInhaled | 30950311000001106 | AirFluSal Forspiro 50micrograms/dose / 500micrograms/dose dry powder inhaler (Sandoz Ltd) |
| 5119 | CorticosteroidsInhaled | 30950511000001100 | Beclometasone 100micrograms/dose inhaler CFC free (DE Pharmaceuticals) |
| 5119 | CorticosteroidsInhaled | 30950711000001105 | Beclometasone 200micrograms/dose inhaler CFC free (DE Pharmaceuticals) |
| 5119 | CorticosteroidsInhaled | 30950911000001107 | Beclometasone 50micrograms/dose inhaler CFC free (DE Pharmaceuticals) |
| 5119 | CorticosteroidsInhaled | 31063111000001106 | Fostair NEXThaler 200micrograms/dose / 6micrograms/dose dry powder inhaler (Chiesi Ltd) |
| 5119 | CorticosteroidsInhaled | 31063411000001101 | Fostair 200micrograms/dose / 6micrograms/dose inhaler (Chiesi Ltd) |
| 5119 | CorticosteroidsInhaled | 31064411000001103 | Fluticasone 125micrograms/dose / Salmeterol 25micrograms/dose inhaler CFC free (A A H Pharmaceuticals Ltd) |
| 5119 | CorticosteroidsInhaled | 31064611000001100 | Fluticasone 250micrograms/dose / Salmeterol 25micrograms/dose inhaler CFC free (A A H Pharmaceuticals Ltd) |
| 5119 | CorticosteroidsInhaled | 31064811000001101 | Fluticasone propionate 500micrograms/dose / Salmeterol 50micrograms/dose dry powder inhaler (A A H Pharmaceuticals Ltd) |
| 5119 | CorticosteroidsInhaled | 31087411000001106 | Beclometasone 200micrograms/dose / Formoterol 6micrograms/dose inhaler CFC free |
| 5119 | CorticosteroidsInhaled | 31087511000001105 | Beclometasone 200micrograms/dose / Formoterol 6micrograms/dose dry powder inhaler |
| 5119 | CorticosteroidsInhaled | 31352911000001106 | Beclometasone 100micrograms/dose inhaler CFC free (J M McGill Ltd) |
| 5119 | CorticosteroidsInhaled | 31353111000001102 | Beclometasone 200micrograms/dose inhaler CFC free (J M McGill Ltd) |
| 5119 | CorticosteroidsInhaled | 31353311000001100 | Beclometasone 50micrograms/dose inhaler CFC free (J M McGill Ltd) |
| 5119 | CorticosteroidsInhaled | 32199311000001102 | Beclometasone 100micrograms/dose inhaler CFC free (Niche Pharma Ltd) |
| 5119 | CorticosteroidsInhaled | 32199911000001101 | Beclometasone 200micrograms/dose inhaler CFC free (Niche Pharma Ltd) |
| 5119 | CorticosteroidsInhaled | 32200311000001106 | Beclometasone 50micrograms/dose inhaler CFC free (Niche Pharma Ltd) |
| 5119 | CorticosteroidsInhaled | 32926011000001100 | Symbicort 200micrograms/dose / 6micrograms/dose pressurised inhaler (AstraZeneca UK Ltd) |
| 5119 | CorticosteroidsInhaled | 32960711000001105 | Budesonide 200micrograms/dose / Formoterol 6micrograms/dose inhaler CFC free |
| 5119 | CorticosteroidsInhaled | 33679711000001103 | Aerivio Spiromax 50micrograms/dose / 500micrograms/dose dry powder inhaler (Teva UK Ltd) |
| 5119 | CorticosteroidsInhaled | 34023611000001101 | Sereflo 25micrograms/dose / 125micrograms/dose inhaler (Cipla EU Ltd) |
| 5119 | CorticosteroidsInhaled | 34023811000001102 | Sereflo 25micrograms/dose / 250micrograms/dose inhaler (Cipla EU Ltd) |
| 5119 | CorticosteroidsInhaled | 34215311000001107 | AirFluSal 25micrograms/dose / 125micrograms/dose inhaler (Sandoz Ltd) |
| 5119 | CorticosteroidsInhaled | 34215511000001101 | AirFluSal 25micrograms/dose / 250micrograms/dose inhaler (Sandoz Ltd) |
| 5119 | CorticosteroidsInhaled | 34675711000001103 | Aloflute 25micrograms/dose / 250micrograms/dose inhaler (Viatris UK Healthcare Ltd) |
| 5119 | CorticosteroidsInhaled | 34677011000001107 | Aloflute 25micrograms/dose / 125micrograms/dose inhaler (Viatris UK Healthcare Ltd) |
| 5119 | CorticosteroidsInhaled | 34681611000001100 | Trimbow 87micrograms/dose / 5micrograms/dose / 9micrograms/dose inhaler (Chiesi Ltd) |
| 5119 | CorticosteroidsInhaled | 34683311000001106 | Generic Trimbow 87micrograms/dose / 5micrograms/dose / 9micrograms/dose inhaler |
| 5119 | CorticosteroidsInhaled | 34812111000001106 | Fobumix Easyhaler 320micrograms/dose / 9micrograms/dose dry powder inhaler (Orion Pharma (UK) Ltd) |
| 5119 | CorticosteroidsInhaled | 34950311000001108 | Fobumix Easyhaler 160micrograms/dose / 4.5micrograms/dose dry powder inhaler (Orion Pharma (UK) Ltd) |
| 5119 | CorticosteroidsInhaled | 34950611000001103 | Fobumix Easyhaler 80micrograms/dose / 4.5micrograms/dose dry powder inhaler (Orion Pharma (UK) Ltd) |
| 5119 | CorticosteroidsInhaled | 35369511000001103 | Salbutamol 200microgram / Beclometasone 100microgram inhalation powder capsules |
| 5119 | CorticosteroidsInhaled | 35430111000001100 | Kelhale 50micrograms/dose inhaler (Cipla EU Ltd) |
| 5119 | CorticosteroidsInhaled | 35430311000001103 | Kelhale 100micrograms/dose inhaler (Cipla EU Ltd) |
| 5119 | CorticosteroidsInhaled | 35515311000001106 | Fusacomb Easyhaler 50micrograms/dose / 500micrograms/dose dry powder inhaler (Orion Pharma (UK) Ltd) |
| 5119 | CorticosteroidsInhaled | 35515511000001100 | Fusacomb Easyhaler 50micrograms/dose / 250micrograms/dose dry powder inhaler (Orion Pharma (UK) Ltd) |
| 5119 | CorticosteroidsInhaled | 35594011000001105 | Combisal 25micrograms/dose / 50micrograms/dose inhaler (Aspire Pharma Ltd) |
| 5119 | CorticosteroidsInhaled | 35594211000001100 | Combisal 25micrograms/dose / 125micrograms/dose inhaler (Aspire Pharma Ltd) |
| 5119 | CorticosteroidsInhaled | 35594411000001101 | Combisal 25micrograms/dose / 250micrograms/dose inhaler (Aspire Pharma Ltd) |
| 5119 | CorticosteroidsInhaled | 35647311000001101 | Flutiform K-haler 125micrograms/dose / 5micrograms/dose breath actuated inhaler (Napp Pharmaceuticals Ltd) |
| 5119 | CorticosteroidsInhaled | 35647511000001107 | Fluticasone 125micrograms/dose / Formoterol 5micrograms/dose breath actuated inhaler CFC free |
| 5119 | CorticosteroidsInhaled | 35647611000001106 | Fluticasone 50micrograms/dose / Formoterol 5micrograms/dose breath actuated inhaler CFC free |
| 5119 | CorticosteroidsInhaled | 35650811000001109 | Flutiform K-haler 50micrograms/dose / 5micrograms/dose breath actuated inhaler (Napp Pharmaceuticals Ltd) |
| 5119 | CorticosteroidsInhaled | 35907611000001101 | Beclometasone 100microgram inhalation powder blisters |
| 5119 | CorticosteroidsInhaled | 35907711000001105 | Beclometasone 100microgram inhalation powder blisters with device |
| 5119 | CorticosteroidsInhaled | 35907811000001102 | Beclometasone 100micrograms/dose breath actuated inhaler |
| 5119 | CorticosteroidsInhaled | 35907911000001107 | Beclometasone 100micrograms/dose breath actuated inhaler CFC free |
| 5119 | CorticosteroidsInhaled | 35908011000001109 | Beclometasone 100micrograms/dose dry powder inhaler |
| 5119 | CorticosteroidsInhaled | 35908111000001105 | Beclometasone 100micrograms/dose inhaler CFC free |
| 5119 | CorticosteroidsInhaled | 35908211000001104 | Beclometasone 200microgram inhalation powder blisters |
| 5119 | CorticosteroidsInhaled | 35908311000001107 | Beclometasone 200microgram inhalation powder blisters with device |
| 5119 | CorticosteroidsInhaled | 35908411000001100 | Beclometasone 200micrograms/dose dry powder inhaler |
| 5119 | CorticosteroidsInhaled | 35908511000001101 | Beclometasone 200micrograms/dose inhaler |
| 5119 | CorticosteroidsInhaled | 35908611000001102 | Beclometasone 250micrograms/dose breath actuated inhaler |
| 5119 | CorticosteroidsInhaled | 35908711000001106 | Beclometasone 250micrograms/dose dry powder inhaler |
| 5119 | CorticosteroidsInhaled | 35908811000001103 | Beclometasone 250micrograms/dose inhaler |
| 5119 | CorticosteroidsInhaled | 35908911000001108 | Beclometasone 400microgram inhalation powder blisters |
| 5119 | CorticosteroidsInhaled | 35909011000001104 | Beclometasone 400microgram inhalation powder blisters with device |
| 5119 | CorticosteroidsInhaled | 35909111000001103 | Beclometasone 400micrograms/dose dry powder inhaler |
| 5119 | CorticosteroidsInhaled | 35909211000001109 | Beclometasone 50micrograms/dose breath actuated inhaler |
| 5119 | CorticosteroidsInhaled | 35909311000001101 | Beclometasone 50micrograms/dose breath actuated inhaler CFC free |
| 5119 | CorticosteroidsInhaled | 35909411000001108 | Beclometasone 50micrograms/dose dry powder inhaler |
| 5119 | CorticosteroidsInhaled | 35909511000001107 | Beclometasone 50micrograms/dose inhaler |
| 5119 | CorticosteroidsInhaled | 35909611000001106 | Beclometasone 50micrograms/dose inhaler CFC free |
| 5119 | CorticosteroidsInhaled | 35912011000001109 | Budesonide 100micrograms/dose / Formoterol 6micrograms/dose dry powder inhaler |
| 5119 | CorticosteroidsInhaled | 35912111000001105 | Budesonide 100micrograms/dose dry powder inhaler |
| 5119 | CorticosteroidsInhaled | 35912411000001100 | Budesonide 200micrograms/dose dry powder inhaler |
| 5119 | CorticosteroidsInhaled | 35912511000001101 | Budesonide 200micrograms/dose inhaler |
| 5119 | CorticosteroidsInhaled | 35912711000001106 | Budesonide 400micrograms/dose dry powder inhaler |
| 5119 | CorticosteroidsInhaled | 35912811000001103 | Budesonide 50micrograms/dose inhaler |
| 5119 | CorticosteroidsInhaled | 35936211000001105 | Salbutamol 100micrograms/dose / Beclometasone 50micrograms/dose inhaler |
| 5119 | CorticosteroidsInhaled | 36059111000001102 | Fluticasone 25micrograms/dose inhaler |
| 5119 | CorticosteroidsInhaled | 36059211000001108 | Fluticasone propionate 100microgram inhalation powder blisters |
| 5119 | CorticosteroidsInhaled | 36059311000001100 | Fluticasone propionate 100microgram inhalation powder blisters with device |
| 5119 | CorticosteroidsInhaled | 36059411000001107 | Fluticasone propionate 250microgram inhalation powder blisters with device |
| 5119 | CorticosteroidsInhaled | 36059511000001106 | Fluticasone propionate 250microgram inhalation powder blisters |
| 5119 | CorticosteroidsInhaled | 36059611000001105 | Fluticasone propionate 500microgram inhalation powder blisters |
| 5119 | CorticosteroidsInhaled | 36059711000001101 | Fluticasone propionate 500microgram inhalation powder blisters with device |
| 5119 | CorticosteroidsInhaled | 36059811000001109 | Fluticasone propionate 50microgram inhalation powder blisters |
| 5119 | CorticosteroidsInhaled | 36059911000001104 | Fluticasone propionate 50microgram inhalation powder blisters with device |
| 5119 | CorticosteroidsInhaled | 36565411000001101 | Fluticasone propionate 250micrograms/dose dry powder inhaler |
| 5119 | CorticosteroidsInhaled | 36603211000001106 | Soprobec 50micrograms/dose inhaler (Glenmark Pharmaceuticals Europe Ltd) |
| 5119 | CorticosteroidsInhaled | 36603411000001105 | Soprobec 200micrograms/dose inhaler (Glenmark Pharmaceuticals Europe Ltd) |
| 5119 | CorticosteroidsInhaled | 36603611000001108 | Soprobec 100micrograms/dose inhaler (Glenmark Pharmaceuticals Europe Ltd) |
| 5119 | CorticosteroidsInhaled | 36603811000001107 | Soprobec 250micrograms/dose inhaler (Glenmark Pharmaceuticals Europe Ltd) |
| 5119 | CorticosteroidsInhaled | 36604711000001102 | Stalpex 50micrograms/dose / 500micrograms/dose dry powder inhaler (Glenmark Pharmaceuticals Europe Ltd) |
| 5119 | CorticosteroidsInhaled | 36889311000001107 | DuoResp Spiromax 160micrograms/dose / 4.5micrograms/dose dry powder inhaler (Pharmaram Ltd) |
| 5119 | CorticosteroidsInhaled | 36889511000001101 | DuoResp Spiromax 320micrograms/dose / 9micrograms/dose dry powder inhaler (Pharmaram Ltd) |
| 5119 | CorticosteroidsInhaled | 37391711000001105 | Flutiform 125micrograms/dose / 5micrograms/dose inhaler (CST Pharma Ltd) |
| 5119 | CorticosteroidsInhaled | 37397211000001108 | DuoResp Spiromax 320micrograms/dose / 9micrograms/dose dry powder inhaler (Mawdsley-Brooks & Company Ltd) |
| 5119 | CorticosteroidsInhaled | 37397411000001107 | DuoResp Spiromax 160micrograms/dose / 4.5micrograms/dose dry powder inhaler (Mawdsley-Brooks & Company Ltd) |
| 5119 | CorticosteroidsInhaled | 37400911000001108 | Qvar 100 Autohaler (CST Pharma Ltd) |
| 5119 | CorticosteroidsInhaled | 37434411000001103 | DuoResp Spiromax 160micrograms/dose / 4.5micrograms/dose dry powder inhaler (CST Pharma Ltd) |
| 5119 | CorticosteroidsInhaled | 37434611000001100 | DuoResp Spiromax 320micrograms/dose / 9micrograms/dose dry powder inhaler (CST Pharma Ltd) |
| 5119 | CorticosteroidsInhaled | 37443211000001101 | Seretide 250 Accuhaler (CST Pharma Ltd) |
| 5119 | CorticosteroidsInhaled | 37443411000001102 | Seretide 500 Accuhaler (CST Pharma Ltd) |
| 5119 | CorticosteroidsInhaled | 37443611000001104 | Seretide 250 Evohaler (CST Pharma Ltd) |
| 5119 | CorticosteroidsInhaled | 37444011000001108 | Seretide 125 Evohaler (CST Pharma Ltd) |
| 5119 | CorticosteroidsInhaled | 37444211000001103 | Symbicort 400/12 Turbohaler (CST Pharma Ltd) |
| 5119 | CorticosteroidsInhaled | 37444611000001101 | Qvar 100 inhaler (CST Pharma Ltd) |
| 5119 | CorticosteroidsInhaled | 37446211000001108 | Flixotide 100micrograms/dose Accuhaler (CST Pharma Ltd) |
| 5119 | CorticosteroidsInhaled | 37446411000001107 | Flixotide 250micrograms/dose Accuhaler (CST Pharma Ltd) |
| 5119 | CorticosteroidsInhaled | 37446611000001105 | Flixotide 500micrograms/dose Accuhaler (CST Pharma Ltd) |
| 5119 | CorticosteroidsInhaled | 37448711000001102 | Flutiform 250micrograms/dose / 10micrograms/dose inhaler (CST Pharma Ltd) |
| 5119 | CorticosteroidsInhaled | 37484711000001101 | Flutiform 250micrograms/dose / 10micrograms/dose inhaler (Mawdsley-Brooks & Company Ltd) |
| 5119 | CorticosteroidsInhaled | 37484911000001104 | Flutiform 125micrograms/dose / 5micrograms/dose inhaler (Mawdsley-Brooks & Company Ltd) |
| 5119 | CorticosteroidsInhaled | 37577111000001108 | Alvesco 160 inhaler (CST Pharma Ltd) |
| 5119 | CorticosteroidsInhaled | 37631511000001104 | DuoResp Spiromax 160micrograms/dose / 4.5micrograms/dose dry powder inhaler (Ethigen Ltd) |
| 5119 | CorticosteroidsInhaled | 37631711000001109 | DuoResp Spiromax 320micrograms/dose / 9micrograms/dose dry powder inhaler (Ethigen Ltd) |
| 5119 | CorticosteroidsInhaled | 37665111000001104 | Flutiform 125micrograms/dose / 5micrograms/dose inhaler (Pilsco Ltd) |
| 5119 | CorticosteroidsInhaled | 37665311000001102 | Flutiform 250micrograms/dose / 10micrograms/dose inhaler (Pilsco Ltd) |
| 5119 | CorticosteroidsInhaled | 37699111000001100 | Pulmicort 0.5mg Respules (Pilsco Ltd) |
| 5119 | CorticosteroidsInhaled | 37699311000001103 | Pulmicort 1mg Respules (Pilsco Ltd) |
| 5119 | CorticosteroidsInhaled | 37699711000001104 | Qvar 100 Autohaler (Pilsco Ltd) |
| 5119 | CorticosteroidsInhaled | 37702211000001106 | Seretide 250 Accuhaler (Pilsco Ltd) |
| 5119 | CorticosteroidsInhaled | 37702411000001105 | Seretide 250 Evohaler (Pilsco Ltd) |
| 5119 | CorticosteroidsInhaled | 37702611000001108 | Seretide 500 Accuhaler (Pilsco Ltd) |
| 5119 | CorticosteroidsInhaled | 37714411000001100 | AirFluSal Forspiro 50micrograms/dose / 500micrograms/dose dry powder inhaler (Pilsco Ltd) |
| 5119 | CorticosteroidsInhaled | 37714611000001102 | AirFluSal 25micrograms/dose / 125micrograms/dose inhaler (Pilsco Ltd) |
| 5119 | CorticosteroidsInhaled | 37714811000001103 | AirFluSal 25micrograms/dose / 250micrograms/dose inhaler (Pilsco Ltd) |
| 5119 | CorticosteroidsInhaled | 37727811000001101 | Qvar 100 inhaler (Pilsco Ltd) |
| 5119 | CorticosteroidsInhaled | 37729411000001107 | Sereflo 25micrograms/dose / 250micrograms/dose inhaler (Pilsco Ltd) |
| 5119 | CorticosteroidsInhaled | 37739011000001101 | Symbicort 200/6 Turbohaler (CST Pharma Ltd) |
| 5119 | CorticosteroidsInhaled | 37996511000001103 | Seretide 125 Evohaler (Pharmaram Ltd) |
| 5119 | CorticosteroidsInhaled | 37996711000001108 | Seretide 250 Evohaler (Pharmaram Ltd) |
| 5119 | CorticosteroidsInhaled | 37997311000001107 | Symbicort 100/6 Turbohaler (Pharmaram Ltd) |
| 5119 | CorticosteroidsInhaled | 37997511000001101 | Symbicort 200/6 Turbohaler (Pharmaram Ltd) |
| 5119 | CorticosteroidsInhaled | 37997711000001106 | Symbicort 400/12 Turbohaler (Pharmaram Ltd) |
| 5119 | CorticosteroidsInhaled | 38130011000001108 | DuoResp Spiromax 160micrograms/dose / 4.5micrograms/dose dry powder inhaler (DE Pharmaceuticals) |
| 5119 | CorticosteroidsInhaled | 38130211000001103 | DuoResp Spiromax 320micrograms/dose / 9micrograms/dose dry powder inhaler (DE Pharmaceuticals) |
| 5119 | CorticosteroidsInhaled | 38130411000001104 | Easyhaler Beclometasone 200micrograms/dose dry powder inhaler (DE Pharmaceuticals) |
| 5119 | CorticosteroidsInhaled | 38130611000001101 | Easyhaler Budesonide 100micrograms/dose dry powder inhaler (DE Pharmaceuticals) |
| 5119 | CorticosteroidsInhaled | 38130811000001102 | Easyhaler Budesonide 200micrograms/dose dry powder inhaler (DE Pharmaceuticals) |
| 5119 | CorticosteroidsInhaled | 38131011000001104 | Easyhaler Budesonide 400micrograms/dose dry powder inhaler (DE Pharmaceuticals) |
| 5119 | CorticosteroidsInhaled | 38134511000001109 | Flutiform 125micrograms/dose / 5micrograms/dose inhaler (DE Pharmaceuticals) |
| 5119 | CorticosteroidsInhaled | 38134711000001104 | Flutiform 250micrograms/dose / 10micrograms/dose inhaler (DE Pharmaceuticals) |
| 5119 | CorticosteroidsInhaled | 38134911000001102 | Flutiform 50micrograms/dose / 5micrograms/dose inhaler (DE Pharmaceuticals) |
| 5119 | CorticosteroidsInhaled | 38135111000001101 | Flutiform K-haler 125micrograms/dose / 5micrograms/dose breath actuated inhaler (DE Pharmaceuticals) |
| 5119 | CorticosteroidsInhaled | 38135311000001104 | Flutiform K-haler 50micrograms/dose / 5micrograms/dose breath actuated inhaler (DE Pharmaceuticals) |
| 5119 | CorticosteroidsInhaled | 38136011000001106 | Fostair NEXThaler 100micrograms/dose / 6micrograms/dose dry powder inhaler (DE Pharmaceuticals) |
| 5119 | CorticosteroidsInhaled | 38166911000001103 | Sirdupla 25micrograms/dose / 125micrograms/dose inhaler (DE Pharmaceuticals) |
| 5119 | CorticosteroidsInhaled | 38167111000001103 | Sirdupla 25micrograms/dose / 250micrograms/dose inhaler (DE Pharmaceuticals) |
| 5119 | CorticosteroidsInhaled | 38640111000001109 | Sirdupla 25micrograms/dose / 125micrograms/dose inhaler (Pilsco Ltd) |
| 5119 | CorticosteroidsInhaled | 38640311000001106 | Sirdupla 25micrograms/dose / 250micrograms/dose inhaler (Pilsco Ltd) |
| 5119 | CorticosteroidsInhaled | 38807211000001100 | Budesonide 500micrograms/2ml nebuliser liquid unit dose vials (Medihealth (Northern) Ltd) |
| 5119 | CorticosteroidsInhaled | 38807411000001101 | Budesonide 1mg/2ml nebuliser liquid unit dose vials (Medihealth (Northern) Ltd) |
| 5119 | CorticosteroidsInhaled | 38895411000001109 | Mometasone 200micrograms/dose dry powder inhaler |
| 5119 | CorticosteroidsInhaled | 38895611000001107 | Mometasone 400micrograms/dose dry powder inhaler |
| 5119 | CorticosteroidsInhaled | 38896811000001103 | Budesonide 200micrograms/dose / Formoterol 6micrograms/dose dry powder inhaler |
| 5119 | CorticosteroidsInhaled | 38897411000001103 | Fluticasone propionate 500micrograms/dose / Salmeterol 50micrograms/dose dry powder inhaler |
| 5119 | CorticosteroidsInhaled | 38897511000001104 | Fluticasone propionate 250micrograms/dose / Salmeterol 50micrograms/dose dry powder inhaler |
| 5119 | CorticosteroidsInhaled | 38897611000001100 | Fluticasone propionate 100micrograms/dose / Salmeterol 50micrograms/dose dry powder inhaler |
| 5119 | CorticosteroidsInhaled | 38897711000001109 | Fluticasone propionate 500micrograms/dose dry powder inhaler |
| 5119 | CorticosteroidsInhaled | 38897811000001101 | Fluticasone propionate 100micrograms/dose dry powder inhaler |
| 5119 | CorticosteroidsInhaled | 38897911000001106 | Fluticasone propionate 50micrograms/dose dry powder inhaler |
| 5119 | CorticosteroidsInhaled | 38960111000001101 | Fluticasone 500micrograms/2ml nebuliser liquid unit dose vials (Imported) |
| 5119 | CorticosteroidsInhaled | 38960511000001105 | Fluticasone 2mg/2ml nebuliser liquid unit dose vials (Imported) |
| 5119 | CorticosteroidsInhaled | 39089311000001103 | Flutiform K-haler 125micrograms/dose / 5micrograms/dose breath actuated inhaler (Pilsco Ltd) |
| 5119 | CorticosteroidsInhaled | 39105811000001102 | Symbicort 100micrograms/dose / 3micrograms/dose pressurised inhaler (AstraZeneca UK Ltd) |
| 5119 | CorticosteroidsInhaled | 39110911000001100 | Fluticasone 50micrograms/dose inhaler CFC free |
| 5119 | CorticosteroidsInhaled | 39111011000001108 | Fluticasone 50micrograms/dose / Salmeterol 25micrograms/dose inhaler CFC free |
| 5119 | CorticosteroidsInhaled | 39111111000001109 | Fluticasone 125micrograms/dose / Salmeterol 25micrograms/dose inhaler CFC free |
| 5119 | CorticosteroidsInhaled | 39111211000001103 | Fluticasone 125micrograms/dose inhaler CFC free |
| 5119 | CorticosteroidsInhaled | 39111311000001106 | Fluticasone 250micrograms/dose / Salmeterol 25micrograms/dose inhaler CFC free |
| 5119 | CorticosteroidsInhaled | 39111411000001104 | Fluticasone 250micrograms/dose inhaler CFC free |
| 5119 | CorticosteroidsInhaled | 39114511000001103 | Atectura Breezhaler 125micrograms/62.5micrograms inhalation powder capsules with device (Sandoz Ltd) |
| 5119 | CorticosteroidsInhaled | 39115411000001101 | Atectura Breezhaler 125micrograms/127.5micrograms inhalation powder capsules with device (Sandoz Ltd) |
| 5119 | CorticosteroidsInhaled | 39115911000001109 | Atectura Breezhaler 125micrograms/260micrograms inhalation powder capsules with device (Sandoz Ltd) |
| 5119 | CorticosteroidsInhaled | 39116311000001103 | Indacaterol 125micrograms/dose / Mometasone 127.5micrograms/dose inhalation powder capsules with device |
| 5119 | CorticosteroidsInhaled | 39116411000001105 | Indacaterol 125micrograms/dose / Mometasone 260micrograms/dose inhalation powder capsules with device |
| 5119 | CorticosteroidsInhaled | 39116511000001109 | Indacaterol 125micrograms/dose / Mometasone 62.5micrograms/dose inhalation powder capsules with device |
| 5119 | CorticosteroidsInhaled | 39133611000001108 | Budesonide 100micrograms/dose / Formoterol 3micrograms/dose inhaler CFC free |
| 5119 | CorticosteroidsInhaled | 39134511000001107 | Generic Enerzair Breezhaler 114micrograms/dose / 46micrograms/dose / 136micrograms/dose inhalation powder capsules with device |
| 5119 | CorticosteroidsInhaled | 39134711000001102 | Enerzair Breezhaler 114micrograms/dose / 46micrograms/dose / 136micrograms/dose inhalation powder capsules with device (Sandoz Ltd) |
| 5119 | CorticosteroidsInhaled | 39327311000001104 | Trixeo Aerosphere 5micrograms/dose / 7.2micrograms/dose / 160micrograms/dose pressurised inhaler (AstraZeneca UK Ltd) |
| 5119 | CorticosteroidsInhaled | 39343511000001103 | Generic Trixeo Aerosphere 5micrograms/dose / 7.2micrograms/dose / 160micrograms/dose inhaler CFC free |
| 5119 | CorticosteroidsInhaled | 39360211000001103 | Fostair NEXThaler 100micrograms/dose / 6micrograms/dose dry powder inhaler (Pilsco Ltd) |
| 5119 | CorticosteroidsInhaled | 39360411000001104 | Sereflo 25micrograms/dose / 125micrograms/dose inhaler (Pilsco Ltd) |
| 5119 | CorticosteroidsInhaled | 39567411000001102 | Fixkoh Airmaster 50micrograms/dose / 500micrograms/dose dry powder inhaler (Thornton & Ross Ltd) |
| 5119 | CorticosteroidsInhaled | 39567611000001104 | Fixkoh Airmaster 50micrograms/dose / 250micrograms/dose dry powder inhaler (Thornton & Ross Ltd) |
| 5119 | CorticosteroidsInhaled | 39567811000001100 | Fixkoh Airmaster 50micrograms/dose / 100micrograms/dose dry powder inhaler (Thornton & Ross Ltd) |
| 5119 | CorticosteroidsInhaled | 39691211000001100 | Fluticasone propionate 100micrograms/dose dry powder inhaler (Imported) |
| 5119 | CorticosteroidsInhaled | 39695511000001104 | Beclometasone 100microgram inhalation powder capsules |
| 5119 | CorticosteroidsInhaled | 39695611000001100 | Beclometasone 200microgram inhalation powder capsules |
| 5119 | CorticosteroidsInhaled | 39695711000001109 | Beclometasone 400microgram inhalation powder capsules |
| 5119 | CorticosteroidsInhaled | 39696311000001100 | Salbutamol 400microgram / Beclometasone 200microgram inhalation powder capsules |
| 5119 | CorticosteroidsInhaled | 39712311000001105 | Fluticasone 500micrograms/2ml nebuliser liquid unit dose vials |
| 5119 | CorticosteroidsInhaled | 39712511000001104 | Fluticasone 2mg/2ml nebuliser liquid unit dose vials |
| 5119 | CorticosteroidsInhaled | 39817511000001103 | Luforbec 100micrograms/dose / 6micrograms/dose inhaler (Lupin Healthcare (UK) Ltd) |
| 5119 | CorticosteroidsInhaled | 39939611000001107 | Fostair 100micrograms/dose / 6micrograms/dose inhaler (CST Pharma Ltd) |
| 5119 | CorticosteroidsInhaled | 39993311000001105 | Trimbow NEXThaler 88micrograms/dose / 5micrograms/dose / 9micrograms/dose dry powder inhaler (Chiesi Ltd) |
| 5119 | CorticosteroidsInhaled | 40034211000001104 | Avenor 25micrograms/dose / 50micrograms/dose inhaler (Zentiva Pharma UK Ltd) |
| 5119 | CorticosteroidsInhaled | 40040711000001101 | Avenor 25micrograms/dose / 125micrograms/dose inhaler (Zentiva Pharma UK Ltd) |
| 5119 | CorticosteroidsInhaled | 40040911000001104 | Avenor 25micrograms/dose / 250micrograms/dose inhaler (Zentiva Pharma UK Ltd) |
| 5119 | CorticosteroidsInhaled | 40087411000001104 | Generic Trimbow NEXThaler 88micrograms/dose / 5micrograms/dose / 9micrograms/dose dry powder inhaler |
| 5119 | CorticosteroidsInhaled | 40106011000001102 | WockAIR 160micrograms/dose / 4.5micrograms/dose dry powder inhaler (Wockhardt UK Ltd) |
| 5119 | CorticosteroidsInhaled | 40106211000001107 | WockAIR 320micrograms/dose / 9micrograms/dose dry powder inhaler (Wockhardt UK Ltd) |
| 5119 | CorticosteroidsInhaled | 40220711000001106 | Seretide 50 Evohaler (CST Pharma Ltd) |
| 5119 | CorticosteroidsInhaled | 40220911000001108 | Fostair NEXThaler 100micrograms/dose / 6micrograms/dose dry powder inhaler (CST Pharma Ltd) |
| 5119 | CorticosteroidsInhaled | 40377811000001105 | Alvesco 160 inhaler (DE Pharmaceuticals) |
| 5119 | CorticosteroidsInhaled | 40444911000001101 | Seffalair Spiromax 12.75micrograms/dose / 202micrograms/dose dry powder inhaler (Teva UK Ltd) |
| 5119 | CorticosteroidsInhaled | 40445111000001100 | Seffalair Spiromax 12.75micrograms/dose / 100micrograms/dose dry powder inhaler (Teva UK Ltd) |
| 5119 | CorticosteroidsInhaled | 40455711000001101 | Fluticasone propionate 100micrograms/dose / Salmeterol 12.75micrograms/dose dry powder inhaler |
| 5119 | CorticosteroidsInhaled | 40455811000001109 | Fluticasone propionate 202micrograms/dose / Salmeterol 12.75micrograms/dose dry powder inhaler |
| 5119 | CorticosteroidsInhaled | 40504911000001103 | Sereflo Ciphaler 50micrograms/dose / 250micrograms/dose dry powder inhaler (Cipla EU Ltd) |
| 5119 | CorticosteroidsInhaled | 40752211000001109 | Trimbow 172micrograms/dose / 5micrograms/dose / 9micrograms/dose inhaler (Chiesi Ltd) |
| 5119 | CorticosteroidsInhaled | 40766811000001104 | Generic Trimbow 172micrograms/dose / 5micrograms/dose / 9micrograms/dose inhaler |
| 5119 | CorticosteroidsInhaled | 40852311000001103 | Luforbec 200micrograms/dose / 6micrograms/dose inhaler (Lupin Healthcare (UK) Ltd) |
| 5267 | Prednisolone | 325426006 | Prednisolone 1mg tablets |
| 5267 | Prednisolone | 325427002 | Prednisolone 5mg tablets |
| 5267 | Prednisolone | 325442004 | Prednisolone 2.5mg gastro-resistant tablets |
| 5267 | Prednisolone | 325443009 | Prednisolone 5mg gastro-resistant tablets |
| 5267 | Prednisolone | 325444003 | Prednisolone 5mg soluble tablets |
| 5267 | Prednisolone | 325445002 | Prednisolone 50mg tablets |
| 5267 | Prednisolone | 325450008 | Prednisolone 25mg tablets |
| 5267 | Prednisolone | 416533002 | Prednisolone 15mg/5ml oral solution |
| 5267 | Prednisolone | 429995001 | Prednisolone 10mg/5ml oral solution |
| 5267 | Prednisolone | 432224007 | Prednisolone 5mg/5ml oral suspension |
| 5267 | Prednisolone | 432225008 | Prednisolone 15mg/5ml oral suspension |
| 5267 | Prednisolone | 52911000001107 | Prednisolone 1mg tablets (Alliance Healthcare (Distribution) Ltd) |
| 5267 | Prednisolone | 85511000001101 | Prednisolone 5mg tablets (Accord Healthcare Ltd) |
| 5267 | Prednisolone | 86711000001103 | Prednisolone 5mg tablets (The Boots Company Plc) |
| 5267 | Prednisolone | 110811000001100 | Prednisolone 5mg tablets (Teva UK Ltd) |
| 5267 | Prednisolone | 113391000001105 | Prednisolone 5mg soluble tablets |
| 5267 | Prednisolone | 157711000001100 | Prednisolone 5mg soluble tablets (Alliance Healthcare (Distribution) Ltd) |
| 5267 | Prednisolone | 238511000001107 | Prednisolone 1mg tablets (Teva UK Ltd) |
| 5267 | Prednisolone | 245011000001108 | Prednisolone 5mg soluble tablets (A A H Pharmaceuticals Ltd) |
| 5267 | Prednisolone | 255211000001101 | Prednisolone 5mg gastro-resistant tablets (Alliance Healthcare (Distribution) Ltd) |
| 5267 | Prednisolone | 313911000001107 | Prednisolone 25mg tablets (Alliance Healthcare (Distribution) Ltd) |
| 5267 | Prednisolone | 331111000001100 | Deltacortril 5mg gastro-resistant tablets (Phoenix Labs Ltd) |
| 5267 | Prednisolone | 350611000001104 | Prednisolone 5mg tablets (Kent Pharma (UK) Ltd) |
| 5267 | Prednisolone | 380011000001100 | Prednisolone 2.5mg gastro-resistant tablets (Accord Healthcare Ltd) |
| 5267 | Prednisolone | 392511000001108 | Prednisolone 5mg soluble tablets (Advanz Pharma) |
| 5267 | Prednisolone | 447211000001105 | Prednisolone 5mg gastro-resistant tablets (Kent Pharma (UK) Ltd) |
| 5267 | Prednisolone | 459611000001100 | Deltacortril 2.5mg gastro-resistant tablets (Phoenix Labs Ltd) |
| 5267 | Prednisolone | 482891000001105 | Prednisolone 10mg/5ml oral solution |
| 5267 | Prednisolone | 512811000001108 | Prednisolone 5mg gastro-resistant tablets (Accord Healthcare Ltd) |
| 5267 | Prednisolone | 646211000001106 | Prednisolone 1mg tablets (Accord Healthcare Ltd) |
| 5267 | Prednisolone | 649711000001109 | Prednisolone 1mg tablets (A A H Pharmaceuticals Ltd) |
| 5267 | Prednisolone | 662011000001105 | Prednisolone 5mg gastro-resistant tablets (A A H Pharmaceuticals Ltd) |
| 5267 | Prednisolone | 685311000001104 | Prednisolone 2.5mg gastro-resistant tablets (Kent Pharma (UK) Ltd) |
| 5267 | Prednisolone | 707911000001100 | Prednisolone 1mg tablets (Kent Pharma (UK) Ltd) |
| 5267 | Prednisolone | 716111000001103 | Prednisolone 25mg tablets (A A H Pharmaceuticals Ltd) |
| 5267 | Prednisolone | 772111000001107 | Prednisolone 5mg tablets (Alliance Healthcare (Distribution) Ltd) |
| 5267 | Prednisolone | 779211000001109 | Prednisolone 2.5mg gastro-resistant tablets (Alliance Healthcare (Distribution) Ltd) |
| 5267 | Prednisolone | 798611000001108 | Prednisolone 1mg tablets (The Boots Company Plc) |
| 5267 | Prednisolone | 844111000001103 | Prednisolone 1mg tablets (Wockhardt UK Ltd) |
| 5267 | Prednisolone | 858811000001104 | Prednisolone 2.5mg gastro-resistant tablets (A A H Pharmaceuticals Ltd) |
| 5267 | Prednisolone | 876911000001107 | Prednisolone 5mg tablets (A A H Pharmaceuticals Ltd) |
[truncated: 14,688 more chars]
